# Novel Quinazoline Derivatives as Highly Effective A2A Adenosine Receptor Antagonists

**DOI:** 10.3390/molecules29163847

**Published:** 2024-08-14

**Authors:** Amélie Laversin, Robin Dufossez, Raphaël Bolteau, Romain Duroux, Séverine Ravez, Sergio Hernandez-Tapia, Martin Fossart, Mathilde Coevoet, Maxime Liberelle, Saïd Yous, Nicolas Lebègue, Patricia Melnyk

**Affiliations:** Univ. Lille, Inserm, CHU Lille, U1172—LilNCog—Lille Neuroscience & Cognition, F-59000 Lille, France; amelie.laversin@univ-lille.fr (A.L.); robin.dufossez@univ-lille.fr (R.D.); raphael.bolteau@hotmail.fr (R.B.); romain.duroux@gmail.com (R.D.); severine.ravez@univ-lille.fr (S.R.); sergioguillermo.hernandeztapia@univ-lille.fr (S.H.-T.); martin.fossart@inserm.fr (M.F.); mathilde.coevoet@univ-lille.fr (M.C.); maxime.liberelle@univ-lille.fr (M.L.); said.yous@univ-lille.fr (S.Y.); nicolas.lebegue@univ-lille.fr (N.L.)

**Keywords:** A_2A_ receptor antagonist, quinazoline, fluorescence polarization, cAMP assay

## Abstract

The adenosine A_2A_ receptor (A_2A_R) has been identified as a therapeutic target for treating neurodegenerative diseases and cancer. In recent years, we have highlighted the 2-aminoquinazoline heterocycle as an promising scaffold for designing new A_2A_R antagonists, exemplified by 6-bromo-4-(furan-2-yl)quinazolin-2-amine **1** (*K_i_* (*h*A_2A_R) = 20 nM). Here, we report the synthesis of new 2-aminoquinazoline derivatives with substitutions at the C6- and C7-positions, and the introduction of aminoalkyl chains containing tertiary amines at the C2-position to enhance antagonist activity and solubility properties. Compound **5m** showed a high affinity for *h*A_2A_R with a *K_i_* value of 5 nM and demonstrated antagonist activity with an IC_50_ of 6 µM in a cyclic AMP assay. Introducing aminopentylpiperidine and 4-[(piperidin-1-yl)methyl]aniline substituents maintained the binding affinities (**9x**, *K_i_* = 21 nM; **10d**, *K_i_* = 15 nM) and functional antagonist activities (**9x**, IC_50_ = 9 µM; **10d**, IC_50_ = 5 µM) of the synthesized compounds while improving solubility. This study provides insights into the future development of A_2A_R antagonists for therapeutic applications.

## 1. Introduction

**Figure 1:**
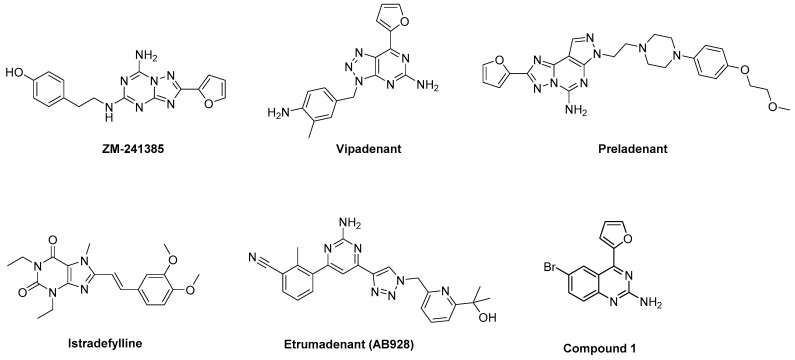
Structures of A_2A_R antagonists.

Adenosine A_2A_ receptors (A_2A_R) are membrane proteins that belong to the G protein-coupled adenosine receptor family, which includes four receptor subtypes: A_1_, A_2A_, A_2B_, and A_3_ [1]. Extracellular adenosine acts as the endogenous agonist for all adenosine receptors exerting diverse functions throughout the body [2]. Adenosine has a high affinity for A_2A_ receptors (*K_i_* (*h*A_2A_R) = 310 nM), and their activation leads to an increase in intracellular cAMP levels [3]. This rise in cAMP activates protein kinase A (PKA), which in turn phosphorylates the cAMP response element-binding protein (CREB). A_2A_Rs are highly expressed in the central nervous system, specifically in neurons, microglia, oligodendrocytes, and astrocytes [4,5]. Their presence is well documented in the dendritic spines and postsynaptic regions of the basal ganglia. These receptors are also prominently located in presynaptic regions, where they modulate the release of neurotransmitters such as glutamate, acetylcholine, GABA, and noradrenaline [6,7]. Epidemiological studies have demonstrated a link between caffeine consumption, a non-selective A_2A_ receptor antagonist, and a reduced risk of developing neurodegenerative diseases [8,9,10,11,12]. New selective and potent A_2A_R antagonists were initially developed for neurodegenerative diseases including Alzheimer’s disease (AD), Parkinson’s disease (PD), Huntington’s disease (HD), and attention-deficit/hyperactivity disorder (ADHD) [13,14,15,16]. In AD, the selective blockade of A_2A_Rs reduces memory impairment associated with amyloid burden and the development of Tau pathology [17]. Improvements are associated with a reduction in lesions and related neuroinflammation. Caffeine-treated cells and animal models have demonstrated a decrease in Tau protein phosphorylation [8,18,19]. The development of A_2A_R antagonists has led to the approval of istradefylline (Figure 1), a xanthine-like compound similar to caffeine, in Japan, Korea, and the USA as an adjunct therapy for PD in combination with levodopa [20]. Unfortunately, the development of other antagonists, such as ZM-241385, vipadenant, or preladenant (Figure 1), has often been discontinued due to insufficient efficacy associated with poor pharmacokinetic properties or toxic side effects [21]. In recent years, A_2A_R has emerged as a drug target for cancer immunotherapy, with its signaling identified as a novel immune checkpoint pathway. Studies have demonstrated that A_2A_Rs are over-expressed and exert immunosuppressive effects in the tumor microenvironment [22,23,24]. Some antagonists, such as etrumadenant (AB928), have shown significant antitumor effects in B16-F10 melanoma or AT3-OVA tumor models [25]. EXS21546 (pyrazolopyrimidine derivative, structure not disclosed) [26], developed by Evotec and Exscientia, reduces the viable fraction of tumor cells and increases the fraction and relative number of viable CD8+T cells in models using malignant pleural effusions [27]. This research has led to the discovery of novel A_2A_R antagonist structures, with some antagonists initially developed for neurodegenerative diseases being repurposed for cancer therapy [28].

**Figure 2:**
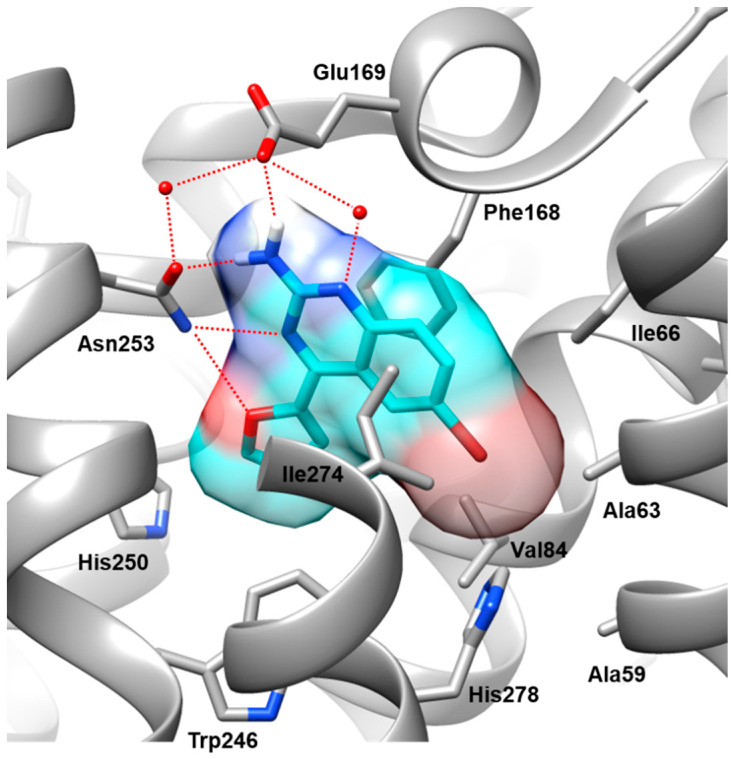
Co-crystallized structure of compound **1** bound to the *h*A_2A_ receptor (PDB code: 8DU3).

Our team previously identified 6-bromo-4-(furan-2-yl)quinazolin-2-amine (compound **1**) as a new quinazoline hit compound with high affinity for the adenosine A_2A_ receptor (*K_i_* (*h*A_2A_R) = 20 nM), as described in R. Bolteau et al. [29]. Co-crystallization of compound **1** bound to A_2A_R (PDB code: 8DU3) highlighted key interactions, including hydrogen bonds between the 2-aminoquinazoline ring and Asn253, Glu169 and water molecules, π-stacking interactions with Phe168, and hydrophobic interactions between the furan ring and His278 and Trp246 (Figure 2). Structure–activity relationship (SAR) studies focused on quinazoline’s 4-position revealed that 2-furanyl and 4-fluorophenyl rings are most compatible with the steric bulk of substituents. Building on these promising results, we report here the synthesis of new 2-aminoquinazolines derivatives with substitution at the C6- and C7-positions and the introduction of substituents containing hindered tertiary amines on the nitrogen at the C2-position to enhance antagonist activity and solubility properties. We have investigated their binding affinities and antagonist activities to establish a comprehensive SAR.

## 2. Results and Discussion

### 2.1. Chemistry

**Scheme 1:**
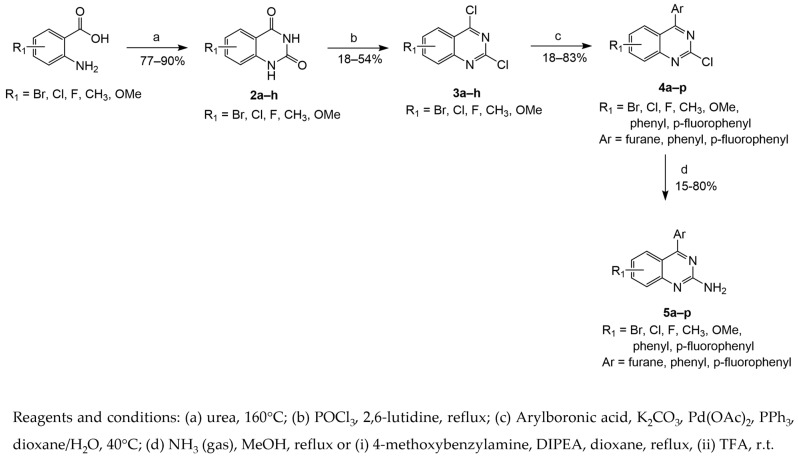
Synthesis of derivatives **5a**–**p**.

The synthesis of compounds **5a**–**p** is outlined in Scheme 1. First, quinazolinediones **2a**–**h** were obtained by heating commercially available anthranilic acid derivatives with urea until they melted [29]. Then, a chlorination reaction was performed by refluxing the mixture in POCl_3_ with a catalytic amount of 2,6-lutidine to afford 2,4-dichloroquinazolines **3a**–**h** [30]. A regioselective Suzuki reaction was then achieved at the 4-position with appropriate arylboronic acids under controlled conditions to provide 2-chloroquinazolines **4a**–**p** [31]. Even though chlorine in position 4 is more reactive than bromine in position 6, the Suzuki reaction on compound **3a** is not completely selective and yields di-arylated compounds **4i** and **4j**. Finally, 2-aminoquinazoline derivatives **5a**–**p** were synthesized either directly through nucleophilic substitution by refluxing in a sealed tube with a saturated solution of ammonia in methanol or via the introduction of 4-methoxybenzylamine followed by a debenzylation step in TFA [32].

**Scheme 2:**
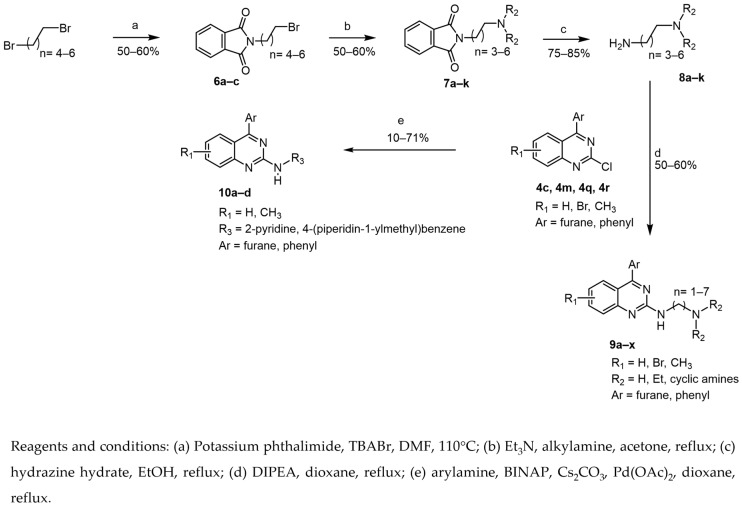
Synthesis of derivatives **9a**–**x** and **10a**–**d**.

Substituted 2-aminoquinazoline derivatives **9a**–**x** and **10a**–**d** were prepared as described in Scheme 2. The synthesis started with the substitution of potassium phthalimide using a large excess of the appropriate dibromoalkane in the presence of tetra-*n*-butylammonium bromide as a catalyst in DMF, providing bromine derivatives **6a**–**c**. Tertiary alkylamines **7a**–**k** were then synthesized via nucleophilic substitution with the corresponding amines in the presence of triethylamine in refluxing acetone with moderate yields. Hydrazinolysis allowed access to the primary alkylamines **8a**–**k**, which were condensed with 2-chloroquinazolines **4c**, **4m**, **4q**, and **4r** to afford the corresponding 2-aminoalkylquinazoline derivatives **9a**–**x**. Finally, aromatic amines were introduced by a Buchwald reaction, yielding the corresponding 2-aminoarylquinazolines **10a**–**d** [33].

### 2.2. Structure—Affinity Relationship Studies

Previously, binding studies of molecules on the *h*A_2A_ receptor were conducted using a competitive radioligand displacement assay using [^3^H]-ZM241385. However, radioligand binding (RB) assays are associated with high operating costs and significant health risks [34]. Consequently, these assays have been progressively replaced by fluorescence polarization (FP) assays for discovering new GPCR ligands and determining their binding affinities [35,36,37]. The binding affinities of quinazoline derivatives **5a**–**p** for the *h*A_2A_ receptor were subsequently determined using an FP assay using MRS7416 as the fluorescent probe [38]. The FP assay was developed in a 384-well plate format and yields *K_i_* values comparable to those obtained from the RB assay. For instance, the *K_i_* values of compounds **1** and **5c** were determined to be 20 ± 5 and 22 ± 7 nM using RB assay. Similarly, the FP assay quantified these values at 23 ± 7 and 45 ± 7 nM, respectively. As previously described, introducing a 2-furan moiety at the C4-position of the quinazoline, compared to a phenyl or a *p*-fluorophenyl group, resulted in the best binding affinities (Table 1). These results confirm that furan is the optimal heterocyclic substituent for aromatic hydrophobic interactions with Trp246 and His250, as well as for completing the hydrogen bonding network between furan oxygen and Asn253.

#### 2.2.1. Modulations at the C6-Position

Modulations at the C6-position were then performed. Replacing the bromine with small susbtituents, such as a methyl or chlorine group, maintained the affinity within the same order of magnitude (**5c**, *K_i_* = 45 nM; **5h**, *K_i_* = 28 nM) as reference compound 1 (*K_i_* = 23 nM). In contrast, larger substituents like phenyl or *p*-fluorophenyl significantly reduced or nearly abolished affinity (**5i**, *K_i_* > 10 µM; **5j**, *K_i_* = 318 nM). Interestingly, substitution with a small methoxy or fluorine group also completely abolished affinity (**5f** and **5g**, *K_i_* > 10 µM). These results clearly indicate that small hydrophobic motifs such as methyl, chlorine, or bromine are most suitable for interacting with the hydrophobic pocket formed by Ala59, Ala63, Val84, and His278.

#### 2.2.2. Modulations at the C7-Position

**Figure 3:**
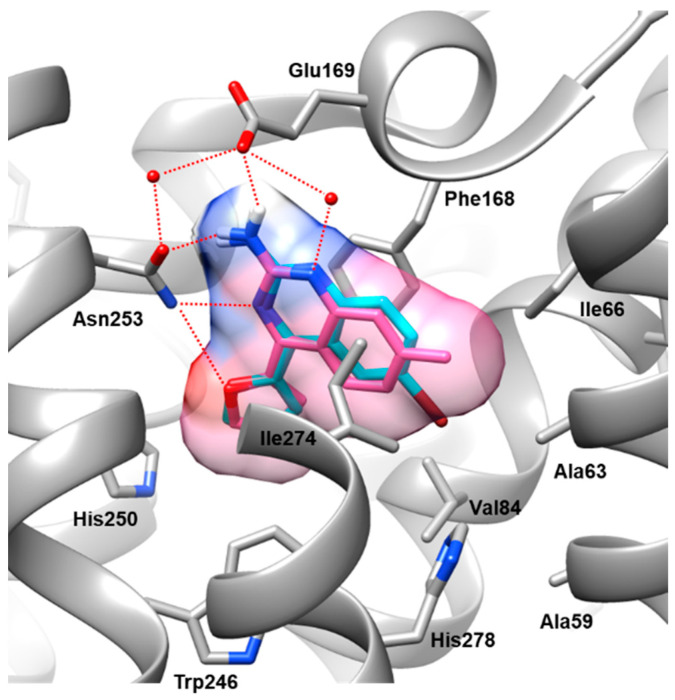
Predicted binding mode of the new hit **5m** (pink) within the co-crystallized structure of compound **1** (cyan) bound to the *h*A_2A_ receptor (PDB code: 8DU3).

Modifications were also introduced at the C7-position to investigate potential differences compared to the C6-position. Regardless of the substituent at the C4-position, the introduction of the optimal substituents such as bromine (**5k**), chlorine (**5p**), or methyl groups (**5m**) at the C7-position results in binding affinities comparable to those observed for the corresponding C6-substituted molecules. Interestingly, the C7 methyl-substituted compound **5m** exhibits an even higher affinity than its C-6 counterpart **5c** (*K_i_* = 45 nM) and reference compound **1** (*K_i_* = 23 nM), rendering it a highly potent ligand for A_2A_R (*K_i_* = 5 nM). Docking studies reveal that compound **5m** superimposes perfectly with compound **1** co-crystallized with the *h*A_2A_ receptor, forming the same network of hydrogen bonds. Focusing on the interactions involving the methyl group at the C7-position, it displays close van der Waals contacts with a hydrophobic pocket formed by Ala63, Ile66, and Ile274, improving binding affinity compared to brominated analogues (Figure 3). Taken together, these results show that the affinity of our compounds is mediated via the amine function of 2-aminoquinazoline and the furan ring, the same as for a number of antagonists described in the literature, such as ZM-241385, vipadenant, and preladenant. On the other hand, the binding mode of our molecules remains original, since very few of these ligands interact strongly by van der Waals contacts with the large hydrophobic pocket constituted by Ala59, Ala63, Ile66, Val84, and Ile274.

#### 2.2.3. Antagonist Activity

**Figure 4:**
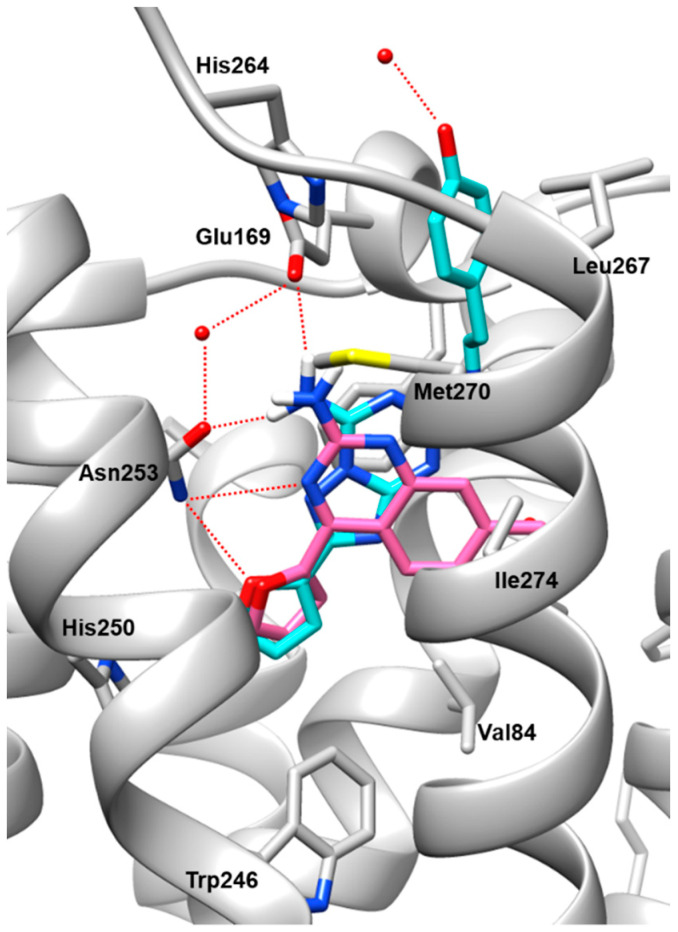
Predicted binding mode of the new hit **5m** (pink) within the co-crystallized structure of ZM-241385 (cyan) bound to the *h*A_2A_ receptor (PDB code: 3EML).

The functional activity of compounds with binding affinities below 70 nM were assessed using the Ultra Lance cAMP assay to measure the inhibition of *h*A_2A_ receptor-stimulated release of cAMP in HEK cells. The CGS21680 agonist was used at its EC_80_ concentration as recommended by the manufacturer. Inactive compound **5g** served as a negative control, while ZM-241385, with an IC_50_ of 0.1 µM, served as a positive control. This result was consistent with our previously obtained data [29]. Surprisingly, only compounds **5m** and **5p**, bearing a 7-methyl or 7-chloro substitution, demonstrated antagonist activities with half inhibitory concentrations (IC_50_) of 6 and 8 µM, respectively (Table 1). The other compounds showed no activity at concentrations above 30 µM. The discrepancy between the binding affinity results and the antagonist activities may be explained by the different binding modes of the molecules compared with ZM-241385, the reference selective A_2A_ adenosine antagonist (Figure 4). ZM-241385 shows the same interaction network as compound **5m** through the position of its primary amine and furan, but it also interacts with the top of the binding pocket. Indeed, the phenolic hydroxyl group of ZM-241385 forms a hydrogen bond with an ordered water molecule, while the phenyl ring engages hydrophobic interactions with His264, Leu267, and Met270. However, ZM-241385 does not interact with the hydrophobic pocket occupied by the substituted phenyl groups of quinazoline derivatives. In order to improve the antagonistic potency of these molecules, we considered introducing larger substituents, similar to those found on preladenant and ZM-241385 [39,40,41]. It is known that increasing the size and volume of GPCR ligands can promote a switch to full antagonist activity [42].

#### 2.2.4. Introduction of Amino Substituents at the C2-Position

To facilitate synthesis, a structure–activity relationship study was conducted on 2-amino-substituted quinazoline derivatives containing a phenyl group at the C4-position and no substituents at the C6- and C7-positions (Table 2) with compound **11** as the reference compound [28]. The introduction of 2-pyridine, benzylpiperidine, or benzylamine derivatives, as well as the ZM-241385 substituent 4-(2-aminoethyl)phenol, resulted in compounds with low (**9a**, *K_i_* = 1.1 µM and **9f**, *K_i_* = 1.4 µM) or no affinity (**9c**, **9d**, and **9e**, *K_i_* > 10 µM) for the *h*A_2A_ receptor. Subsequently, we investigated the introduction of aminoalkyl chains, varying both the spacer length and the nature of the tertiary amine. Initially, we studied the incorporation of a piperidine moiety separated from the 2-aminoquinazoline heterocycle by alkyl chains ranging from two to seven carbon atoms. Optimal binding activities were observed with spacer lengths of five and six carbon atoms (**9j**, *K_i_* = 294 nM; **9t**, *K_i_* = 233 nM), whereas further elongation reduced affinity (**9u**, *K_i_* = 834 nM). The chemical nature of the tertiary amine was then investigated. Replacing piperidine either with more polar six-membered ring analogues such as morpholine (**9k**, *K_i_* = 674 nM) or piperazine (**9m**, *K_i_* = 687 nM) and its derivatives (**9l**, *K_i_* = 790 nM; **9n**, *K_i_* = 1218 nM), or with a more constrained analogue such as pyrrolidine (**9o**, *K_i_* = 519 nM), significantly reduced affinity. However, piperidine could be replaced without loss of affinity by a less hydrophobic tertiary amine like diethylamine (**9p**, *K_i_* = 310 nM) or more hydrophobic ones like tetrahydroisoquinoline (**9q**, *K_i_* = 297 nM). The introduction of a more hydrophobic and sterically hindered benzylpiperidine reduced affinity to the micromolar range (**9r**, *K_i_* = 0.927 µM). Finally, the primary amine analogue of **9j** (**9s**, *K_i_* > 10 µM) lost all binding affinity. These findings show that while the presence of a tertiary amine is crucial for affinity, hydrophobicity and steric hindrance are tolerated to some extent. Interestingly, replacing the aminopentylpiperidine chain (**9j**, *K_i_* = 294 nM) with a constrained equivalent 4-[(piperidin-1-yl)methyl]aniline, maintaining a similar linear length of five carbons, significantly increased binding affinity toward the *h*A_2A_ receptor (**9b**, *K_i_* = 52 nM). This result reinforces the conclusion that an optimum spacer length is required and that the nature of the spacer, whether linear alkyl or more rigid aromatic, does not adversely affect binding affinity.

Both aminopentylpiperidine and 4-[(piperidin-1-yl)methyl]aniline groups were then introduced into 6- and 7-substituted 4-(furan-2-yl)quinazolin-2-amine derivatives, which exhibited the best binding activities (Table 3). Regardless of the chain type, compounds showed binding affinities around 60 nM for the 6-methyl-substituted derivatives (**9w**, *K_i_* = 61 nM; **10c**, *K_i_* = 65 nM) and between 15 and 20 nM for their 7-methyl counterparts (**9x**, *K_i_* = 21 nM; **10d**, *K_i_* = 15 nM). 

**Figure 5:**
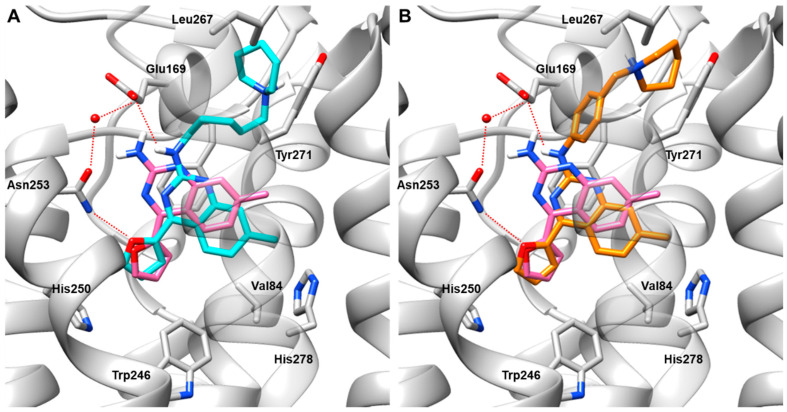
Predicted binding mode of (**A**) **5m** (pink) and **9x** (cyan) and (**B**) **5m** (pink) and **10d** (orange) within the *h*A_2A_ receptor (PDB code: 8DU3).

Docking studies were carried out to better understand the slight loss of affinity observed in compounds **9x** and **10d** following the introduction of these chains (Figure 5). Both compounds occupy the binding site similarly, with the aminoquinazoline ring shifting approximately 1.5 Å from its position in compound **5m**, resulting in a loss of interactions within the hydrogen bonding network. This loss is compensated by an increase in hydrophobic interactions through the piperidine motif, which is situated near Leu267 and Tyr271. These findings suggest that the introduction of hindered tertiary amines, such as diethylamine (**9p**), piperidine (**9j**), or tetrahydroisoquinoline (**9q**), enhances affinity more effectively than the introduction of a highly polar primary amine (**9s**). Surprisingly, the bromo derivative **9v** completely lost its affinity for the receptor. This suggests that the presence of a bulky bromine at C6-position prevents the molecule from correctly binding to the active site due to a steric clash with Val84. 

The hypothesis that increasing the size and volume of molecules to mimic the reference A_2A_ adenosine antagonists can enhance their antagonistic activities is not strongly supported by the findings. Although introducing a piperidine-containing chain slightly improved the antagonistic activity of compounds **9w** and **10c**, which IC_50_ around 10 µM compared with the 6-methyl-2-aminoquinazoline **5c** (IC_50_ > 30 µM), this improvement was not consistent for compounds **9x** (IC_50_ = 9 µM) and **10d** (IC_50_ = 5 µM). These compounds maintained their antagonistic activities close to the micromolar range, similar to compound **5m** (IC_50_ = 6 µM). Incorporating a tertiary amine functional group that can be protonated at physiological pH (7.4) can significantly enhance compound solubility. While 2-aminoquinazolines **1**, **5c**, and **5m** exhibit low (<200 µM) or moderate (<1 mM) solubilities, the introduction of piperidine chains increases their solubility to concentrations ranging from 2 to 14 mM. These results confirm that substitutions at the C2-position substitutions in quinazolines are beneficial, maintaining both affinity and activity, while also enhancing solubility.

## 3. Materials and Methods

### 3.1. Chemistry

Chemicals and solvents were obtained from commercial sources and used without further purification unless otherwise noted. Reactions were monitored by TLC performed on Macherey–Nagel Alugram^®^ Sil 60/UV254 sheets (thickness 0.2 mm, Macherey-Nagel GmbH & Co. KG, Düren, Germany). Purification of products was carried out by recrystallization or column chromatography. Column chromatography was carried out using Macherey–Nagel silica gel (230–400 mesh, Macherey-Nagel GmbH & Co. KG, Düren, Germany). Melting points were determined on a Büchi SMP-20 capillary apparatus (Büchi SARL, Villebon sur Yvette, France) and are uncorrected. NMR spectra were recorded on a Bruker DRX 300 spectrometer (Division Biospin, Wissembourg, France) operating at 300 MHz for ^1^H and 75 MHz for ^13^C). Chemical shifts are expressed in ppm relative to tetramethylsilane (TMS). Chemical shifts are reported as position (δ in ppm), multiplicity (s = singlet, d = doublet, t = triplet, q = quartet, p = pentet, dd = double doublet, br = broad, and m = multiplet), coupling constant (*J* in Hz), relative integral, and assignment. Mass spectra of compounds **2a**–**h**, **3a**–**h**, **4a**–**q**, **6a**–**c**, **7a**–**k**, and **8a**–**k** were recorded to unit accuracy with a LCMS (Waters Alliance Micromass ZQ 2000, Waters Corporation, Milford, MA, USA) with UV detection (PDA), an electrospray mode (ESI), and a Waters XBridge C18 column (5 μm particle size column, dimensions 50 mm × 4.6 mm, Waters Corporation, Milford, MA, USA). A gradient starting from 98% H_2_O/formate buffer 5 mM (pH 3.8) and reaching 100% CH3CN/formate buffer 5 mM (pH 3.8) within 4 min at a flow rate of 2 mL/min was used followed by a return to the starting conditions within 1 min. Mass spectra of compounds **5a**–**p**, **9a**–**x**, and **10a**–**d** were recorded with decimal precision using a Waters AcQuity UPLC I-Class with UV detection (PDA) and an electrospray mode (ESI) (Waters Corporation, Milford, MA, USA). UPLC-MS Waters system was equipped with a UPLC I SMP MGR-FTN sample manager, an ACQUITY UPLC I-Class eK photodiode array detector (210–400 nm), and an ACQUITY QDa (Performance) detector (scan 50–1250) (Waters Corporation, Milford, MA, USA). Acquity BEH C18 column (50 mm × 2.1 mm, 1.7 μm, Waters) was used. The injection volume was 0.5 μL. A mixture of water and acetonitrile was used as mobile phase in gradient elution. The pH of the mobile phase was adjusted with HCOOH and NH_4_OH to form a buffer solution at pH 3.8. The analysis time was 5 min (at a flow rate of 600 μL/min), 10 min (at a flow rate of 600 μL/min), or 30 min (at a flow rate of 600 μL/min). Unless otherwise specified, the purity of evaluated compounds was judged to be >95% as determined by UPLC-UV-MS system. 

NMR and LC-MS spectra of compounds **5a**–**p**, **9a**–**x** and **10a**–**d** are provided as supplementary materials (Figure S1).

General procedure for synthesis of compounds **2a**–**h**. The formation of quinazolinediones was carried out according to published procedures [29]. To a round-bottom flask were added the corresponding anthranilic acid (1.0 eq.) and urea (10.0 eq.). The mixture was stirred and heated at 160 °C overnight. Solid was cooled to 50 °C, and a 1M NaOH solution was added to dissolve the solid. The solution was filtered, filtrate was acidified with a 6M HCl solution up to acid pH and filtered again. Solid was washed with methanol to afford the corresponding quinazolinedione.

6-Bromoquinazoline-2,4-(1H,3H)-dione (**2a**). Yield: 8.9 g, 80%; yellow solid; m.p. > 300 °C. ^1^H NMR (300 MHz, DMSO-*d_6_*, δ ppm, *J* Hz): 11.44 (br s, 1H), 11.27 (br s, 1H), 7.95 (d, 1H, *J* = 2.3 Hz), 7.80 (dd, 1H, *J* = 2.3 Hz, *J* = 8.7 Hz), 7.13 (d, 1H, *J* = 8.7 Hz). LC-MS (ESI) *m*/*z* found: 239, 241 [M − H]^−^. 

6-Methylquinazoline-2,4-(1H,3H)-dione (**2b**). Yield: 8.5 g, 73%; white solid; m.p. > 300 °C. ^1^H NMR (300 MHz, DMSO-*d_6_*, δ ppm, *J* Hz): 11.20–11.17 (m, 2H), 7.68 (d, 1H, *J* = 1.5 Hz), 7.43 (dd, 1H, *J* = 2.1 Hz, *J* = 8.3 Hz), 7.10 (d, 1H, *J* = 8.3 Hz). LC-MS (ESI) *m*/*z* found: 175 [M − H]^−^.

6-Methoxyquinazoline-2,4-(1H,3H)-dione (**2c**). Yield: 5.1 g, 89%; yellow solid; m.p. > 300 °C. ^1^H NMR (300 MHz, DMSO-*d_6_*, δ ppm, *J* Hz): 11.16–11.14 (m, 2H), 7.32 (d, 1H, *J* = 2.9 Hz), 7.43 (dd, 1H, *J* = 2.9 Hz, *J* = 8.9 Hz), 7.13 (d, 1H, *J* = 8.8 Hz), 3.78 (s, 3H). LC-MS (ESI) *m*/*z* found: 191 [M − H]^−^.

6-Fluoroquinazoline-2,4-(1H,3H)-dione (**2d**). Yield: 8.9 g, 77%; yellow solid; m.p. > 300 °C. ^1^H NMR (300 MHz, DMSO-*d_6_*, δ ppm, *J* Hz): 11.20–11.18 (m, 2H), 7.68 (d, 1H, *J* = 1.5 Hz), 7.43 (dd, 1H, *J* = 2.1 Hz, *J* = 8.3 Hz), 7.10 (d, 1H, *J* = 8.3 Hz). LC-MS (ESI) *m*/*z* found: 179 [M − H]^−^.

6-Chloroquinazoline-2,4-(1H,3H)-dione (**2e**). Yield: 10.0 g, 87%; yellow solid; m.p. > 300 °C. ^1^H NMR (300 MHz, DMSO-*d_6_*, δ ppm, *J* Hz): 11.28 (m, 2H), 7.79 (d, 1H, *J* = 2.5 Hz), 7.67 (dd, 1H, *J* = 2.5 Hz, *J* = 8.7 Hz), 7.19 (d, 1H, *J* = 8.8 Hz). LC-MS (ESI) *m*/*z* found: 196, 198 [M − H]^−^.

7-Bromoquinazoline-2,4-(1H,3H)-dione (**2f**). Yield: 9.1 g, 82%; yellow solid; m.p. > 300 °C. ^1^H NMR (300 MHz, DMSO-*d_6_*, δ ppm, *J* Hz): 11.40 (br s, 1H), 11.25 (br s, 1H), 7.95 (d, 1H, *J* = 2.3 Hz), 7.80 (dd, 1H, *J* = 2.3 Hz, *J* = 8.7 Hz), 7.13 (d, 1H, *J* = 8.7 Hz). LC-MS (ESI) *m*/*z* found: 239, 241 [M − H]^−^.

7-Methylquinazoline-2,4-(1H,3H)-dione (**2g**). Yield: 5.0 g, 86%; white solid; m.p. > 300 °C. ^1^H NMR (300 MHz, DMSO-*d_6_*, δ ppm, *J* Hz): 11.33–11.29 (m, 2H), 7.68 (d, 1H, *J* = 1.9 Hz), 7.43 (dd, 1H, *J* = 2.2 Hz, *J* = 8.3 Hz), 7.10 (d, 1H, *J* = 8.3 Hz). LC-MS (ESI) *m*/*z* found: 175 [M − H]^−^.

7-Chloroquinazoline-2,4-(1H,3H)-dione (**2h**). Yield: 10.0 g, 87%; yellow solid; m.p. > 300 °C. ^1^H NMR (300 MHz, DMSO-*d_6_*, δ ppm, *J* Hz): 11.41 (s, 1H), 11.26 (s, 1H), 7.87 (d, 1H, *J* = 8.4 Hz), 7.21 (dd, 1H, *J* = 1.9 Hz, *J* = 8.7 Hz), 7.17 (d, 1H, *J* = 1.6 Hz). LC-MS (ESI) *m*/*z* found: 196, 198 [M − H]^−^.

General procedure for synthesis of compounds **3a**–**h**. The formation of dichloroquinazolines was carried out according to published procedures [30]. To a solution of the corresponding quinazolinedione (1.0 eq.) in POCl_3_ (10.0 eq.) was added 2,6-lutidine (1.0 eq.). The solution was stirred and heated at reflux overnight. The mixture was cooled to room temperature and concentrated in vacuo. The residue was dissolved in chloroform, and the solution was stirred for 5 min. Ice was added, and aqueous layer was extracted three times with chloroform. Combined organic layers were dried over MgSO_4_, filtered, and concentrated in vacuo. Crude product was purified by flash chromatography (cyclohexane/acetone (10/0 to 9/1)) to afford the corresponding dichloroquinazoline.

6-Bromo-2,4-dichloroquinazoline (**3a**). Yield: 2.4 g, 41%; white solid; m.p. 153 °C. ^1^H NMR (300 MHz, DMSO-*d_6_*, δ ppm, *J* Hz): 8.45 (d, 1H, *J* = 2.3 Hz), 8.08 (dd, 1H, *J* = 2.2 Hz *J* = 9.0 Hz), 7.91 (d, 1H, *J* = 8.9 Hz). LC-MS (ESI) *m*/*z* found: 277, 279, 281 [M + H]^+^.

2,4-Dichloro-6-methylquinazoline (**3b**). Yield: 2.4 g, 40%; white solid; m.p. 135–138 °C. ^1^H NMR (300 MHz, DMSO-*d_6_*, δ ppm, *J* Hz): 8.05 (m, 1H), 8.02 (dd, 1H, *J* = 1.8 Hz, *J* = 8.7 Hz), 7.94 (d, 1H, *J* = 8.6 Hz), 2.54 (s, 3H). LC-MS (ESI) *m*/*z* found: 213, 215 [M + H]^+^.

2,4-Dichloro-6-methoxyquinazoline (**3c**). Yield: 850 mg, 18%; yellow solid; m.p. 170–171 °C. ^1^H NMR (300 MHz, DMSO-*d_6_*, δ ppm, *J* Hz): 7.98 (d, 1H, *J* = 9.3 Hz), 7.80 (dd, 1H, *J* = 2.9 Hz, *J* = 9.2 Hz), 7.47 (d, 1H, *J* = 2.9 Hz), 3.99 (s, 3H). LC-MS (ESI) *m*/*z* found: 229, 231 [M + H]^+^.

2,4-Dichloro-6-fluoroquinazoline (**3d**). Yield: 2.4 g, 40%; yellow solid; m.p. 136–137 °C. ^1^H NMR (300 MHz, DMSO-*d_6_*, δ ppm, *J* Hz): 8.08 (m, 2H), 8.15 (m, 1H). LC-MS (ESI) *m*/*z* found: 217, 219 [M + H]^+^.

2,4-Dichloro-6-chloroquinazoline (**3e**). Yield: 1.7 g, 29%; yellow solid; m.p. 126–128 °C. ^1^H NMR (300 MHz, DMSO-*d_6_*, δ ppm, *J* Hz): 8.08 (m, 2H), 8.15 (m, 1H). LC-MS (ESI) *m*/*z* found: 233, 235 [M + H]^+^.

7-Bromo-2,4-dichloroquinazoline (**3f**). Yield: 2.0 g, 35%; white solid; m.p. 189–191 °C. ^1^H NMR (300 MHz, DMSO-*d_6_*, δ ppm, *J* Hz): 8.36 (d, 1H, *J* = 1.9 Hz), 8.23 (d, 1H, *J* = 8.9 Hz), 8.05 (dd, 1H, *J* = 1.9 Hz, *J* = 8.9 Hz). LC-MS (ESI) *m*/*z* found: 277, 279, 281 [M + H]^+^.

2,4-Dichloro-7-methylquinazoline (**3g**). Yield: 2.0 g, 54%; white solid; m.p. 142 °C. ^1^H NMR (300 MHz, CDCl_3_, δ ppm, *J* Hz): 8.15 (d, 1H, *J* = 8.5 Hz), 7.80–7.77 (m, 1H), 7.60–7.55 (m, 1H), 2.64 (s, 3H). LC-MS (ESI) *m*/*z* found: 213, 215 [M + H]^+^.

2,4-Dichloro-7-chloroquinazoline (**3h**). Yield: 1.3 g, 23%; yellow solid; m.p. 130–132 °C. ^1^H NMR (300 MHz, DMSO-*d_6_*, δ ppm, *J* Hz): 7.86 (d, 1H, *J* = 8.5 Hz), 7.25 (d, 1H, *J* = 2.0 Hz), 7.47 (dd, 1H, *J* = 2.0 Hz, *J* = 8.6 Hz). LC-MS (ESI) *m*/*z* found: 233, 235 [M + H]^+^.

General procedure for synthesis of compounds **4a**–**q**.

The formation of dichloroquinazolines was carried out according to published procedures [31]. To a tube were added the corresponding dichloroquinazoline derivatives (1.0 eq.), K_2_CO_3_ (2.0 eq.), and triphenylphosphine (0.04 eq.) and the corresponding boronic acid (1.2 eq.) in dioxane/water (4/1). The solution was degassed for 5 min with a nitrogen flow, and then palladium diacetate (0.02 eq.) was added, and the tube was sealed. The mixture was stirred and heated at 40 °C overnight. The solution was cooled to room temperature and hydrolyzed with water. Aqueous layer was extracted three times with EtOAc. Combined organic layers were dried over MgSO_4_, filtered, and concentrated in vacuo. Crude product was purified by flash chromatography (cyclohexane/EtOAc (10/0 to 9/1)) to afford compounds **4a**–**p**.

6-Bromo-2-chloro-4-(4-fluorophenyl)quinazoline (**4a**). Yield: 214 mg, 35%; white solid; m.p. 197–198 °C. ^1^H NMR (300 MHz, CDCl_3_, δ ppm, *J* Hz): 8.26 (d, 1H, *J* = 1.8 Hz), 8.04 (dd, 1H, *J* = 1.9 Hz, *J* = 9.0 Hz), 7.95 (d, 1H, *J* = 9.0 Hz), 7.83 (m, 2H), 7.32 (m, 2H). LC-MS (ESI) *m*/*z* found: 339, 341, 343 [M + H]^+^.

6-Bromo-2-chloro-4-phenylquinazoline (**4b**). Yield: 811 mg, 47%; white solid; m.p. 160–162 °C. ^1^H NMR (300 MHz, CDCl_3_, δ ppm, *J* Hz): 8.25 (dd, 1H, *J* = 2.2 Hz, *J* = 9.0 Hz), 8.17 (d, 1H, *J* = 1.9 Hz), 8.02 (d, 1H, *J* = 9.0 Hz), 7.82 (m, 2H), 6.67 (m, 3H). LC-MS (ESI) *m*/*z* found: 319, 321, 322 [M + H]^+^.

2-Chloro-4-(furan-2-yl)-6-methylquinazoline (**4c**). Yield: 840 mg, 73%; yellow solid; m.p. 124 °C. ^1^H NMR (300 MHz, CDCl_3_, δ ppm, *J* Hz): 8.59 (m, 1H), 8.23 (dd, 1H, *J* = 0.6 Hz, *J* = 1.7 Hz), 7.90 (dd, 1H, *J* = 1.9 Hz, *J* = 8.8 Hz), 7.85 (d, 1H, *J* = 8.6 Hz), 7.71 (dd, 1H, *J* = 0.6 Hz, *J* = 3.7 Hz), 6.88 (dd, 1H, *J* = 1.7 Hz, *J* = 3.7 Hz), 2.56 (s, 3H). LC-MS (ESI) *m*/*z* found: 245, 247 [M + H]^+^.

2-Chloro-4-(4-fluorophenyl)-6-methylquinazoline (**4d**). Yield: 1.2 g, 68%; white solid; m.p. 211–213 °C. ^1^H NMR (300 MHz, CDCl_3_, δ ppm, *J* Hz): 7.97 (d, 1H, *J* = 8.7 Hz), 7.82 (m, 4H), 7.30 (m, 2H), 2.55 (s, 3H). LC-MS (ESI) *m*/*z* found: 273, 275 [M + H]^+^.

2-Chloro-6-methyl-4-phenylquinazoline (**4e**). Yield: 300 mg, 63%; white solid; m.p. 154–155 °C. ^1^H NMR (300 MHz, CDCl_3_, δ ppm, *J* Hz): 7.81 (m, 5H), 7.65 (m, 2H), 6.50 (1H), 2.48 (s, 3H). LC-MS (ESI) *m*/*z* found: 255, 257 [M + H]^+^.

2-Chloro-4-(furan-2-yl)-6-methoxyquinazoline (**4f**). Yield: 470 mg, 83%; green solid. ^1^H NMR (300 MHz, CDCl_3_, δ ppm, *J* Hz): 8.22 (m, 1H), 7.91 (dd, 1H, *J* = 0.6 Hz, *J* = 1.7 Hz), 7.83 (dd, 1H, *J* = 1.9 Hz, *J* = 8.8 Hz), 7.66 (d, 1H, *J* = 8.6 Hz), 7.56 (dd, 1H, *J* = 0.6 Hz, *J* = 3.7 Hz), 6.73 (dd, 1H, *J* = 1.7 Hz, *J* = 3.7 Hz), 4.02 (s, 3H). LC-MS (ESI) *m*/*z* found: 261, 263 [M + H]^+^.

2-Chloro-4-(furan-2-yl)-6-fluoroquinazoline (**4g**). Yield: 248 mg, 54%; yellow solid. ^1^H NMR (300 MHz, CDCl_3_, δ ppm, *J* Hz): 8.64 (dd, 1H, *J* = 2.9 Hz, *J* = 9.8 Hz), 8.02 (dd, 1H, *J* = 5.4 Hz, *J* = 9.4 Hz), 7.85 (dd, 1H, *J* = 0.9 Hz, *J* = 1.8 Hz), 7.72 (m, 2H), 6.74 (dd, 1H, *J* = 1.6 Hz, *J* = 3.6 Hz). LC-MS (ESI) *m*/*z* found: 249, 251 [M + H]^+^.

2-Chloro-4-(furan-2-yl)-6-chloroquinazoline (**4h**). Yield: 250 mg, 44%; yellow solid; 209–211 °C. ^1^H NMR (300 MHz, CDCl_3_, δ ppm, *J* Hz): 8.98 (dd, 1H, *J* = 0.5 Hz, *J* = 2.2 Hz), 7.93 (dd, 1H, *J* = 0.5 Hz, *J* = 9.1 Hz), 7.87 (m, 2H), 7.74 (dd, 1H, *J* = 0.8 Hz, *J* = 3.6 Hz), 6.74 (dd, 1H, *J* = 1.8 Hz, *J* = 3.7 Hz). LC-MS (ESI) *m*/*z* found: 265, 267, 269 [M + H]^+^.

2-Chloro-4,6-diphenylquinazoline (**4i**). Yield: 212 mg, 62%; yellow solid; m.p. 168–170 °C. ^1^H NMR (300 MHz, CDCl_3_, δ ppm, *J* Hz): 8.32 (m, 2H), 8.24 (m, 1H), 7.89–7.86 (m, 2H), 7.65–7.62 (m, 5H), 7.54–7.44 (m, 4H). LC-MS (ESI) *m*/*z* found: 317, 319 [M + H]^+^.

2-Chloro-4,6-bis(4-fluorophenyl)quinazoline (**4j**). Yield: 787 mg, 62%; yellow solid; m.p. 185 °C. ^1^H NMR (300 MHz, DMSO-*d_6_*, δ ppm, *J* Hz): 7.34 (m, 2H), 7.49 (m, 2H), 7.81 (m, 2H), 7.97 (m, 2H), 8.12 (d, 1H, *J* = 8.8 Hz), 8.18 (d, 1H, *J* = 1.7 Hz), 8.40 (dd, 1H, *J* = 2.1 Hz, 8.8 Hz). LC-MS (ESI) *m*/*z* found: 353, 355 [M + H]^+^.

7-Bromo-2-chloro-4-(2-furan)quinazoline (**4k**). Yield: 200 mg, 18%; yellow solid; m.p. 129–131 °C. ^1^H NMR (300 MHz, CDCl_3_, δ ppm, *J* Hz): 9.14 (d, 1H, *J* = 2.1 Hz), 8.00 (dd, 1H, *J* = 2.1 Hz, *J* = 9.0 Hz), 7.89 (m, 2H), 7.75 (dd, 1H, *J* = 0.8 Hz, *J* = 3.6 Hz), 6.74 (dd, 1H, *J* = 1.6 Hz, *J* = 3.6 Hz). LC-MS (ESI) *m*/*z* found: 309, 311, 313 [M + H]^+^.

7-Bromo-2-chloro-4-(4-fluorophenyl)quinazoline (**4l**). Yield: 204 mg, 31%; white solid; m.p. 197–198 °C. ^1^H NMR (300 MHz, CDCl_3_, δ ppm, *J* Hz): 8.26 (d, 1H, *J* = 1.8 Hz), 8.04 (dd, 1H, *J* = 1.9 Hz, *J* = 9.0 Hz), 7.95 (d, 1H, *J* = 9.0 Hz), 7.83 (m, 2H), 7.32 (m, 2H). LC-MS (ESI) *m*/*z* found: 339, 341, 343 [M + H]^+^.

2-Chloro-4-(furan-2-yl)-7-methylquinazoline (**4m**). Yield: 140 mg, 50%; yellow solid; m.p. 184–186 °C. ^1^H NMR (300 MHz, CDCl_3_, δ ppm, *J* Hz): 8.82 (d, 1H, *J* = 8.7 Hz), 7.83 (m, 1H), 7.76 (m, 1H), 7.67 (d, 1H, *J* = 3.7 Hz), 7.52 (dd, 1H, *J* = 1.4 Hz, *J* = 8.7 Hz), 6.72 (dd, 1H, *J* = 1.5 Hz, *J* = 3.5 Hz), 2.62 (s, 3H). LC-MS (ESI) *m*/*z* found: 245, 247 [M + H]^+^.

2-Chloro-4-(4-fluorophenyl)-7-methylquinazoline (**4n**). Yield: 300 mg, 78%; white solid; m.p. 161–163 °C. ^1^H NMR (300 MHz, CDCl_3_, δ ppm, *J* Hz): 8.00 (d, 1H, *J* = 8.6 Hz), 7.86–7.79 (m, 3H), 7.50–7.45 (m, 1H), 7.34–7.25 (m, 2H), 2.64 (s, 3H). LC-MS (ESI) *m*/*z* found: 273, 275 [M + H]^+^.

2-Chloro-4-phenyl-7-methylquinazoline (**4o**). Yield: 300 mg, 78%; white solid; m.p. 256–258 °C. ^1^H NMR (300 MHz, CDCl_3_, δ ppm, *J* Hz): 8.00 (d, 1H, *J* = 8.6 Hz), 7.86–7.79 (m, 3H), 7.50–7.45 (m, 1H), 7.34–7.25 (m, 2H), 2.64 (s, 3H). LC-MS (ESI) *m*/*z* found: 273, 275 [M + H]^+^.

2-Chloro-4-(furan-2-yl)-7-chloroquinazoline (**4p**). Yield: 200 mg, 44%; yellow solid; 133–135 °C. ^1^H NMR (300 MHz, CDCl_3_, δ ppm, *J* Hz): 8.92 (d, 1H, *J* = 9.2 Hz), 7.96 (d, 1H, *J* = 2.2 Hz), 7.85 (m, 1H), 7.74 (d, 1H, *J* = 3.8 Hz), 7.64 (dd, 1H, *J* = 2.2 Hz, *J* = 9.2 Hz), 6.73 (dd, 1H, *J* = 1.8 Hz, *J* = 3.6 Hz). LC-MS (ESI) *m*/*z* found: 265, 267, 269 [M + H]^+^.

The formation of 2-chloro-4-phenylquinazoline (**4q**) and 6-bromo-2-chloro-4-(furan-2-yl)quinazoline (**4r**) was carried out according to published procedures [43].

General procedure for synthesis of compounds **5a**–**d**, **5i**–**l**, **5n**–**o**.

To a tube was added the corresponding chloroquinazoline in MeOH solution saturated with ammonia and the tube was immediately sealed. The solution was stirred at reflux overnight. The solution was cooled to room temperature and solid was filtered. Crude product was purified by flash chromatography (cyclohexane/EtOAc (5/5)) to afford the corresponding aminoquinazoline.

2-Amino-6-bromo-4-(4-fluorophenyl)quinazoline (**5a**). Yield: 28 mg, 15%; yellow solid; m.p. 200–203 °C. ^1^H NMR (300 MHz, DMSO-*d_6_*, δ ppm, *J* Hz): 7.81 (m, 5H), 7.44 (m, 3H), 7.17 (s, 2H). ^13^C NMR (300 MHz, DMSO-*d_6_*, δ ppm, *J* Hz): 168.4 (C), 165.2 (C), 161.9 (C), 160.5 (C), 151.9 (C), 137.2 (CH), 133.2 (CH), 132.3 (CH), 132.2 (CH), 129.1 (CH), 127.8 (CH), 118.9 (CH), 116.1 (CH), 114.3 (C). LC-MS (ESI) *m*/*z* found: 317.9, 319.9 [M + H]^+^. 

2-Amino-6-bromo-4-phenylquinazoline (**5b**). Yield: 50 mg, 20%; yellow solid; m.p. 204–207 °C. ^1^H NMR (300 MHz, DMSO-*d_6_*, δ ppm, *J* Hz): 7.81 (dd, 1H, *J* = 2.2 Hz, *J* = 8.9 Hz), 7.75 (d, 1H, *J* = 2.2 Hz), 7.68 (m, 2H), 7.60 (m, 3H), 7.46 (d, 1H, *J* = 8.9 Hz), 7.09 (s, 2H). ^13^C NMR (300 MHz, DMSO-*d_6_*, δ ppm, *J* Hz): 169.2 (C), 160.9 (C), 152.5 (C), 137.0 (CH), 136.86 (CH), 130.4 (C), 129.7 (CH), 129.1 (2 CH), 129.0 (CH), 128.2 (2 CH), 118.9 (C), 113.9 (C). LC-MS (ESI) *m*/*z* found: 299.9, 301.9 [M + H]^+^. 

4-(Furan-2-yl)-6-methylquinazolin-2-amine (**5c**). Yield: 30 mg, 38%; yellow solid; m.p. 157–159 °C. ^1^H NMR (300 MHz, CDCl_3_, δ ppm, *J* Hz): 8.25 (m, 1H), 8.09 (m, 1H), 7.56 (dd, 1H, *J* = 2.1 Hz, *J* = 8.8 Hz), 7.42–7.38 (m, 2H), 6.80 (dd, 1H, *J* = 1.9 Hz, *J* = 3.5 Hz), 6.71 (s, 2H), 2.43 (s, 3H). ^13^C NMR (300 MHz, CDCl_3_, δ ppm, *J* Hz): 165.0 (C), 160.7 (C), 157.6 (C), 157.2 (C), 151.4 (C), 140.9 (CH), 136.7 (CH), 130.6 (CH), 130.1 (C), 120.7 (CH), 120.4 (CH), 117.5 (CH), 26.3 (CH_3_). LC-MS (ESI) *m*/*z* found: 226.2 [M + H]^+^. 

2-Amino-4-(4-fluorophenyl)-6-methylquinazoline (**5d**). Yield: 37 mg, 10%; yellow solid; m.p. 180–182 °C. ^1^H NMR (300 MHz, DMSO-*d_6_*, δ ppm, *J* Hz): 7.70 (m, 2H), 7.53 (m, 1H), 7.44 (m, 4H), 6.78 (s, 2H, NH_2_), 3.07 (s, 3H). ^13^C NMR (300 MHz, DMSO-*d_6_*, δ ppm, *J* Hz): 168.1 (C), 164.1 (C), 161.6 (C), 160.2 (C), 152.1 (C), 136.2 (CH), 134.0 (CH), 132.1 (2 CH), 131.6 (C), 125.8 (CH), 117.6 (C), 115.2 (2 CH), 21.3 (CH_3_). LC-MS (ESI) *m*/*z* found: 254.0 [M + H]^+^. 

4,6-Di(4-fluorophenyl)quinazolin-2-amine (**5i**). Yield: 12 mg, 46%; white solid; m.p. 239–241 °C. ^1^H NMR (300 MHz, DMSO-*d_6_*, δ ppm, *J* Hz): 8.32–8.29 (m, 2H), 8.21–8.11 (m, 3H), 8.06–8.02 (m, 2H), 8.01–7.66 (m, 4H). ^13^C NMR (300 MHz, DMSO-*d_6_*, δ ppm, *J* Hz): 169.0 (C), 165.1 (C), 161.5 (C), 160.7 (C), 160.6 (C), 153.2 (CH), 136.6 (C), 133.8 (C), 133.3 (C), 132.3 (C), 129.1 (2 CH), 126.5 (CH), 124.6 (CH), 117.8 (C), 116.4 (CH), 116.1 (2 CH), 115.8 (2 CH). LC-MS (ESI) *m*/*z* found: 334.2 [M + H]^+^. 

4,6-Diphenylquinazolin-2-amine (**5j**). Yield: 12 mg, 32%; yellow solid; m.p. 214–216 °C. ^1^H NMR (300 MHz, CDCl_3_, δ ppm, *J* Hz): 8.28–8.03 (m, 3H), 7.94–7.82 (m, 2H), 7.77–7.60 (m, 4H), 7.59–7.38 (m, 4H). ^13^C NMR (300 MHz, CDCl_3_, δ ppm, *J* Hz): 154.5 (C), 139.8 (C), 138.5 (C), 137.0 (C), 135.0 (C), 132.4 (CH), 130.0 (CH), 129.3 (CH), 129.1 (2 CH), 128.8 (2 CH), 127.3 (2 CH), 126.7 (2 CH), 119.1 (CH), 117.5 (C). LC-MS (ESI) *m*/*z* found: 298.1 [M + H]^+^. 

7-bromo-4-(furan-2-yl)quinazolin-2-amine (**5k**). Yield: 29 mg, 21%; yellow solid; m.p. 193–195 °C. ^1^H NMR (300 MHz, DMSO-*d_6_*, δ ppm, *J* Hz): 7.97 (d, 1H, *J* = 2.1 Hz), 7.80 (dd, 1H, *J* = 2.0 Hz, *J* = 9.0 Hz), 7.74–7.71 (m, 2H), 7.57 (d, 1H, *J* = 8.9 Hz), 7.32–7.29 (m, 2H), 5.53 (s, 2H). ^13^C NMR (300 MHz, DMSO-*d_6_*, δ ppm, *J* Hz): 169.1 (C), 165.1 (C), 163.0 (C), 159.2 (C), 137.6 (C), 132.6 (CH), 132.1 (2 CH), 131.6 (CH), 129.3 (CH), 127.4 (C), 119.4 (C), 116.3 (CH), 116.1 (CH). LC-MS (ESI) *m*/*z* found: 290.1, 292.1 [M + H]^+^. 

2-Amino-7-bromo-4-(4-fluorophenyl)quinazoline (**5l**). Yield: 35 mg, 13%; yellow solid; m.p. 211–213 °C. ^1^H NMR (300 MHz, CDCl_3_, δ ppm, *J* Hz): 7.97 (d, 1H, *J* = 2.1 Hz), 7.80 (dd, 1H, *J* = 2.0 Hz, *J* = 9.0 Hz), 7.74–7.71 (m, 2H), 7.57 (d, 1H, *J* = 8.9 Hz), 7.32–7.29 (m, 2H), 5.53 (s, 2H). ^13^C NMR (300 MHz, CDCl_3_, δ ppm, *J* Hz): 169.1 (C), 165.1 (C), 163.0 (C), 159.2 (C), 137.6 (C), 132.6 (CH), 132.1 (2 CH), 131.6 (CH), 129.3 (CH), 127.4 (C), 119.4 (C), 116.3 (CH), 116.1 (CH). LC-MS (ESI) *m*/*z* found: 318.0, 320.1 [M + H]^+^. 

4-(4-Fluorophenyl)-7-methylquinazolin-2-amine (**5n**). Yield: 121 mg, 65%; white solid; m.p. 161 °C. ^1^H NMR (300 MHz, CDCl_3_, δ ppm, *J* Hz): 7.76–7.69 (m, 3H), 7.47–7.44 (m, 1H), 7.27–7.22 (m, 2H), 7.09 (dd, 1H, *J* = 1.4 Hz, *J* = 8.5 Hz), 5.22 (br s, 2H), 2.53 (s, 3H). ^13^C NMR (300 MHz, CDCl_3_, δ ppm, *J* Hz): 168.9 (C), 165.4 (C), 162.1 (C), 159.6 (C), 153.5 (C), 145.1 (C), 131.6 (CH), 131.5 (CH), 126.9 (CH), 125.5 (CH), 125.1 (CH), 116.7 (C), 115.8 (CH), 115.5 (CH), 22.2 (CH_3_). LC-MS (ESI) *m*/*z* found: 254.3 [M + H]^+^. 

7-Methyl-4-phenylquinazolin-2-amine (**5o**). Yield: 121 mg, 65%; white solid; m.p. 160–162 °C. ^1^H NMR (300 MHz, CDCl_3_, δ ppm, *J* Hz): 7.76–7.69 (m, 3H), 7.47–7.44 (m, 1H), 7.27–7.22 (m, 2H), 7.09 (dd, 1H, *J* = 1.4 Hz, *J* = 8.5 Hz), 5.22 (br s, 2H), 2.53 (s, 3H). ^13^C NMR (300 MHz, CDCl_3_, δ ppm, *J* Hz): 168.9 (C), 165.4 (C), 162.1 (C), 159.6 (C), 153.5 (C), 145.1 (C), 131.6 (CH), 131.5 (CH), 126.9 (CH), 125.5 (CH), 125.1 (CH), 116.7 (C), 115.8 (CH), 115.5 (CH), 22.2 (CH_3_). LC-MS (ESI) *m*/*z* found: 236.3 [M + H]^+^. 

General procedure for synthesis of compounds **5e**–**h**, **5m**, **5p**.

To a round-bottom flask were added 2-chloroquinazoline derivative (1.0 eq.), DIPEA (3.0 eq.), and 4-methoxybenzylamine (2.0 eq.) in dioxane. The mixture was stirred and heated at reflux overnight. The solution was cooled to room temperature and hydrolyzed with water. Aqueous layer was extracted three times with EtOAc. Combined organic layers were dried over MgSO_4_, filtered, and concentrated in vacuo. These intermediates were not isolated, and the next step was carried out without any further purification. Crude product was dissolved in TFA (10 mL), and the mixture was stirred at room temperature for 72 h. A saturated aqueous solution of sodium bicarbonate was added up to alkaline pH. Aqueous layer was extracted three times with EtOAc. Combined organic layers were dried over MgSO_4_, filtered, and concentrated in vacuo. Crude product was purified by flash chromatography (DCM/MeOH (9/1)) to afford the corresponding aminoquinazoline.

6-Methyl-4-phenylquinazolin-2-amine (**5e**). Yield: 27 mg, 41%; yellow solid; m.p. 190–192 °C. ^1^H NMR (300 MHz, DMSO-*d_6_*, δ ppm, *J* Hz): 7.81 (m, 1H), 7.76 (m, 2H), 7.65 (m, 5H), 2.39 (s, 3H). ^13^C NMR (300 MHz, DMSO-*d_6_*, δ ppm, *J* Hz): 174.5 (C), 159.3 (C), 155.8 (C), 138.6 (CH), 136.1 (C), 134.9 (C), 131.5 (CH), 130.1 (2 CH), 129.1 (CH), 127.8 (2 CH), 119.4 (CH), 117.1 (C), 21.2 (CH_3_). LC-MS (ESI) *m*/*z* found: 236.3 [M + H]^+^. 

4-(Furan-2-yl)-6-methoxyquinazolin-2-amine (**5f**). Yield: 45 mg, 56%; yellow solid; m.p. 169–171 °C. ^1^H NMR (300 MHz, DMSO-*d_6_*, δ ppm, *J* Hz): 8.09 (dd, 1H, *J* = 0.8 Hz, *J* = 1.8 Hz), 7.83 (m, 1H), 7.43 (m, 3H), 6.80 (dd, 1H, *J* = 1.8 Hz, *J* = 3.5 Hz), 6.62 (s, 2H), 3.86 (s, 3H). ^13^C NMR (300 MHz, DMSO-*d_6_*, δ ppm, *J* Hz): 159.8 (C), 155.2 (C), 154.8 (C), 152.5 (C), 150.4 (C), 146.6 (CH), 127.6 (CH), 126.4 (CH), 116.0 (C), 115.4 (CH), 112.8 (CH), 104.9 (CH), 55.7 (CH_3_). LC-MS (ESI) *m*/*z* found: 242.2 [M + H]^+^. 

6-Fluoro-4-(furan-2-yl)quinazolin-2-amine (**5g**). Yield: 45 mg, 46%; yellow solid; m.p. 181–183 °C. ^1^H NMR (300 MHz, DMSO-*d_6_*, δ ppm, *J* Hz): 8.23 (dd, 1H, *J* = 2.9 Hz, *J* = 10.4 Hz), 8.11 (m, 1H), 7.69–7.62 (m, 1H), 7.54 (m, 1H), 7.46 (m, 1H), 6.82 (m, 1H), 6.80 (s, 2H, NH_2_). ^13^C NMR (300 MHz, DMSO-*d_6_*, δ ppm, *J* Hz): 160.5 (C), 158.9 (C), 156.1 (C), 155.8 (C), 152.3 (C), 151.7 (CH), 147.1 (CH), 128.5 (CH), 124.1 (C), 115.9 (CH), 112.9 (CH), 110.3 (CH). LC-MS (ESI) *m*/*z* found: 230.2 [M + H]^+^. 

6-Chloro-4-(furan-2-yl)quinazolin-2-amine (**5h**). Yield: 45 mg, 46%; yellow solid; m.p. 184–186 °C. ^1^H NMR (300 MHz, DMSO-*d_6_*, δ ppm, *J* Hz): 8.51 (d, 1H, *J* = 2.3 Hz), 8.15 (dd, 1H, *J* = 0.8 Hz, *J* = 1.8 Hz), 7.73 (dd, 1H, *J* = 2.5 Hz, *J* = 9.2 Hz), 7.47 (m, 2H), 6.97 (s, 2H, NH_2_), 6.82 (dd, 1H, *J* = 1.8 Hz, *J* = 3.5 Hz). ^13^C NMR (300 MHz, DMSO-*d_6_*, δ ppm, *J* Hz): 160.8 (C), 155.8 (C), 153.1 (C), 152.4 (C), 147.4 (CH), 134.5 (C), 128.8 (CH), 126.5 (CH), 125.6 (CH), 116.3 (C), 116.2 (CH), 113.0 (CH). LC-MS (ESI) *m*/*z* found: 246.2, 248.2 [M + H]^+^. 

4-(furan-2-yl)-7-methylquinazolin-2-amine (**5m**). Yield: 21 mg, 13%; yellow solid; m.p. 235–237 °C. ^1^H NMR (300 MHz, DMSO-*d_6_*, δ ppm, *J* Hz): 8.38 (d, 1H, *J* = 8.8 Hz), 8.07 (m, 1H), 7.39 (dd, 1H, *J* = 0.7 Hz, *J* = 3.5 Hz), 7.27 (m, 1H), 7.10 (dd, 1H, *J* = 1.5 Hz, *J* = 8.7 Hz), 6.79 (dd, 1H, *J* = 1.7 Hz, *J* = 3.5 Hz), 6.74 (s, 2H), 2.43 (s, 3H). ^13^C NMR (300 MHz, DMSO-*d_6_*, δ ppm, *J* Hz): 168.44 (C), 165.19 (C), 161.91 (C), 160.50 (C), 151.98 (C), 137.23 (CH), 133.24 (CH), 132.27 (CH), 132.15 (CH), 129.10, 127.84 (CH), 118.9 (CH), 116.07 (CH), 114.25 (C). LC-MS (ESI) *m*/*z* found: 226.3 [M + H]^+^. 

7-Chloro-4-(furan-2-yl)quinazolin-2-amine (**5p**). Yield: 45 mg, 46%; yellow solid; m.p. 181–183 °C. ^1^H NMR (300 MHz, DMSO-*d_6_*, δ ppm, *J* Hz): 8.53 (d, 1H, *J* = 9.1 Hz), 8.11 (dd, 1H, *J* = 0.8 Hz, *J* = 1.7 Hz), 7.48 (d, 1H, *J* = 2.1 Hz), 7.45 (dd, 1H, *J* = 0.8 Hz, *J* = 3.6 Hz), 7.28 (dd, 1H, *J* = 2.2 Hz, *J* = 9.0 Hz), 7.03 (s, 2H, NH_2_), 6.82 (dd, 1H, *J* = 1.8 Hz, *J* = 3.5 Hz). ^13^C NMR (300 MHz, DMSO-*d_6_*, δ ppm, *J* Hz): 161.2 (C), 156.7 (C), 155.2 (C), 152.2 (C), 147.2 (C), 138.9 (CH), 129.0 (CH), 124.5 (CH), 123.2 (CH), 116.3 (C), 114.6 (CH), 113.0 (CH). LC-MS (ESI) *m*/*z* found: 246.2, 248.2 [M + H]^+^. 

General procedure for synthesis of compounds **6a**–**c**. To a suspension of dibromoalkane derivatives (5.1 eq.) in DMF (50 mL) with tetra-n-butylammonium bromide (1%) potassium phthalimide (1.0 eq.) was slowly added. The reaction mixture was stirred at 100 °C overnight, concentrated in vacuo, hydrolyzed with water, and extracted three times with diethyl ether. Combined organic layers were washed with K_2_CO_3_ 0.5 M solution, dried over MgSO_4_, and concentrated in vacuo. Product was precipitated in PE at 0 °C to afford compounds **6a**–**c** after filtration. 

2-(5-Bromopentyl)isoindoline-1,3-dione (**6a**). Yield: 9.0 g, 51%; white solid; m.p. 60–62 °C. ^1^H NMR (300 MHz, CDCl_3_, δ ppm, *J* Hz): 7.89–7.85 (m, 2H), 7.74–7.68 (m, 2H), 3.69 (t, 2H, *J* = 7.0 Hz), 3.38 (t, 2H, *J* = 7.2 Hz), 1.74 (m, 4H), 1.45–1.35 (m, 2H). LC-MS (ESI) *m*/*z* found: 296, 298 [M + H]^+^.

2-(6-Bromohexyl)isoindoline-1,3-dione (**6b**). Yield: 3.9 g, 47%; white solid; m.p. 55–57 °C. ^1^H NMR (300 MHz, CDCl_3_, δ ppm, *J* Hz): 7.87–7.82 (m, 2H), 7.75–7.69 (m, 2H), 3.69 (t, 2H, *J* = 7.2 Hz), 3.40 (t, 2H, *J* = 6.9 Hz), 1.86 (m, 2H), 1.70 (m, 2H), 1.54–1.45 (m, 2H), 1.42–1.32 (m, 2H). LC-MS (ESI) *m*/*z* found: 310, 312 [M + H]^+^.

2-(7-Bromoheptyl)isoindoline-1,3-dione (**6c**). Yield: 708 mg, 58%; white solid; m.p. 52–54 °C. ^1^H NMR (300 MHz, CDCl_3_, δ ppm, *J* Hz): 7.87–7.80 (m, 2H), 7.74–7.67 (m, 2H), 3.67 (t, 2H, *J* = 7.0 Hz), 3.39 (t, 2H, *J* = 6.8 Hz), 1.84 (m, 2H), 1.70–1.65 (m, 2H), 1.44–1.33 (m, 6H). LC-MS (ESI) *m*/*z* found: 324, 326 [M + H]^+^. 

General procedure for synthesis of compounds **7a**–**k**. To a solution of bromoisoindoline-1,3-dione derivatives (1.0 eq.) in acetonitrile (30 mL) was added the corresponding amine (1.2 eq.) and Et_3_N (1.2 eq.). The reaction mixture was stirred at reflux overnight, cooled to room temperature, hydrolyzed with water, and acidified with HCl 1 N solution and extracted with EtOAc. Aqueous layer was alkalized with NaOH 0.5 M solution and then extracted three times with EtOAc. Combined organic layers were dried over MgSO_4_ and concentrated in vacuo. Oil was suspended in PE to obtain a solid, which was filtered to afford the corresponding protected amine.

2-(4-(Piperidin-1-yl)butyl)isoindoline-1,3-dione (**7a**). Yield: 1.6 g, 97%; white solid; m.p. 82–84 °C. ^1^H NMR (300 MHz, CDCl_3_, δ ppm, *J* Hz): 7.82–7.80 (m, 2H), 7.70–7.67 (m, 2H), 3.68 (t, 2H, *J* = 6.9 Hz), 2.32–2.26 (m, 6H), 1.66 (m, 2H), 1.56–1.46 (m, 6H), 1.39 (m, 2H). LC-MS (ESI) *m*/*z* found: 287 [M + H]^+^.

2-(4-(Piperidin-1-yl)pentyl)isoindoline-1,3-dione (**7b**). Yield: 1.3 g, 79%; white solid; m.p. 72–76 °C. ^1^H NMR (300 MHz, CDCl_3_, δ ppm, *J* Hz): 7.85–7.82 (m, 2H), 7.73–7.69 (m, 2H), 3.68 (t, 2H, *J* = 6.9 Hz), 2.38–2.27 (m, 6H), 1.69 (m, 2H), 1.64–1.51 (m, 4H), 1.44–1.31 (m, 6H). LC-MS (ESI) *m*/*z* found: 301 [M + H]^+^.

2-(5-Morpholinopentyl)isoindoline-1,3-dione (**7c**). Yield: 1.7 g, 56%; white solid; m.p. 66–68 °C. ^1^H NMR (300 MHz, CDCl_3_, δ ppm, *J* Hz): 7.86–7.83 (m, 2H), 7.73–7.70 (m, 2H), 3.72–3.67 (m, 6H), 2.44–2.41 (m, 4H), 2.32 (t, 2H, *J* = 7.8 Hz), 1.71 (m, 2H), 1.54 (m, 2H), 1.37 (m, 2H). LC-MS (ESI) *m*/*z* found: 303 [M + H]^+^.

2-(5-(4-Methylpiperazin-1-yl)pentyl)isoindoline-1,3-dione (**7d**). Yield: 800 mg, 38%; yellow oil. ^1^H NMR (300 MHz, CDCl_3_, δ ppm, *J* Hz): 7.86–7.83 (m, 2H), 7.74–7.70 (m, 2H), 3.68 (t, 2H, *J* = 7.2 Hz), 3.01 (t, 2H, *J* = 7.1 Hz), 2.26 (m, 8H), 2.10 (s, 3H), 1.71–1.64 (m, 2H), 1.62–1.51 (m, 2H), 1.43–1.33 (m, 2H). LC-MS (ESI) *m*/*z* found: 317 [M + H]^+^.

Tert-butyl 4-(5-(1,3-dioxoisoindolin-2-yl)pentyl)piperazine-1-carboxylate (**7e**). Yield: 3.0 g, 55%; white solid. ^1^H NMR (300 MHz, CDCl_3_, δ ppm, *J* Hz): 7.89–7.82 (m, 2H), 7.76–7.69 (m, 2H), 3.70 (t, 2H, *J* = 7.2 Hz), 3.45 (m, 4H), 2.39 (m, 6H), 1.76–1.66 (m, 2H), 1.60–1.55 (m, 2H), 1.47 (s, 9H, (CH_3_)_3_), 1.40–1.37 (m, 2H). LC-MS (ESI) *m*/*z* found: 402 [M + H]^+^.

2-(5-(Pyrrolidin-1-yl)pentyl)isoindoline-1,3-dione (**7f**). Yield: 630 mg, 54%; white solid; m.p. 58–60 °C. ^1^H NMR (300 MHz, CDCl_3_, δ ppm, *J* Hz): 7.86–7.83 (m, 2H), 7.72–7.70 (m, 2H), 3.69 (t, 2H, *J* = 7.2 Hz), 2.50–2.46 (m, 4H), 2.45–2.40 (m, 2H), 1.82–1.74 (m, 4H), 1.73–1.66 (m, 2H), 1.62–1.51 (m, 2H), 1.43–1.33 (m, 2H). LC-MS (ESI) *m*/*z* found: 287 [M + H]^+^.

2-(5-(Diethylamino)pentyl)isoindoline-1,3-dione (**7g**). Yield: 670 mg, 60%; yellow oil. ^1^H NMR (300 MHz, CDCl_3_, δ ppm, *J* Hz): 7.86–7.83 (m, 2H), 7.73–7.70 (m, 2H), 3.69 (t, 2H, *J* = 7.2 Hz), 2.51 (q, 4H, *J* = 7.3 Hz), 2.40 (t, 2H, *J* = 7.3 Hz), 1.70 (m, 2H), 1.55–1.45 (m, 2H), 1.39–1.29 (m, 2H), 1.15 (t, 6H, *J* = 7.8 Hz). LC-MS (ESI) *m*/*z* found: 289 [M + H]^+^.

2-(5-(3,4-Dihydroisoquinolin-2(1H)-yl)pentyl)isoindoline-1,3-dione (**7h**). Yield: 1.2 g, 34%; white solid; m.p. 74–76 °C. ^1^H NMR (300 MHz, CDCl_3_, δ ppm, *J* Hz): 7.86–7.83 (m, 2H), 7.73–7.70 (m, 2H), 7.13–7.00 (m, 4H), 3.71 (t, 2H, *J* = 7.3 Hz), 3.61 (s, 2H), 2.89 (t, 2H, *J* = 5.9 Hz), 2.71 (t, 2H, *J* = 6.0 Hz), 2.50 (t, 2H, *J* = 7.7 Hz), 1.79–1.61 (m, 4H), 1.47–1.37 (m, 2H). LC-MS (ESI) *m*/*z* found: 349 [M + H]^+^.

2-(5-(4-Benzylpiperidin-1-yl)pentyl)isoindoline-1,3-dione (**7i**). Yield: 410 mg, 31%; white solid; m.p. 60–62 °C. ^1^H NMR (300 MHz, CDCl_3_, δ ppm, *J* Hz): 7.86–7.84 (m, 2H), 7.73–7.70 (m, 2H), 6.94–6.92 (m, 5H), 3.72 (t, 2H, *J* = 7.3 Hz), 3.07 (m, 3H), 2.63–2.50 (m, 8H), 2.29 (m, 2H), 1.35–1.10 (m, 6H). LC-MS (ESI) *m*/*z* found: 392 [M + H]^+^.

2-(4-(Piperidin-1-yl)hexyl)isoindoline-1,3-dione (**7j**). Yield: 1.3 g, 41%; white solid; m.p. 59–61 °C. ^1^H NMR (300 MHz, CDCl_3_, δ ppm, *J* Hz): 7.87–7.81 (m, 2H), 7.74–7.68 (m, 2H), 3.68 (t, 2H, *J* = 7.0 Hz), 2.10 (m, 2H), 2.35 (m, 4H), 2.29–2.24 (m, 2H), 1.73–1.63 (m, 2H), 1.61–1.54 (m, 4H), 1.51–1.32 (m, 6H). LC-MS (ESI) *m*/*z* found: 316 [M + H]^+^.

2-(4-(Piperidin-1-yl)heptyl)isoindoline-1,3-dione (**7k**). Yield: 687 mg, 57%; white solid; m.p. 56–58 °C. ^1^H NMR (300 MHz, CDCl_3_, δ ppm, *J* Hz): 7.86–7.81 (m, 2H), 7.74–7.69 (m, 2H), 3.67 (t, 2H, *J* = 7.2 Hz), 2.35 (m, 4H), 2.28–2.23 (m, 2H), 1.69–1.19 (m, 16H). LC-MS (ESI) *m*/*z* found: 330 [M + H]^+^.

General procedure for synthesis of compounds **8a**–**k**. A mixture of protected amine derivatives (1.0 eq.) in EtOH (85 mL) with hydrazine hydrate (2.5 eq.) was heated at reflux for 3 h, cooled to room temperature, and then phthalhydrazide was filtered off. Filtrate was concentrated in vacuo, suspended in chloroform, and phthalhydrazide was filtered again. Filtrate was then washed with water, dried over MgSO_4_, and evaporated in vacuo to afford the corresponding amine.

4-(Piperidin-1-yl)butan-1-amine (**8a**). Yield: 1.4 g, 90%; yellow oil. ^1^H NMR (300 MHz, CDCl_3_, δ ppm, *J* Hz): 2.71 (t, 2H, *J* = 6.9 Hz), 2.44 (br m, 2H), 2.28 (t, 2H, *J* = 7.1 Hz), 1.61–1.38 (m, 14H). LC-MS (ESI) *m*/*z* found: 157 [M + H]^+^.

5-(Piperidin-1-yl)pentane-1-amine (**8b**). Yield: 1.4 g, 90%; yellow oil. ^1^H NMR (300 MHz, CDCl_3_, δ ppm, *J* Hz): 2.66 (t, 2H, *J* = 6.9 Hz), 2.34 (m, 4H), 2.25 (t, 2H, *J* = 7.1 Hz), 1.59–1.48 (m, 6H), 1.45–1.39 (m, 6H), 1.33–1.26 (m, 2H). LC-MS (ESI) *m*/*z* found: 171 [M + H]^+^.

5-Morpholinopentan-1-amine (**8c**). Yield: 808 mg, 83%; yellow oil. ^1^H NMR (300 MHz, CDCl_3_, δ ppm, *J* Hz): 3.72 (t, 4H, *J* = 4.5 Hz), 2.72–2.68 (m, 2H), 2.45–2.42 (m, 4H), 2.34 (t, 2H, *J* = 7.8 Hz), 1.56–1.42 (m, 4H), 1.39–1.29 (m, 4H). LC-MS (ESI) *m*/*z* found: 173 [M + H]^+^.

5-(4-Methylpiperazin-1-yl)pentan-1-amine (**8d**). Yield: 390 mg, 83%; yellow oil. ^1^H NMR (300 MHz, CDCl_3_, δ ppm, *J* Hz): 2.60–2.56 (m, 2H), 2.35 (m, 6H), 2.25–2.20 (m, 3H), 2.18 (s, 3H), 1.45–1.31 (m, 5H), 1.27–1.17 (m, 4H). LC-MS (ESI) *m*/*z* found: 186 [M + H]^+^.

Tert-butyl 4-(5-aminopentyl)piperazine-1-carboxylate (**8e**). Yield: 730 mg, 72%; yellow oil. ^1^H NMR (300 MHz, CDCl_3_, δ ppm, *J* Hz): 3.70 (t, 4H, *J* = 5.1 Hz), 2.71 (t, 2H, *J* = 6.6 Hz), 2.39–2.30 (m, 6H), 1.97 (m, 3H), 1.59–1.48 (m, 3H), 1.46 (s, 9H, (CH_3_)_3_), 1.36–1.31 (m, 2H). LC-MS (ESI) *m*/*z* found: 272 [M + H]^+^.

5-(Pyrrolidin-1-yl)pentan-1-amine (**8f**). Yield: 265 mg, 81%; yellow oil. ^1^H NMR (300 MHz, CDCl_3_, δ ppm, *J* Hz): 2.69 (t, 2H, *J* = 6.8 Hz), 2.53–2.50 (m, 4H), 2.46 (t, 2H, *J* = 7.6 Hz), 2.19 (m, 2H), 1.85–1.76 (m, 4H), 1.59–1.29 (m, 6H). LC-MS (ESI) *m*/*z* found: 157 [M + H]^+^.

N^1^,N^1^-Diethylpentane-1,5-diamine (**8g**). Yield: 272 mg, 90%; yellow oil. ^1^H NMR (300 MHz, CDCl_3_, δ ppm, *J* Hz): 3.60 (m, 2H), 2.75 (m, 2H), 2.52 (t, 4H, *J* = 7.3 Hz), 2.40 (t, 2H, *J* = 7.3 Hz), 1.54–1.39 (m, 4H), 1.33–1.25 (m, 2H), 1.05 (t, 6H, *J* = 7.5 Hz). LC-MS (ESI) *m*/*z* found: 159 [M + H]^+^.

5-(3,4-Dihydroisoquinolin-2(1H)-yl)pentan-1-amine (**8h**). Yield: 132 mg, 70%; yellow oil. ^1^H NMR (300 MHz, CDCl_3_, δ ppm, *J* Hz): 7.14–7.06 (m, 4H), 3.61 (m, 2H), 2.88 (t, 2H, *J* = 5.9 Hz), 2.71 (t, 2H *J* = 6.0 Hz), 2.50 (t, 2H, *J* = 7.7 Hz), 2.46 (t, 2H, *J* = 7.1 Hz), 1.79–1.61 (m, 4H), 1.47–1.37 (m, 4H). LC-MS (ESI) *m*/*z* found: 219 [M + H]^+^.

5-(4-Benzylpiperidin-1-yl)pentan-1-amine (**8i**). Yield: 260 mg, 97%; limpid oil. ^1^H NMR (300 MHz, CDCl_3_, δ ppm, *J* Hz): 7.16 (m, 5H), 6.84–6.75 (m, 2H), 3.10 (m, 3H), 2.60–2.50 (m, 8H), 2.29 (m, 2H), 1.39–1.10 (m, 6H), 0.80 (m, 2H). LC-MS (ESI) *m*/*z* found: 261 [M + H]^+^.

6-(Piperidin-1-yl)hexan-1-amine (**8j**). Yield: 476 mg, 65%; yellow oil. ^1^H NMR (300 MHz, CDCl_3_, δ ppm, *J* Hz): 2.62 (t, 2H, *J* = 6.9 Hz), 2.36 (m, 4H), 2.30–2.25 (m, 2H), 1.59 (m, 4H), 1.52–1.42 (m, 6H), 1.36–1.25 (m, 6H). LC-MS (ESI) *m*/*z* found: 185 [M + H]^+^.

7-(Piperidin-1-yl)heptan-1-amine (**8k**). Yield: 300 mg, 78%; yellow oil. ^1^H NMR (300 MHz, CDCl_3_, δ ppm, *J* Hz): 2.64 (t, *J* = 7.2 Hz, 2H), 2.34 (m, 4H), 2.26–2.21 (m, 2H), 1.59–1.17 (m, 18H). LC-MS (ESI) *m*/*z* found: 199 [M + H]^+^.

General procedure for synthesis of compounds **9a**–**x**. A mixture of 2-chloroquinazoline (1.0 eq.), the corresponding amine (3.0 eq.), and DIPEA (3.0 eq.) in dioxane was stirred at reflux overnight. After cooling to room temperature, mixture was hydrolyzed with water and extracted three times with EtOAc. Combined organic layers were dried over MgSO_4_ and concentrated in vacuo. Oil was purified by flash chromatography (DCM/MeOH (10/0 to 9/1)) to afford the corresponding quinazoline. 

N-(1-Benzylpiperidin-4-yl)4-phenylquinazolin-2-amine (**9a**). Yield: 395 mg, 67%; orange oil. ^1^H NMR (300 MHz, CDCl_3_, δ ppm, *J* Hz): 7.82–7.80 (m, 1H), 7.76–7.64 (m, 4H), 7.59–7.53 (m, 3H), 7.41–7.25 (m, 5H), 7.22–7.12 (m, 1H), 5.32 (br s, 1H), 4.22–4.07 (m, 1H), 3.58 (s, 2H), 2.95–2.84 (m, 2H), 2.37–2.24 (m, 2H), 2.23–2.11 (m, 2H), 1.74–1.58 (m, 2H). ^13^C NMR (300 MHz, CDCl_3_, δ ppm, *J* Hz): 170.0 (C), 158.4 (C), 153.5 (C), 138.4 (C), 137.5 (C), 133.7 (CH), 129.6 (CH), 129.5 (2 CH), 129.2 (2 CH), 128.5 (2 CH), 128.2 (2 CH), 127.5 (CH), 127.0 (CH), 126.1 (CH), 122.2 (CH), 118.5 (C), 63.3 (CH), 52.3 (2 CH_2_), 47.8 (CH_2_), 32.4 (2 CH_2_). LC-MS (ESI) *m*/*z* found: 395.3 [M + H]^+^. 

4-Phenyl-N-(pyridin-2-ylmethyl)quinazolin-2-amine (**9b**). Yield: 320 mg, 82%; white solid; m.p. 245 °C. ^1^H NMR (300 MHz, CDCl_3_, δ ppm, *J* Hz): 8.63–8.58 (m, 1H), 7.85 (m, 1H), 7.77–7.63 (m, 5H), 7.59–7.53 (m, 3H), 7.43 (m, 1H), 7.23–7.17 (m, 2H), 6.42 (br s, 1H), 4.96 (d, 2H, J = 5.5 Hz). ^13^C NMR (300 MHz, CDCl_3_, δ ppm, *J* Hz): 165.5 (C), 153.9 (C), 153.4 (C), 148.3 (C), 144.3 (CH), 132.7 (CH), 131.9 (CH), 129.0 (CH), 125.0 (CH), 124.8 (2 CH), 123.7 (2 CH), 122.8 (CH), 121.4 (C), 117.8 (CH), 117.4 (CH), 116.7 (CH), 113.9 (C), 42.1 (CH_2_). LC-MS (ESI) *m*/*z* found: 313.2 [M + H]^+^. 

N-Benzyl-4-phenylquinazolin-2-amine (**9c**). Yield: 210 mg, 54%; yellow solid; m.p. 254 °C. ^1^H NMR (300 MHz, CDCl_3_, δ ppm, *J* Hz): 7.88–7.82 (m, 1H), 7.76–7.68 (m, 4H), 7.60–7.52 (m, 3H), 7.51–7.43 (m, 2H), 7.41–7.26 (m, 3H), 7.24–7.17 (m, 1H), 5.68 (m, 1H), 4.85 (d, 2H, J = 5.8 Hz). ^13^C NMR (300 MHz, CDCl_3_, δ ppm, *J* Hz): 170.1 (C), 158.9 (C), 153.4 (C), 139.4 (C), 137.5 (C), 133.7 (CH), 129.7 (CH), 129.6 (2 CH), 128.6 (2 CH), 128.5 (2 CH), 127.7 (2 CH), 127.5 (CH), 127.2 (CH), 126.2 (CH), 122.5 (CH), 118.7 (C), 45.7 (CH_2_). LC-MS (ESI) *m*/*z* found: 312.2 [M + H]^+^. 

4-(((4-Phenylquinazolin-2-yl)amino)methyl)benzonitrile (**9d**). Yield: 302 mg, 72%; white solid; m.p. 273 °C. ^1^H NMR (300 MHz, CDCl_3_, δ ppm, *J* Hz): 7.86–7.83 (m, 1H), 7.75–7.60 (m, 6H), 7.59–7.51 (m, 5H), 7.23 (ddd, 1H, *J* = 1.9 Hz, *J* = 6.2 Hz, *J* = 8.2 Hz), 5.87 (br s, 1H), 4.88 (d, 2H, *J* = 6.2 Hz). ^13^C NMR (300 MHz, CDCl_3_, δ ppm, *J* Hz): 165.9 (C), 153.8 (C), 140.7 (C), 132.4 (C), 129.3 (C), 127.6 (2 CH), 125.5 (CH), 125.4 (CH), 124.9 (2 CH), 123.9 (2 CH), 123.4 (2 CH), 122.9 (CH), 121.3 (C), 118.3 (CH), 114.3 (CH), 114.0 (C), 106.1 (C), 40.2 (CH_2_). LC-MS (ESI) *m*/*z* found: 337.2 [M + H]^+^. 

N-(4-Methoxybenzyl)-4-phenylquinazolin-2-amine (**9e**). Yield: 323 mg, 76%; yellow solid; m.p. 246 °C. ^1^H NMR (300 MHz, DMSO-*d_6_*, δ ppm, *J* Hz): 7.92 (br s, 1H), 7.74–7.63 (m, 4H), 7.62–7.49 (m, 4H), 7.38–7.29 (m, 2H), 7.18 (ddd, 1H, *J* = 1.2 Hz, *J* = 6.9 Hz, *J* = 8.2 Hz), 6.90–6.83 (m, 2H), 4.56 (d, 2H, *J* = 6.3 Hz), 3.70 (s, 3H). ^13^C NMR (300 MHz, CDCl_3_, δ ppm, *J* Hz): 169.7 (C), 159.4 (C), 158.5 (CH), 153.4 (C), 137.5 (C), 134.3 (CH), 132.7 (C), 130.2 (2 CH), 129.9 (2 CH), 129.2 (2 CH), 128.9 (2 CH), 127.6 (CH), 126.2 (C), 122.6 (CH), 118.1 (C), 114.1 (CH), 55.6 (CH_3_), 44.1 (CH_2_). LC-MS (ESI) *m*/*z* found: 342.2 [M + H]^+^.

4-(2-((4-Phenylquinazolin-2-yl)amino)ethyl)phenol (**9f**). Yield: 323 mg, 76%; white solid; m.p. 263 °C. ^1^H NMR (300 MHz, CDCl_3_, δ ppm, *J* Hz): 7.86–7.80 (m, 1H), 7.76–7.64 (m, 4H), 7.59–7.53 (m, 3H), 7.19 (ddd, 1H, *J* = 1.8 Hz, *J* = 6.1 Hz, *J* = 8.1 Hz), 7.10–7.05 (m, 2H), 6.76–6.70 (m, 2H), 5.45 (br s, 1H), 3.84 (q, 2H, *J* = 6.6 Hz), 2.93 (t, 2H, *J* = 6.6 Hz). ^13^C NMR (300 MHz, CDCl_3_, δ ppm, *J* Hz): 170.4 (C), 158.7 (C), 154.8 (C), 153.0 (C), 137.2 (C), 134.1 (CH), 130.5 (CH), 129.89 (2 CH), 129.8 (CH), 129.6 (2 CH), 128.5 (2 CH), 127.6 (CH), 125.6 (C), 122.5 (CH), 118.4 (C), 115.5 (2 CH), 43.2 (CH_2_), 34.9 (CH_2_). LC-MS (ESI) *m*/*z* found: 342.2 [M + H]^+^. 

4-Phenyl-N-(2-(piperidin-1-yl)ethyl)quinazolin-2-amine (**9g**). Yield: 80 mg, 21%; yellow oil. ^1^H NMR (300 MHz, CDCl_3_, δ ppm, *J* Hz): 7.79–7.77 (m, 1H), 7.72–7.69 (m, 2H), 7.67–7.65 (m, 2H), 7.56–7.53 (m, 3H), 7.17–7.12 (m, 1H), 5.93 (br m, 1H), 3.67 (m, 2H, *J* = 5.7 Hz), 2.60 (t, 2H, *J* = 6.12 Hz), 2.45 (m, 4H), 1.63–1.56 (m, 4H), 1.48–1.45 (m, 2H). ^13^C NMR (300 MHz, CDCl_3_, δ ppm, *J* Hz): 170.0 (C), 159.0 (C), 153.5 (C), 137.6 (C), 133.6 (CH), 129.6 (CH), 129.5 (2 CH), 128.5 (2 CH), 127.5 (CH), 126.0 (CH), 122.1 (CH), 118.4 (C), 57.6 (CH_2_), 54.4 (2 CH_2_), 38.4 (CH_2_), 25.9 (2 CH_2_), 24.5 (CH_2_). LC-MS (ESI) *m*/*z* found: 333.0 [M + H]^+^. 

4-Phenyl-N-(3-(piperidin-1-yl)propyl)quinazolin-2-amine hydrochloride (**9h**). Yield: 60 mg, 21%; beige solid; m.p. 202 °C. ^1^H NMR (300 MHz, DMSO-*d_6_*, δ ppm, *J* Hz): 10.26 (br s), 7.7–7.68 (m, 4H), 7.59–7.54 (m, 5H), 7.20 (br t, 1H, *J* = 8.0 Hz), 3.51–3.33 (m, 4H), 3.1 (m, 2H), 2.84 (m, 2H), 2.06 (m, 2H), 1.77–1.66 (m, 5H), 1.37 (m, 1H). ^13^C NMR (300 MHz, DMSO-*d_6_*, δ ppm): 169.7 (C), 159.3 (C), 153.3 (C), 137.4 (C), 134.3 (CH), 130.2 (CH), 129.8 (2 CH), 128.9 (2 CH), 127.5 (CH), 126.1 (CH), 122.7 (CH), 118.0 (C), 54.5 (CH_2_), 52.4 (2 CH_2_), 38.7 (CH_2_), 23.7 (CH_2_), 22.8 (2 CH_2_), 21.9 (CH_2_). LC-MS (ESI) *m*/*z* found: 347.0 [M + H]^+^. 

4-Phenyl-N-(4-(piperidin-1-yl)butyl)quinazolin-2-amine (**9i**). Yield: 163 mg, 54%; yellow oil. ^1^H NMR (CDCl_3_, δ ppm, *J* Hz): 7.82 (m, 1H), 7.70–7.67 (m, 3H) 7.56 (m, 3H), 7.19 (m, 2H), 5.55 (br m, 1H), 3.66 (m, 2H), 3.01 (m, 2H), 2.02–1.26 (m, 14H). ^13^C NMR (300 MHz, CDCl_3_, δ ppm): 170.4 (C), 158.9 (C), 153.4 (C), 137.4 (C), 133.9 (CH), 129.8(CH), 129.6 (2 CH), 128.5 (2 CH), 127.6 (CH), 125.9 (CH), 122.5 (CH), 118.5 (C), 57.2 (CH_2_), 53.1 (2 CH_2_), 40.3 (CH_2_), 27.3 (CH_2_), 22.7 (2 CH_2_), 22.3 (2 CH_2_), 20.9 (CH_2_). LC-MS (ESI) *m*/*z* found: 361.3 [M + H]^+^. 

4-Phenyl-N-(5-(piperidin-1-yl)pentyl)quinazolin-2-amine (**9j**). Yield: 170 mg, 56%; yellow oil. ^1^H NMR (300 MHz, CDCl_3_, δ ppm, *J* Hz): 7.78 (m, 1H), 7.77–7.68 (m, 2H) 7.67–7.65 (m, 2H), 7.55–7.53 (m, 3H), 7.18–7.12 (m, 1H), 5.55 (br t, 1H, *J* = 5.6 Hz), 3.60 (m, 2H), 2.39 (m, 3H), 2.32 (t, 2H, *J* = 7.4 Hz), 1.73–1.66 (m, 2H), 1.64–1.54 (m, 5H), 1.49–1.26 (m, 6H). ^13^C NMR (300 MHz, CDCl_3_, δ ppm): 169.9 (C), 159.1 (C), 153.4 (C), 137.5 (C), 133.7 (CH), 129.6 (CH), 129.5 (2 CH), 128.4 (2 CH), 127.5 (CH), 126.1 (CH), 122.2 (CH), 118.4 (C), 59.3 (CH_2_), 54.6 (2 CH_2_), 41.4 (CH_2_), 29.6 (CH_2_), 26.5 (CH_2_), 25.8 (2 CH_2_), 25.1 (2 CH_2_), 24.4 (CH_2_). LC-MS (ESI) *m*/*z* found: 375.0 [M + H]^+^. 

N-(5-Morpholinopentyl)-4-phenylquinazolin-2-amine (**9k**). Yield: 163 mg, 54%; yellow oil. ^1^H NMR (300 MHz, CDCl_3_, δ ppm, *J* Hz): 7.81–7.78 (m, 1H), 7.73–7.65 (m, 4H), 7.57–7.52 (m, 3H), 7.16 (m, 1H), 5.36–5.32 (m, 1H), 3.72 (t, 4H, *J* = 4.6 Hz), 3.60 (m, 2H), 2.45–2.42 (m, 4H), 2.38–2.33 (m, 2H), 1.71 (m, 2H), 1.63–1.42 (m, 4H). ^13^C NMR (300 MHz, CDCl_3_, δ ppm): 170.0 (C), 159.1 (C), 153.4 (C), 137.5 (C), 133.7 (CH), 129.6 (C), 129.5 (2 CH), 128.5 (2 CH), 127.5 (C), 126.1 (C), 122.3 (C), 118.4 (C), 66.9 (2 CH_2_), 59.0 (CH_2_), 53.7 (2 CH_2_), 41.4 (CH_2_), 29.5 (CH_2_), 26.2 (CH_2_), 24.8 (CH_2_). LC-MS (ESI) *m*/*z* found: 377.2 [M + H]^+^. 

N-(5-(4-Methylpiperazin-1-yl)pentyl)-4-phenylquinazolin-2-amine (**9l**). Yield: 386 mg, 55%; yellow oil. ^1^H NMR (300 MHz, CDCl_3_, δ ppm, *J* Hz): 7.80–7.77 (m, 1H), 7.71–7.65 (m, 4H), 7.54–7.51 (m, 3H), 7.17–7.12 (m, 1H), 5.35 (m, 1H), 3.58 (m, 2H), 2.46 (m, 7H), 2.38–2.33 (m, 3H), 2.28 (s, 3H), 1.75–1.65 (m, 2H), 1.62–1.52 (m, 2H), 1.50–1.40 (m, 2H). ^13^C NMR (300 MHz, CDCl_3_, δ ppm): 169.93 (C), 159.1 (C), 153.4 (C), 137.5 (C), 133.7 (CH), 129.6 (CH), 129.5 (2 CH), 128.5 (2 CH), 127.5 (CH), 126.1 (CH), 122.2 (CH), 118.4 (C), 58.6 (CH_2_), 55.1 (2 CH_2_), 53.2 (2 CH_2_), 46.0 (CH_3_), 41.4 (CH_2_), 29.5 (CH_2_), 26.6 (CH_2_), 24.9 (CH_2_). LC-MS (ESI) *m*/*z* found: 390.3 [M + H]^+^. 

4-Phenyl-N-[5-(piperazin-1-yl)pentyl]quinazolin-2-amine (**9m**). Yield: 155 mg, 56%; orange oil. ^1^H NMR (300 MHz, CDCl_3_, δ ppm, *J* Hz): 7.78 (d, 1H, *J* = 8.5 Hz), 7.70–7.64 (m, 4H), 7.52 (t, 3H, *J* = 3.2 Hz), 7.14 (quintuplet, 1H, *J* = 4.1 Hz), 5.32 (t, 1H, *J* = 5.4 Hz), 3.58 (quadruplet, 2H, *J* = 6.6 Hz), 2.90 (t, 4H, *J* = 4.9 Hz), 2.45–2.33 (m + t, 7H, *J* = 7.5 Hz), 1.70 (quintuplet, 2H, *J* = 7.2 Hz), 1.59–1.42 (m, 4H). ^13^C NMR (300 MHz, CDCl_3_, δ ppm): 170.1 (C), 159.2 (C), 153.5 (C), 153.5 (C), 137.6 (C), 133.8 (CH), 129.7 (CH), 129.6 (2 CH), 128.6 (2 CH), 127.6 (CH), 126.2 (CH), 122.3 (CH), 118.6 (C), 59.3 (CH_2_), 54.5 (2 CH_2_), 46.1 (2 CH_2_), 41.5 (CH_2_), 29.7 (CH_2_), 26.5 (CH_2_), 25.1 (CH_2_).LC-MS (ESI) *m*/*z* found: 376.4 [M + H]^+^. 

Tert-butyl 4-{5-[(4-phenylquinazolin-2-yl)amino]pentyl}piperazine-1-carboxylate (**9n**). Yield: 81 mg, 41%; orange oil. ^1^H NMR (300 MHz, CDCl_3_, δ ppm, *J* Hz): 7.79 (d, 1H, *J* = 8.3 Hz), 7.70–7.65 (m, 4H), 7.54–7.52 (m + t, 3H, *J* = 3.2 Hz), 7.15 (quintuplet, 1H, *J* = 4.1 Hz), 5.30 (m, 1H), 3.59 (quadruplet, 2H, *J* = 6.6 Hz), 3.42 (t, 4H, *J* = 5.0 Hz), 2.38–2.32 (m, 6H), 1.75–1.68 (m, 2H), 1.59–1.45 (m, 13H). ^13^C NMR (300 MHz, CDCl_3_, δ ppm): 170.1 (C), 159.2 (C), 154.9 (C), 153.5 (C), 137.6 (C), 133.8 (CH), 129.8 (CH), 129.6 (2 CH), 128.6 (2 CH), 127.6 (CH), 126.2 (CH), 122.3 (CH), 118.6 (C), 79.7 (C), 58.7 (CH_2_), 53.2 (2 CH_2_), 41.5 (3 CH_2_), 29.7 (CH_2_), 28.6 ((CH_3_)_3_), 26.7 (CH_2_), 25.0 (CH_2_).LC-MS (ESI) *m*/*z* found: 476.4 [M + H]^+^. 

4-Phenyl-N-(5-(pyrrolidin-1-yl)pentyl)quinazolin-2-amine (**9o**). Yield: 107 mg, 49%; yellow oil. ^1^H NMR (300 MHz, CDCl_3_, δ ppm, *J* Hz): 7.80–7.78 (m, 1H), 7.71–7.65 (m, 4H), 7.55–7.52 (m, 3H), 7.17–7.12 (m, 1H), 5.36 (m, 1H), 3.59 (m, 2H), 2.55–2.53 (m, 4H), 2.52–2.46 (m, 2H), 1.82–1.78 (m, 4H), 1.73–1.69 (m, 2H), 1.66–1.58 (m, 2H), 1.52–1.45 (m, 2H). ^13^C NMR (300 MHz, CDCl_3_, δ ppm): 169.9 (C), 159.0 (C), 153.4 (C), 137.2 (C), 134.1 (CH), 129.9 (CH), 129.6 (2 CH), 128.5 (2 CH), 127.7 (CH), 126.1 (CH), 122.6 (CH), 119.2 (CH) 55.5 (CH_2_), 53.5 (2 CH_2_), 40.9 (CH_2_), 28.9 (CH_2_), 25.3 (CH_2_), 24.1 (CH_2_), 23.4 (2 CH_2_). LC-MS (ESI) *m*/*z* found: 361.2 [M + H]^+^. 

N^1^,N^1^-Diethyl-N^5^-(4-phenylquinazolin-2-yl)pentane-1,5-diamine (**9p**). Yield: 103 mg, 45%; yellow oil. ^1^H NMR (300 MHz, CDCl_3_, δ ppm, *J* Hz): 7.81–7.78 (m, 1H), 7.71–7.65 (m, 4H), 7.56–7.52 (m, 3H), 7.18–7.12 (m, 1H), 5.35 (m, 1H), 3.59 (m, 2H), 2.55 (q, 4H, *J* = 7.34 Hz), 2.48–2.43 (m, 2H), 1.71 (m, 2H), 1.59–1.40 (m, 4H), 1.03 (t, 6H, *J* = 7.40 Hz). ^13^C NMR (300 MHz, CDCl_3_, δ ppm): 169.9 (C), 159.1 (C), 153.5 (C), 137.5 (C), 133.7 (CH), 129.6 (CH), 129.5 (2 CH), 128.4 (2 CH), 127.5 (CH), 126.1 (CH), 122.2 (CH), 118.4 (C), 52.7 (CH_2_), 46.8 (2 CH_3_), 41.5 (CH_2_), 29.6 (CH_2_), 26.6 (CH_2_), 25.1 (CH_2_), 11.5 (2 CH_2_). LC-MS (ESI) *m*/*z* found: 363.2 [M + H]^+^. 

N-(5-(3,4-Dihydroisoquinolin-2(1H)-yl)pentyl)-4-phenylquinazolin-2-amine (**9q**). Yield: 44 mg, 36%; yellow oil. ^1^H NMR (300 MHz, CDCl_3_, δ ppm, *J* Hz): 7.82–7.79 (m, 1H), 7.72–7.66 (m, 4H), 7.56–7.53 (m, 3H), 7.19–7.09 (m, 4H), 7.03–7.00 (m, 1H), 5.35–5.32 (m, 1H), 3.65–3.59 (m, 4H), 2.91 (t, 2H, *J* = 5.6 Hz), 2.73 (t, 2H, *J* = 5.7 Hz), 2.56–2.52 (m, 2H), 1.78–1.65 (m, 4H), 1.57–1.27 (m, 2H). ^13^C NMR (300 MHz, CDCl_3_, δ ppm): 170.03 (C), 158.9 (C), 153.4 (C), 137.4 (C), 133.8 (C), 130.0 (C), 129.6 (CH), 129.6 (2 CH), 128.6 (CH), 128.5 (2 CH), 128.3 (CH), 128.2 (CH), 128.1 (CH), 127.9 (CH), 127.8 (CH), 127.7 (CH), 122.7 (CH), 118.2 (C), 56.8 (CH_2_), 54.4 (CH_2_), 50.0 (CH_2_), 41.2 (CH_2_), 29.4 (CH_2_), 26.9 (CH_2_), 25.6 (CH_2_), 24.6 (CH_2_). LC-MS (ESI) *m*/*z* found: 423.4 [M + H]^+^. 

N-(5-(4-Benzylpiperidin-1-yl)pentyl)-4-phenylquinazolin-2-amine (**9r**). Yield: 80 mg, 50%; yellow oil. ^1^H NMR (300 MHz, CDCl_3_, δ ppm, *J* Hz): 7.81–7.78 (m, 1H), 7.72–7.65 (m, 4H), 7.55–7.53 (m, 3H), 7.30–7.12 (m, 6H), 5.38 (m, 1H), 3.58 (m, 2H), 2.94–2.90 (m, 2H), 2.53 (d, 2H, *J* = 6.9 Hz), 2.35–2.30 (m, 2H), 1.90–1.81 (m, 2H), 1.72–1.65 (m, 2H), 1.61–1.51 (m, 4H), 1.49–1.39 (m, 3H), 1.36–1.27 (m, 2H). ^13^C NMR (300 MHz, CDCl_3_, δ ppm): 169.9 (C), 159.1 (C), 153.5 (C), 140.7 (C), 137.5 (C), 133.7 (CH), 129.6 (CH), 129.5 (2 CH), 129.1 (2 CH), 128.5 (2 CH), 128.1 (2 CH), 127.5 (CH), 126.1 (CH), 125.8 (CH), 122.2 (CH), 118.4 (C), 59.0 (CH_2_), 54.0 (2 CH_2_), 43.2 (CH_2_), 41.4 (2 CH_2_), 38.0 (CH_2_), 32.1 (CH_2_), 29.6 (CH_2_), 26.7 (CH), 25.1 (CH_2_). LC-MS (ESI) *m*/*z* found: 465.3 [M + H]^+^. 

N^1^-(4-phenylquinazolin-2-yl)pentane-1,5-diamine (**9s**). Yield: 50 mg, 39%; yellow oil. ^1^H NMR (300 MHz, CDCl_3_, δ ppm, *J* Hz): 7.79–7.76 (m, 1H), 7.71–7.65 (m, 4H), 7.56–7.53 (m, 3H), 7.18–7.12 (m, 1H), 5.53 (m, 1H), 3.60 (m, 2H), 2.69–2.65 (m, 2H), 2.46 (m, 2H), 1.74–1.70 (m, 2H), 1.54–1.53 (m, 4H). ^13^C NMR (300 MHz, CDCl_3_, δ ppm): 170.0 (C), 159.0 (C), 153.4 (C), 137.5 (C), 133.8 (CH), 129.7 (CH), 129.5 (2 CH), 128.5 (2 CH), 127.5 (CH), 126.0 (CH), 122.2 (CH), 118.4 (C), 41.7 (CH_2_), 41.1 (CH_2_), 32.5 (CH_2_), 29.2 (CH_2_), 24.0 (CH_2_). LC-MS (ESI) *m*/*z* found: 307.2 [M + H]^+^. 

4-Phenyl-N-(6-(piperidin-1-yl)hexyl)quinazolin-2-amine (**9t**). Yield: 163 mg, 54%; yellow oil. ^1^H NMR (300 MHz, CDCl_3_, δ ppm, *J* Hz): 7.80–7.77 (m, 1H), 7.71–7.64 (m, 4H), 7.54–7.52 (m, 3H), 7.17–7.11 (m, 1H), 5.37 (m, 1H), 3.57 (m, 2H), 2.37 (m, 4H), 2.31–2.26 (m, 2H), 1.72–1.63 (m, 2H), 1.63–1.55 (m, 4H), 1.52–1.32 (m, 8H). ^13^C NMR (300 MHz, CDCl_3_, δ ppm): 169.9 (C), 159.1 (C), 153.5 (C), 137.5 (C), 133.6 (CH), 129.6 (CH), 129.5 (2 CH), 128.5 (2 CH), 127.5 (CH), 126.1 (CH), 122.1 (CH), 118.4 (C), 59.5 (CH_2_), 54.6 (2 CH_2_), 41.5 (CH_2_), 29.6 (CH_2_), 27.5 (CH_2_), 26.9 (CH_2_), 26.8 (CH_2_), 25.9 (2 CH_2_), 24.5 (CH_2_). LC-MS (ESI) *m*/*z* found: 390.3 [M + H]^+^. 

4-Phenyl-N-(7-(piperidin-1-yl)heptyl)quinazolin-2-amine (**9u**). Yield: 150 mg, 75%; yellow oil. ^1^H NMR (300 MHz, CDCl_3_, δ ppm, *J* Hz): 7.81–7.78 (m, 1H), 7.72–7.65 (m, 4H), 7.56–7.52 (m, 3H), 7.18–7.12 (m, 1H), 5.31 (m, 1H), 3.58 (m, 2H), 2.39 (m, 4H), 2.33–2.28 (m, 2H), 1.70–1.65 (m, 2H), 1.63–1.57 (m, 4H), 1.55–1.49 (m, 2H), 1.47–1.39 (m, 4H), 1.36–1.26 (m, 4H). ^13^C NMR (300 MHz, CDCl_3_, δ ppm): 169.9 (C), 159.0 (C), 153.4 (C), 137.5 (C), 133.7 (CH), 129.6 (CH), 129.5 (2 CH), 128.5 (2 CH), 127.5 (CH), 126.1 (CH), 122.2 (CH), 118.4 (C), 59.4 (CH_2_), 54.5 (2 CH_2_), 41.5 (CH_2_), 29.6 (CH_2_), 29.3 (CH_2_), 27.6 (CH_2_), 26.9 (CH_2_), 26.5 (CH_2_), 25.6 (2 CH_2_), 24.2 (CH_2_). LC-MS (ESI) *m*/*z* found: 403.3 [M + H]^+^. 

6-Bromo-4-(furan-2-yl)-N-[5-(piperidin-1-yl)pentyl]quinazolin-2-amine (**9v**). Yield: 85 mg, 39%; yellow oil. ^1^H NMR (300 MHz, CDCl_3_, δ ppm, *J* Hz): 8.77 (d, 1H, *J* = 2.2 Hz), 7.79 (dd, 1H, *J* = 0.8 Hz, *J* = 1.7 Hz), 7.72 (dd, 1H, *J* = 2.2 Hz, *J* = 9.0 Hz), 7.48 (d, 1H, *J* = 9.0 Hz), 7.44 (d, 1H, *J* = 3.3 Hz), 6.67 (dd, 1H, *J* = 1.7 Hz, *J* = 3.4 Hz), 5.37 (t, 1H, *J* = 5.6 Hz, NH), 5.37 (t, 2H, *J* = 6.2 Hz), 2.90–2.84 (m, 3H), 1.99–1.94 (m, 5H), 1.77–1.70 (m, 4H), 1.55–1.44 (m, 4H), 1.27–1.22 (m, 2H). ^13^C NMR (300 MHz, CDCl_3_, δ ppm, *J* Hz): 159.1 (C), 155.8 (C), 152.8 (C), 145.8 (C), 136.9 (C), 129.1 (CH), 128.0 (CH), 117.7 (CH), 115.8 (C), 115.5 (2 CH), 112.3 (CH), 57.5 (2 CH_2_), 53.3 (CH_2_), 41.0 (CH_2_), 29.1 (CH_2_), 24.3 (CH_2_), 23.5 (CH_2_), 22.8 (CH_2_), 22.4 (2 CH_2_). LC-MS (ESI) *m*/*z* found: 443.3, 445.2 [M + H]^+^. 

4-(furan-2-yl)-6-methyl-N-[5-(piperidin-1-yl)pentyl]quinazolin-2-amine (**9w**). Yield: 20 mg, 14%; yellow oil. ^1^H NMR (300 MHz, (CD_3_)_2_CO, δ ppm, *J* Hz): 8.42 (m, 1H), 7.96 (dd, 1H, *J* = 0.8 Hz, *J* = 1.8 Hz), 7.53 (m, 1H), 7.47 (m, 1H), 7.41 (d, 1H, *J* = 3.2 Hz), 6.74 (dd, 1H, *J* = 1.9 Hz, *J* = 3.5 Hz), 6.33 (t, 1H, *J* = 5.4 Hz), 3.55 (q, 2H, *J* = 6.9 Hz), 2.46 (s, 3H), 2.28 (m, 6H), 1.72 (m, 2H), 1.55–1.39 (m, 11H). ^13^C NMR (300 MHz, (CD_3_)_2_CO, δ ppm, *J* Hz): 159.2 (2 C), 155.6 (C), 153.0 (C), 145.6 (CH), 135.3 (C), 131.6 (CH), 126.1 (CH), 125.3 (C), 116.2 (CH), 114.6 (CH), 111.9 (CH), 59.0 (CH_2_), 54.5 (CH_2_), 41.1 (CH_2_), 29.3 (CH_2_), 26.6 (CH_2_), 26.0 (CH_2_), 24.7 (2 CH_2_), 24.5 (2 CH_2_), 20.6 (CH_3_). LC-MS (ESI) *m*/*z* found: 379.3 [M + H]^+^. 

4-(furan-2-yl)-7-methyl-N-[5-(piperidin-1-yl)pentyl]quinazolin-2-amine (**9x**). Yield: 20 mg, 14%; yellow oil. ^1^H NMR (300 MHz, CDCl_3_, δ ppm, *J* Hz): 8.43 (d, 1H, *J* = 8.6 Hz), 7.73 (d, 1H, *J* = 1.5 Hz), 7.42 (m, 1H), 7.34 (d, 1H, *J* = 3.4 Hz), 7.07 (dd, 1H, *J* = 1.7 Hz, *J* = 8.7 Hz), 6.36 (dd, 1H, *J* = 1.8 Hz, *J* = 3.4 Hz), 5.26 (t, 1H, *J* = 5.8 Hz), 3.55 (q, 2H, *J* = 6.8 Hz), 2.40 (m, 9H), 1.65 (m, 9H), 1.44 (m, 5H). ^13^C NMR (300 MHz, CDCl_3_, δ ppm, *J* Hz): 159.2 (C), 156.4 (2 C), 154.2(C), 145.2 (C), 144.5 (CH), 126.5 (CH), 125.3 (CH), 124.9 (CH), 115.2 (C), 114.9 (CH), 112.1 (CH), 58.0 (CH_2_), 53.6 (CH_2_), 41.1 (CH_2_), 29.3 (CH_2_), 24.5 (CH_2_), 24.3 (CH_2_), 23.6 (2 CH_2_), 22.9 (2 CH_2_), 22.0 (CH_3_). LC-MS (ESI) *m*/*z* found: 379.4 [M + H]^+^. 

General procedure for synthesis of compounds **10a**–**d**. The formation of quinazoline derivatives was carried out according to published procedures [32]. In a tube were added 2-chloroquinazoline (1.0 eq.), BINAP (0.1 eq.), the corresponding amine (1.0 eq.), Cs_2_CO_3_ (3.0 eq.), and palladium diacetate (5%) in 4 mL of anhydrous dioxane. The mixture reaction was degassed for 5 min with a nitrogen flow, and the tube was sealed. The reaction was heated overnight at 130 °C. The mixture was then hydrolyzed with water and extracted three times with EtOAc. Combined organic layers were dried over MgSO_4_ and concentrated in vacuo. Crude was purified using flash chromatography (DCM/MeOH (10/0 to 9/1)) to afford the corresponding quinazoline. 

4-Phenyl-N-(4-(pyridine-2-yl)quinazolin-2-amine (**10a**). Yield: 249 mg, 67%; brown solid; m.p. 287 °C. ^1^H NMR (300 MHz, DMSO-*d_6_*, δ ppm, *J* Hz): 10.00 (br s, 1H), 8.68–8.63 (m, 1H), 8.34 (ddd, 1H, *J* = 0.8 Hz, *J* = 1.9 Hz, *J* = 4.8 Hz), 7.92–7.76 (m, 6H), 7.68–7.61 (m, 3H), 7.47–7.40 (m, 1H), 7.04 (ddd, 1H, *J* = 0.9 Hz, *J* = 4.9 Hz, *J* = 6.5 Hz). ^13^C NMR (300 MHz, CDCl_3_, δ ppm): 170.3 (C), 155.0 (C), 152.6 (C), 152.1 (C), 146.4 (CH), 138.9 (CH), 136.9 (C), 134.1 (2 CH), 130.1 (CH), 129.9 (2 CH), 128.6 (CH), 127.5 (CH), 127.0 (CH), 124.5 (CH), 119.5 (C), 117.5 (CH), 113.3 (CH). LC-MS (ESI) *m*/*z* found: 299.2 [M + H]^+^. 

4-Phenyl-N-(4-(piperidin-1-ylmethyl)phenyl)quinazolin-2-amine (**10b**). Yield: 150 mg, 71%; yellow oil. ^1^H NMR (300 MHz, DMSO-*d_6_*, δ ppm, *J* Hz): 7.91–7.89 (m, 1H), 7.85–7.72 (m, 6H), 7.59–7.57 (m, 3H), 7.43 (m, 1H), 7.36–7.26 (m, 3H), 3.53 (s, 2H), 2.45 (m, 4H), 1.64–1.59 (m, 4H), 1.47–1.45 (m, 2H). ^13^C NMR (300 MHz, CDCl_3_, δ ppm): 170.1 (C), 156.1 (C), 152.8 (C), 138.9 (C), 137.2 (C), 133.9 (CH), 130.1 (CH), 129.9 (CH), 129.8 (CH), 129.6 (2 CH), 128.6 (2 CH), 127.4 (CH), 126.9 (CH), 123.6 (CH), 122.6 (C), 119.1 (C), 118.49 (2 CH), 63.2 (CH_2_), 54.2 (2 CH_2_), 25.7 (2 CH_2_), 24.3 (CH_2_). LC-MS (ESI) *m*/*z* found: 395.2 [M + H]^+^. 

4-(furan-2-yl)-6-methyl-N-{4-[(piperidin-1-yl)methyl]phenyl}quinazolin-2-amine (**10c**). Yield: 25 mg, 19%; yellow oil. ^1^H NMR (300 MHz, (CD_3_)_2_CO, δ ppm, *J* Hz): 8.71 (s, 1H, NH), 8.52 (m, 1H), 8.03 (m, 3H), 7.66 (m, 2H), 7.50 (dd, 1H, *J* = 0.8 Hz, *J* = 3.5 Hz), 7.30 (m, 2H), 6.79 (dd, 1H, *J* = 1.8 Hz, *J* = 3.5 Hz), 3.43 (s, 2H), 2.51 (s, 3H), 2.38 (m, 4H), 1.56 (m, 4H), 1.43 (m, 2H). ^13^C NMR (300 MHz, (CD_3_)_2_CO, δ ppm, *J* Hz): 156.4 (C), 155.6 (C), 153.2 (C), 152.2 (C), 146.0 (C), 139.8 (CH), 135.8 (C), 133.4 (2 CH), 132.1 (C), 129.1 (CH), 126.6 (CH), 125.4 (CH), 118.3 (2 CH), 116.9 (C), 115.4 (CH), 112.2 (CH), 63.1 (CH_2_), 54.2 (2 CH_2_), 26.0 (2 CH_2_), 24.3 (CH_2_), 20.7 (CH_3_). LC-MS (ESI) *m*/*z* found: 399.4 [M + H]^+^. 

4-(furan-2-yl)-7-methyl-N-{4-[(piperidin-1-yl)methyl]phenyl}quinazolin-2-amine (**10d**). Yield: 30 mg, 12%; yellow oil. ^1^H NMR (300 MHz, CD_3_OD, δ ppm, *J* Hz): 8.56 (d, 1H, *J* = 8.8 Hz), 8.44 (s, 1H, NH), 8.00 (m, 2H), 7.90 (d, 1H, *J* = 1.2 Hz), 7.51 (d, 1H, *J* = 3.6 Hz), 7.45 (m, 3H), 7.20 (dd, 1H, *J* = 1.5 Hz, *J* = 8.8 Hz), 6.73 (dd, 1H, *J* = 1.8 Hz, *J* = 3.6 Hz), 4.24 (s, 2H), 3.20 (m, 3H), 2.48 (s, 3H), 1.80 (m, 6H). ^13^C NMR (300 MHz, CD_3_OD, δ ppm, *J* Hz): 156.3 (C), 155.9 (C), 153.3 (C), 153.1 (C), 145.9 (C), 144.9 (CH), 142.4 (C), 131.5 (2 CH), 126.3 (C), 126.1 (CH), 125.2 (CH), 121.4 (CH), 118.7 (2 CH), 115.6 (C), 115.0 (CH), 111.9 (CH), 60.1 (CH_2_), 52.2 (2 CH_2_), 22.7 (2 CH_2_), 21.4 (CH_2_), 20.7 (CH_3_). LC-MS (ESI) *m*/*z* found: 399.4 [M + H]^+^. 

### 3.2. Biology

#### 3.2.1. Materials

Adenosine deaminase was purchased from Sigma Aldrich (St. Louis, MO, USA) (Ref: 10102105001). ZM-241385 and CGS-21680 were purchased from MedChem Express (Sollentuna, Sweden, Ref: HY-19532 and HY-13201A). The synthesis of MRS7416 is described in the article by Duroux et al. [38].

#### 3.2.2. Cell Culture and Membrane Preparation

For cell culture and membrane preparation, we were inspired by the protocol described in the article by Kecskes et al. [44]. HEK293T cells were cultured in DMEM supplemented with 10% FBS, penicillin (100 µg/mL), and streptomycin (100 U/mL) and incubated at 5% CO_2_ and 37 °C. Cells were transfected with ADORA2A plasmid using LipoD293 transfection reagent (SigmaGen, Frederick, MD, USA). Then, 48 h after transfection, cells were lysed with 500 µL of lysis buffer (50 mM Tris-HCl, pH 7.5, 10 mM MgCl_2_ supplemented with protease inhibitors) per 10 cm dish, transferred to a 15 mL conical tube, and incubated for 30 min on ice. The suspension was homogenized with a Branson sonifier with an output of 2 and duty cycle of 50% for 20 s and then was subcellular fractionated to recover the membrane-rich fraction. First, centrifugation at 750 RCF was performed to pellet the nuclei and cell debris, the resultant supernatant was centrifuged at 10,000 RCF for 10 min to pellet the microsomes, and finally the supernatant was centrifuged at 100,000 RCF for 1 h to precipitate the membranes. All the centrifugation steps were performed at 4 °C. The resultant pellet was resuspended in lysis buffer supplemented with 3 U/mL adenosine deaminase and homogenized by brief sonication. The suspension was divided into small aliquots to prevent several freeze–thaw cycles and stored at −80 °C until the binding experiments. The protein concentration was measured using the Bradford assay and adjusted to 9 mg/mL.

#### 3.2.3. FP Binding Assay

Assays were performed in Greiner 384-well black clear-bottom plates. The binding buffer used contained 50 mM Tris-HCl, pH 7.5, and 10 mM MgCl_2_. Competition assays were performed with 2 µL of MRS7416 (final concentration was 10 nM), 2 µL of competitor (final concentration 10 µM–0.1 nM), and 16 µL of A_2A_R membranes, diluted in buffer solution (final concentration 0.8 mg/mL) in the binding buffer for a total volume of 20 µL. The plate was incubated at 30 °C for 10 min. Fluorescence polarization was read on Clariostar Plus microplate reader (BMG Labtech, Offenburg, Germany) at λ_ex_ = 480 nm and λ_em_ = 520 nm. Data analysis was performed with GraphPad Prism Software, version 8.3.0 (GraphPad Software, Inc., San Diego, CA, USA), and *K_i_* values were calculated using the Cheng–Prusoff equation. The *K*_D_ (*K*_D_ = 2 nM) for A_2A_R membrane preparation was obtained by a kinetic on/off experiment. Displacement reference curves were performed with ZM241385 in accordance with the literature. All compounds were tested in three independent experiments, in duplicate.

#### 3.2.4. cAMP Assay

cAMP assays were conducted using the Lance Ultra cAMP Detection kit (Perkin Elmer, Waltham, MA, USA) in 384-well plates. Stimulation buffer containing 1X Hank’s Balanced Salt Solution (HBSS), 5 mM HEPES (pH 7.4), 0.1% BSA stabilizer, and 0.5 mM IBMX was prepared. A cAMP standard curve was prepared at 4x the desired final concentration in stimulation buffer, and 10 μL was added to the assay plate. Serial dilutions of compounds were also prepared at 4x the desired final concentration (30 µM–10 nM) in stimulation buffer, and 2.5 μL was added to the assay plate. Following the instructions in the Lance Ultra cAMP Detection kit, a single concentration of the agonist CGS21680, corresponding to the EC_80_, was prepared at 4x the desired final concentration (4.5 µM) in stimulation buffer. Then, 2.5 µL of this solution was added to the assay plate. The cells were detached by versene action, prepared at a concentration of 5.10^5^ cells per mL in stimulation buffer, and 5 μL was added to each well, except wells containing the cAMP standard curve. After incubating for 30 min at room temperature, Eu-cAMP tracer and uLIGHT-anti-cAMP working solutions were added per the manufacturer’s instructions. After 1 h of incubation at room temperature, the TR-FRET signal (ex 337 nm) was read on a Varioskan Lux multimode plate reader (Thermo Fisher Scientific, Asnières-sur-Seine, France). The TR-FRET signal (665 nm) was converted to fmol cAMP by interpolating from the standard cAMP curve. Fmol cAMP was plotted against the log of compound concentration, and data were fit to a three-parameter logistic curve to generate IC_50_ values (GraphPad Prism, GraphPad Software, Inc., San Diego, CA, USA). All compounds were tested in three independent experiments, in duplicate.

### 3.3. Molecular Docking

Molecular modelling studies were performed using AutoDock Vina software, using A_2A_ receptor co-crystallized structure of compound **1** (PDB: 8DU3) as described in R. Bolteau et al. [29]. Data analysis was performed with the UCSF ChimeraX software, version 1.6.1.

## 4. Conclusions

A new series of A_2A_R antagonists based on the quinazoline scaffold has been designed and synthesized. Structure–affinity relationship studies on compound **1** (*K_i_* (*h*A_2A_R) = 20 nM) at the C6- and C7-positions led to the identification of a highly potent hit compound, **5m**, with an impressive affinity of 5 nM for *h*A_2A_R. Functional activity assays confirmed the antagonistic effect of **5m**, demonstrating a half-maximal inhibitory concentration (IC_50_) of 6 µM.

The introduction of aminopentylpiperidine and 4-[(piperidin-1-yl)methyl]aniline chains preserved the binding affinities of the 2-amino-substituted quinazolines (**9w**, *K_i_* = 61 nM; **9x**, *K_i_* = 21 nM; **10c**, *K_i_* = 65 nM; **10d**, *K_i_* = 15 nM). These modulations also maintained antagonistic activities close to the micromolar range for compounds **9x** (IC_50_ = 9 µM) and **10d** (IC_50_ = 5 µM), similar to compound **5m** (IC_50_ = 6 µM), while improving their solubility. These findings underscore the beneficial effects of C2-position substitutions in quinazolines.

These quinazoline derivatives demonstrated significant potential as potent A_2A_R antagonists with favorable physicochemical properties. Future studies will focus on testing the most promising compounds in various cell models to assess their applicability in treating neurodegenerative diseases and cancer.

## Figures and Tables

**Table 1:** Modulations at the C4-, C6-, and C7-positions of quinazoline scaffold.

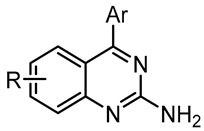				
Compound	R	Ar	*h*A_2A_AR (*K_i_* nM) ^a^	cAMP Assay IC_50_ (µM) ^b^
ZM-241385			3 ± 1	0.1 ± 0.02
**1**	6-Br	2-furane	23 ± 7	>30
**5a**	6-Br	4-fluorophenyl	220 ± 64	n.d.
**5b**	6-Br	Phenyl	92 ± 17	n.d.
**5c**	6-CH_3_	2-furane	45 ± 7	>30
**5d**	6-CH_3_	4-fluorophenyl	283 ± 74	n.d.
**5e**	6-CH_3_	Phenyl	83 ± 4	n.d.
**5f**	6-OMe	2-furane	>10,000	n.d.
**5g**	6-F	2-furane	>10,000	>30
**5h**	6-Cl	2-furane	28 ± 8	>30
**5i**	6-(4-fluorophenyl)	4-fluorophenyl	>10,000	n.d.
**5j**	6-phenyl	Phenyl	318 ± 23	n.d.
**5k**	7-Br	2-furane	28 ± 9	>30
**5l**	7-Br	4-fluorophenyl	150 ± 27	n.d.
**5m**	7-CH_3_	2-furane	5 ± 2	6 ± 1
**5n**	7-CH_3_	4-fluorophenyl	73 ± 9	n.d.
**5o**	7-CH_3_	Phenyl	45 ± 6	>30
**5p**	7-Cl	2-furane	20 ± 1	8 ± 6

^a^ *K_i_* values were determined from nonlinear analysis of competition curves using MRS7416 as fluorescent probe and are expressed as mean ± SEM of three independent experiments, in duplicate. Dose-response curve of ZM241385 was provided in Figure S2. FP competition binding curves were provided in Figure S3. ^b^ Ultra LANCE cAMP assay, data are expressed as mean ± SEM of three independent experiments, in duplicate. n.d.: not determined. Some cAMP assay curves were provided in Figure S4.

**Table 2:** Modulations at the C2-position of quinazoline scaffold.

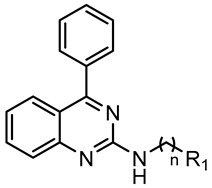							
Cpd.	n	R_1_	*h*A_2A_AR (*K_i_* nM) ^a^	Cpd.	n	R_1_	*h*A_2A_AR (*K_i_* nM) ^a^
**11** [43]	0	H	203 ± 56	**9j**	5	Piperidine	294 ± 37
**10a**	0	2-pyridine	>10,000	**9k**	5	Morpholine	674 ± 96
**9a**	0	4-benzylpiperidine	1110 ± 412	**9l**	5	Methylpiperazine	790 ± 126
**10b**	0	4-(piperidin-1-ylmethyl)benzene	52 ± 16	**9m**	5	Piperazine	687 ± 192
**9b**	1	2-pyridine	>10,000	**9n**	5	Boc-piperazine	1218 ± 385
**9c**	1	Phenyl	>10,000	**9o**	5	Pyrrolidine	519 ± 44
**9d**	1	4-benzonitrile	>10,000	**9p**	5	Diethylamine	310 ± 107
**9e**	1	4-methoxyphenyl	>10,000	**9q**	5	Tetrahydroisoquinoline	297 ± 86
**9f**	2	4-phenol	1453 ± 405	**9r**	5	4-benzylpiperidine	927 ± 146
**9g**	2	Piperidine	1781 ± 315	**9s**	5	NH_2_	>10,000
**9h**	3	Piperidine	1335 ± 482	**9t**	6	Piperidine	233 ± 68
**9i**	4	Piperidine	469 ± 83	**9u**	7	Piperidine	834 ± 91

^a^ *K_i_* values were determined from nonlinear analysis of competition curves using MRS7416 as fluorescent probe and are expressed as mean ± SEM of three independent experiments, in duplicate. FP competition binding curves were provided in Figure S3.

**Table 3:** Modulations at the C2-position and solubility properties of 4-(furan-2-yl)quinazolin-2-amine derivatives.

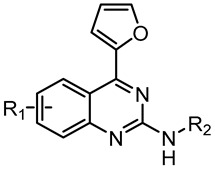					
Cpd.	R_1_	R_2_	*h*A_2A_AR (*K_i_* nM) ^a^	cAMP Assay IC_50_ (µM) ^b^	Solubility (mM) ^c^
**1**	6-Br	H	23 ± 7	>30	0.15
**9v**	6-Br	(CH_2_)_5_-piperidine	>10,000	n.d.	14.15
**5c**	6-CH_3_	H	45 ± 7	>30	0.10
**9w**	6-CH_3_	(CH_2_)_5_-piperidine	61 ± 15	14 ± 2	2.15
**10c**	6-CH_3_	4-[(piperidin-1-yl)methyl]benzene	65 ± 21	12 ± 2	10.3
**5m**	7-CH_3_	H	5 ± 2	6 ± 1	0.8
**9x**	7-CH_3_	(CH_2_)_5_-piperidine	21 ± 2	9 ± 5	12.0
**10d**	7-CH_3_	4-[(piperidin-1-yl)methyl]benzene	15 ± 2	5 ± 1	3.0

^a^ *K_i_* values were determined from nonlinear analysis of competition curves using MRS7416 as fluorescent probe and are expressed as mean ± SEM of three independent experiments, in duplicate. Dose-response curve of ZM241385 was provided in Figure S2. FP competition binding curves were provided in Figure S3. ^b^ Ultra LANCE cAMP assay, data are expressed as mean ± SEM of three independent experiments, in duplicate. Some cAMP assay curves were provided in Figure S4. ^c^ Solubility measured in PBS pH 7.4 starting from a 4 mg/mL solution in DMSO. n.d.: not determined.

## Data Availability

All data were provided in this manuscript and supplementary information section.

## Supplementary Materials

The following supporting information can be downloaded at: https://www.mdpi.com/article/10.3390/molecules29163847/s1, Figure S1: LC-MS and NMR spectra of compounds **5a**–**p**, **9a**–**x** and **10a**–**d**; Figure S2: Dose-response curve of ZM241385; Figure S3: FP competition binding experiments with compound 1 and some final compounds; Figure S4: cAMP assay of compounds **5m**, **9w**, **9x**, **10c**, **10d**.

## Abbreviations

A_2A_R, A_2A_ adenosine receptor; AD, Alzheimer’s disease; PD, Parkinson’s disease; GPCR, G protein-coupled receptor; RB, radioligand binding; FP, fluorescence polarization; IC_50_, inhibitory concentration; *K_i_*, inhibitory constant; SAR, structure–activity relationship; GABA, acid gamma-aminobutyrique; PDB, protein database; BINAP, 2,2′-bis(diphenylphosphino)-1,1′-binaphthyl; DCM, dichloromethane; TBABr, tetrabutylammonium bromide; DIPEA, *N*,*N*-diisopropylethylamine; DMF, *N*,*N*-dimethylformamide; IBMX, 3-isobutyl-1-méthylxanthine; FBS, fetal bovine serum; DMEM, Dulbecco’s Modified Eagle medium; RCF, relative centrifugal force; HEPES, 4-(2-hydroxyethyl)-1-piperazineethanesulfonic acid; *K*_D_, equilibrium dissociation constant; PE, petroleum ether; TFA, trifluoroacetic acid.
