# Supplementary material for: Novel Quinazoline Derivatives as Highly Effective A2A Adenosine Receptor Antagonists
Source: Molecules. 2024 Aug 14;29(16):3847. doi: 10.3390/molecules29163847 (PMC11357017; doi:10.3390/molecules29163847)
Supplement: Supplementary file 1 [file molecules-29-03847-s001.zip › molecules-3126652-supplementary.pdf]

# Novel quinazoline derivatives as highly effective $A_{2A}$ adenosine receptor antagonists

Amélie Laversin, Robin Dufossez[†], Raphaël Bolteau[†], Romain Duroux, Séverine Ravez, Sergio Hernandez-Tapia, Martin Fossart, Mathilde Coevoet, Maxime Liberelle, Saïd Yous, Nicolas Lebègue, Patricia Melnyk[*]

*Univ. Lille, Inserm, CHU Lille, U1172 - LilNCog - Lille Neuroscience & Cognition, F-59000 Lille, France.*

[†]*These authors contributed equally*

**Figure S1.** NMR and LC-MS spectra of final compounds

1H- and 13C-NMR Spectra in DMSO-d$_6$ of compound 5a

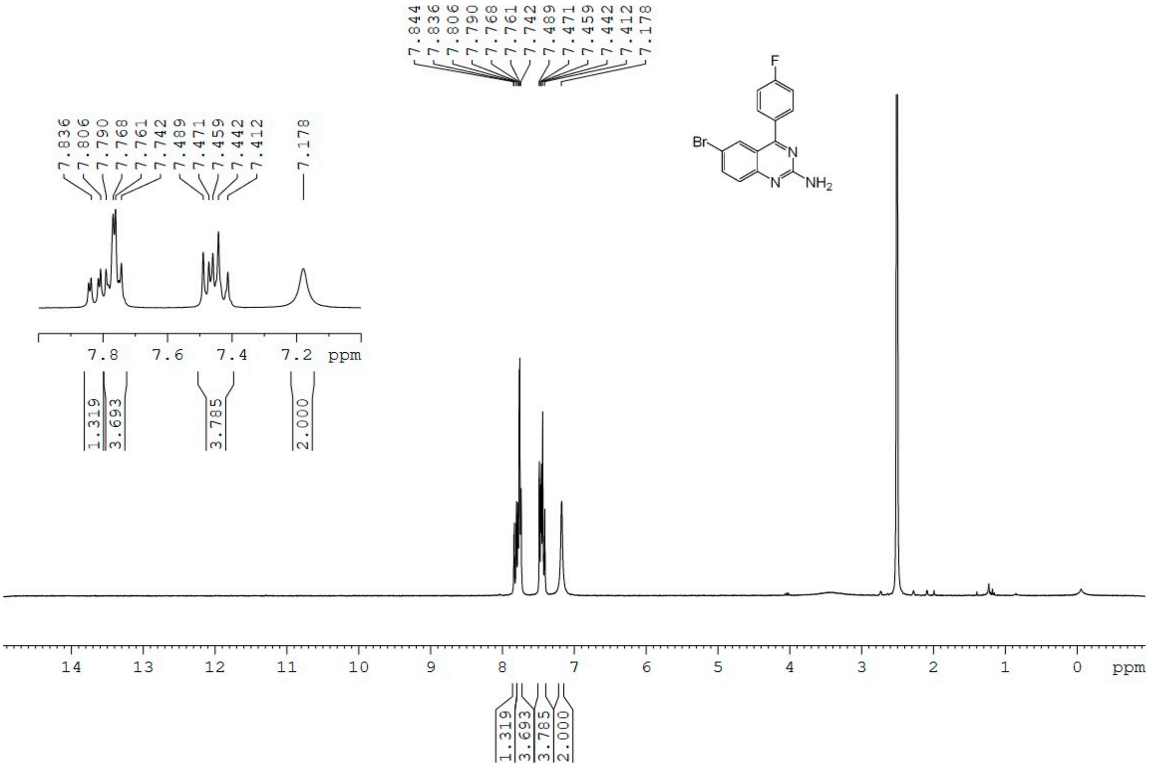


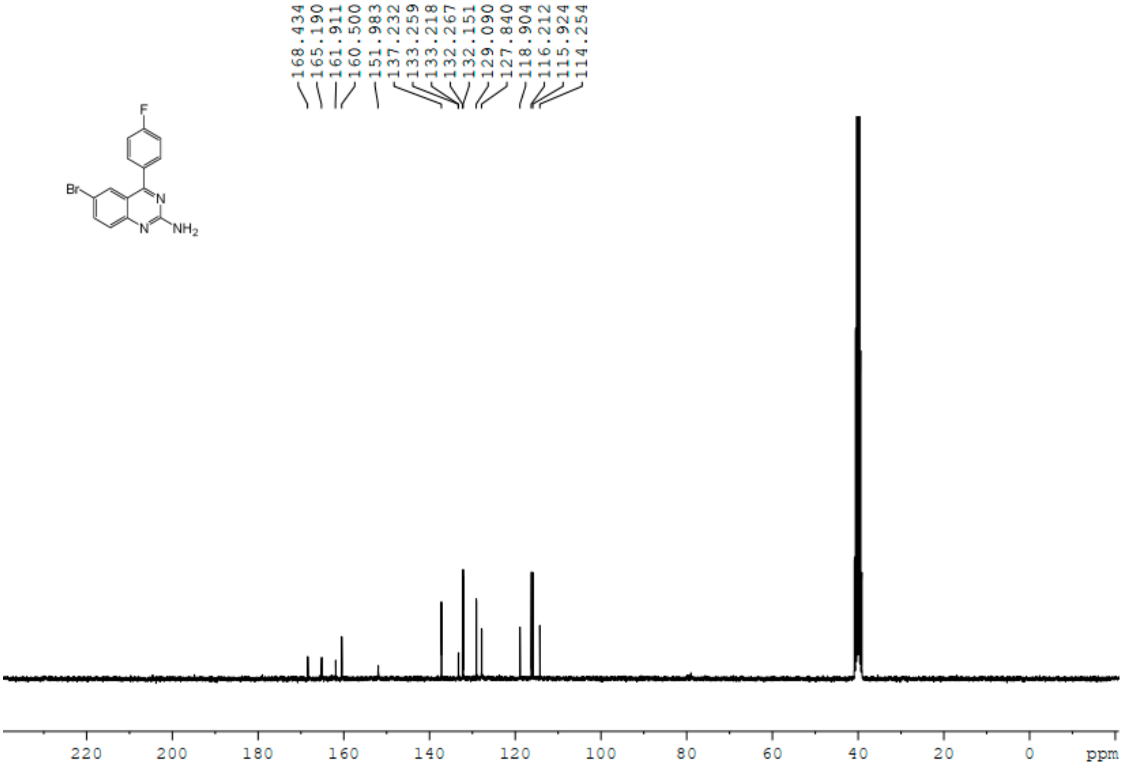

**LC-MS mass spectrum of compound 5a**

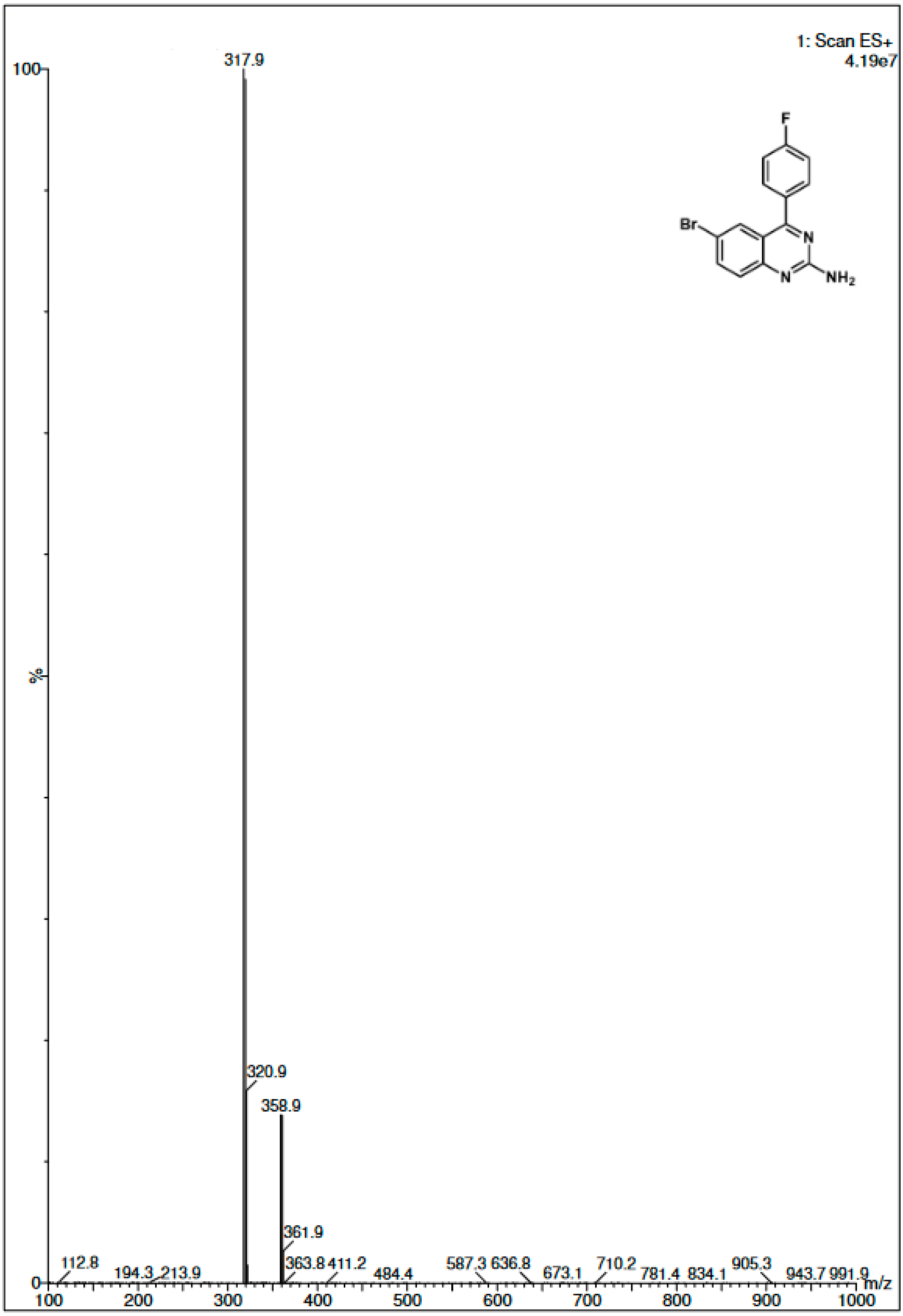

**1H- and 13C-NMR Spectra in DMSO-$d_6$ of compound 5b**

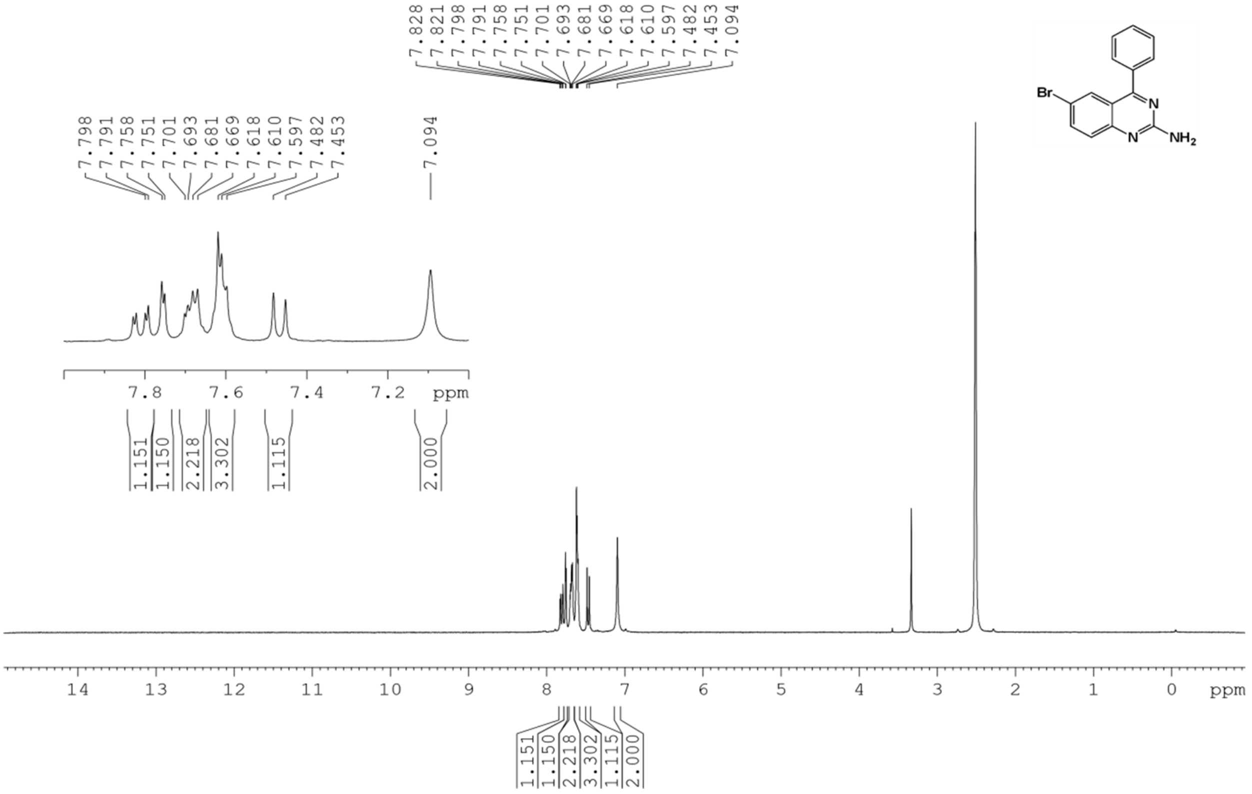


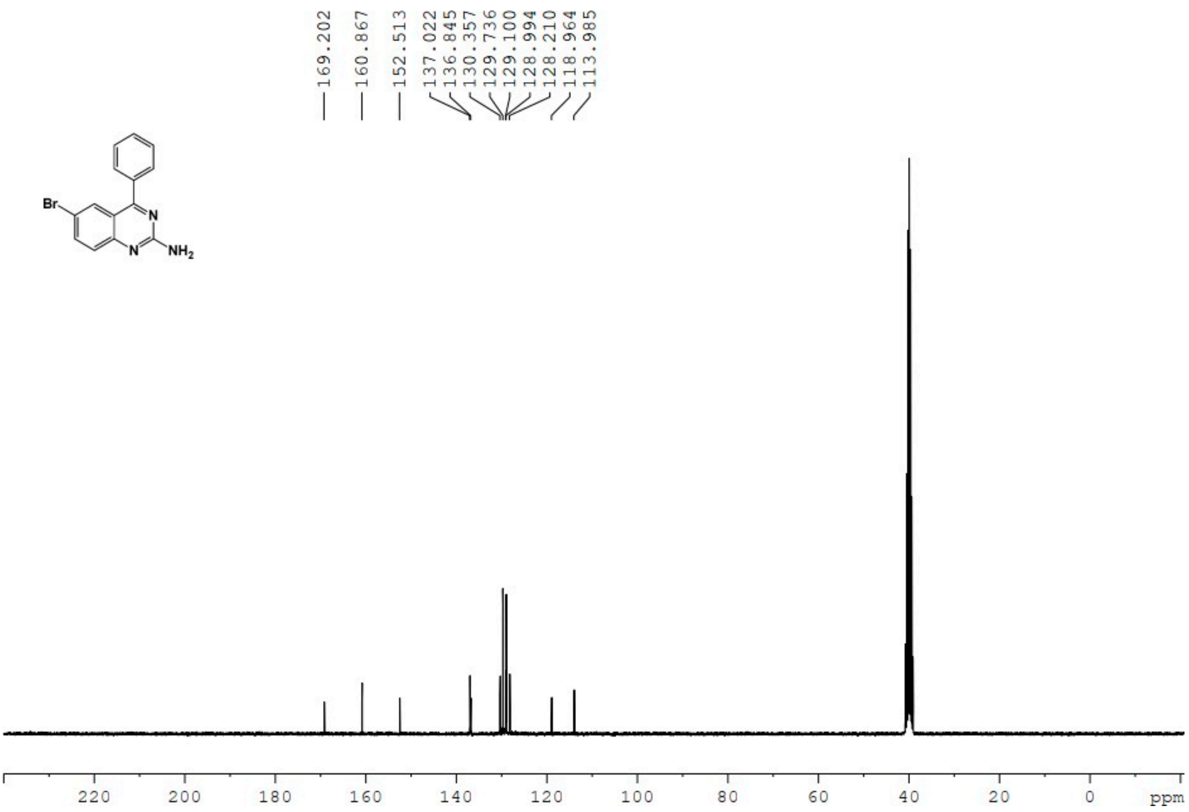

**LC-MS mass spectrum of compound 5b**

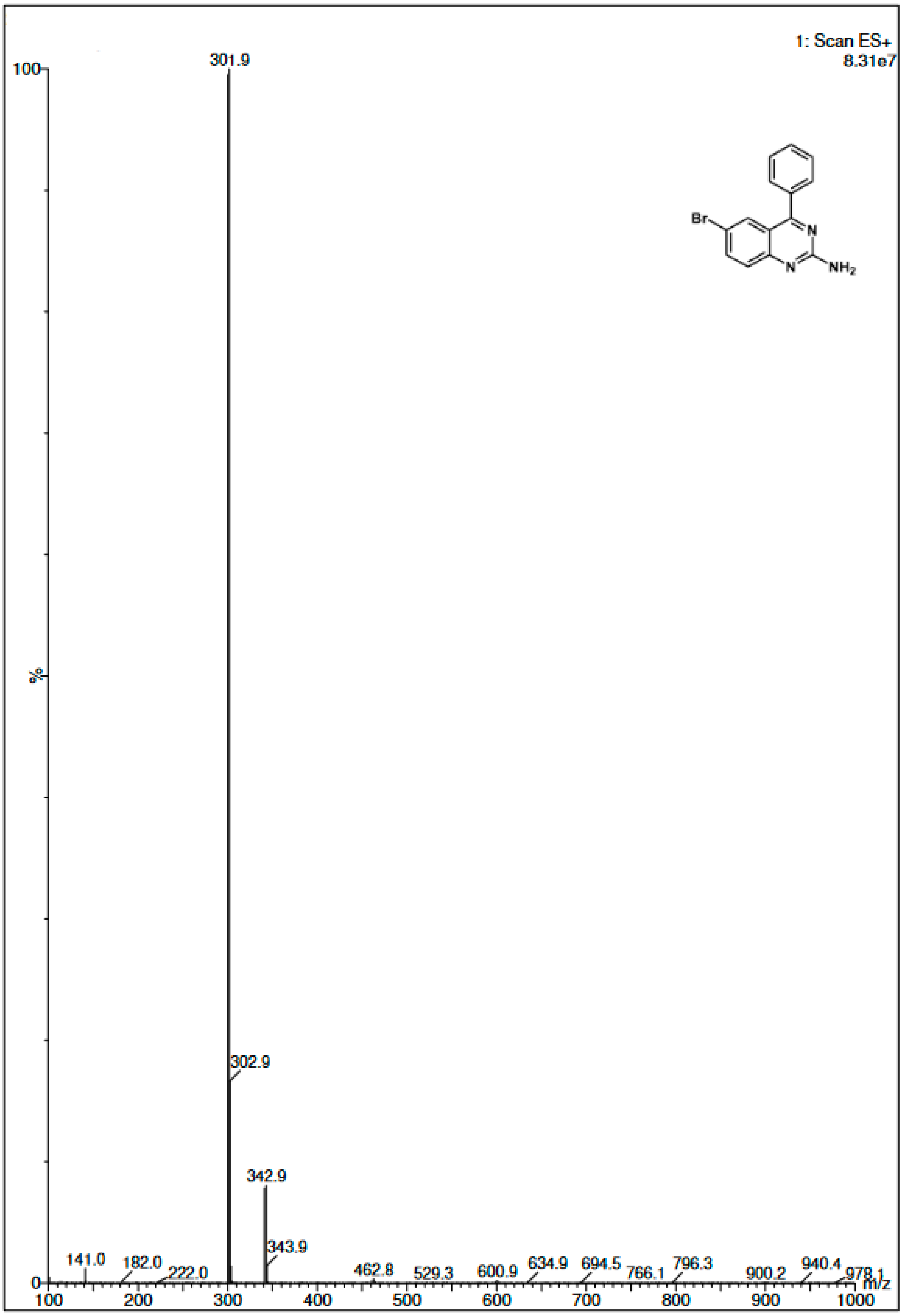

**1H- and 13C-NMR Spectra in $CDCl_3$ of compound 5c**

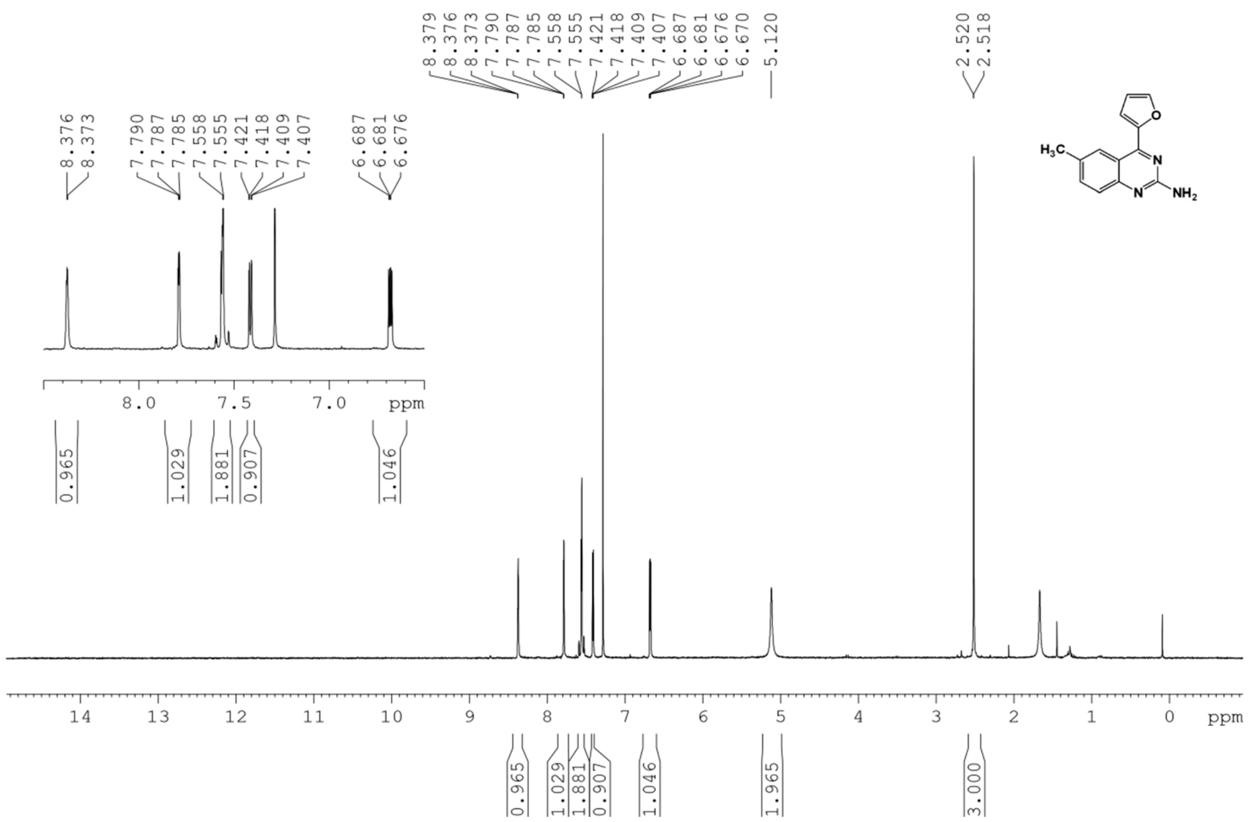


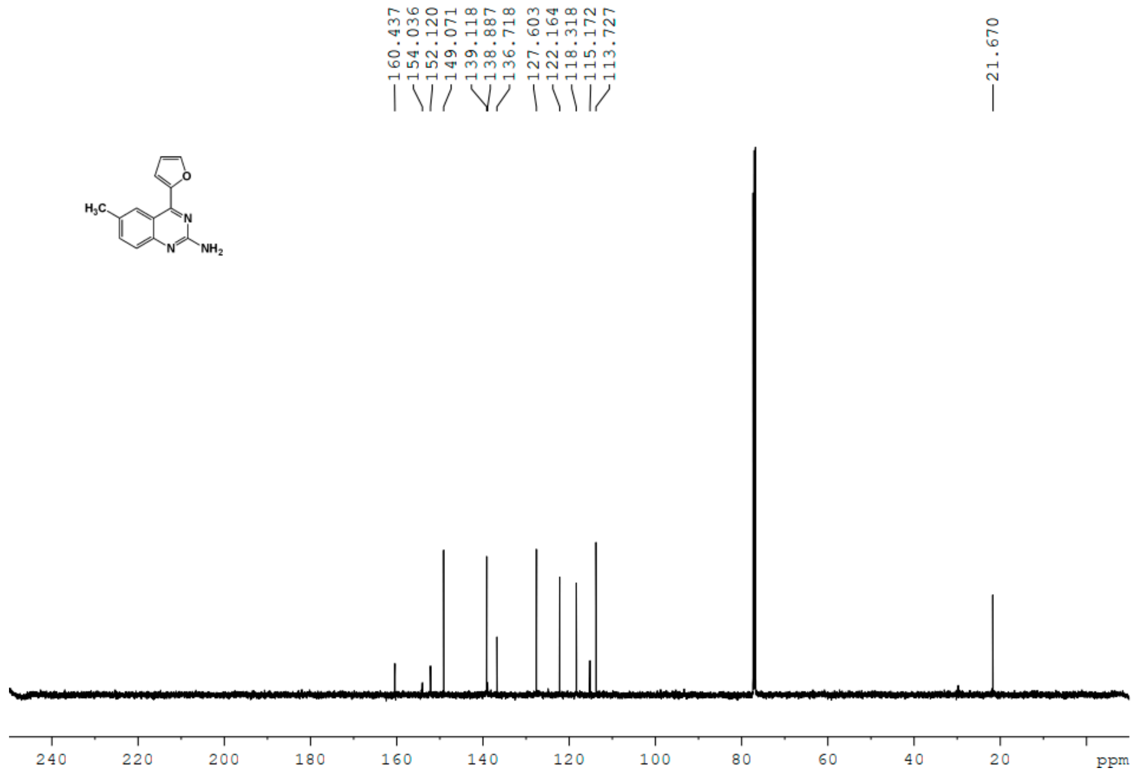

**LC-MS mass spectrum of compound 5c**

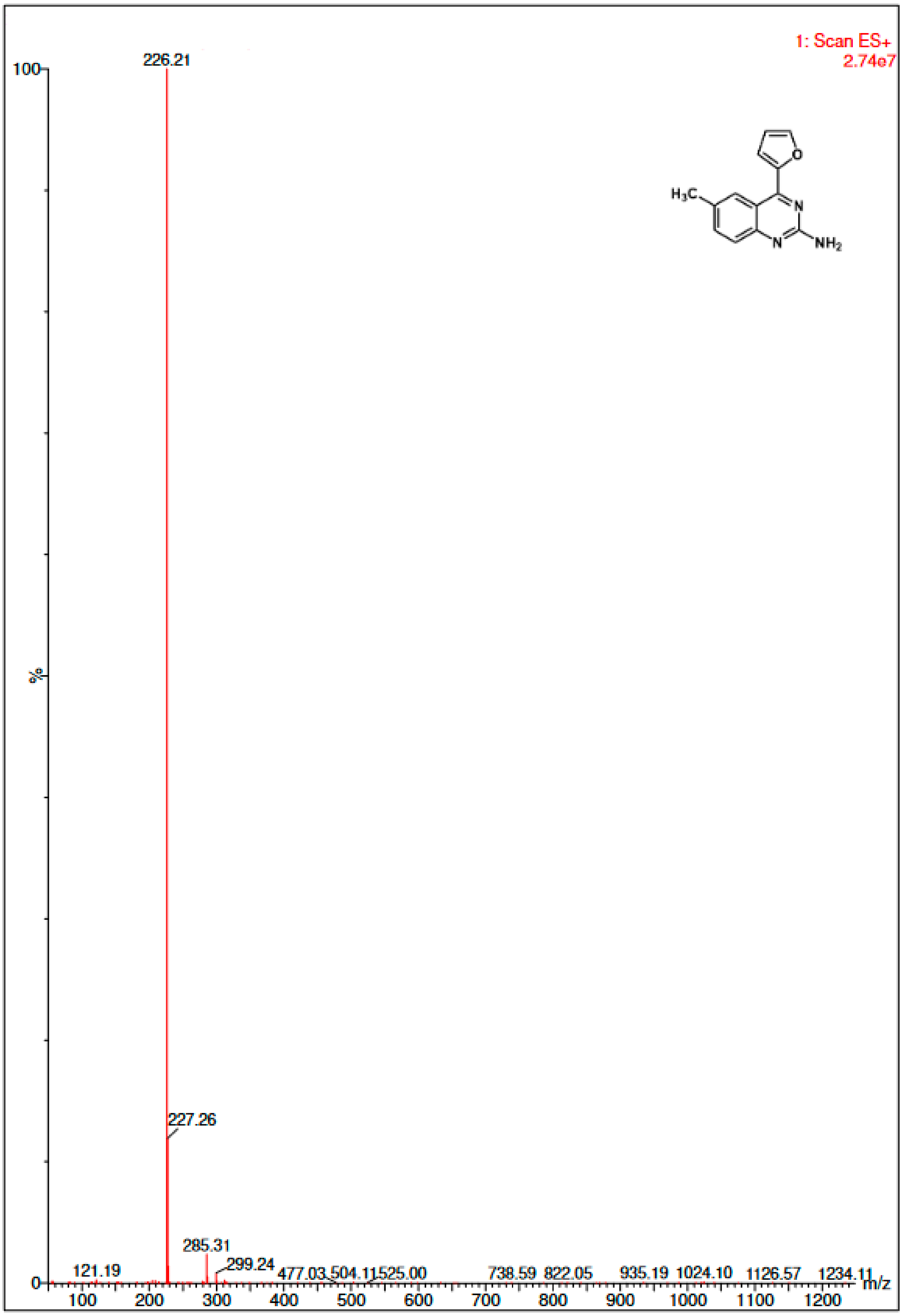

**1H- and 13C-NMR Spectra in DMSO-$d_6$ of compound 5d**

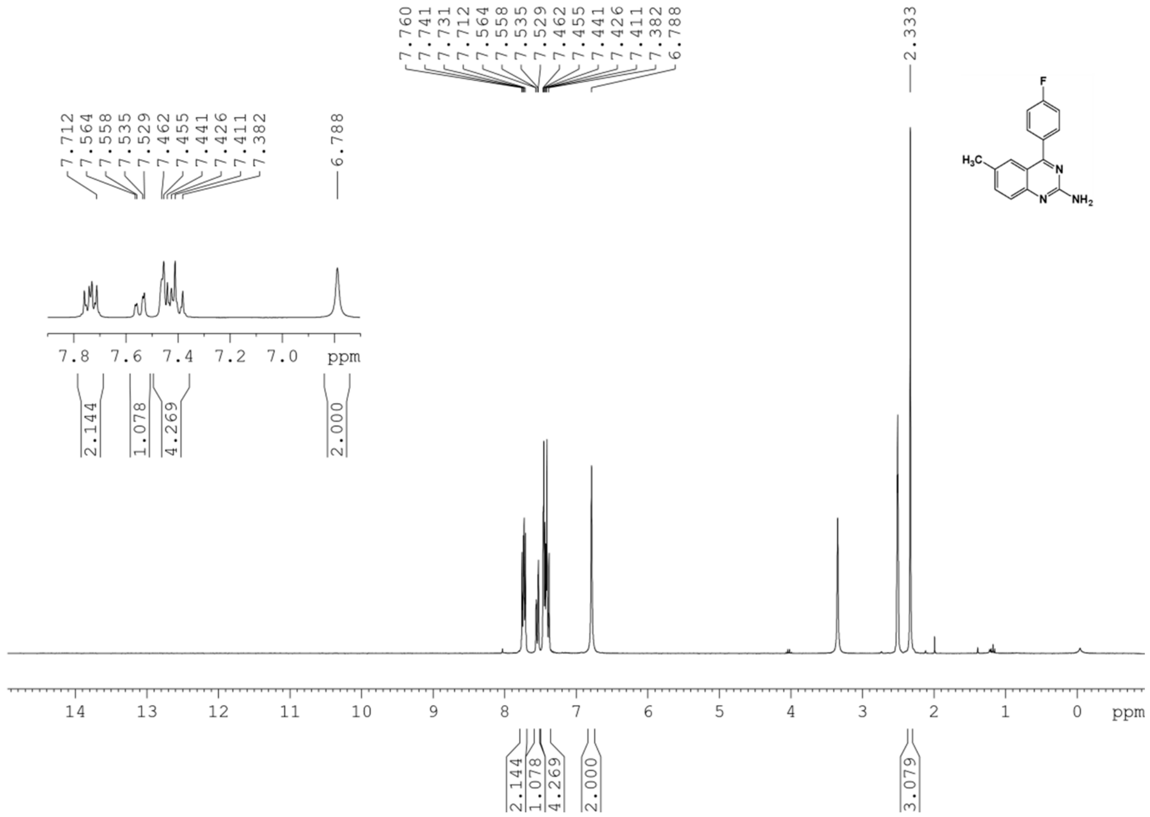


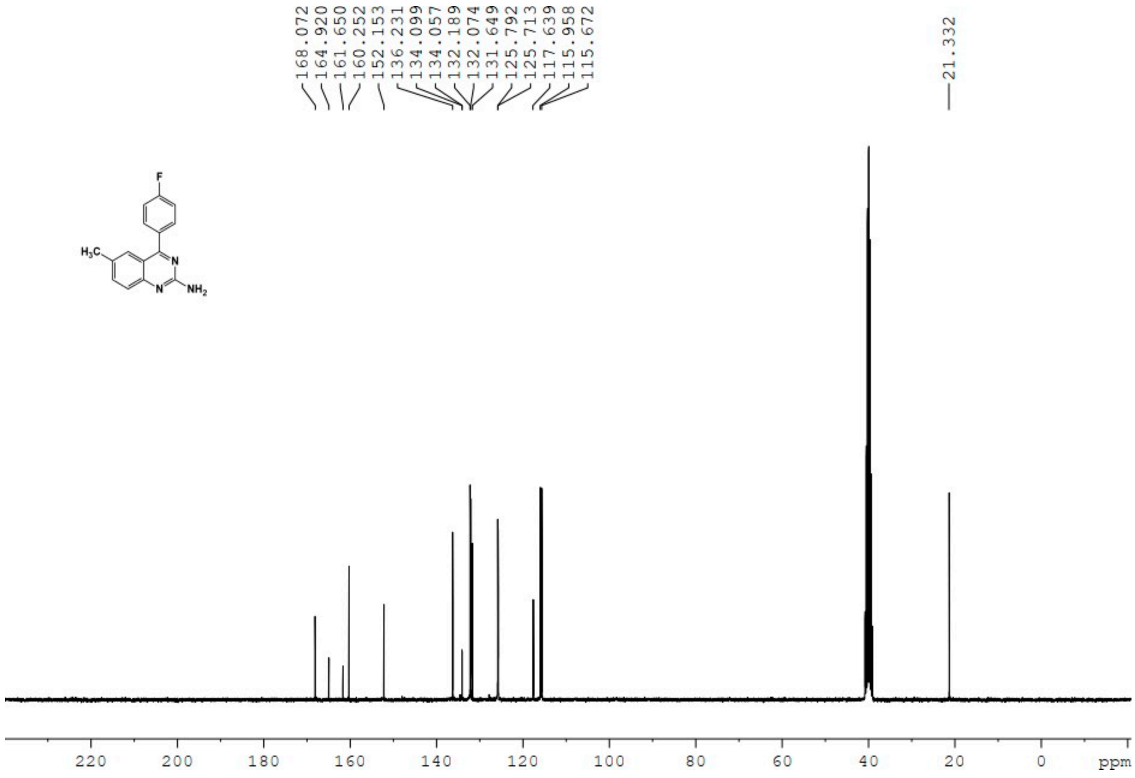

**LC-MS mass spectrum of compound 5d**

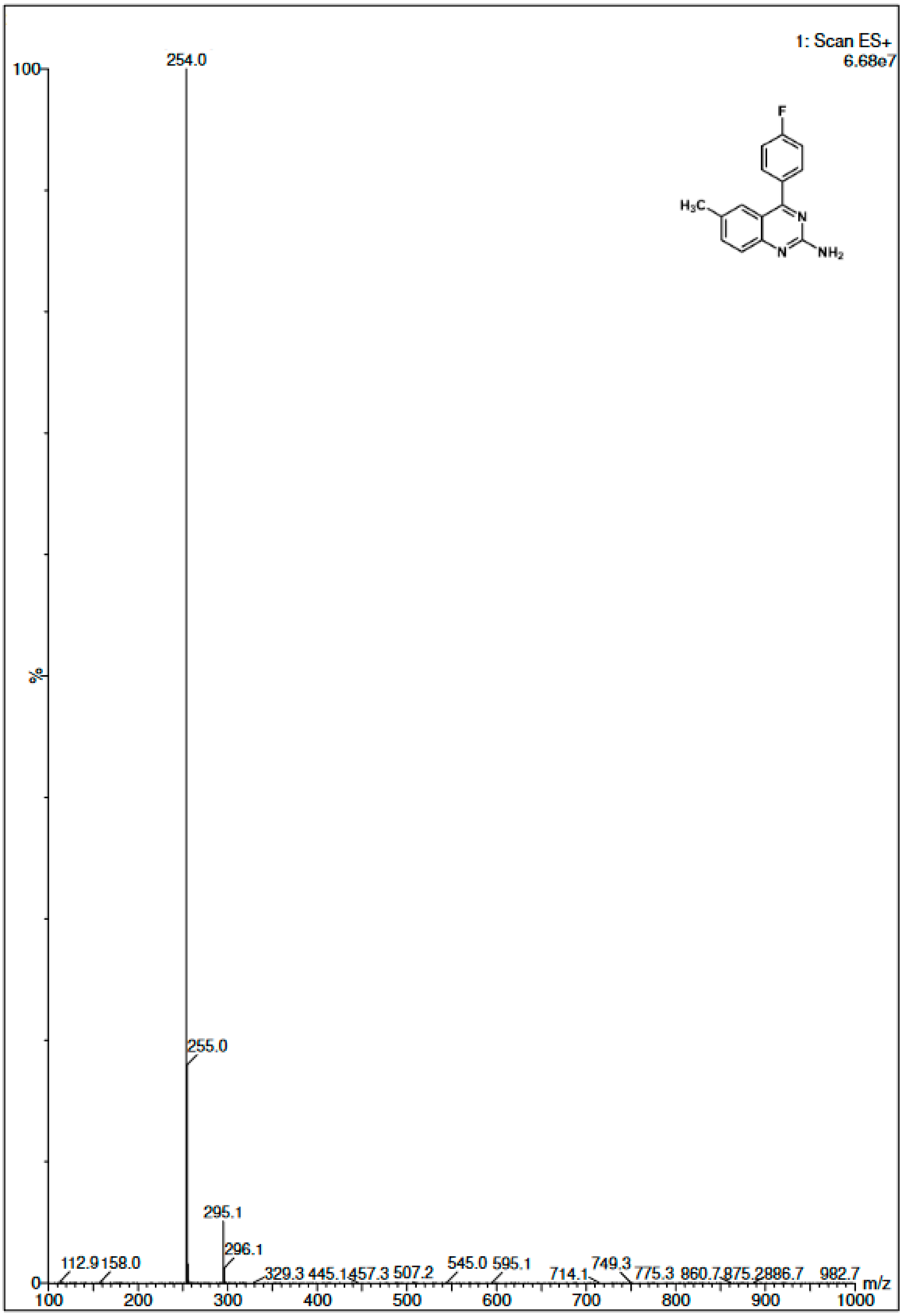

**1H- and 13C-NMR Spectra in DMSO-$d_6$ of compound 5e**

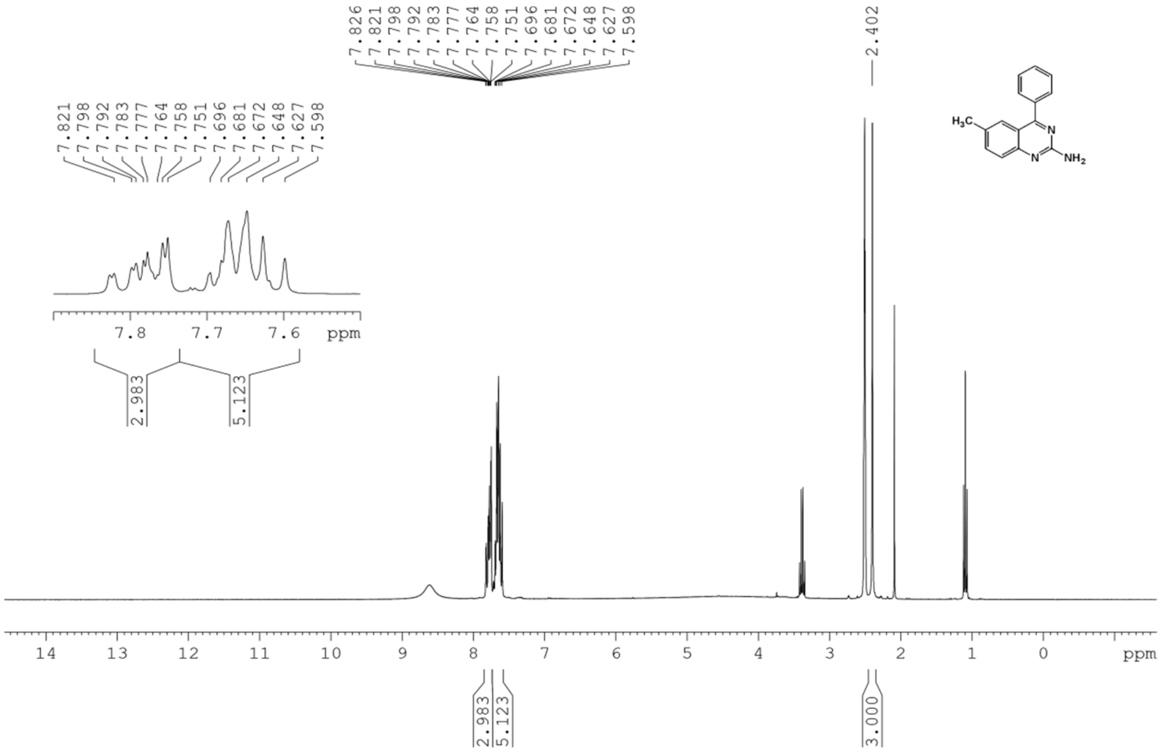


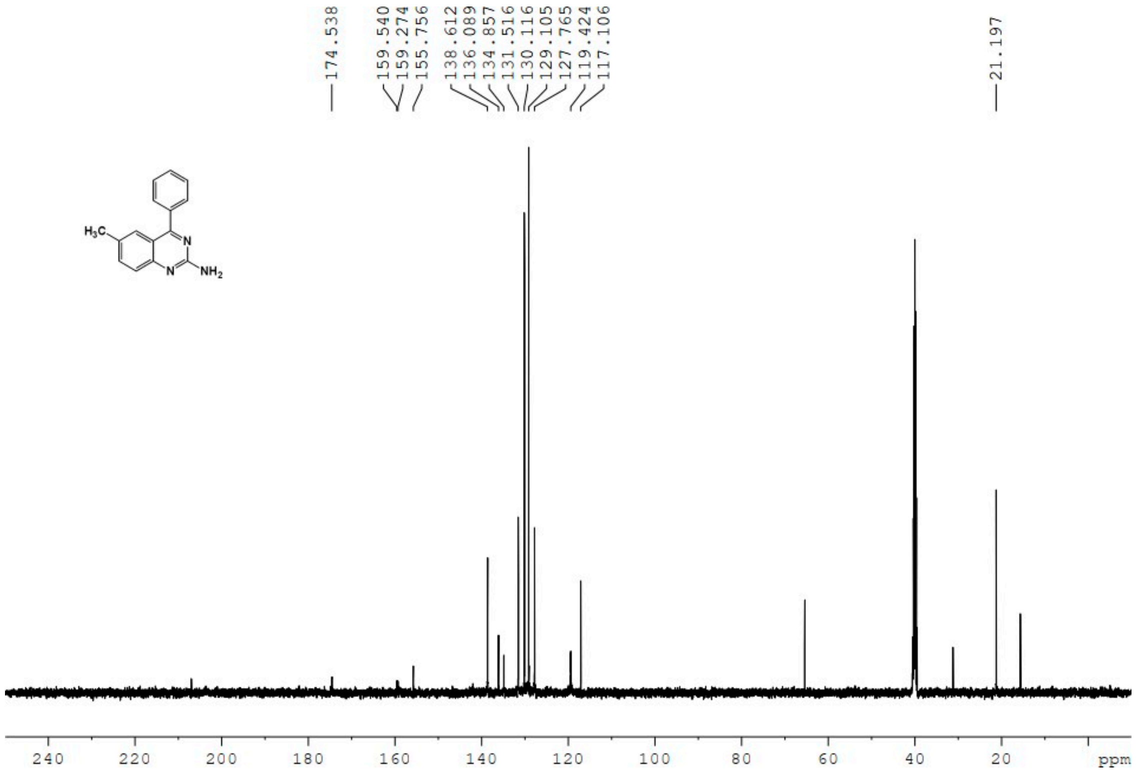

**LC-MS mass spectrum of compound 5e**

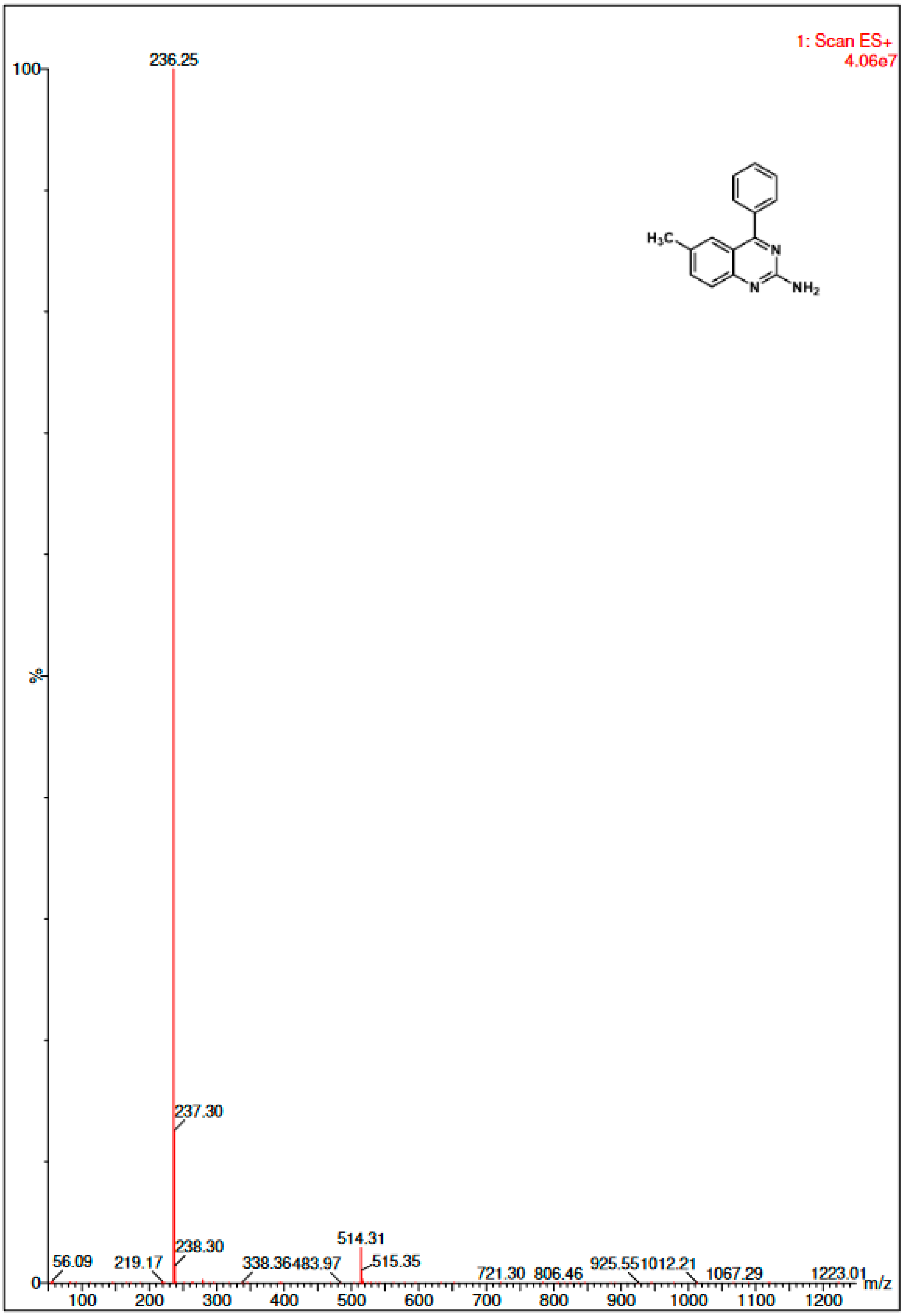

**1H- and 13C-NMR Spectra in DMSO-$d_6$ of compound 5f**

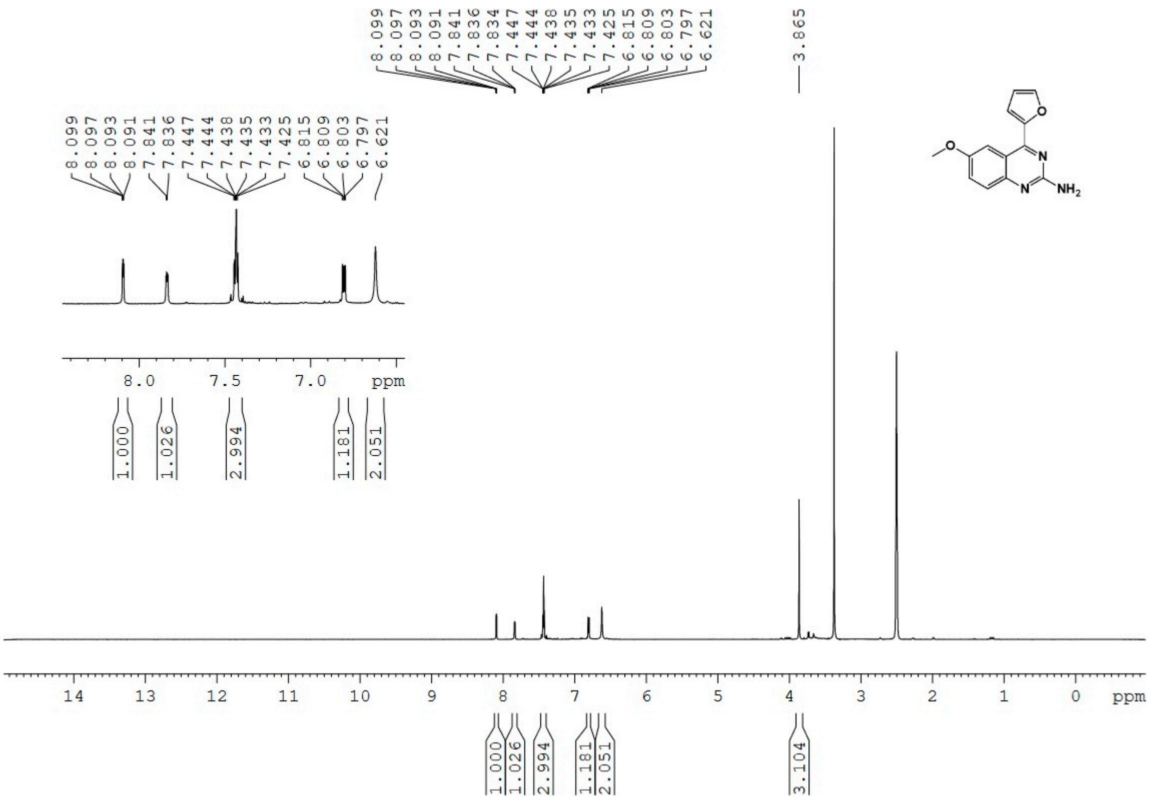


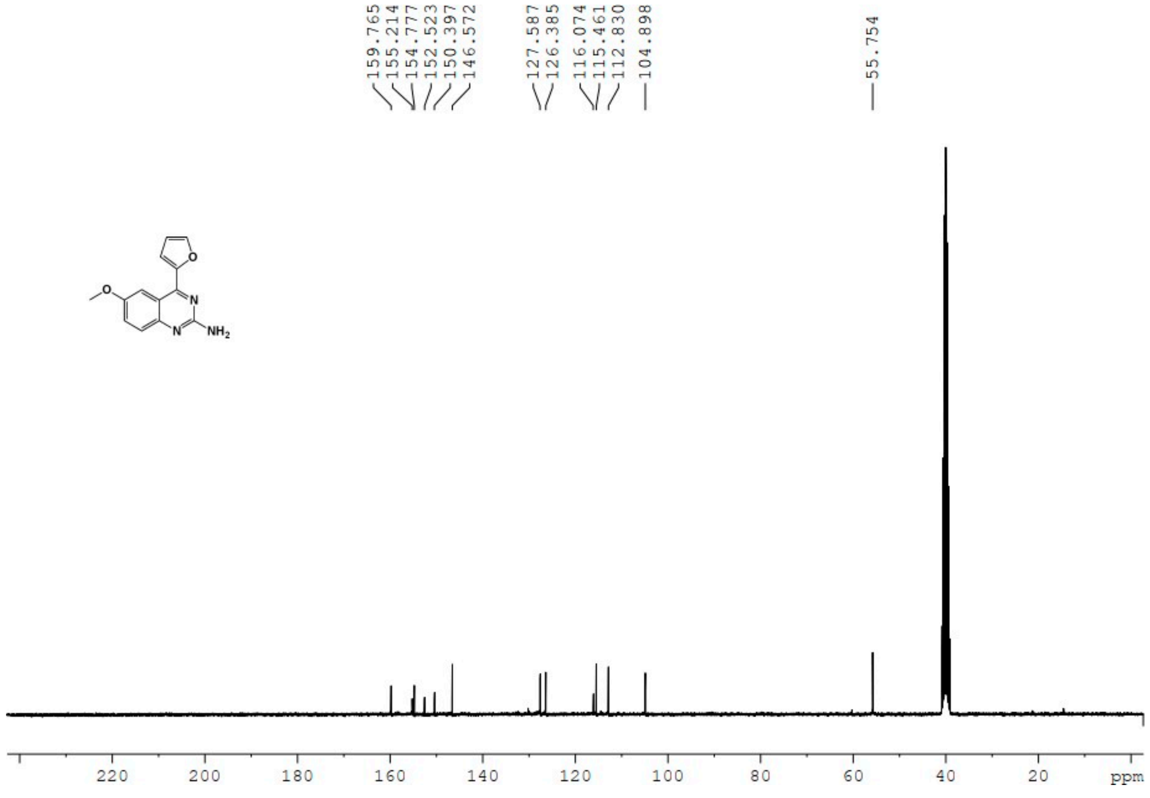

**LC-MS mass spectrum of compound 5f**

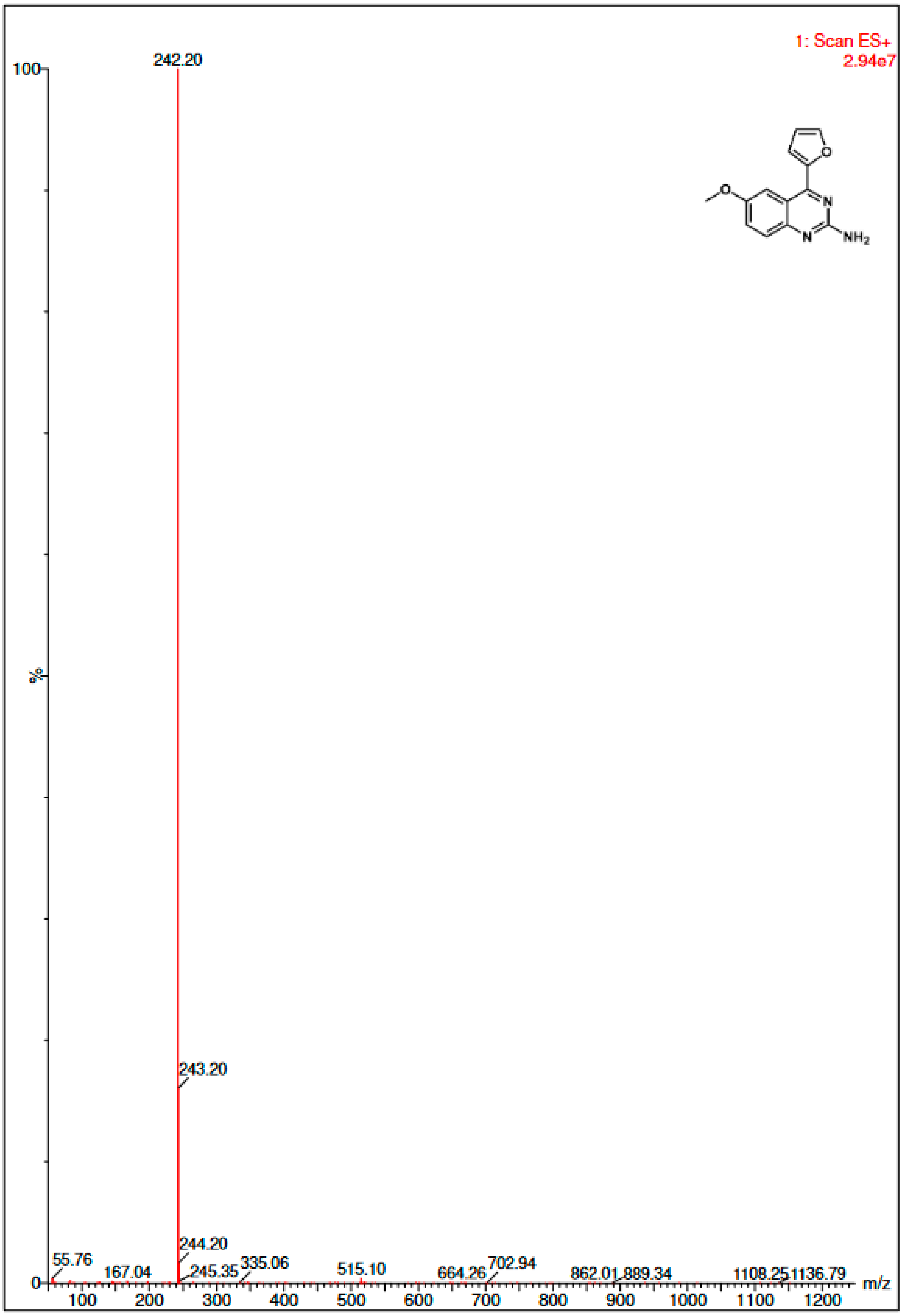

**1H- and 13C-NMR Spectra in DMSO-$d_6$ of compound 5g**

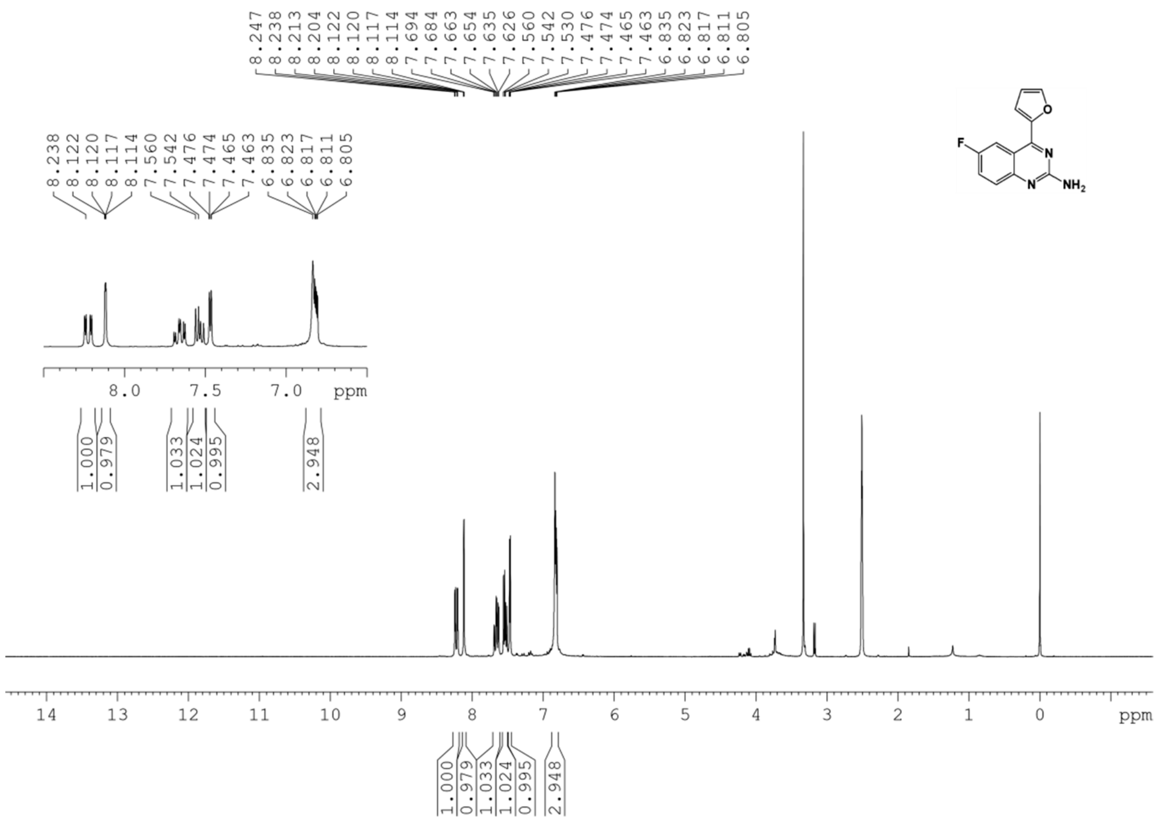


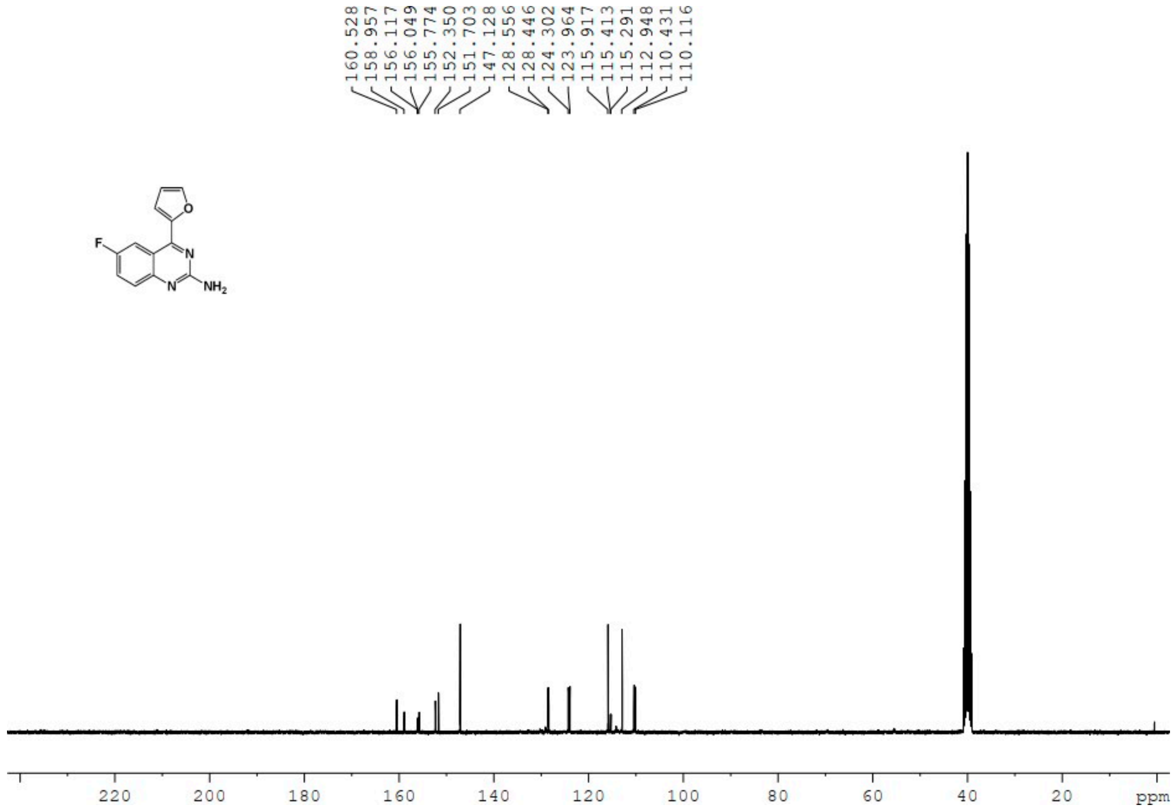

**LC-MS mass spectrum of compound 5g**

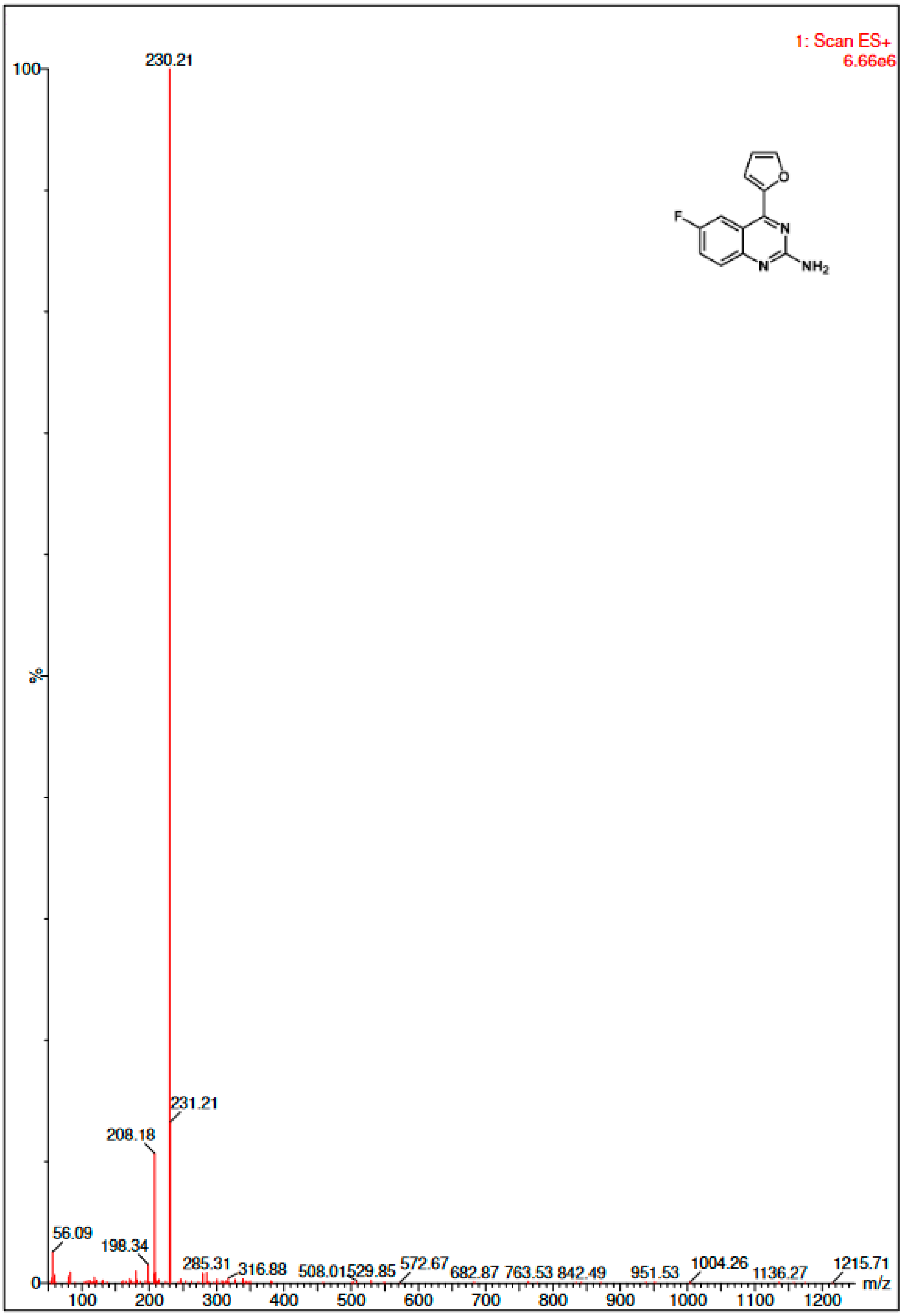

**1H- and 13C-NMR Spectra in DMSO-$d_6$ of compound 5h**

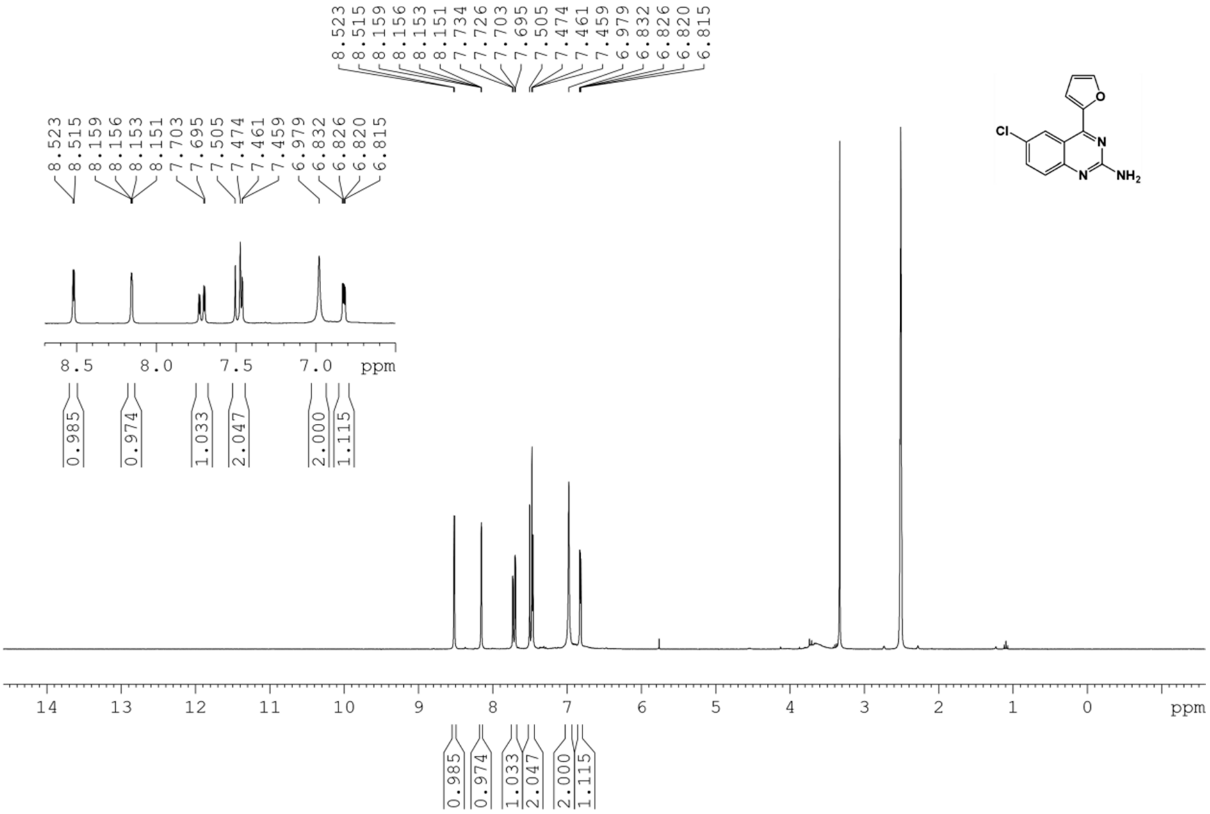


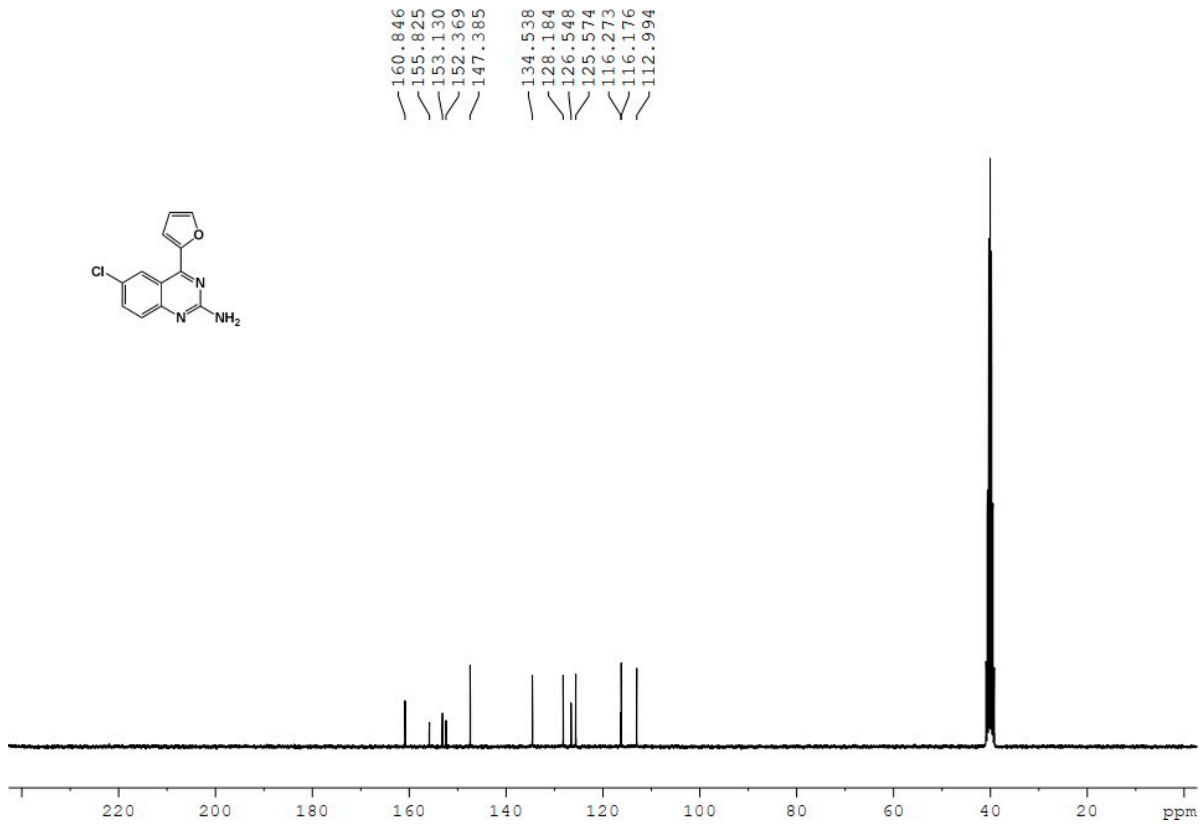

**LC-MS mass spectrum of compound 5h**

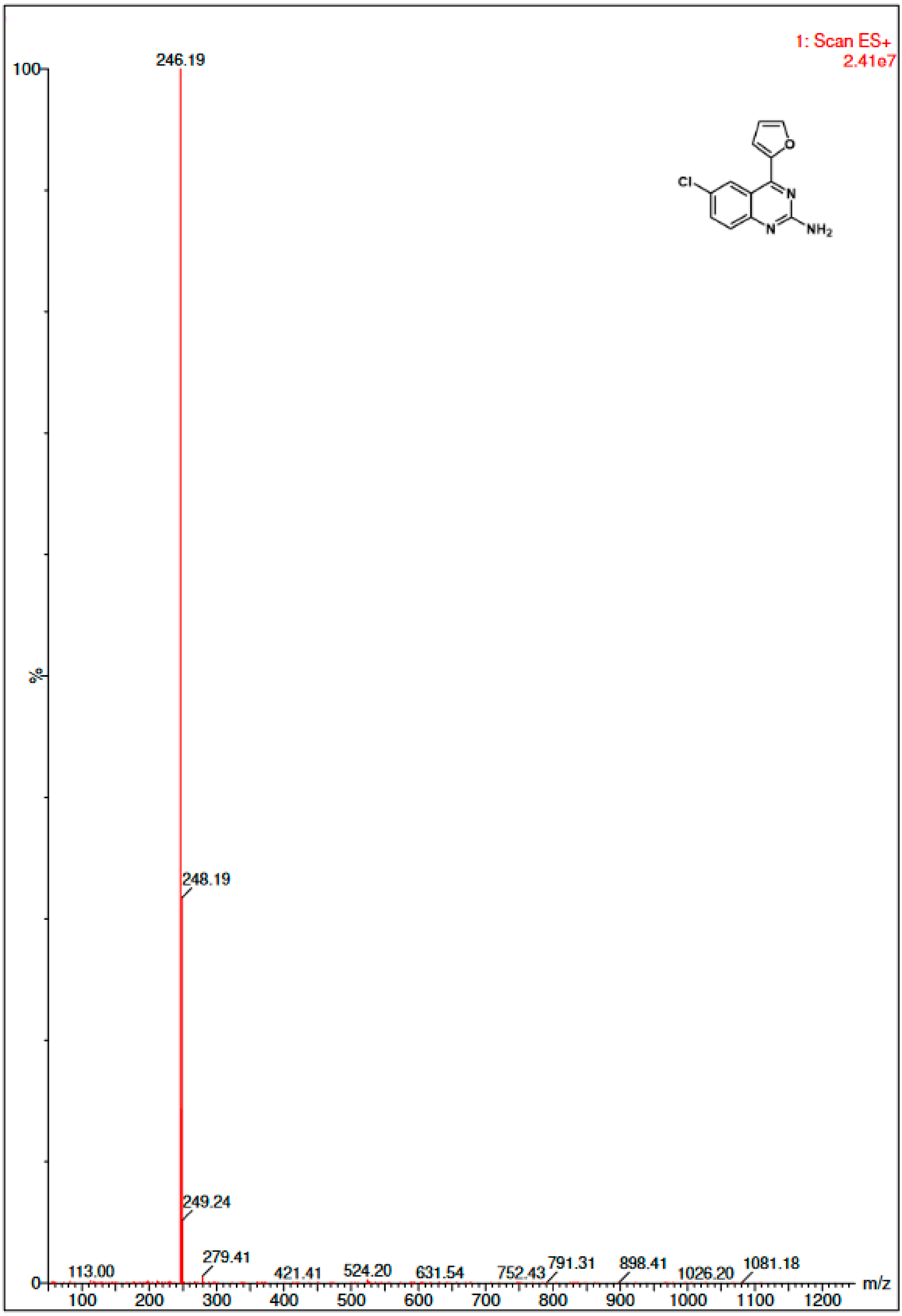

**1H- and 13C-NMR Spectra in DMSO-$d_6$ of compound 5i**

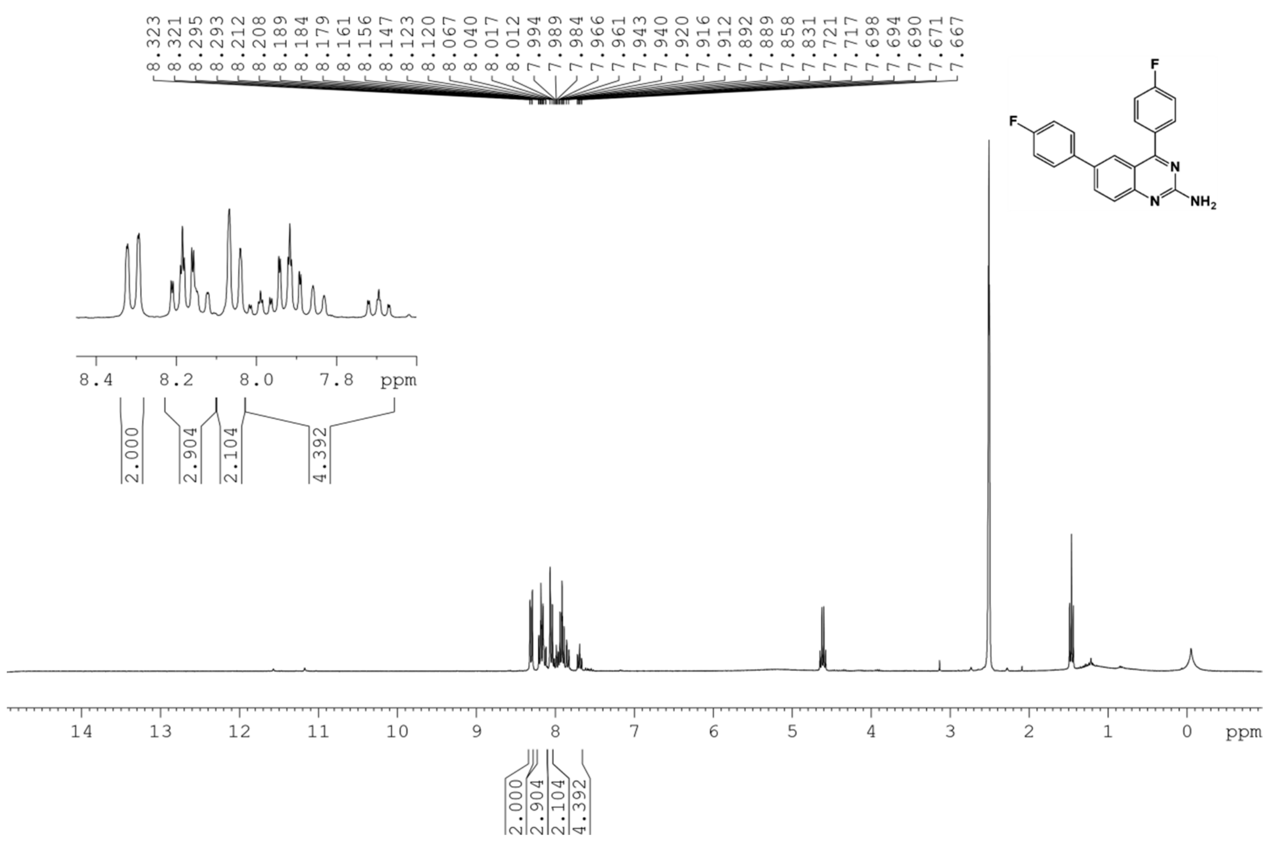

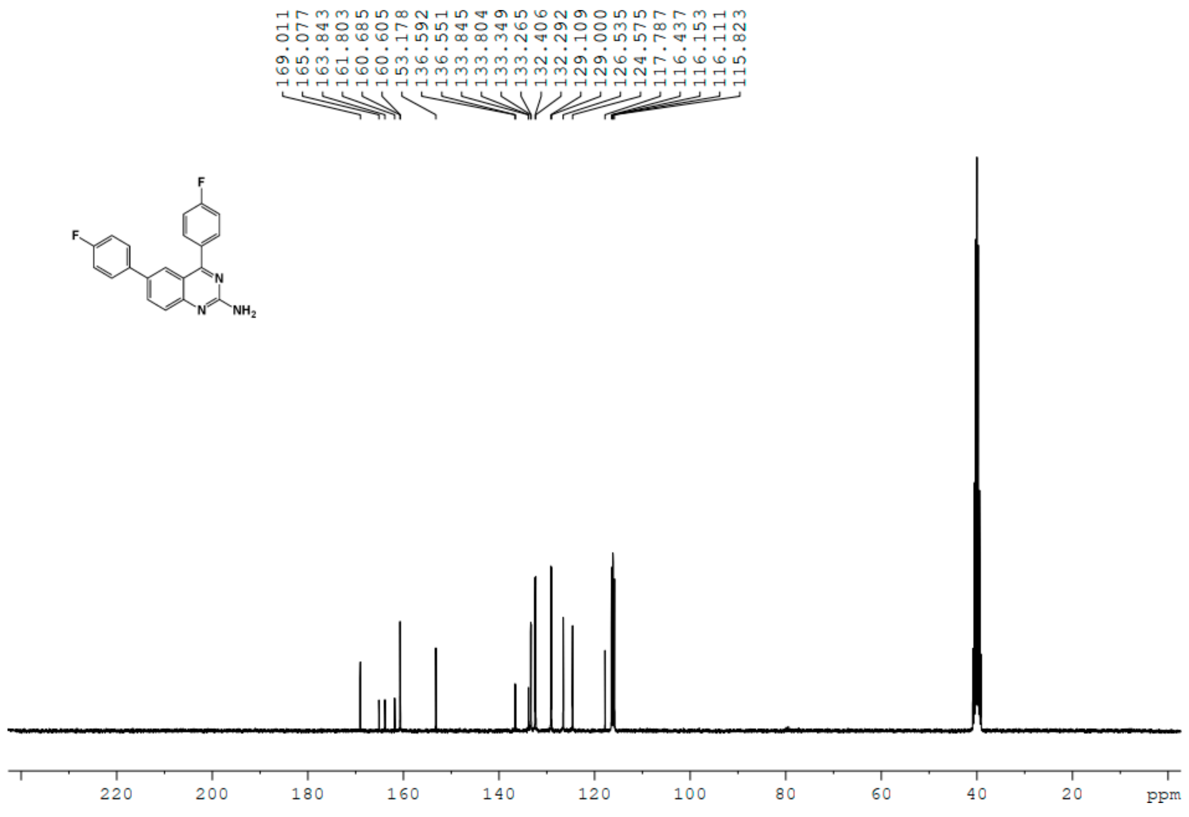

**LC-MS mass spectrum of compound 5i**

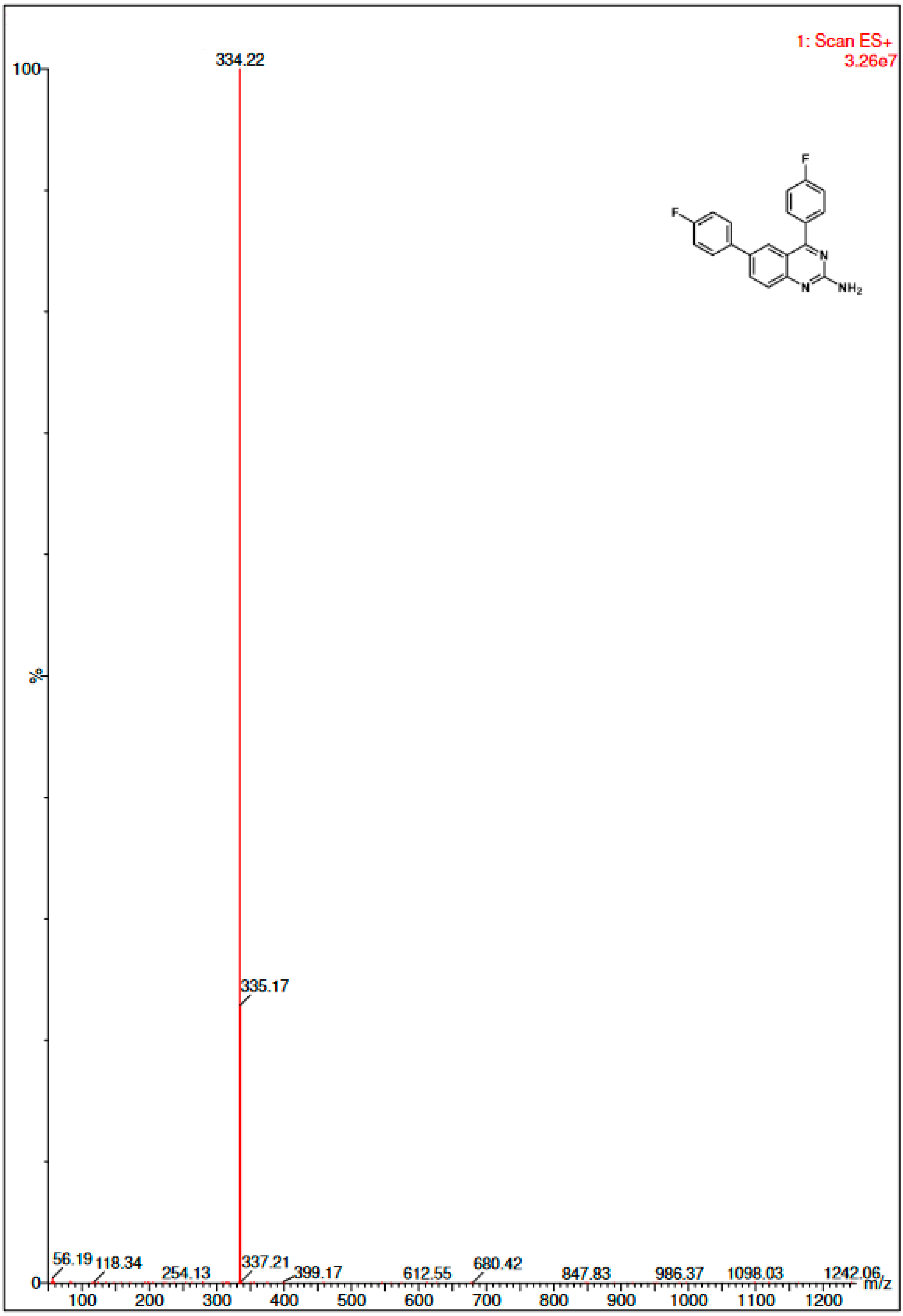

**1H- and 13C-NMR Spectra in $CDCl_3$ of compound 5j**

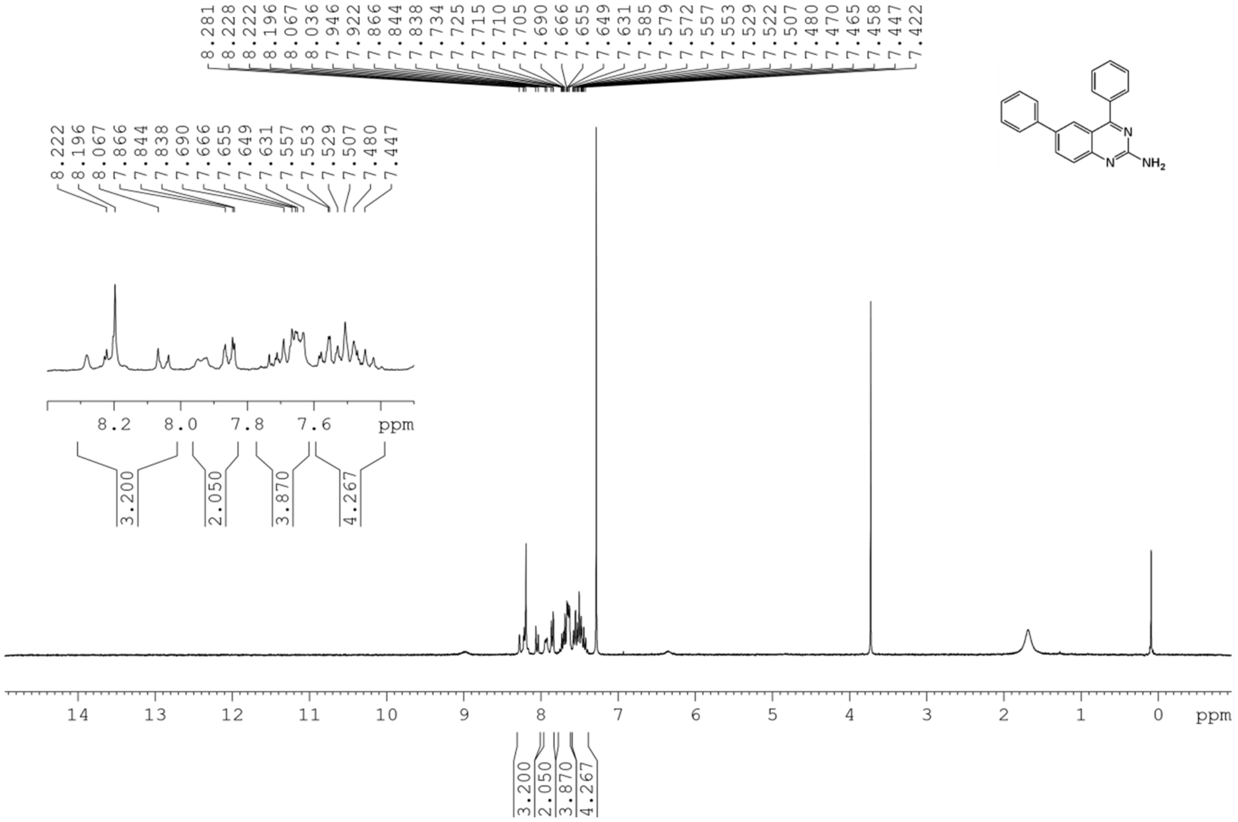


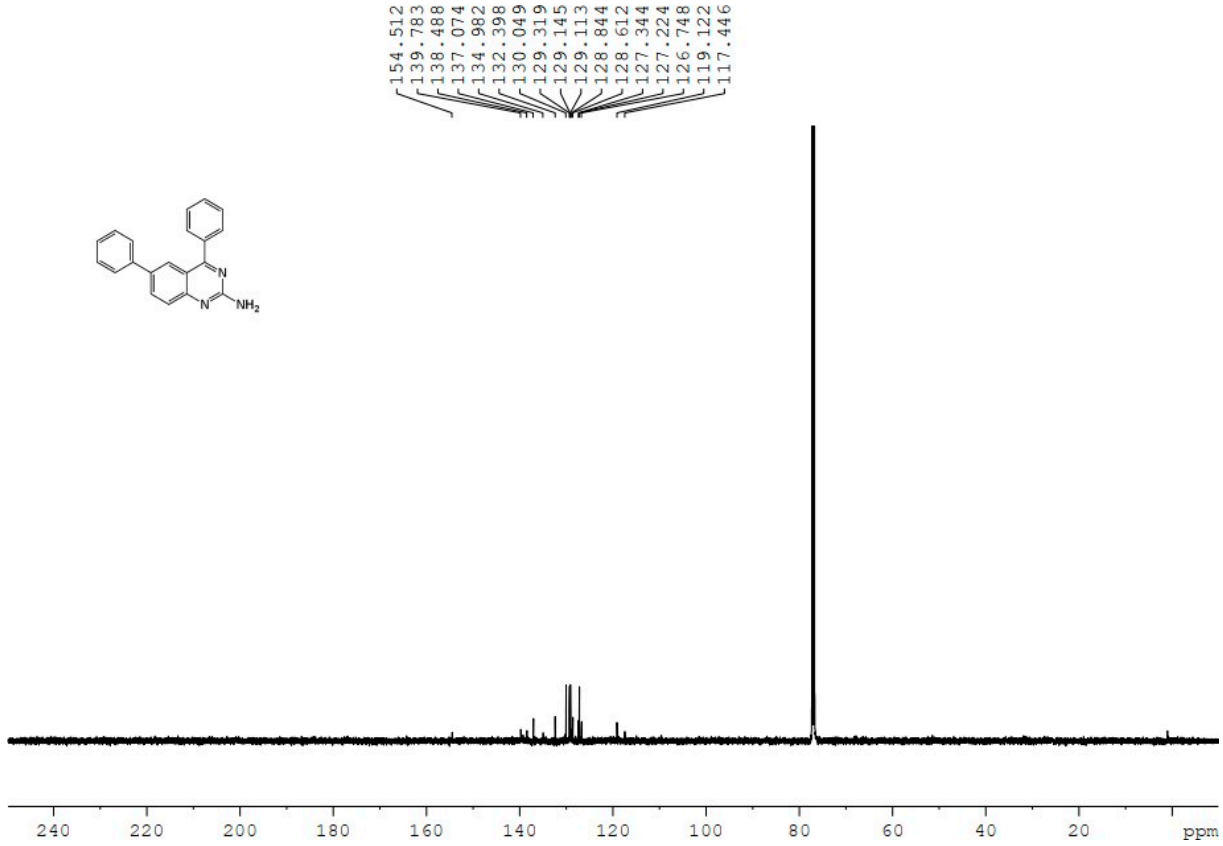

**LC-MS mass spectrum of compound 5j**

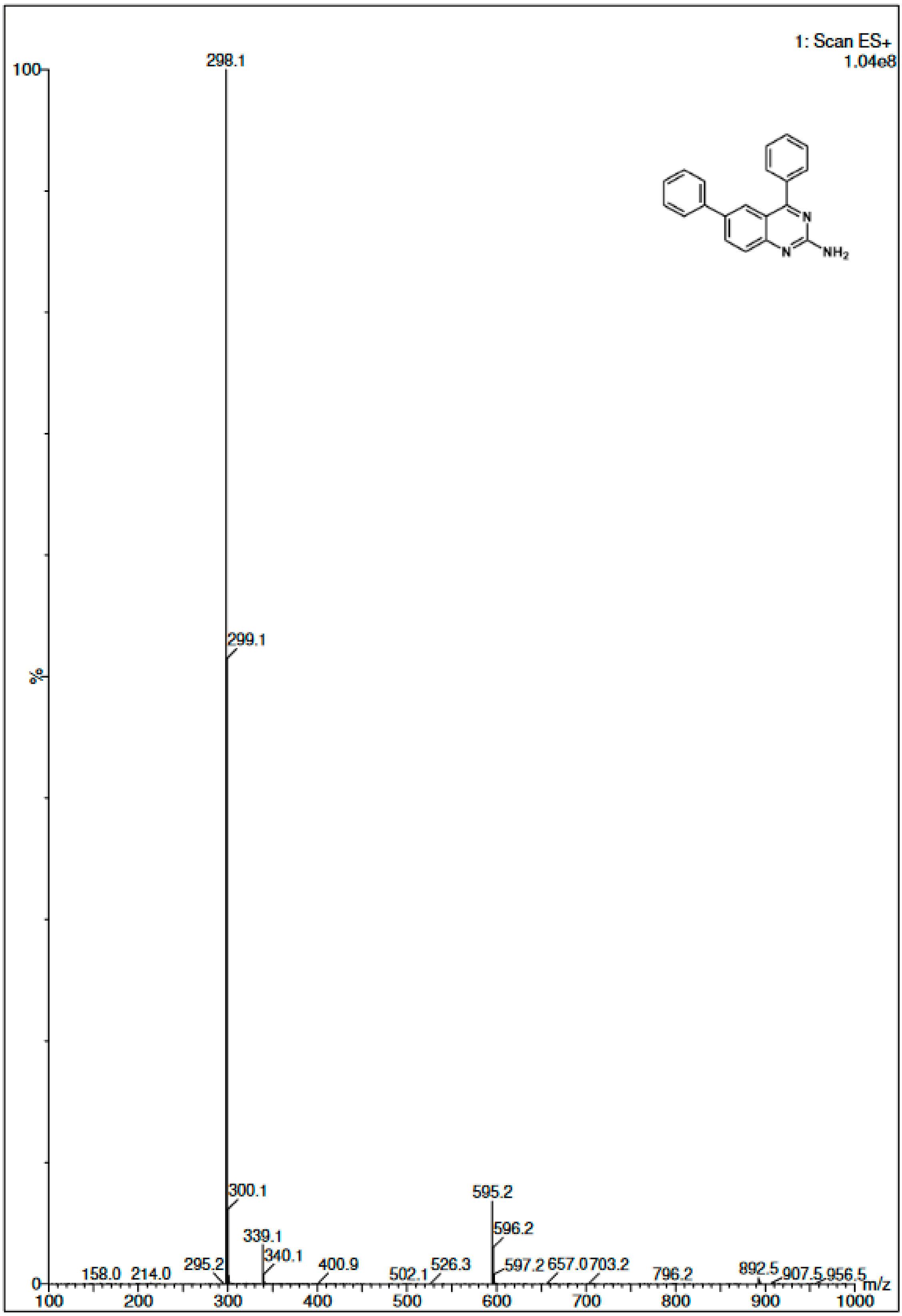

**1H- and 13C-NMR Spectra in DMSO-$d_6$ of compound 5k**

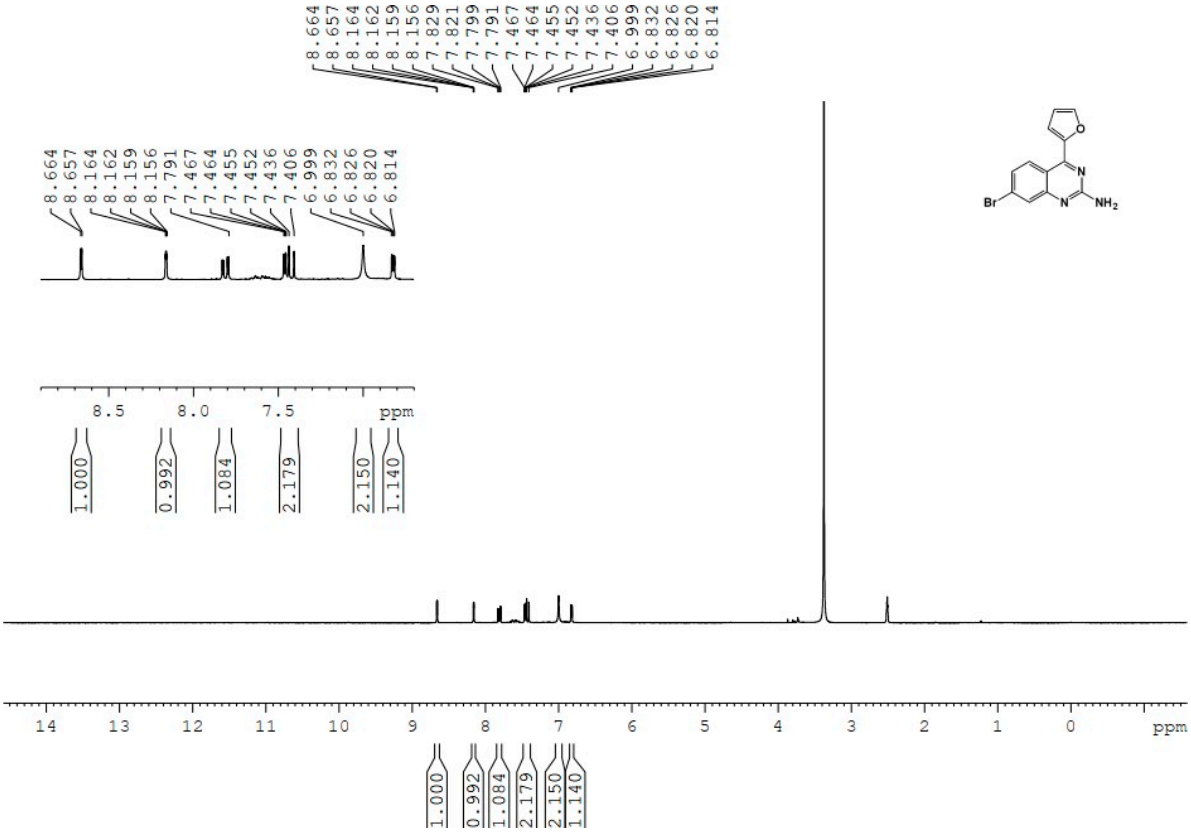


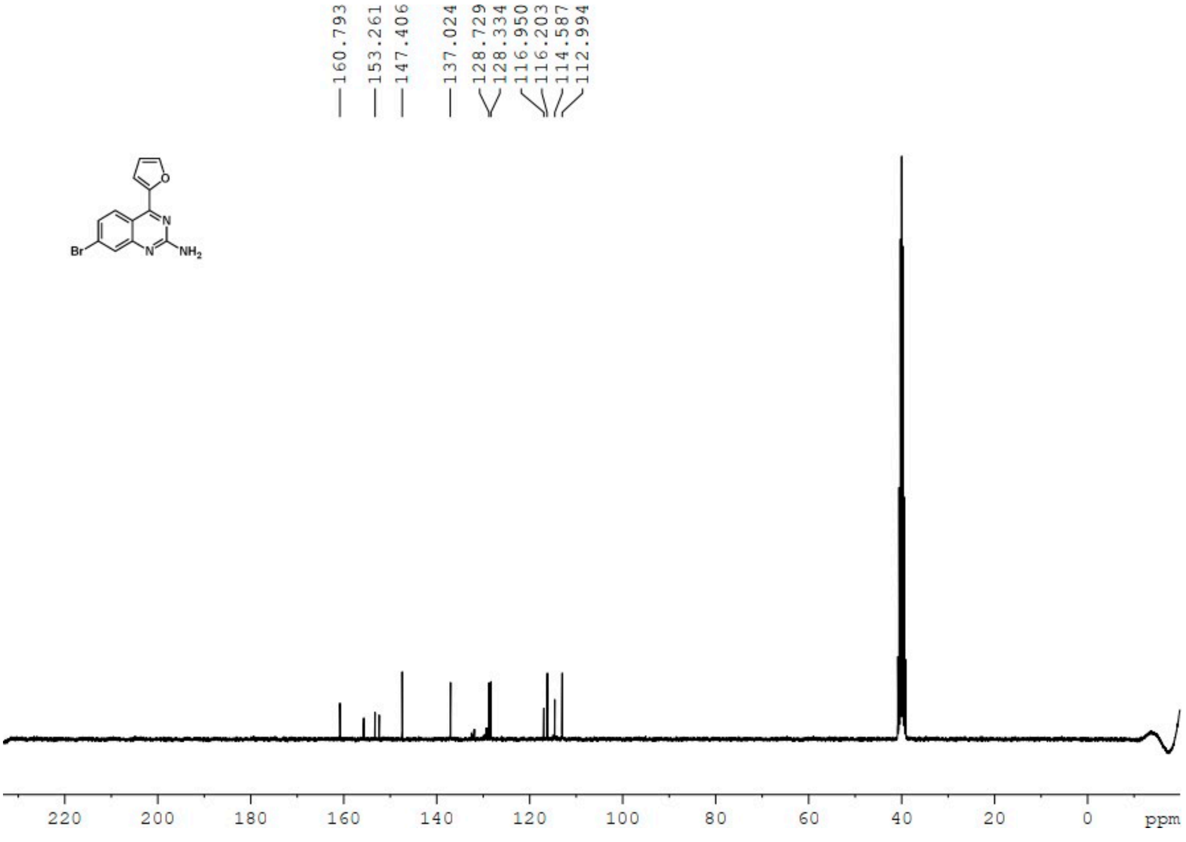

**LC-MS mass spectrum of compound 5k**

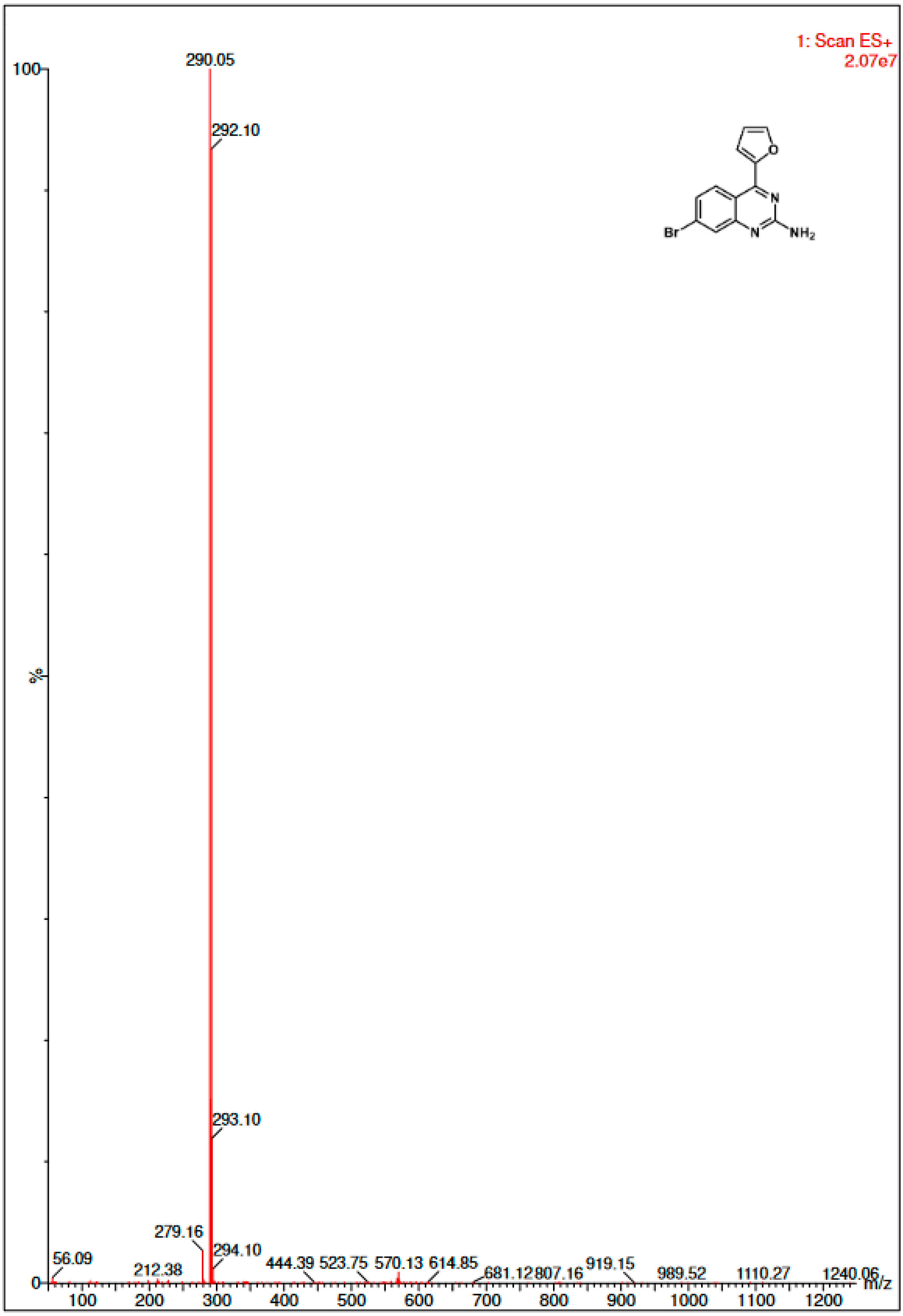

**1H- and 13C-NMR Spectra in $CDCl_3$ of compound 5l**

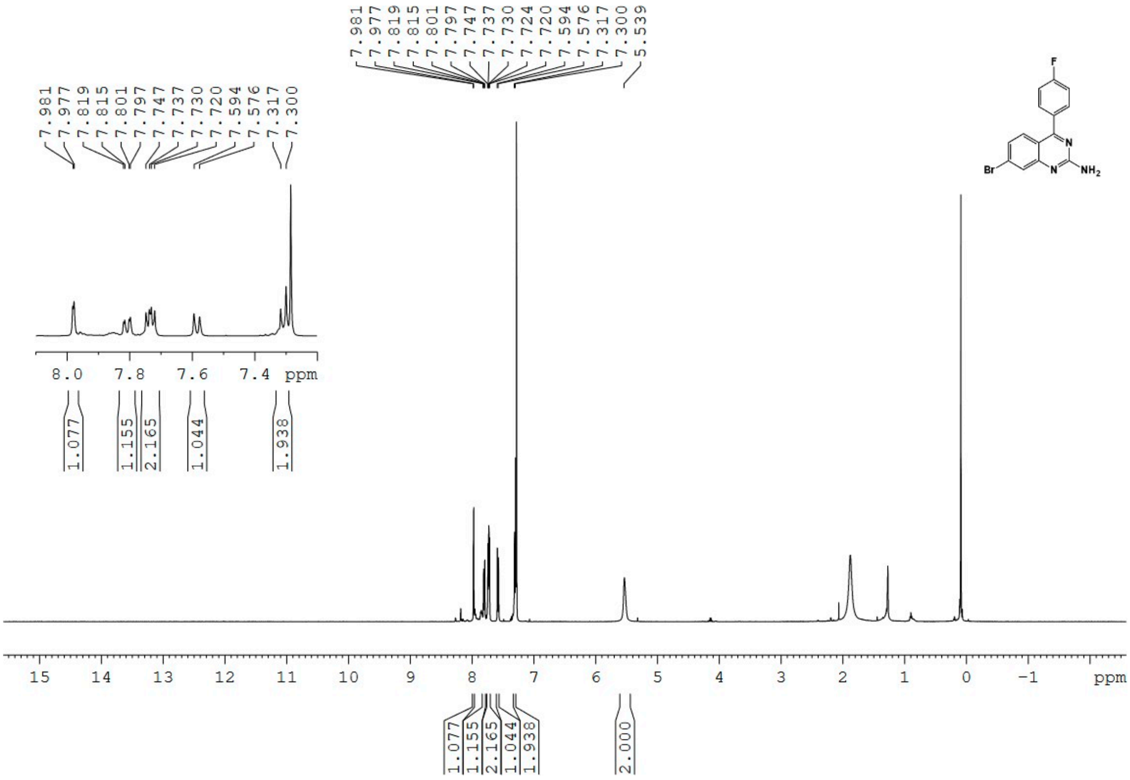


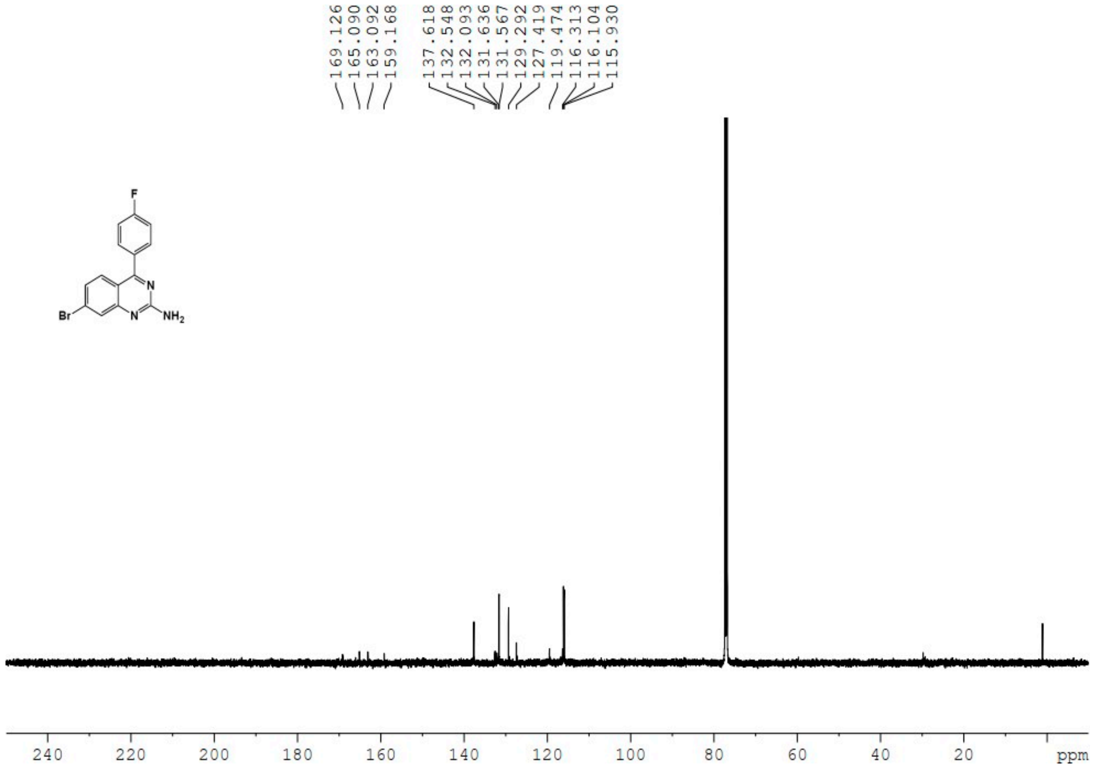

**LC-MS mass spectrum of compound 5l**

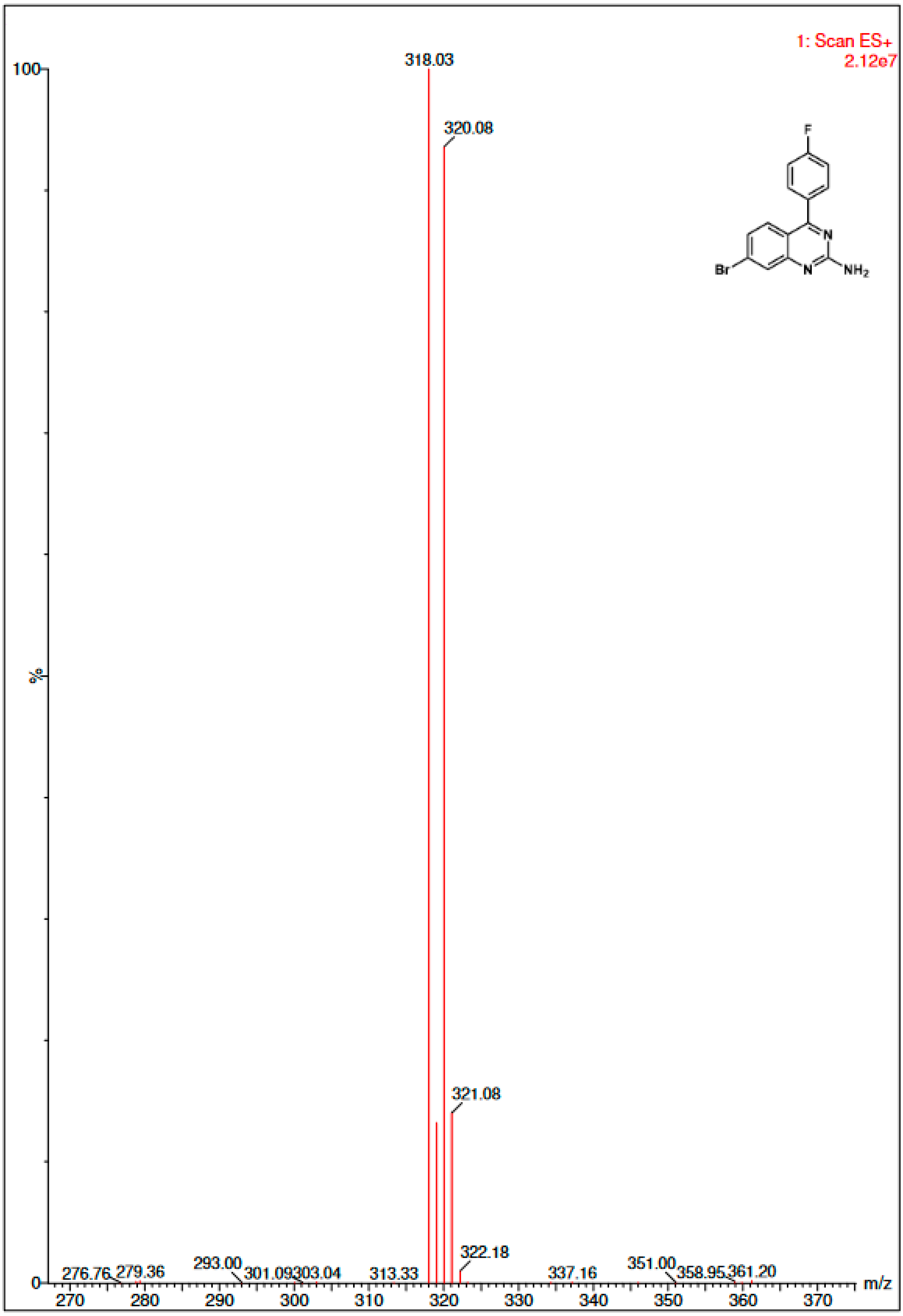

## 1H- and 13C-NMR Spectra in DMSO-d$_6$ of compound 5m

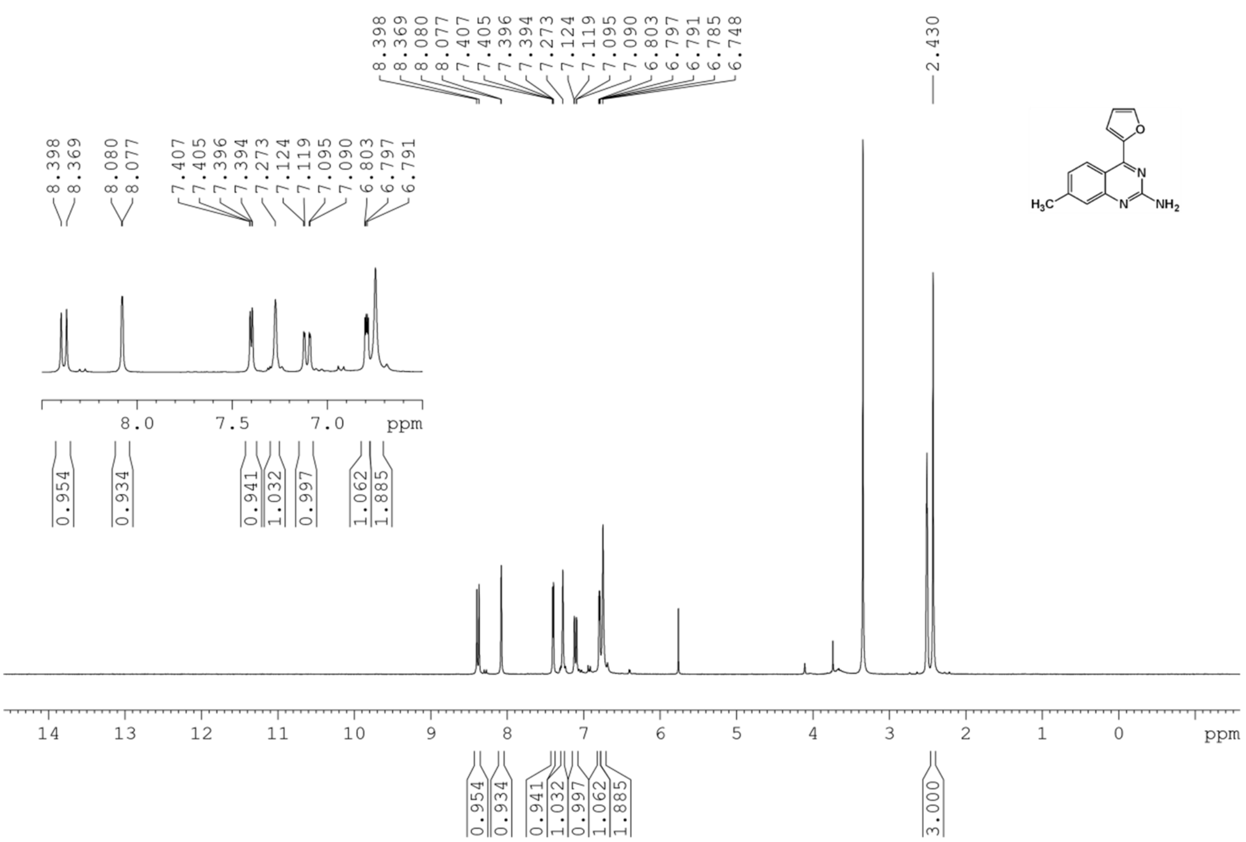


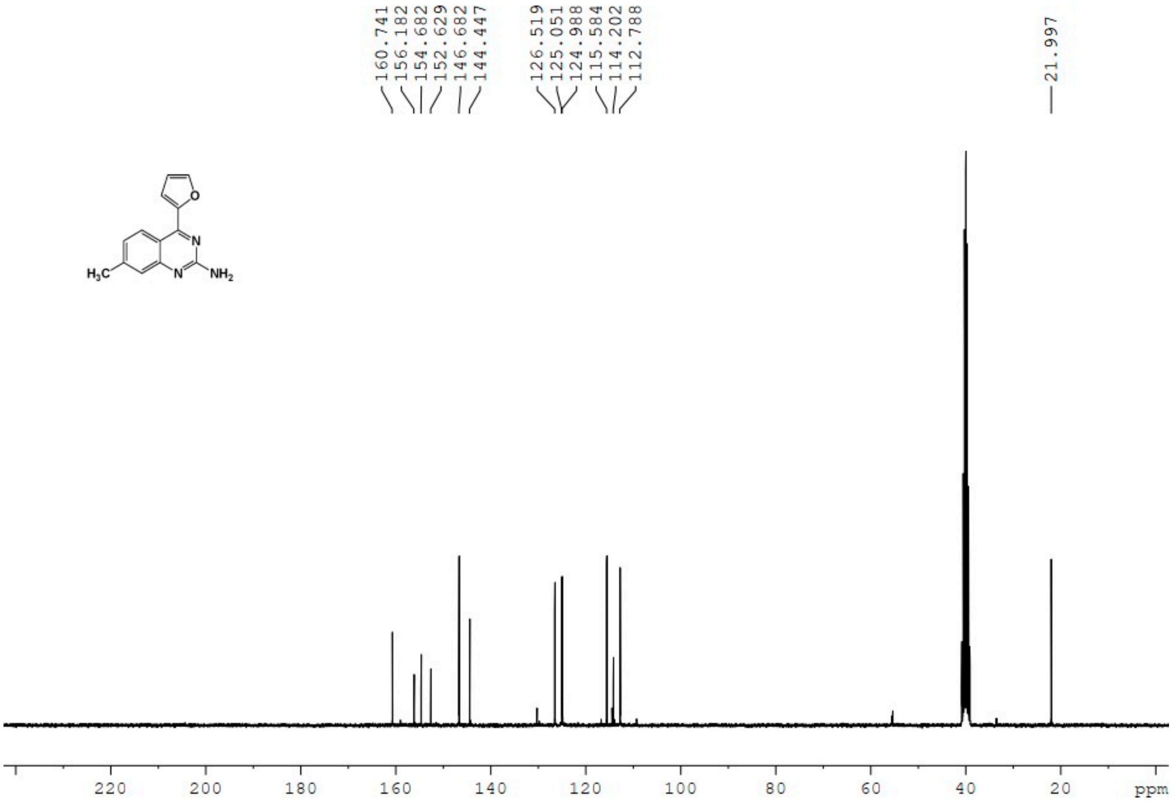

**LC-MS mass spectrum of compound 5m**

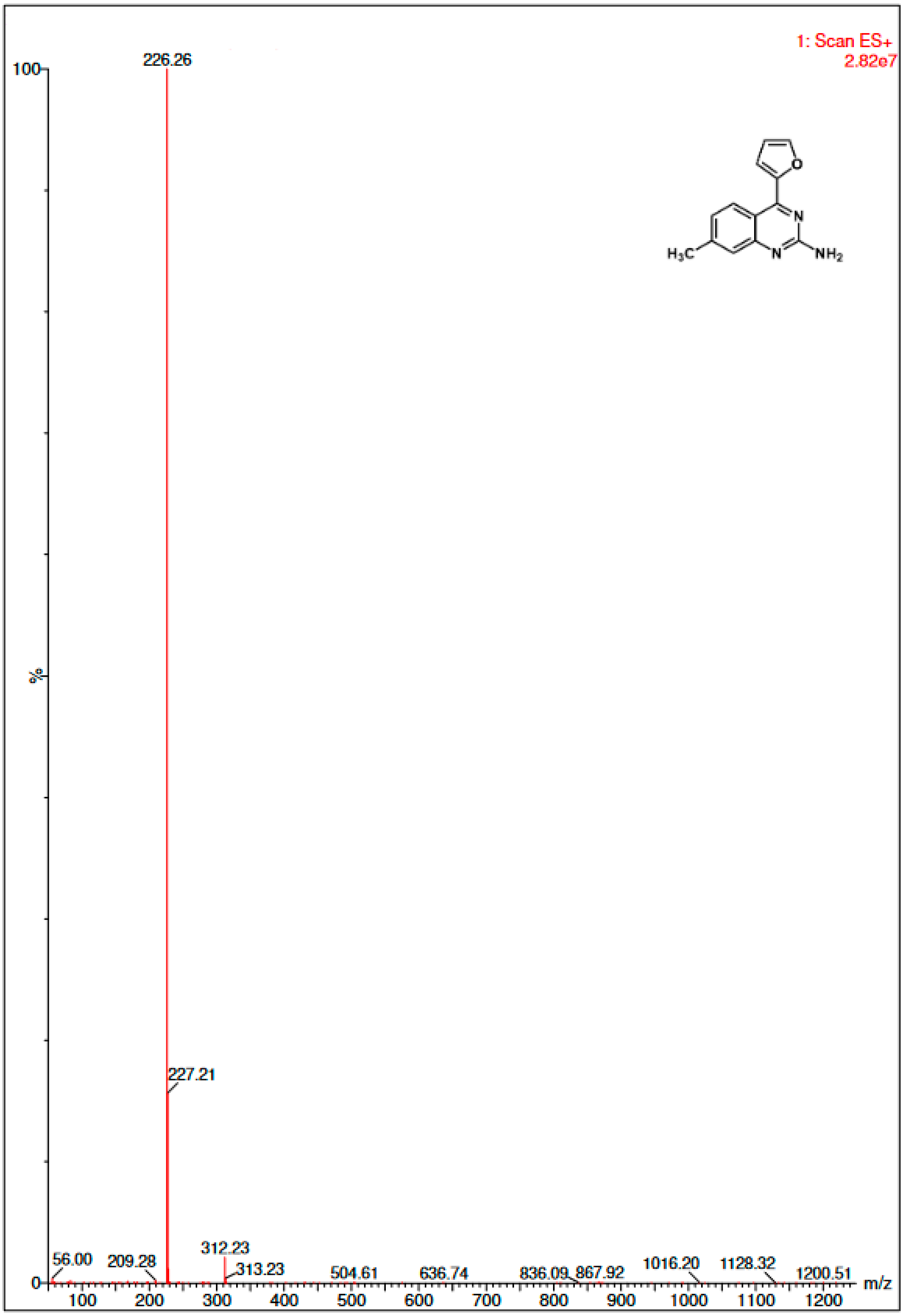

**1H- and 13C-NMR Spectra in $CDCl_3$ of compound 5n**

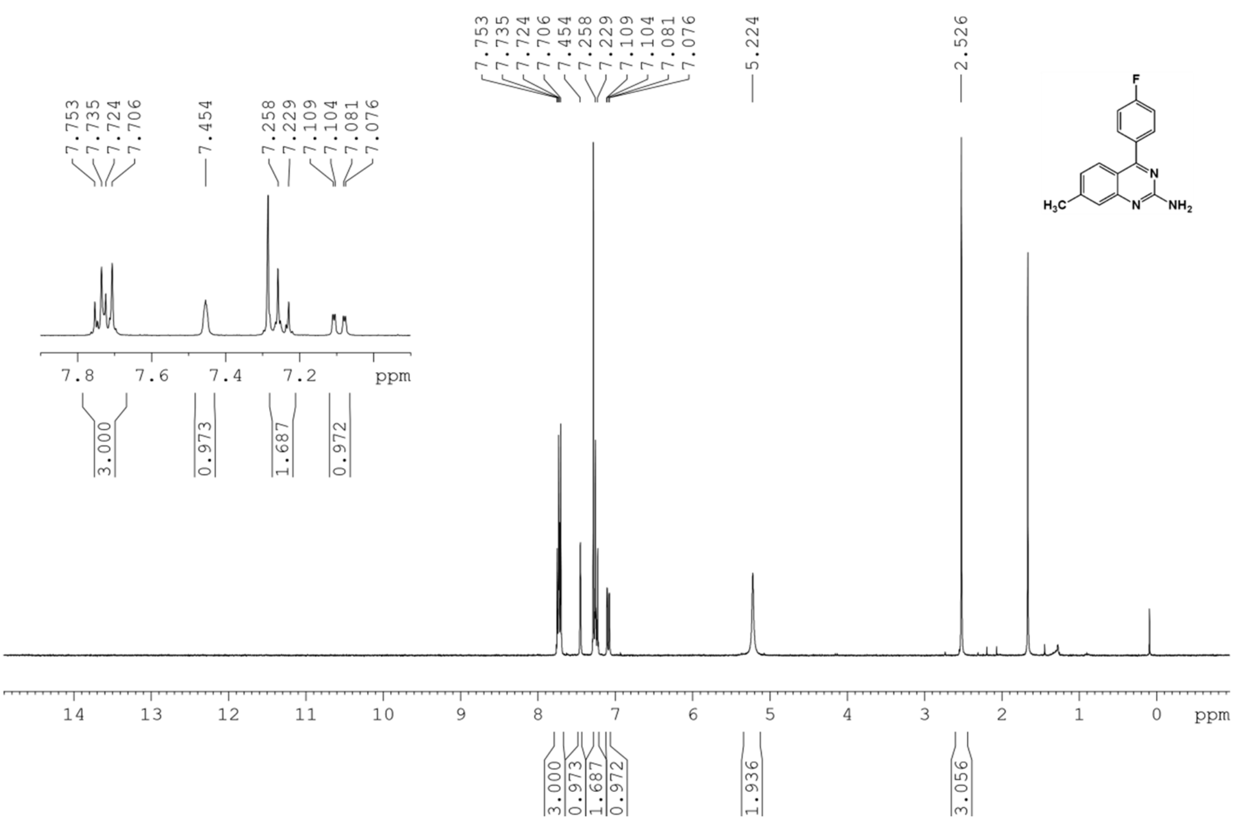


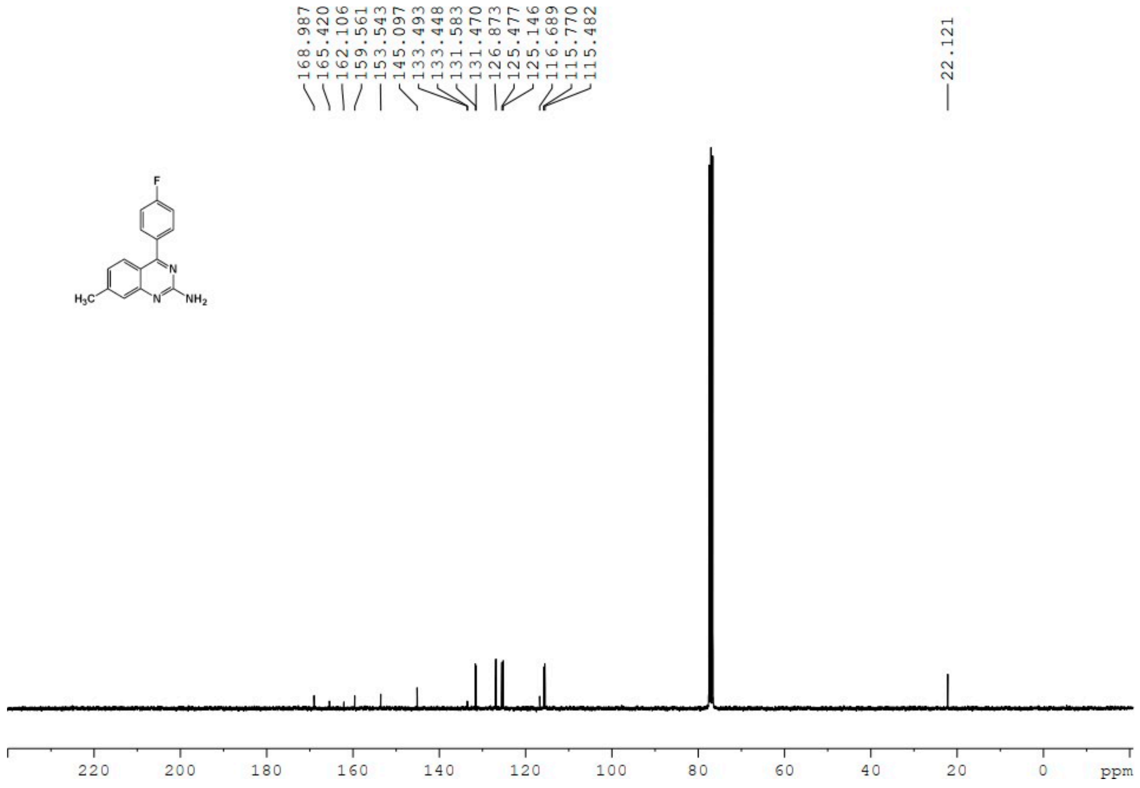

**LC-MS mass spectrum of compound 5n**

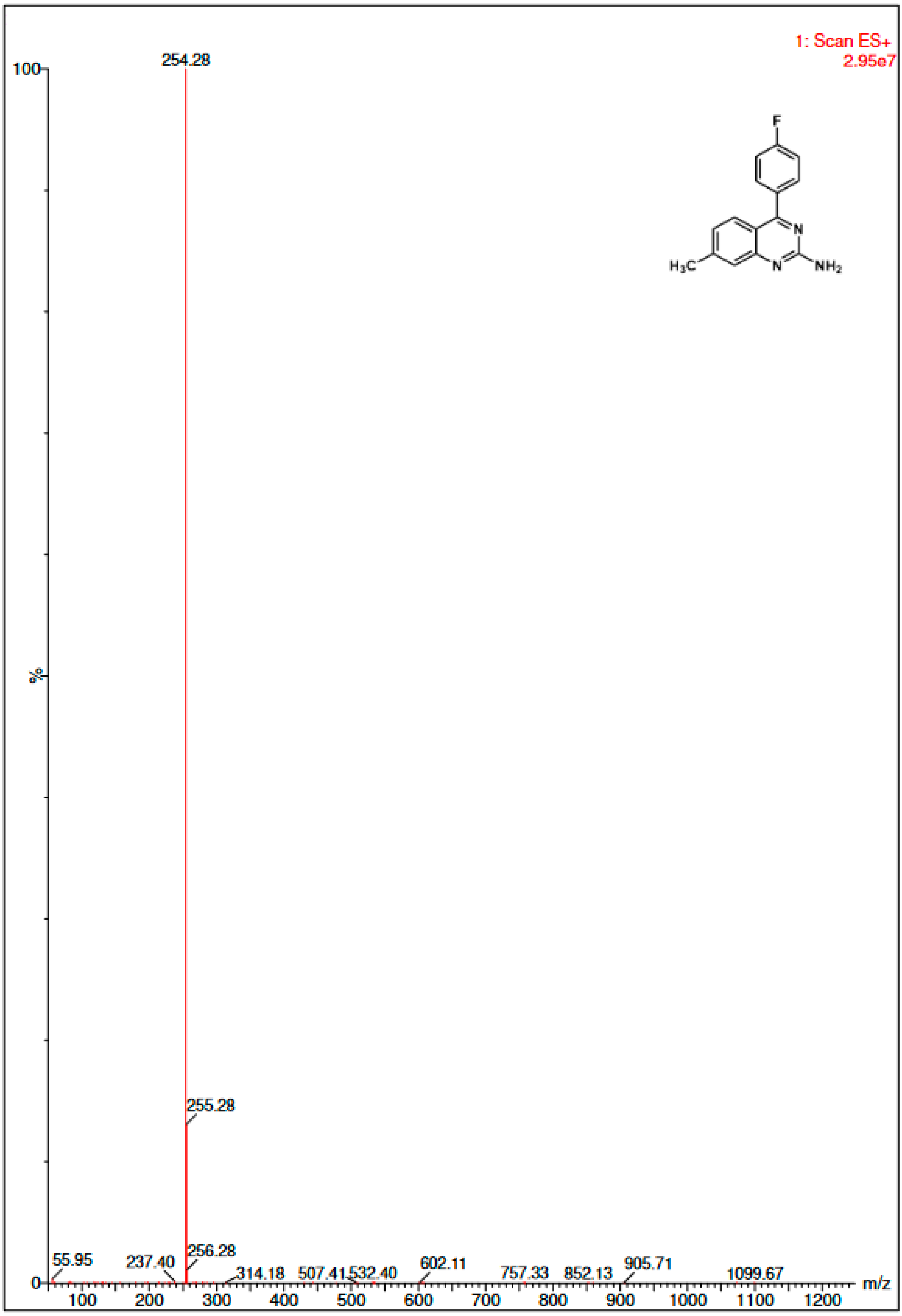

**1H- and 13C-NMR Spectra in $CDCl_3$ of compound 5o**

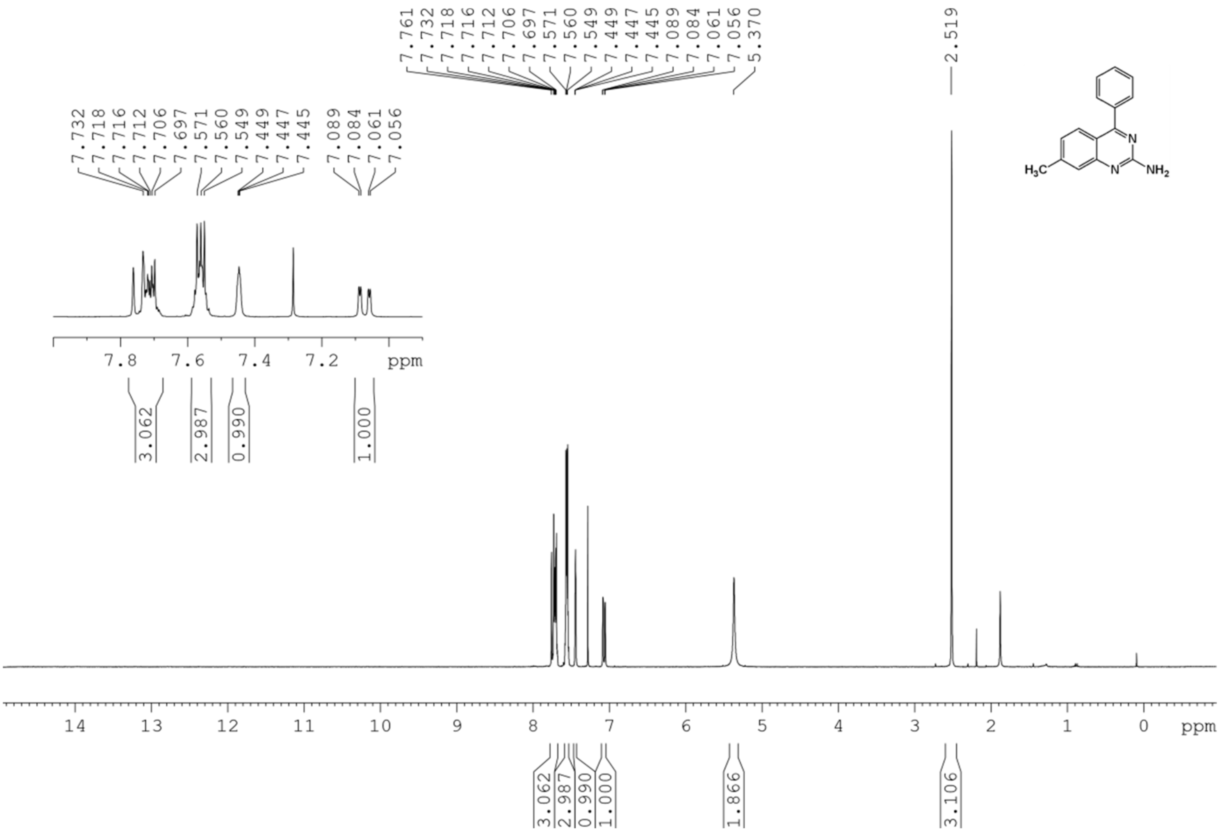


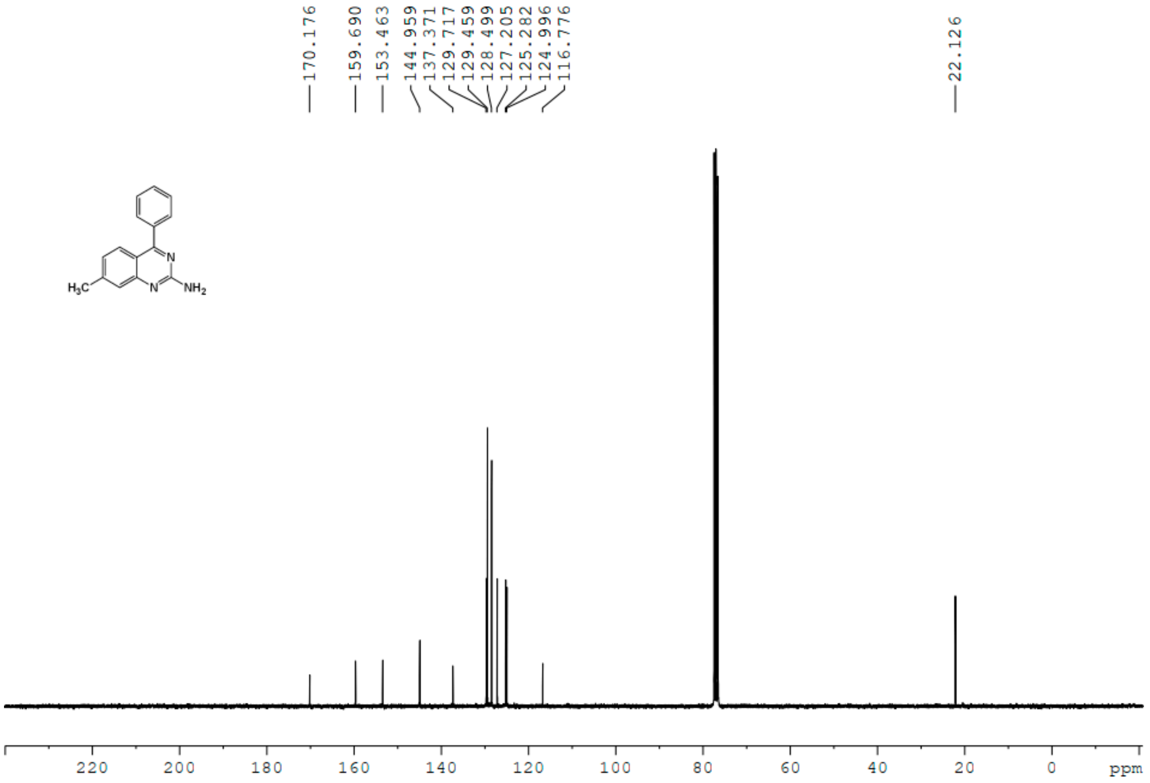

**LC-MS mass spectrum of compound 5o**

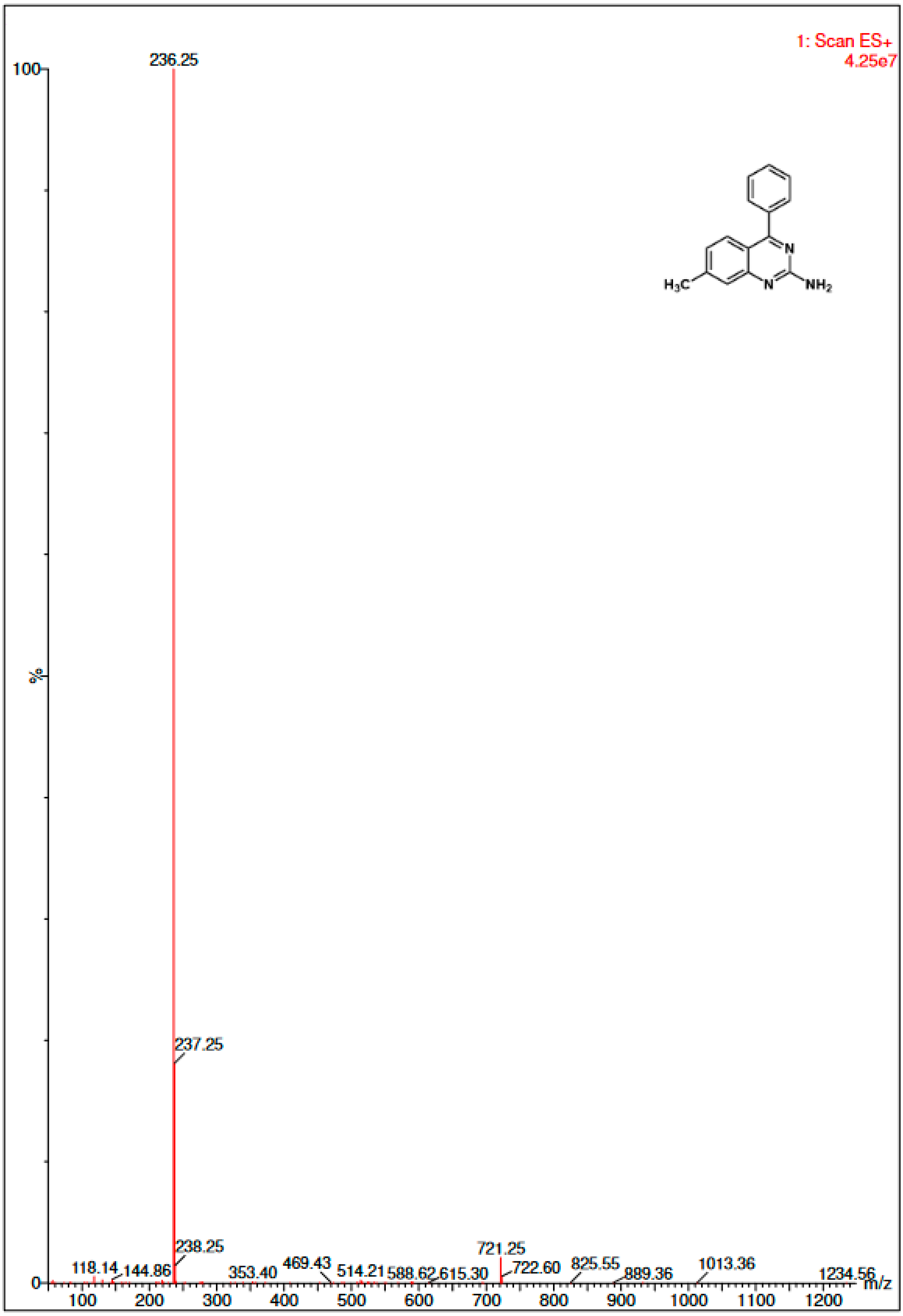

**1H- and 13C-NMR Spectra in DMSO-$d_6$ of compound 5p**

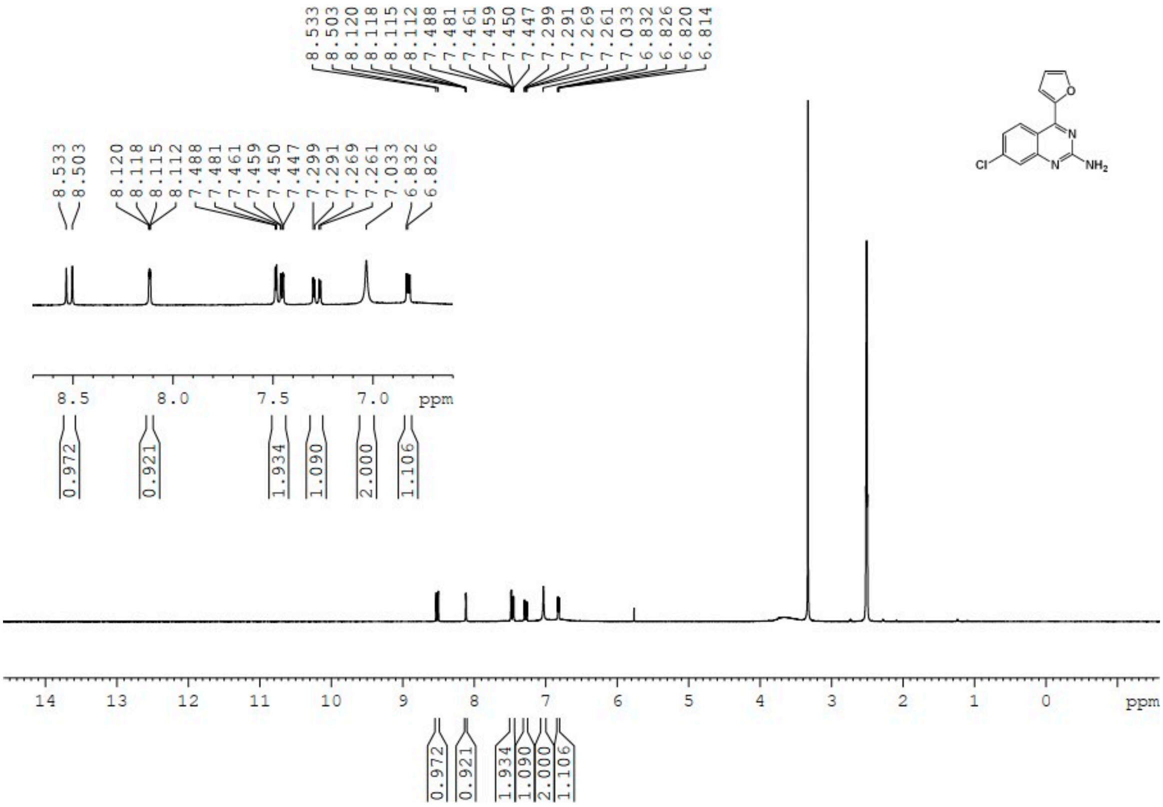

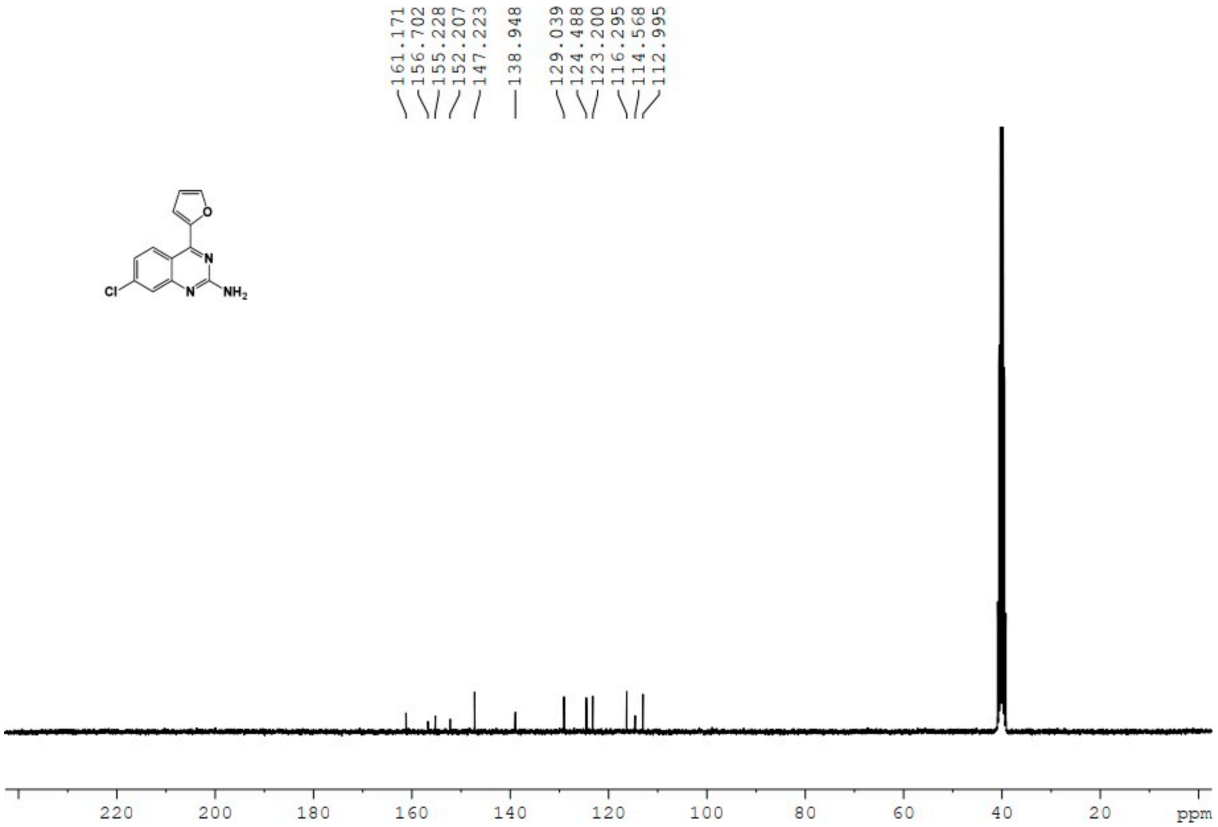

LC-MS mass spectrum of compound 5p

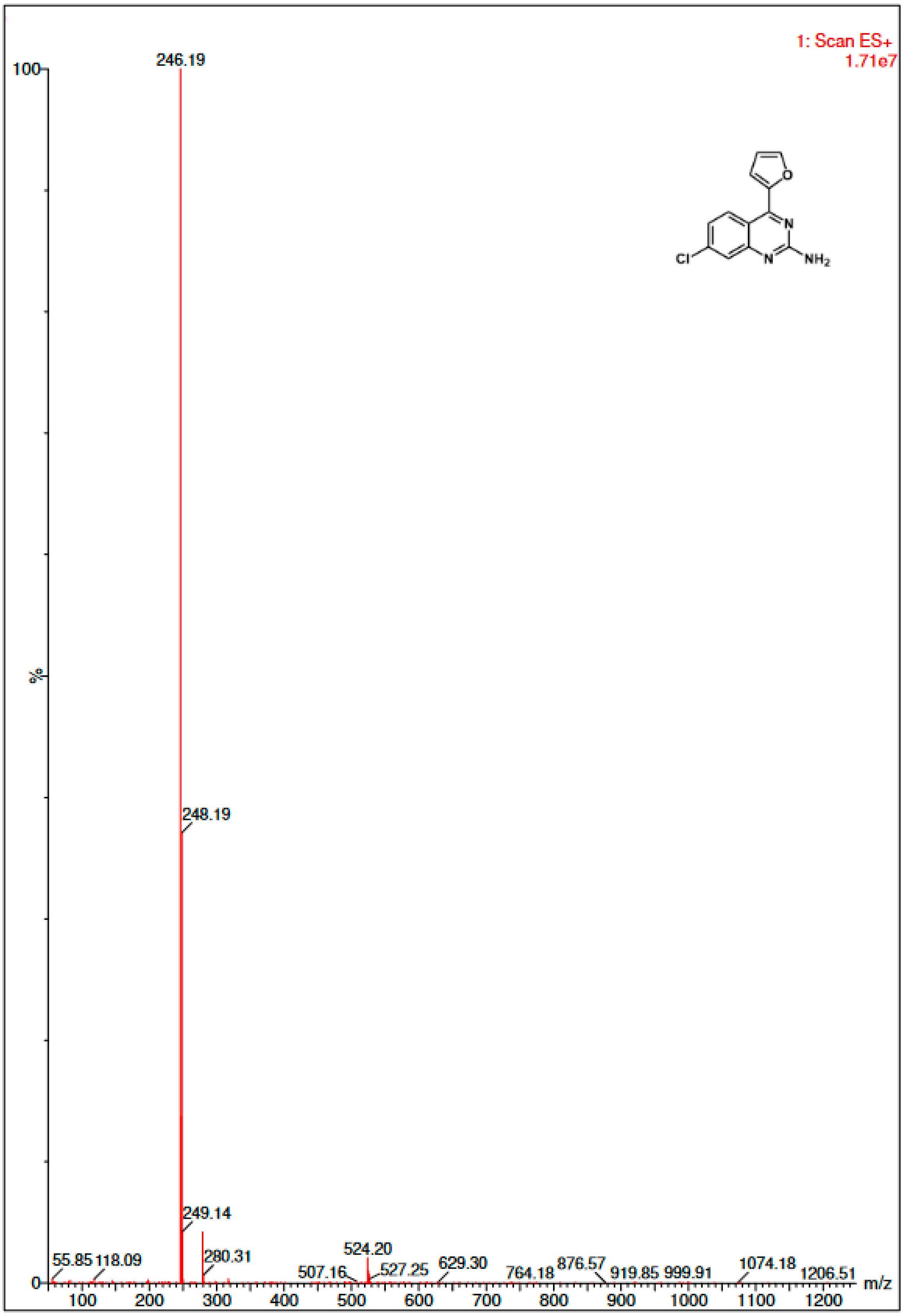

**1H- and 13C-NMR Spectra in $CDCl_3$ of compound 9a**

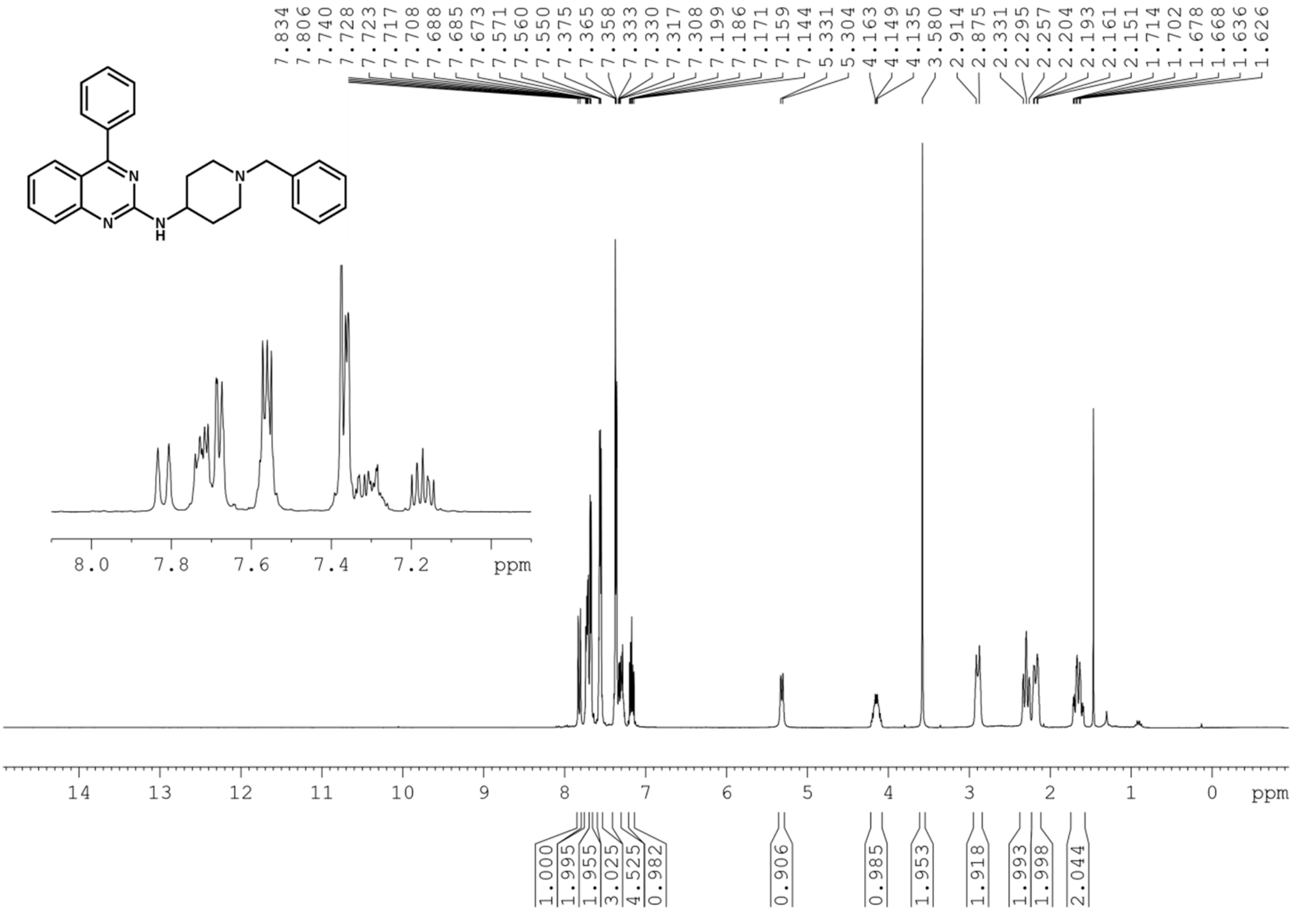


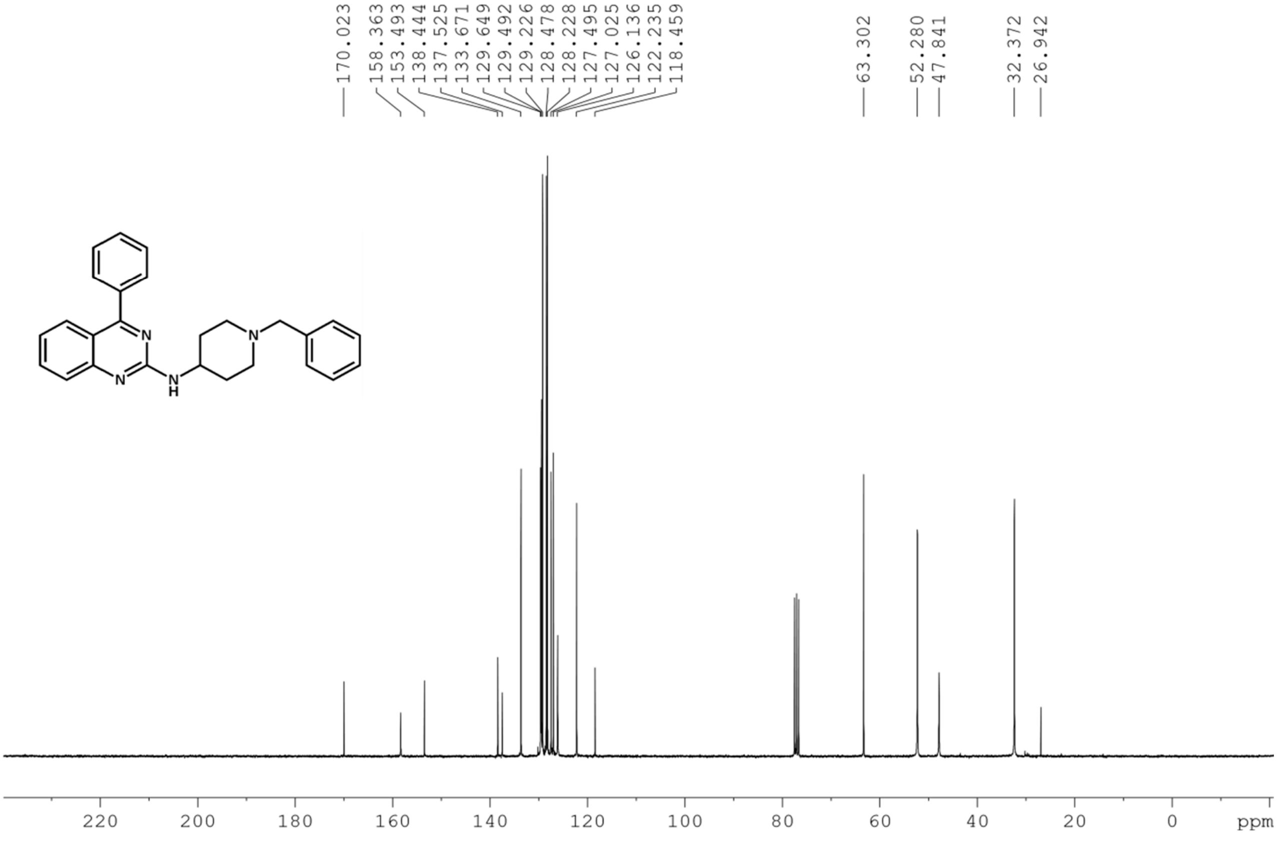

**LC-MS mass spectrum of compound 9a**

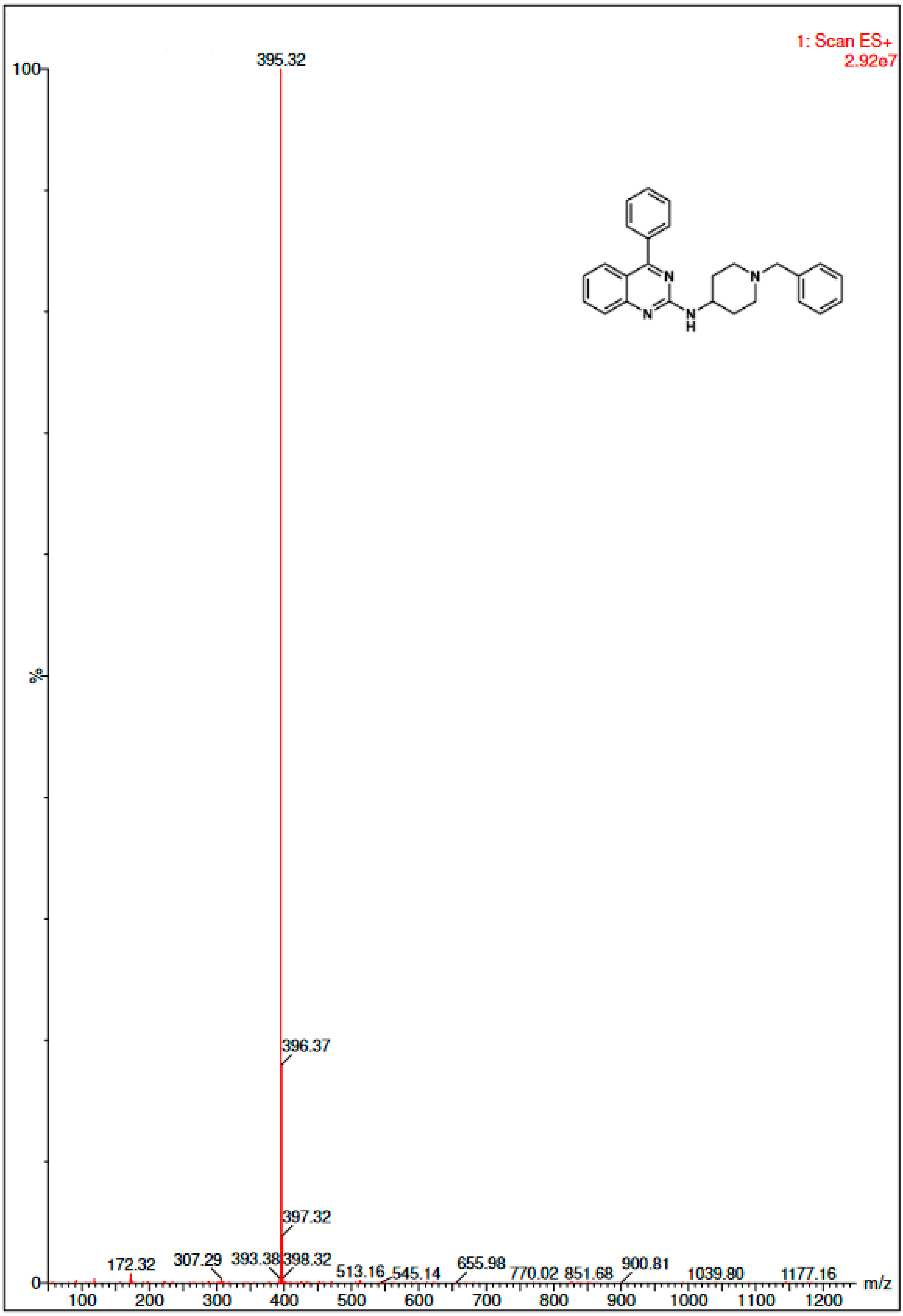

**1H- and 13C-NMR Spectra in $CDCl_3$ of compound 9b**

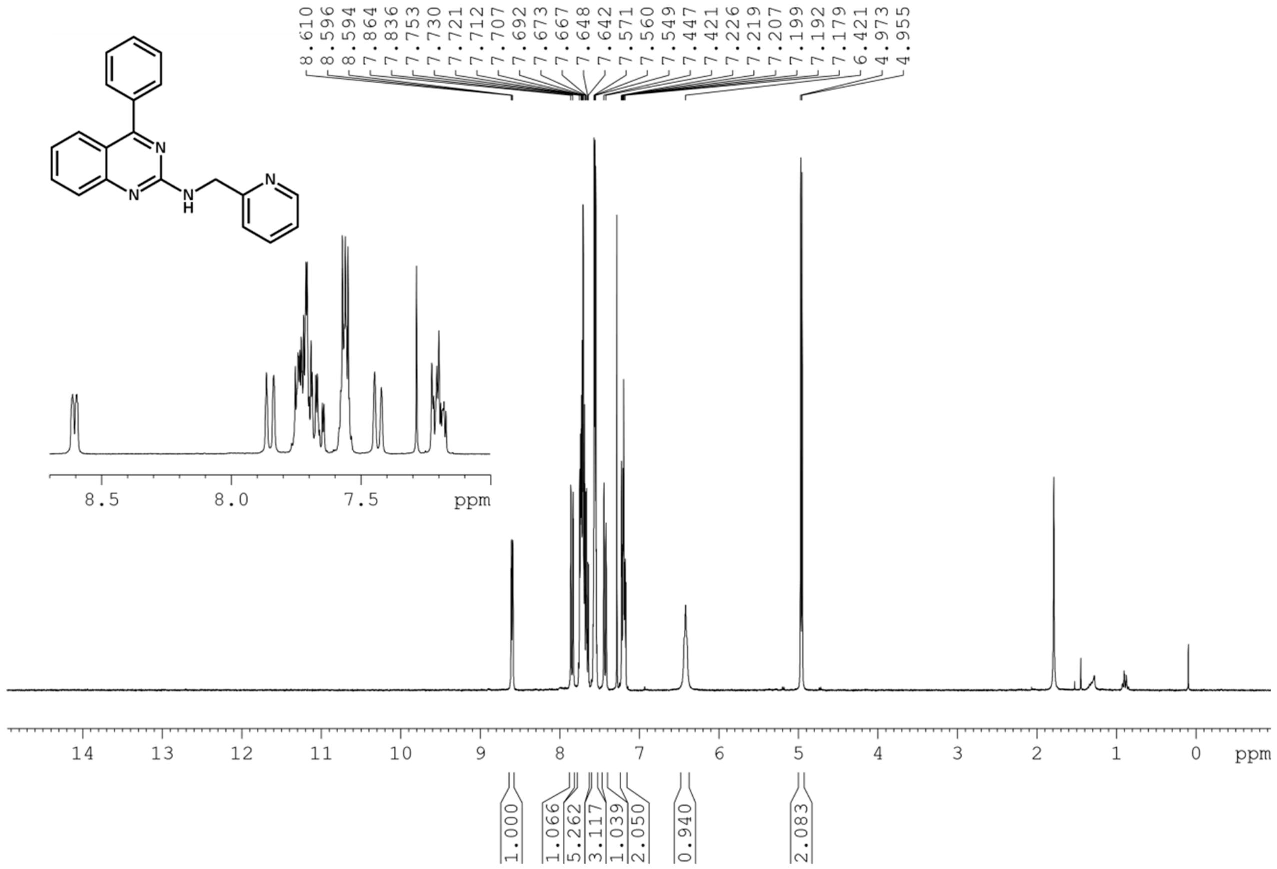


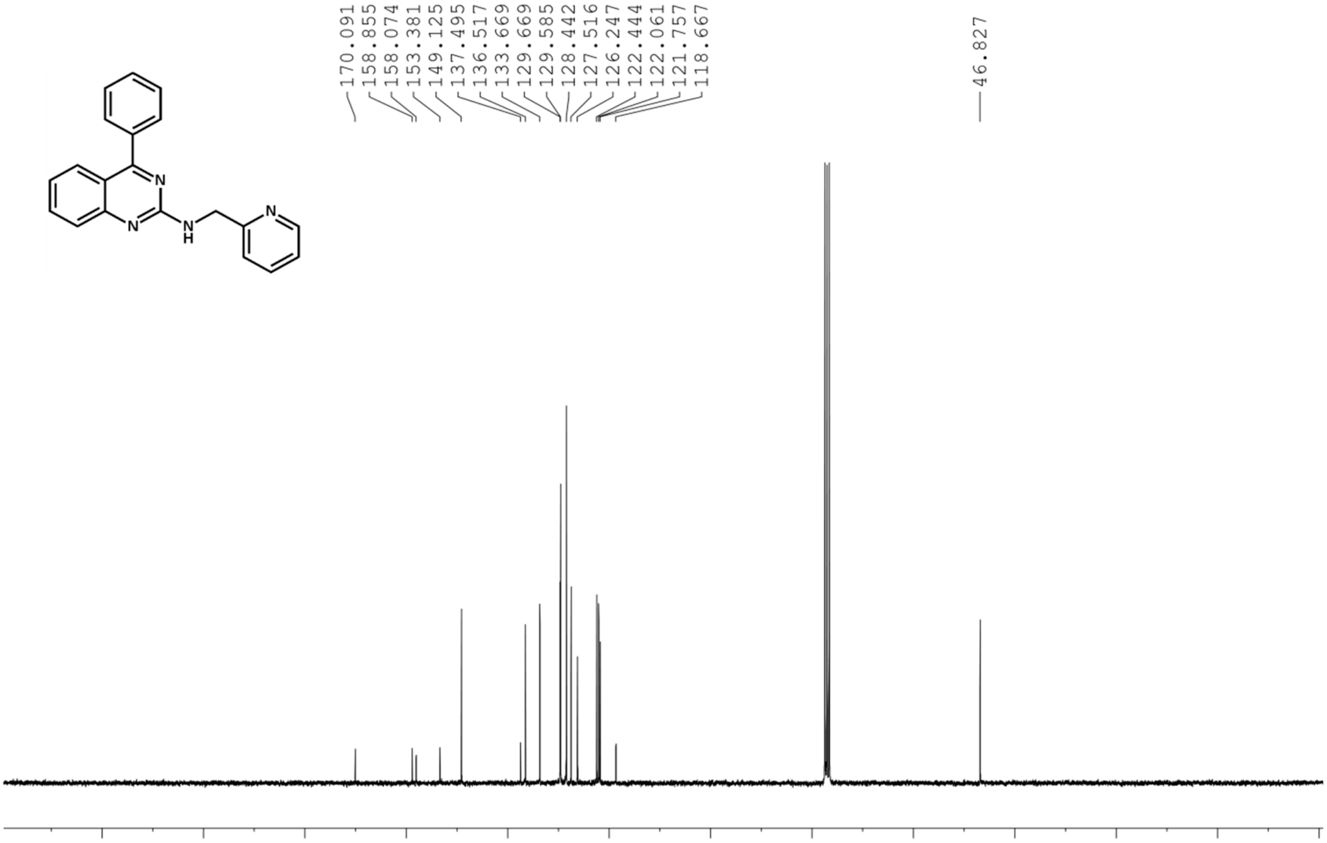

**LC-MS mass spectrum of compound 9b**

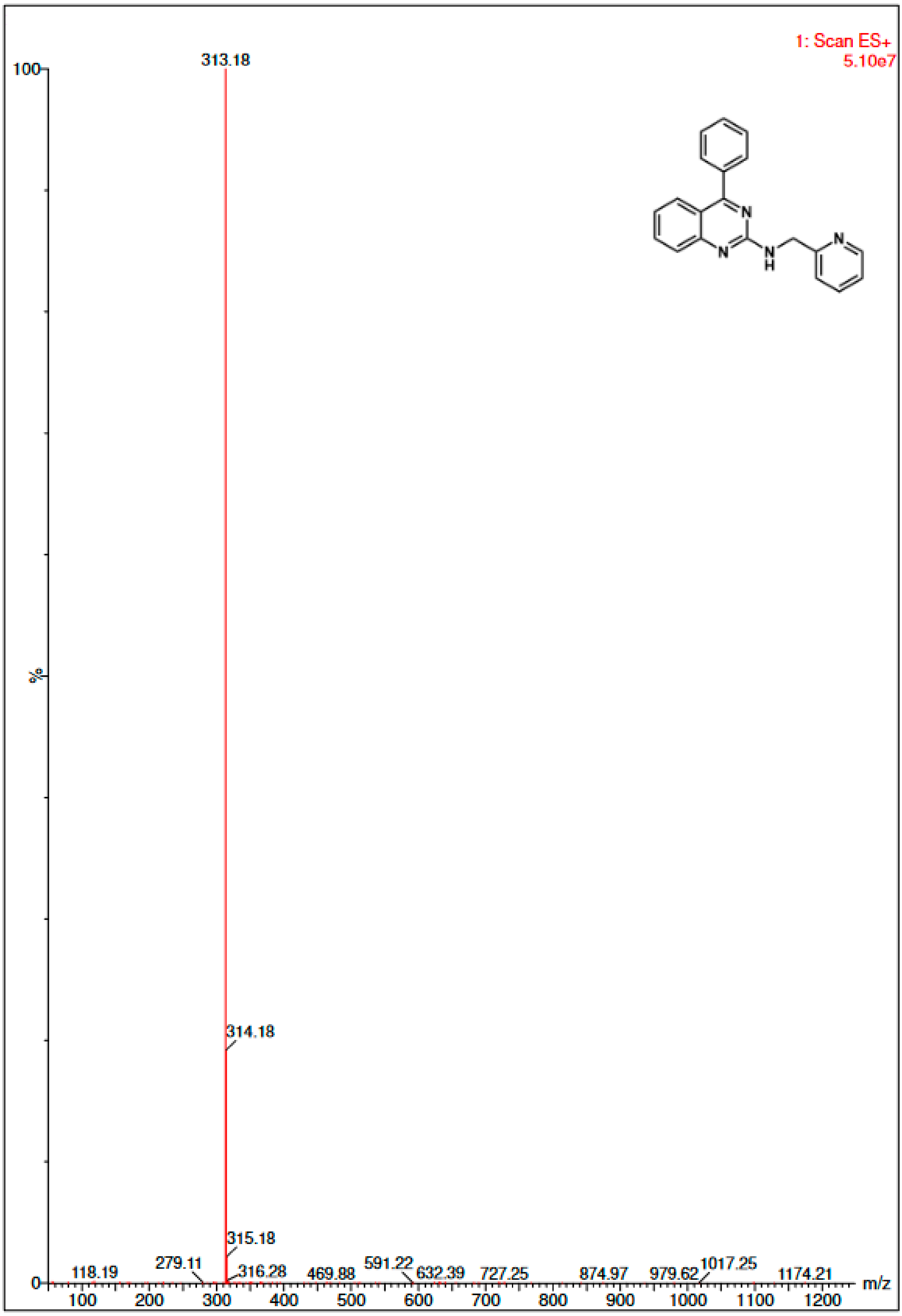

**1H- and 13C-NMR Spectra in $CDCl_3$ of compound 9c**

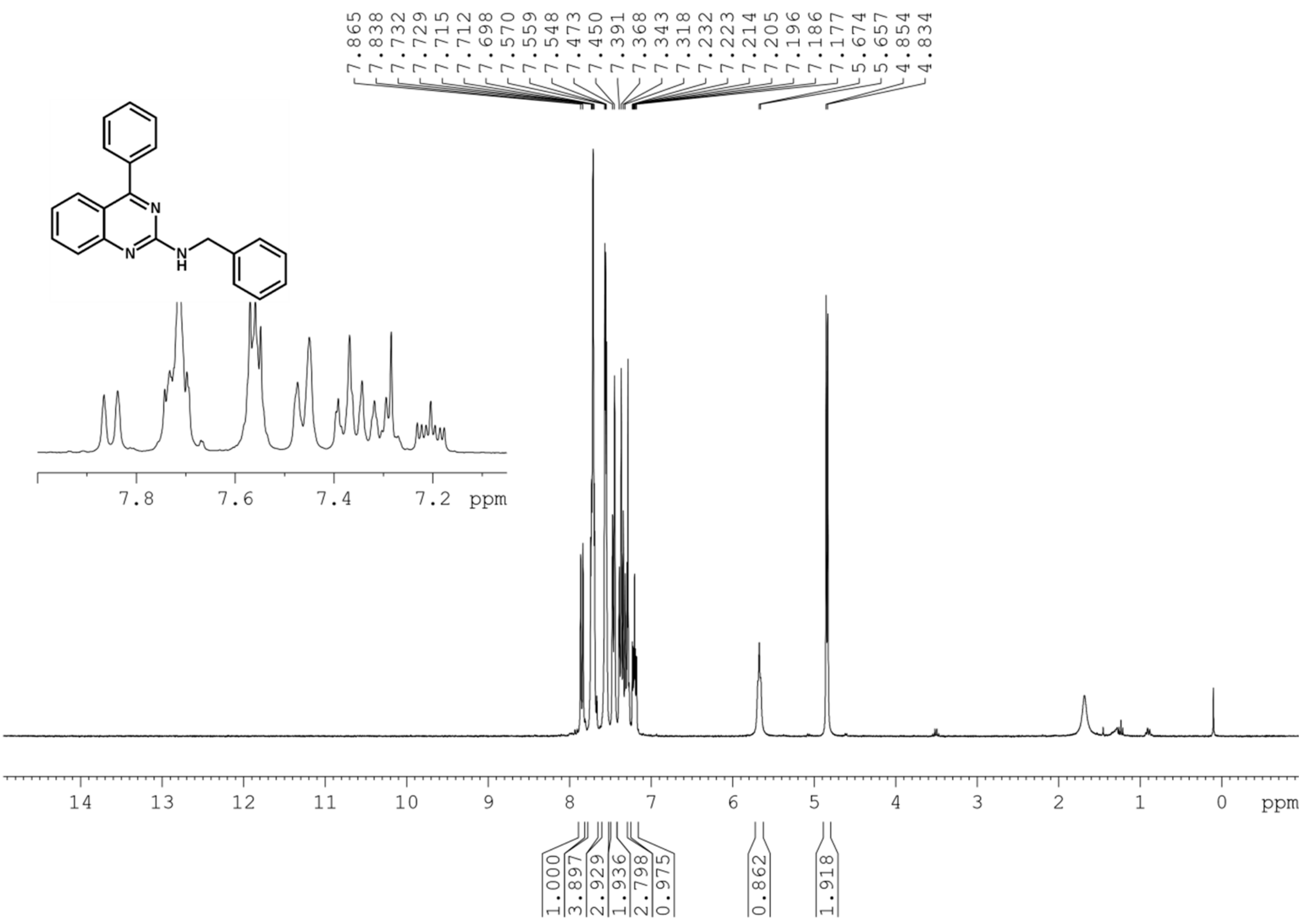


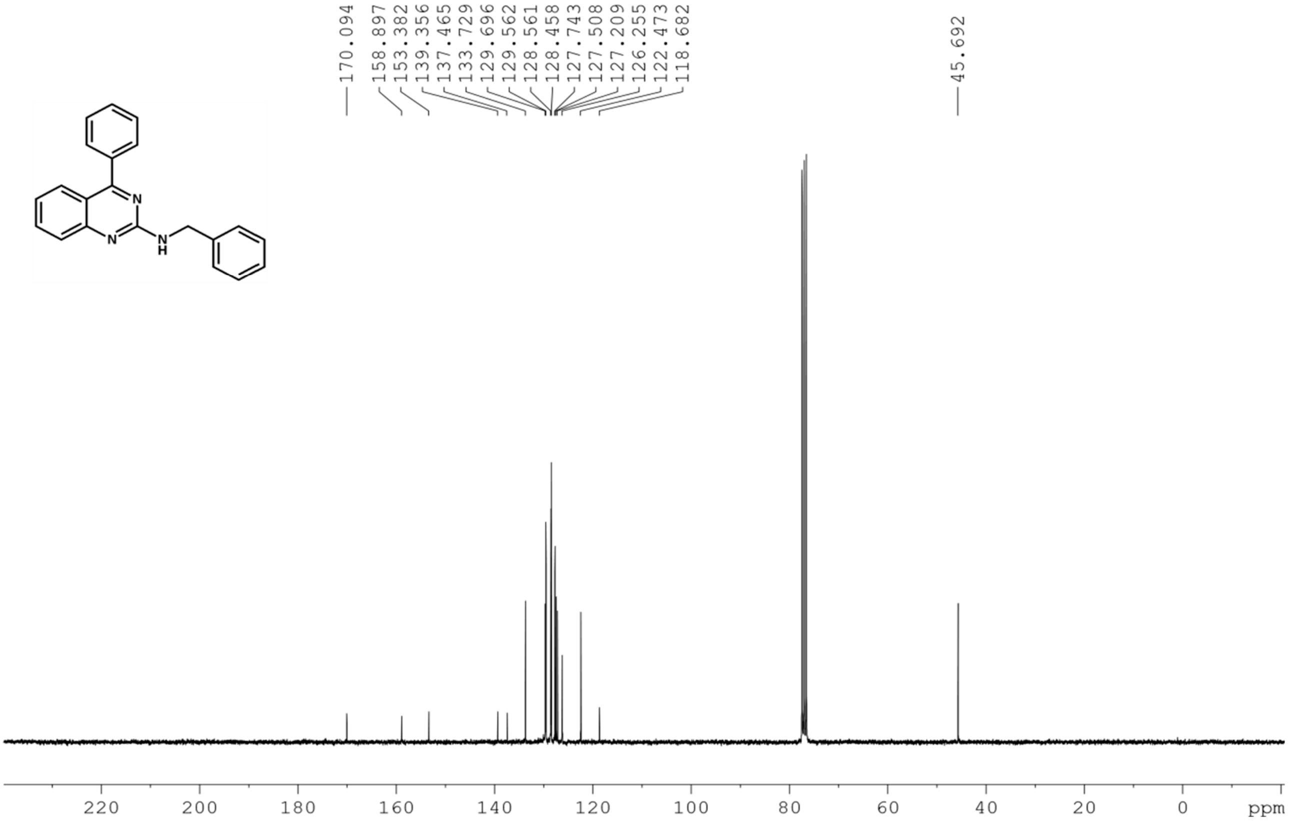

**LC-MS mass spectrum of compound 9c**

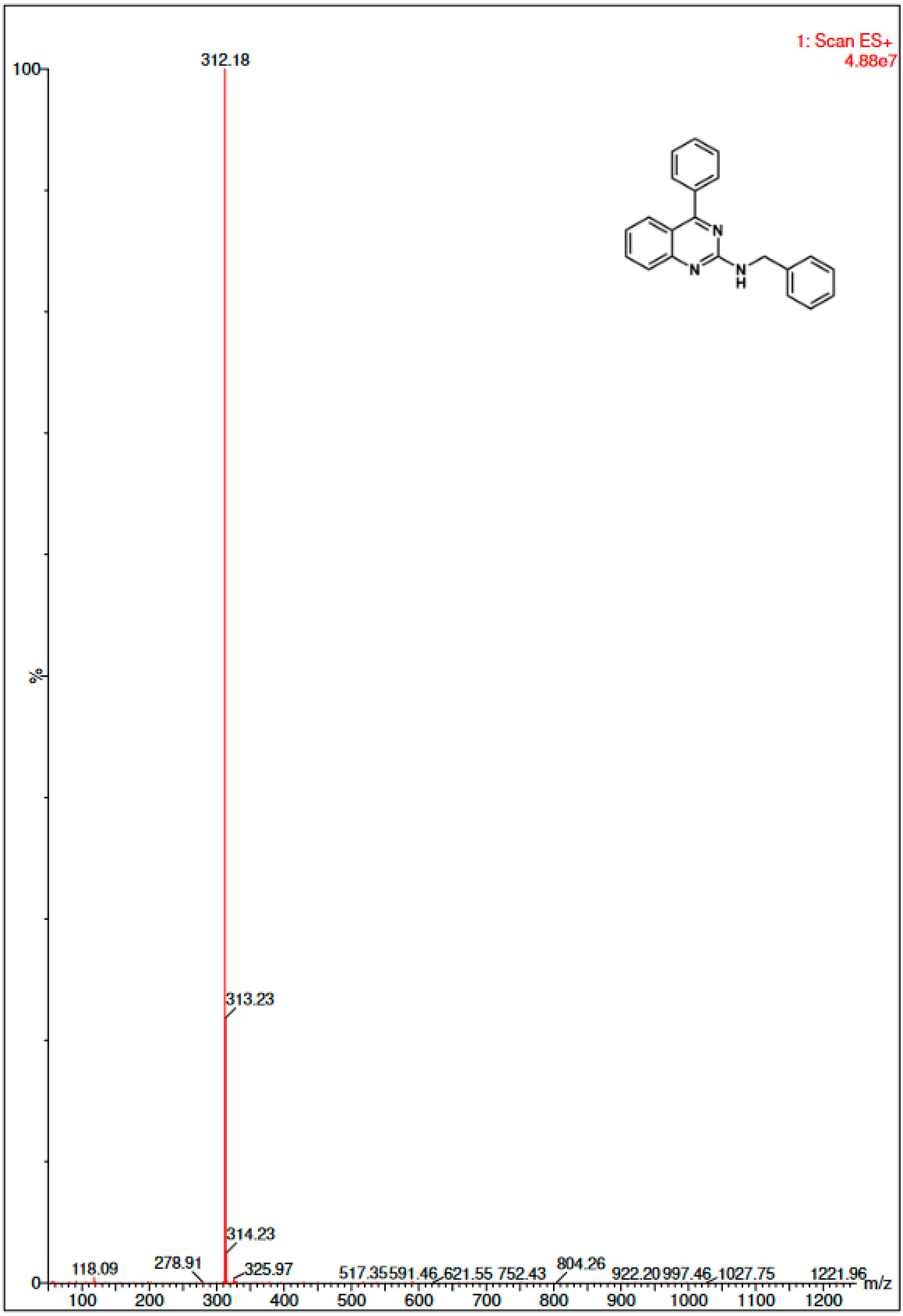

**1H- and 13C-NMR Spectra in $CDCl_3$ of compound 9d**

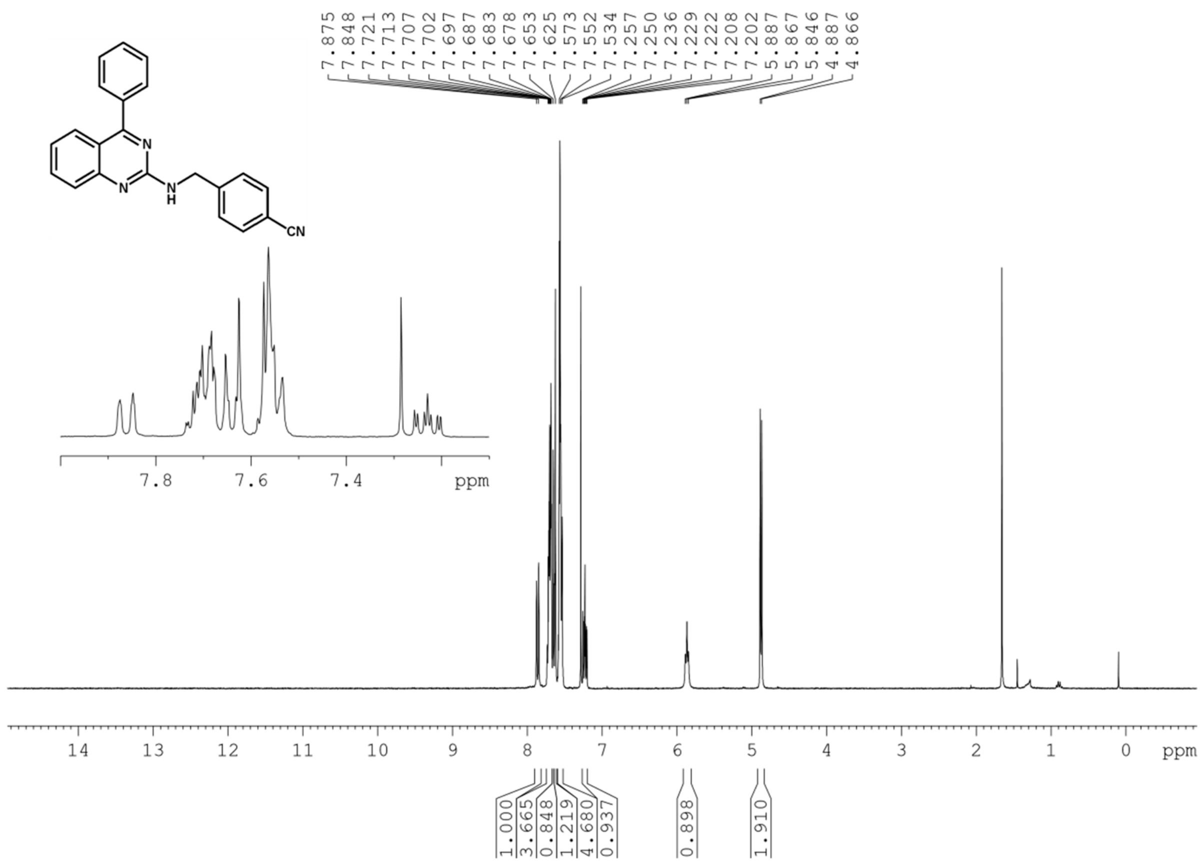


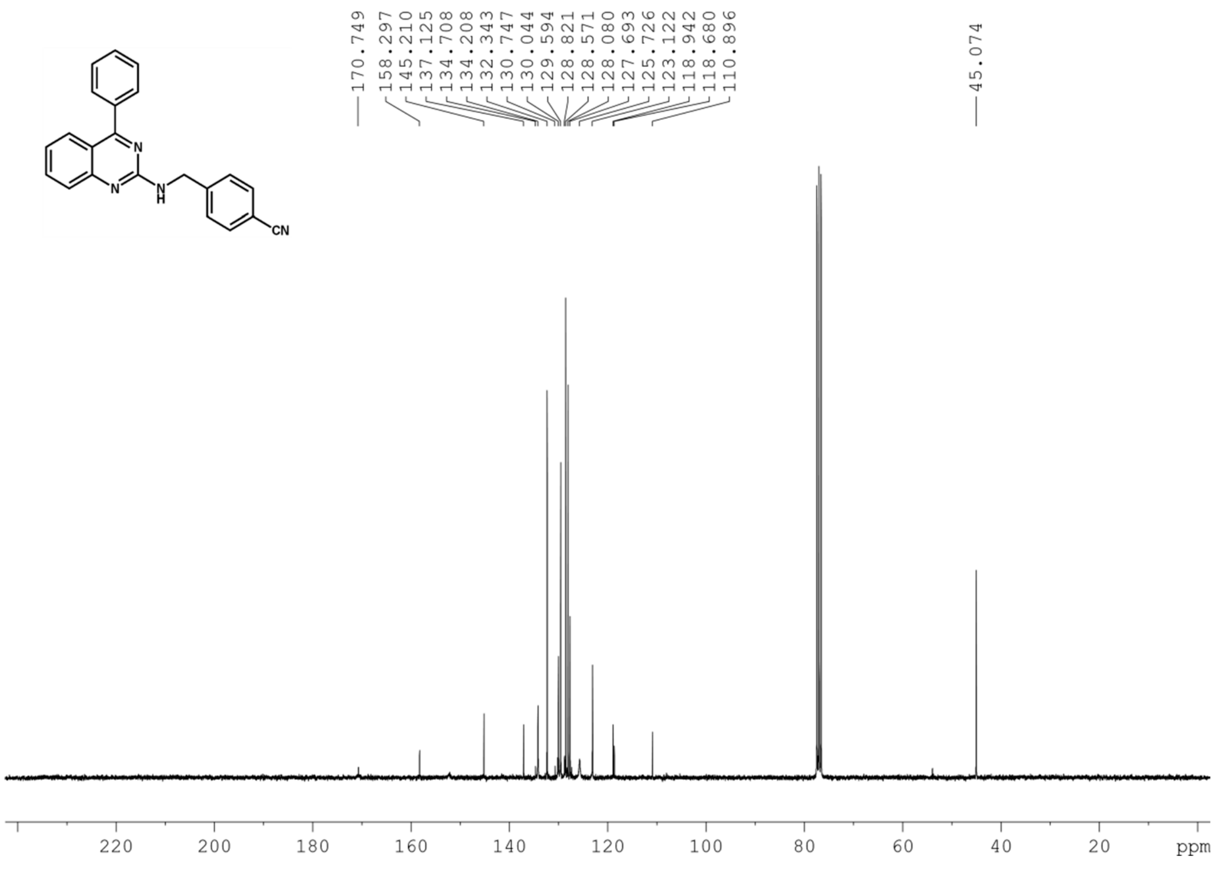

**LC-MS mass spectrum of compound 9d**

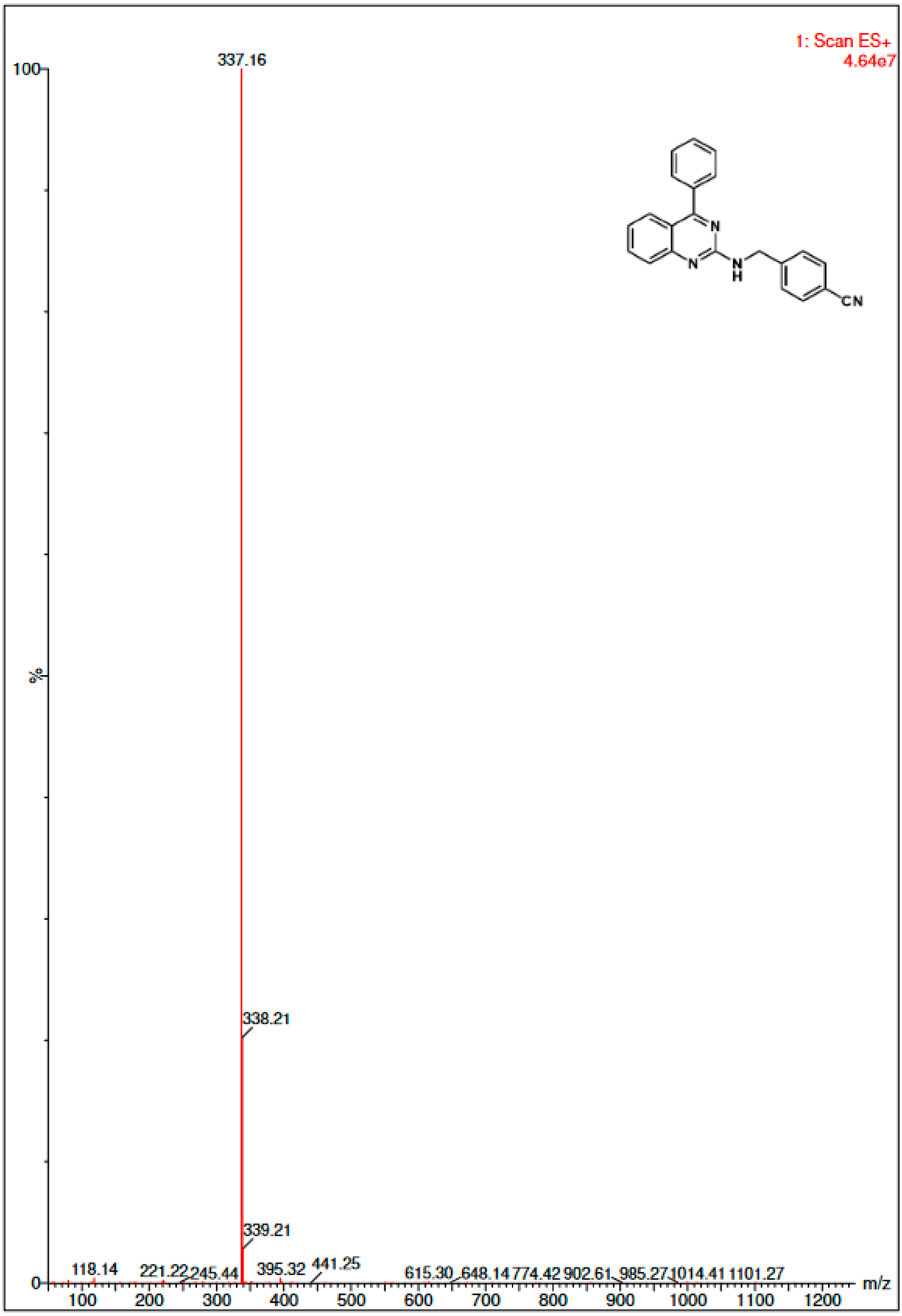

**1H-NMR Spectra in DMSO-d6 and 13C-NMR Spectra in $CDCl_3$ of compound 9e**

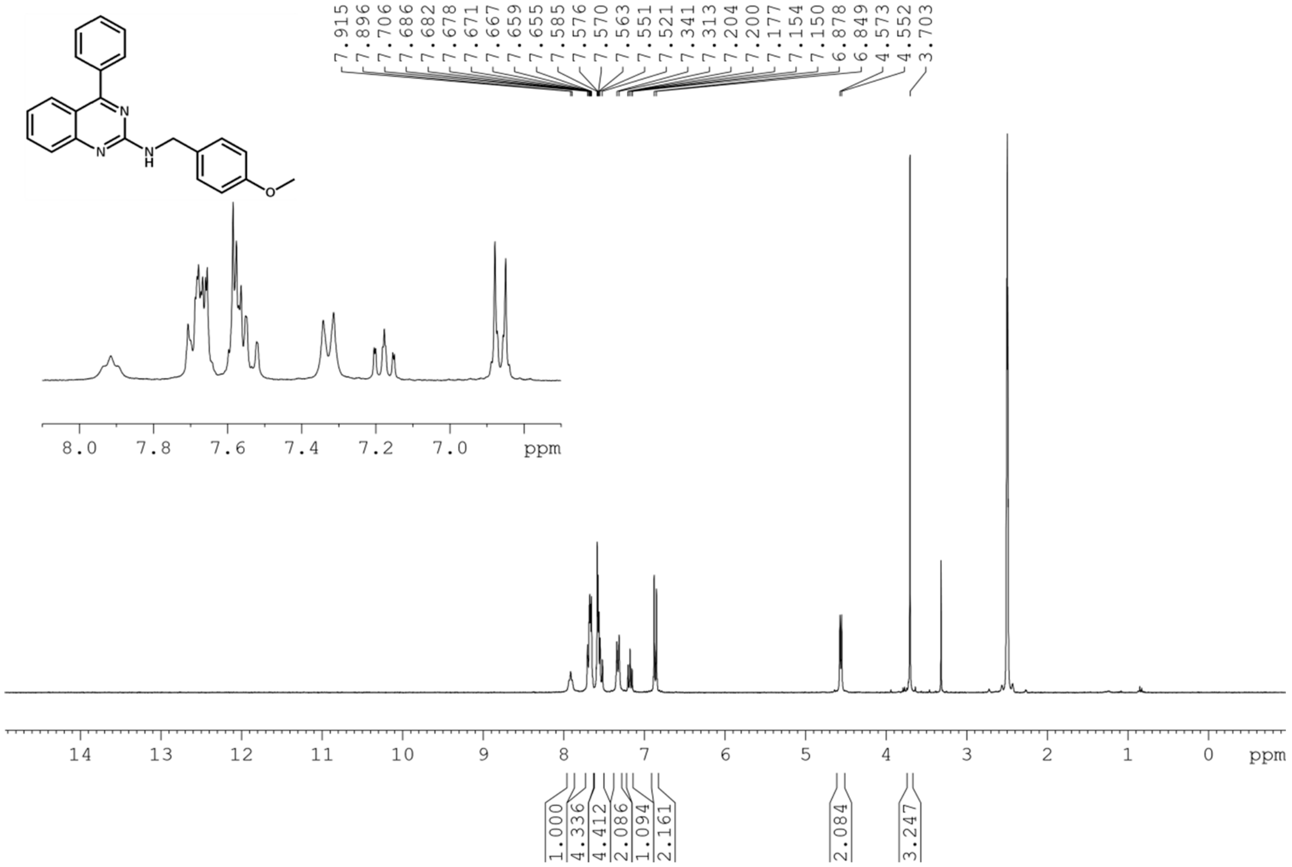


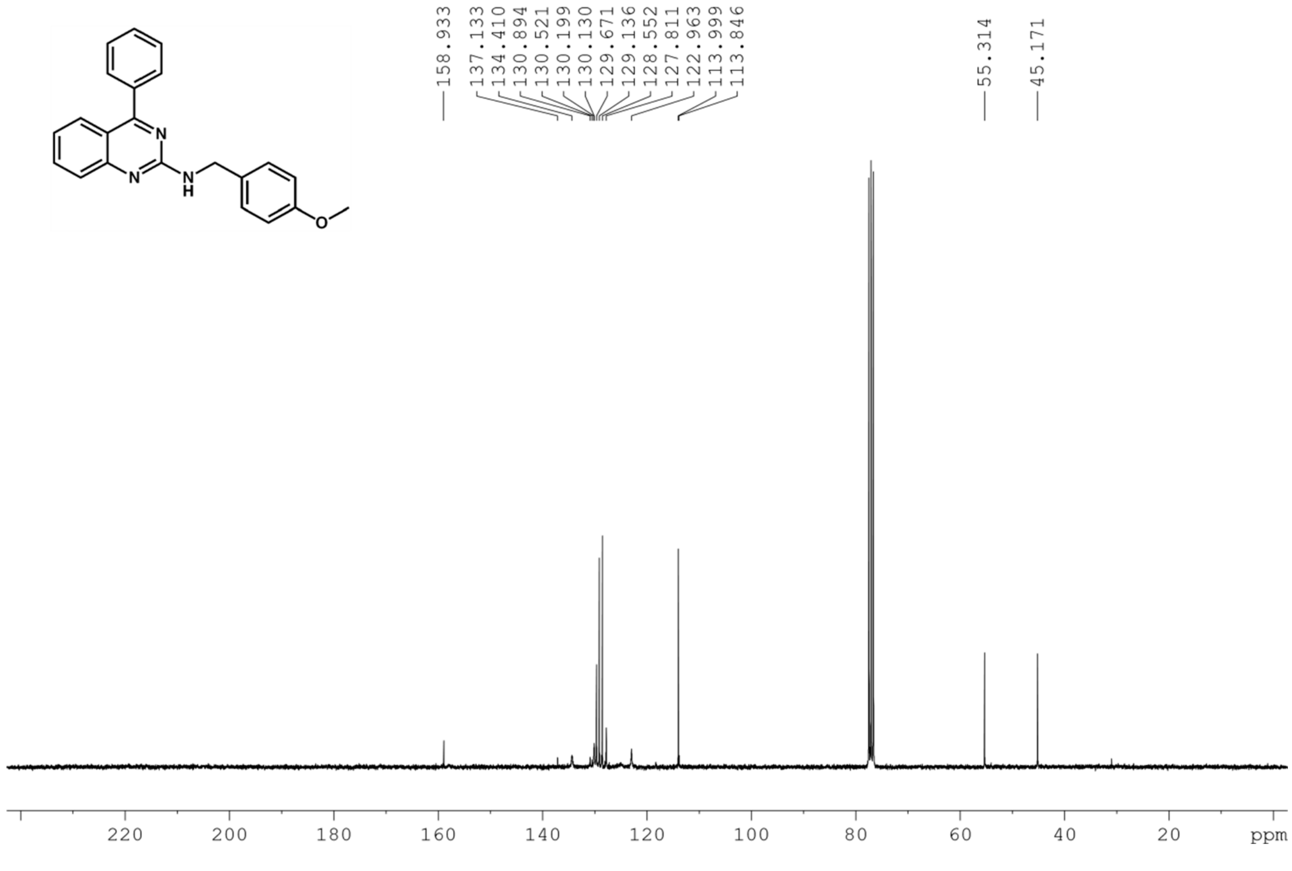

**LC-MS mass spectrum of compound 9e**

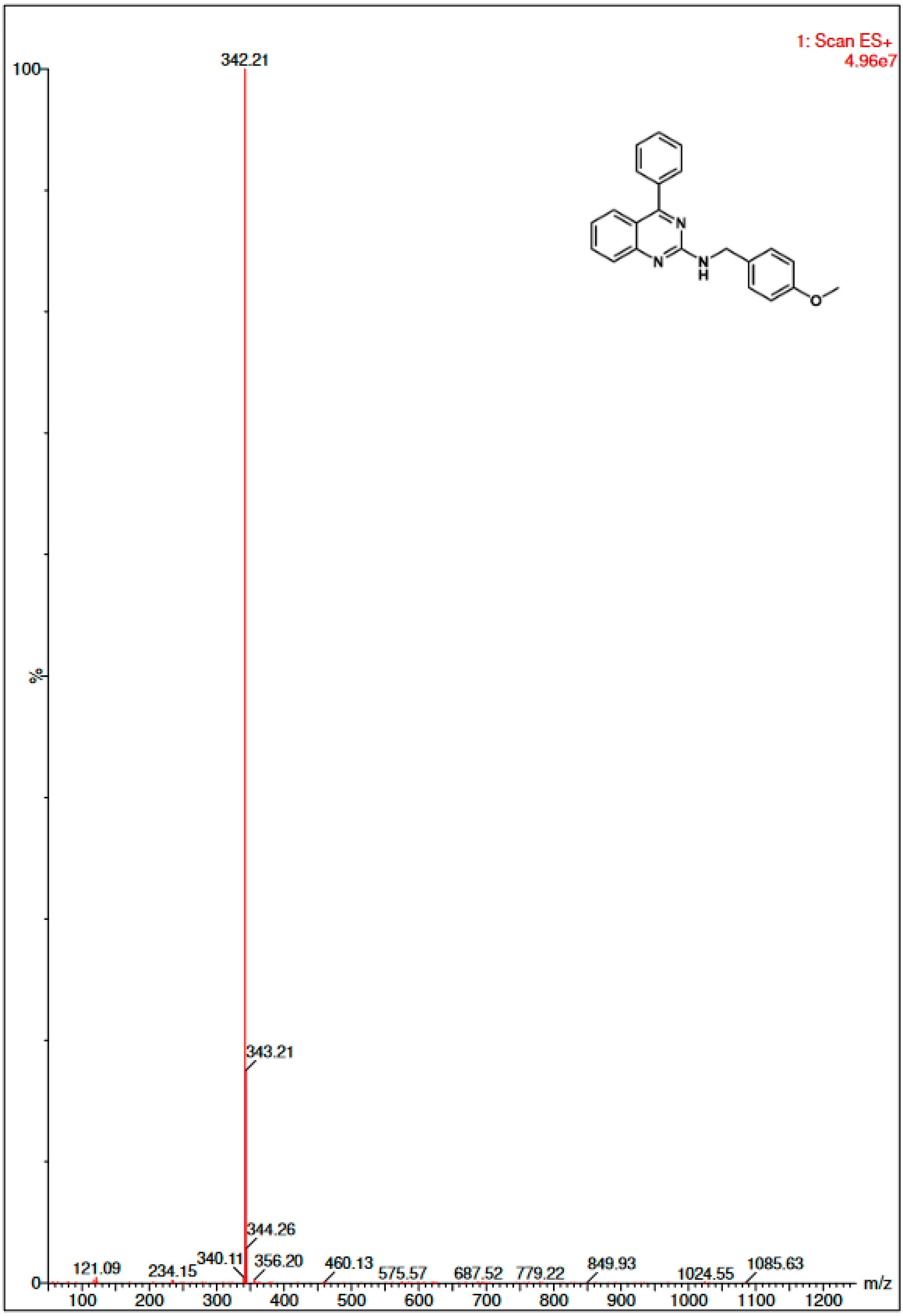

**1H- and 13C-NMR Spectra in $CDCl_3$ of compound 9f**

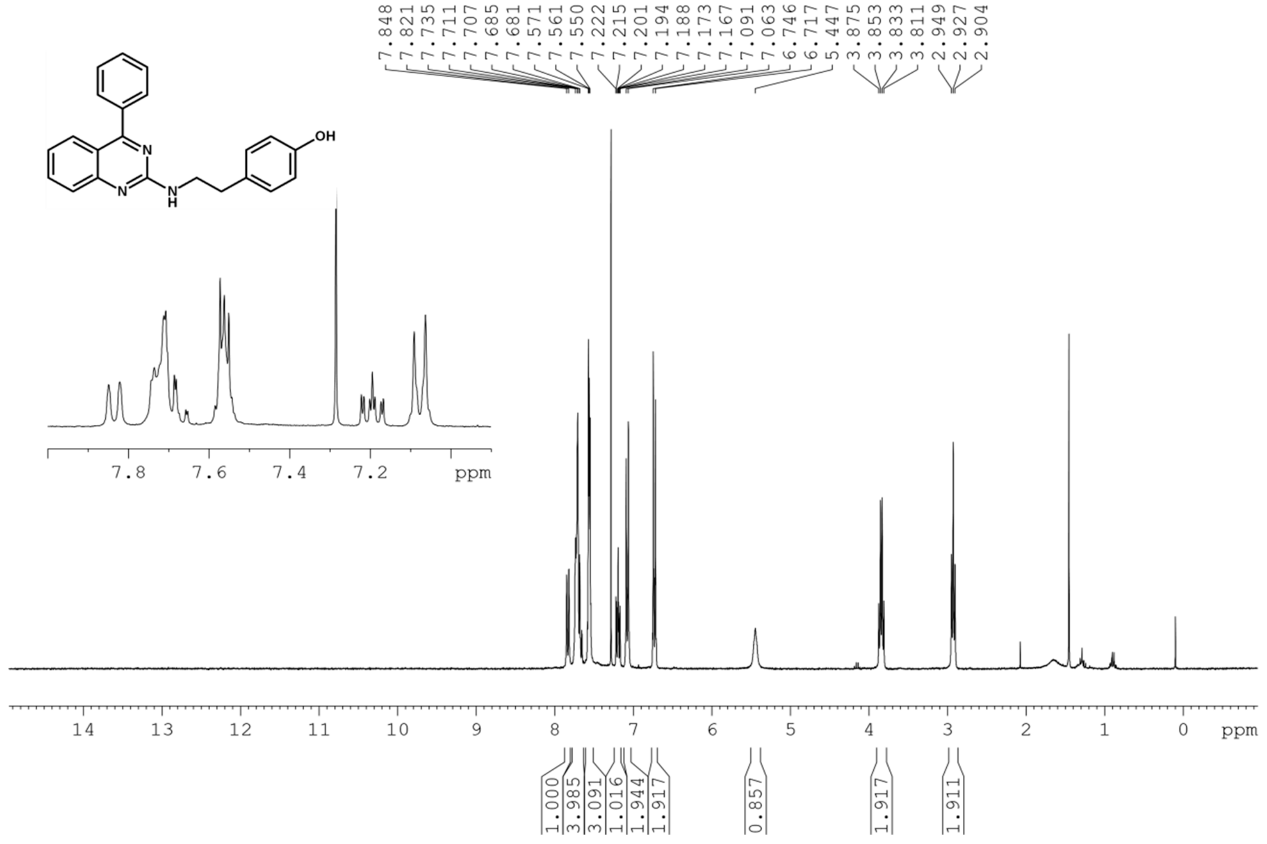


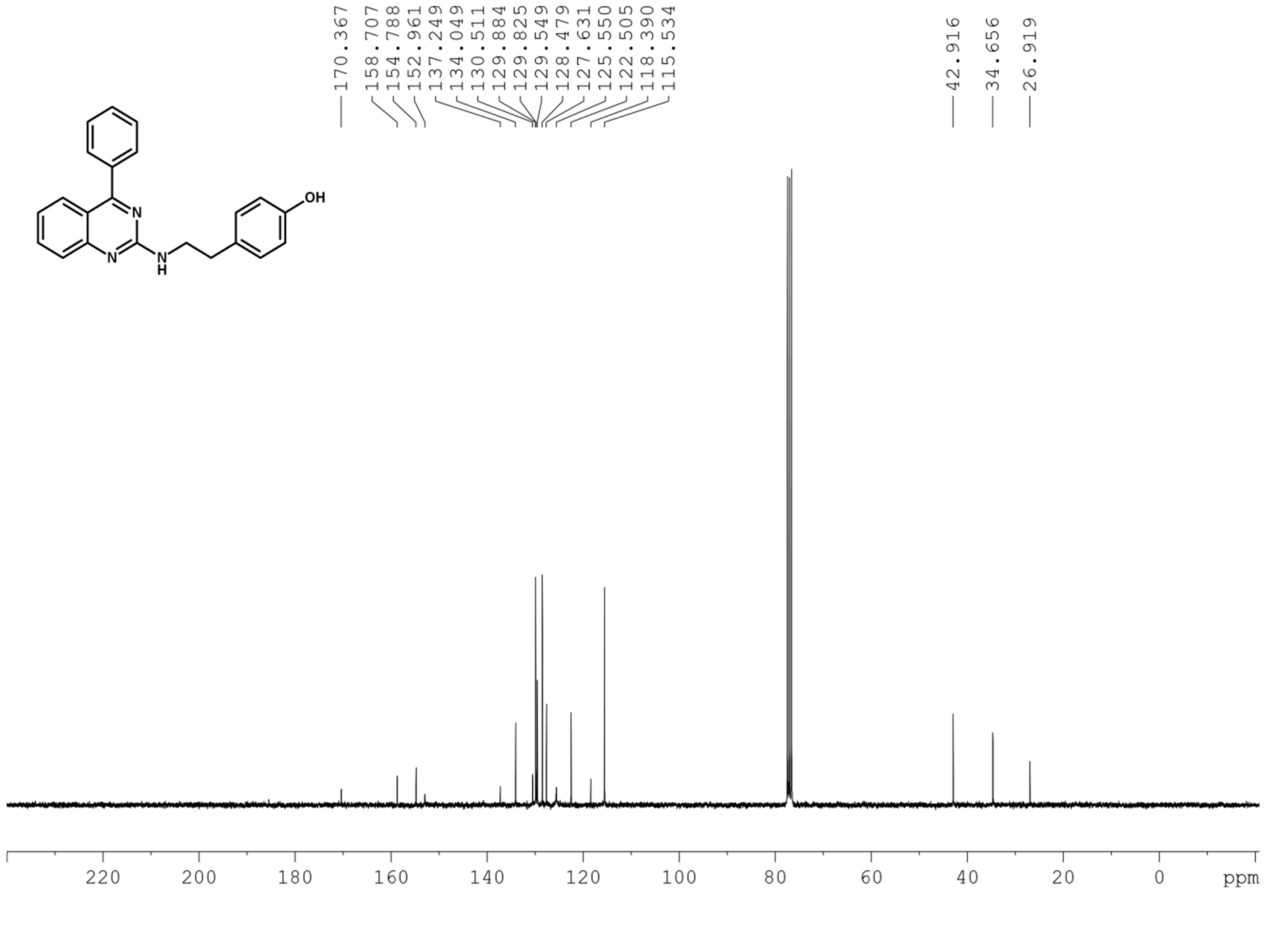

LC-MS mass spectrum of compound 9f

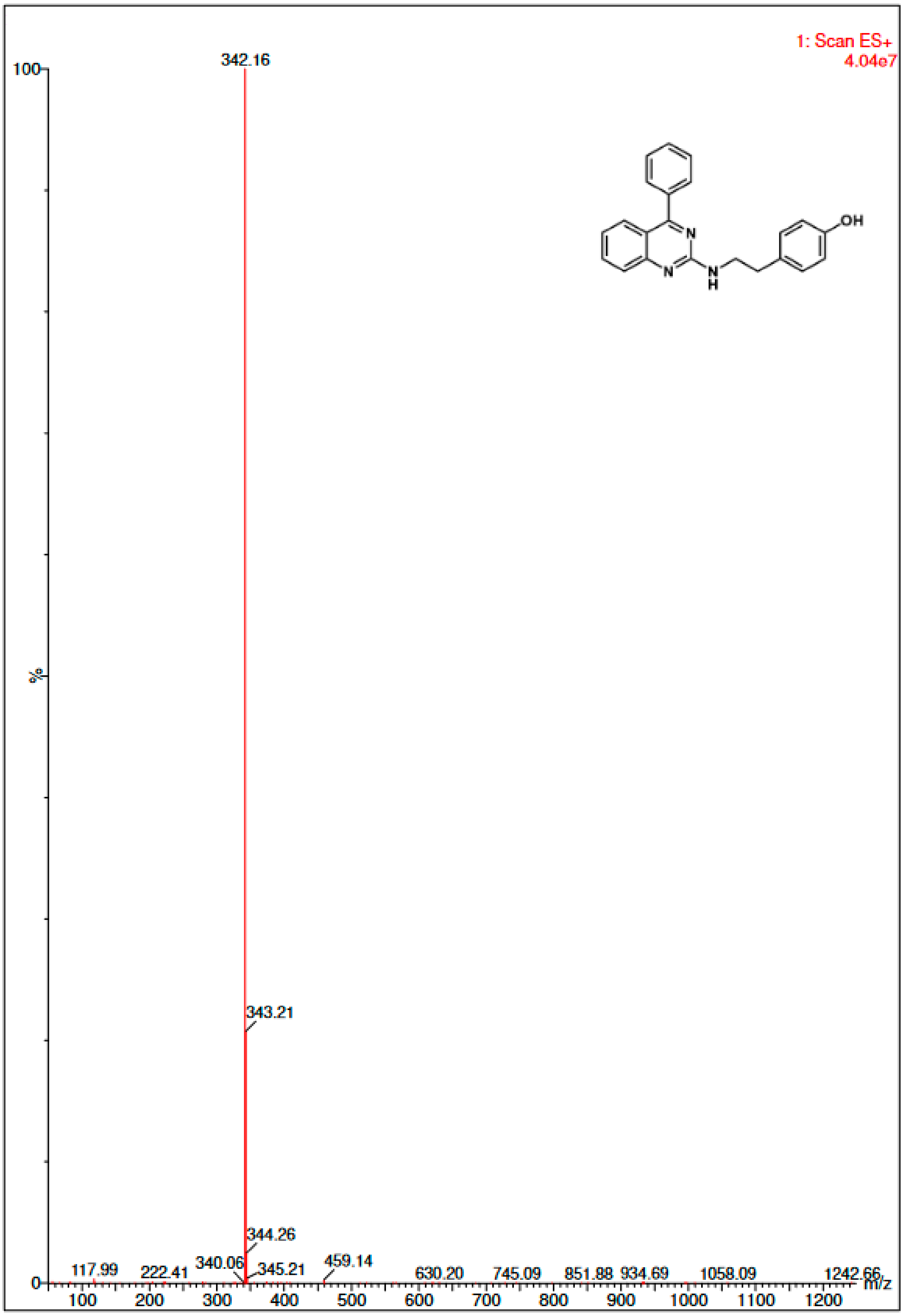

**1H- and 13C-NMR Spectra in $CDCl_3$ of compound 9g**

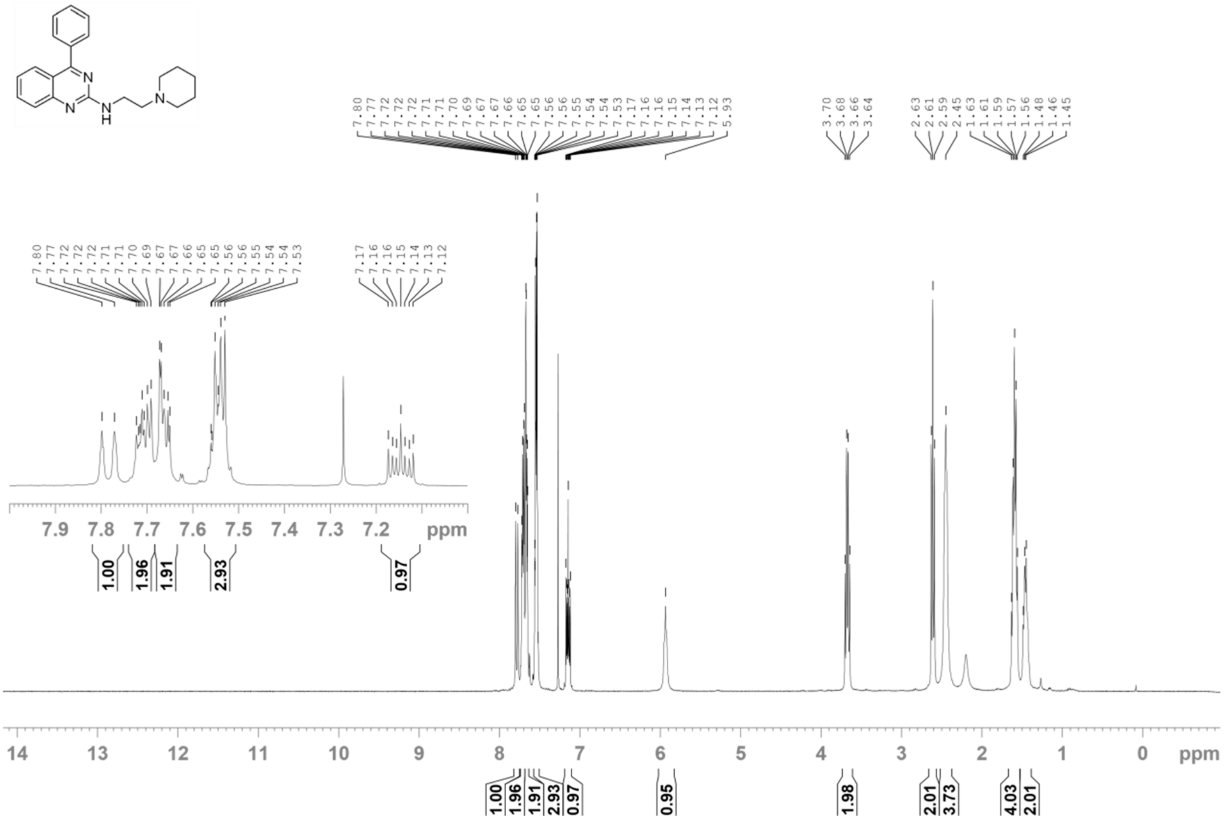

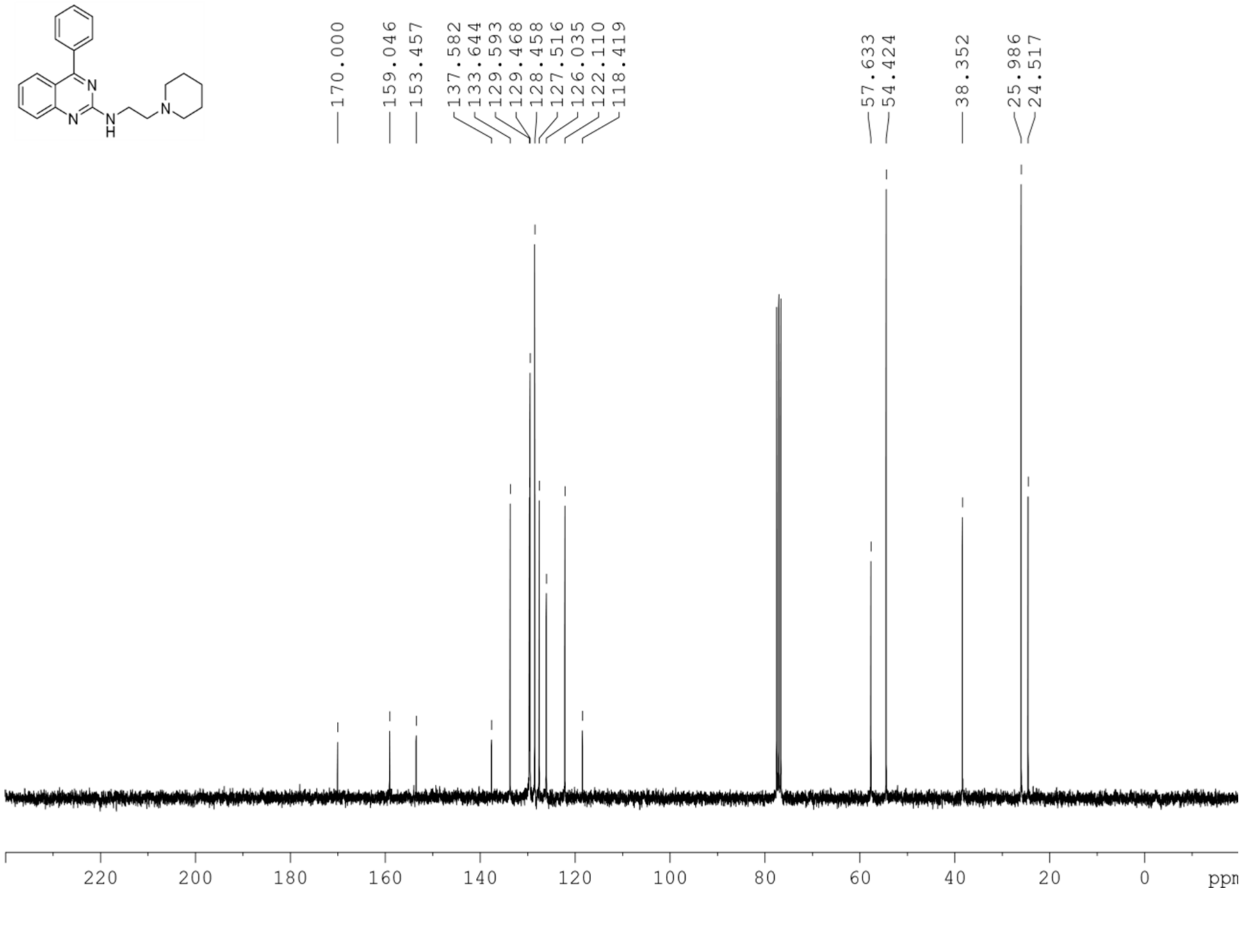

**LC-MS mass spectrum of compound 9g**

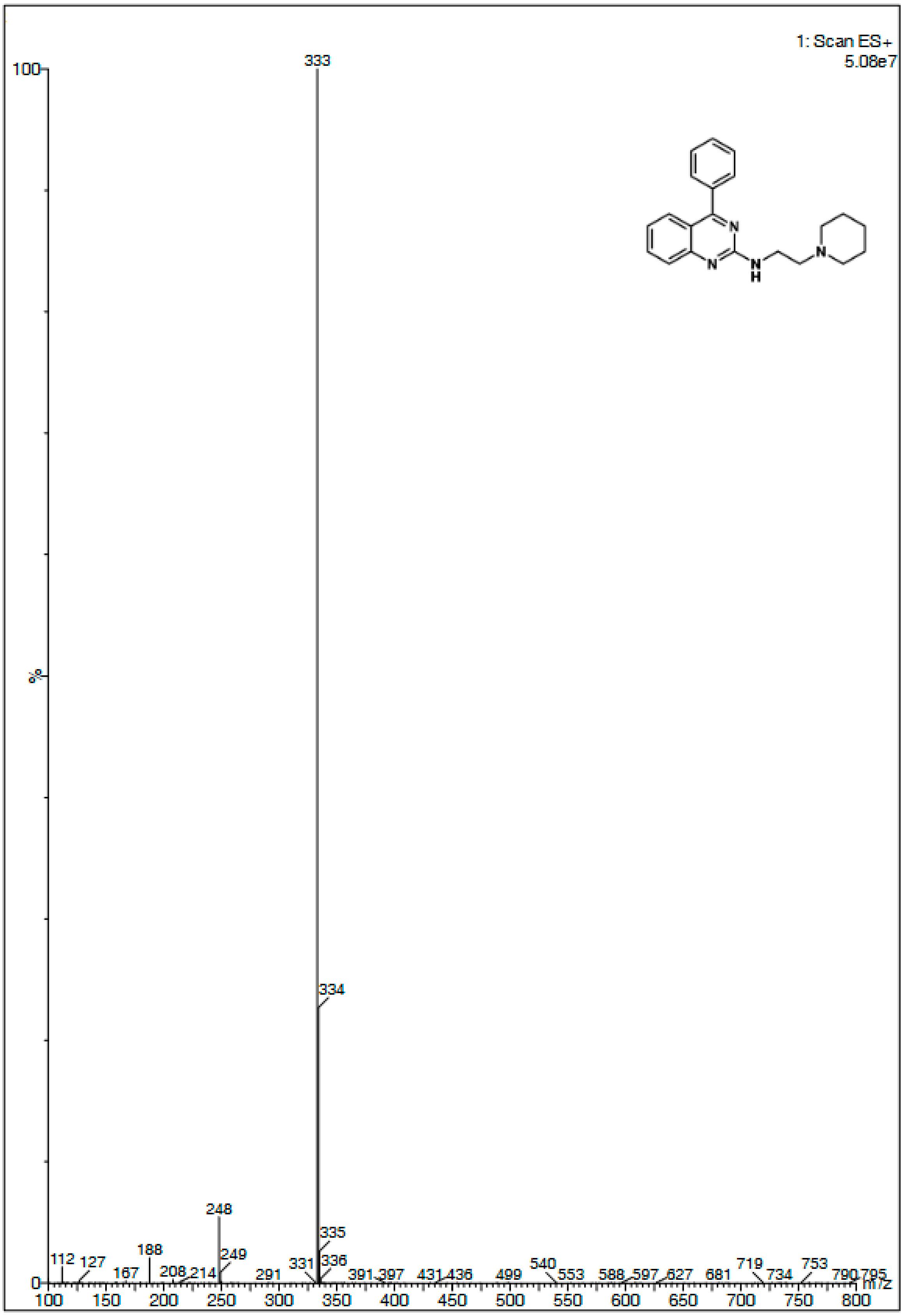

## 1H- and 13C-NMR Spectra in DMSO-d6 of compound 9h

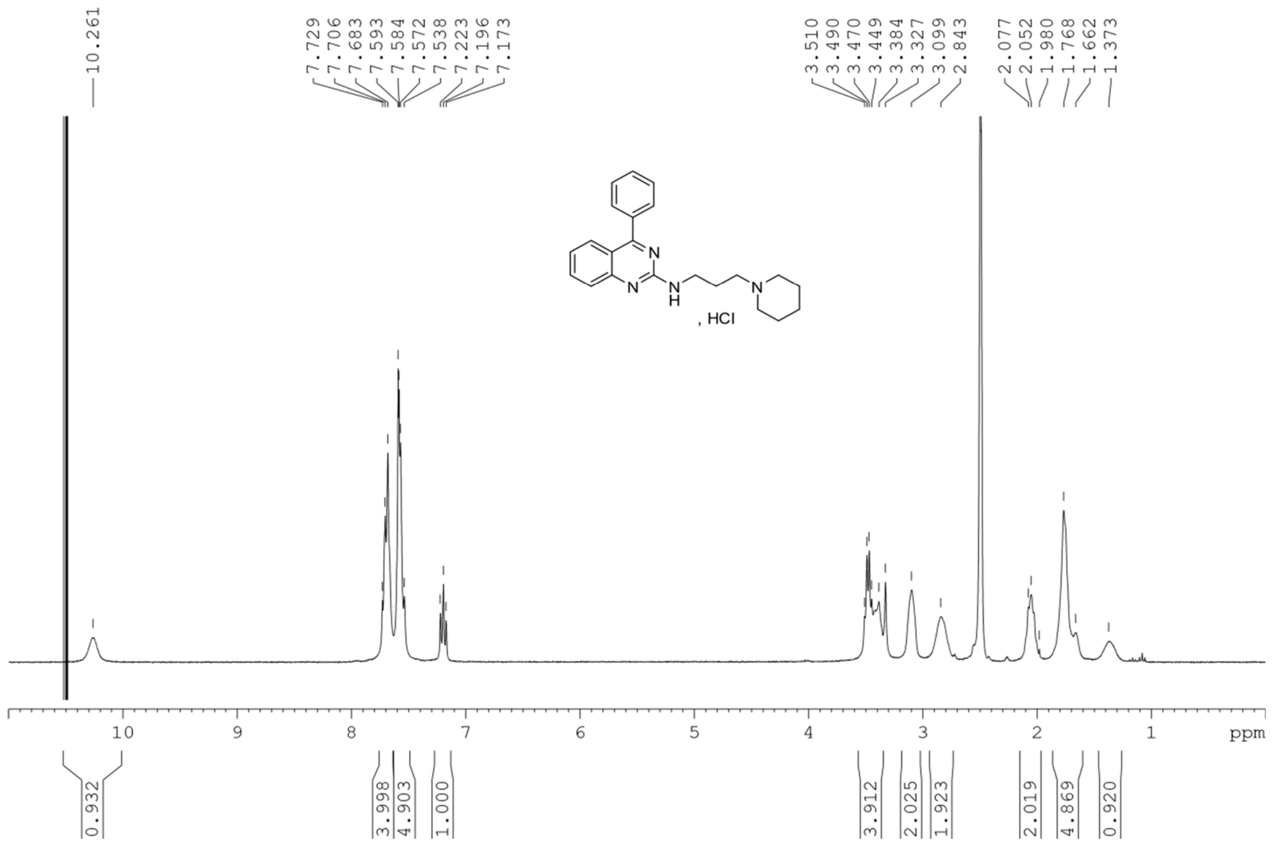


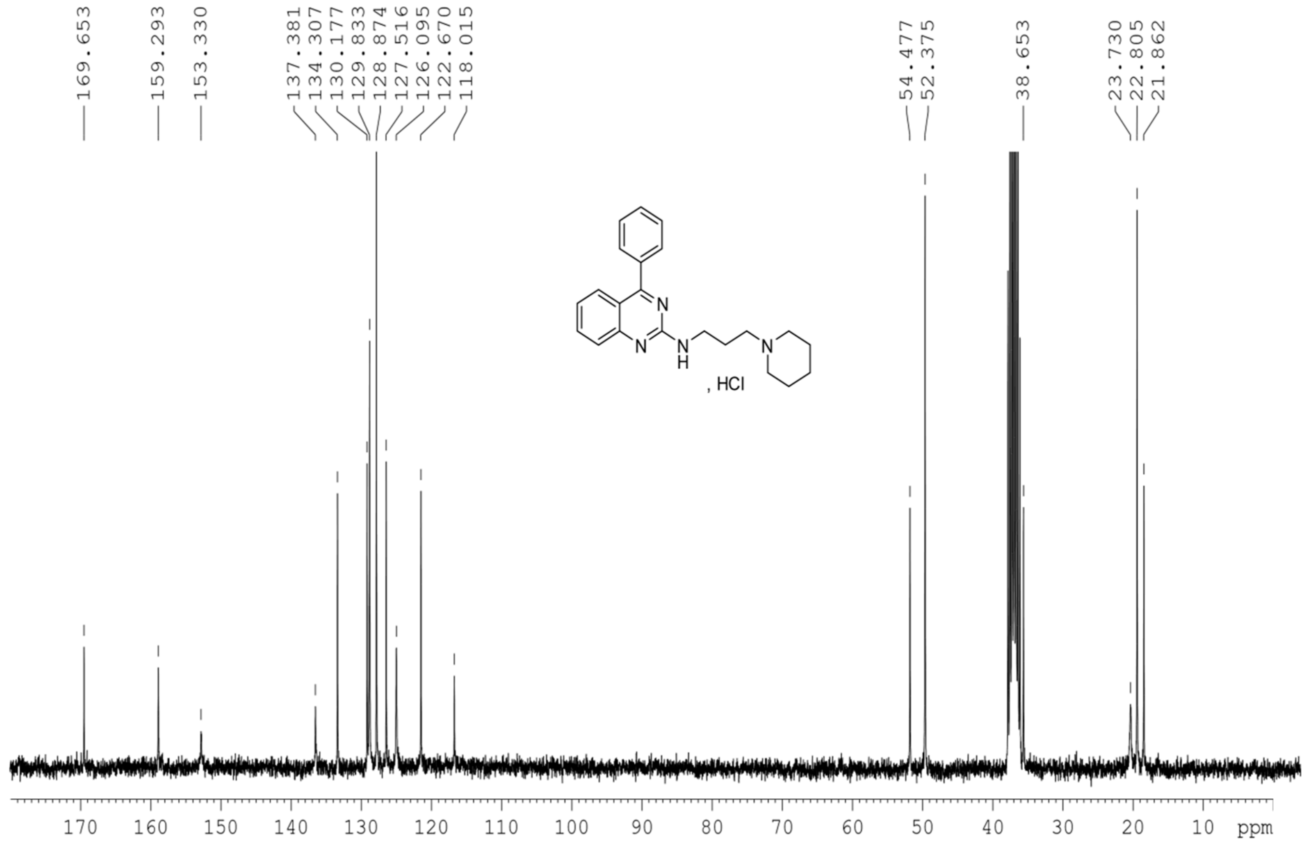

**LC-MS mass spectrum of compound 9h**

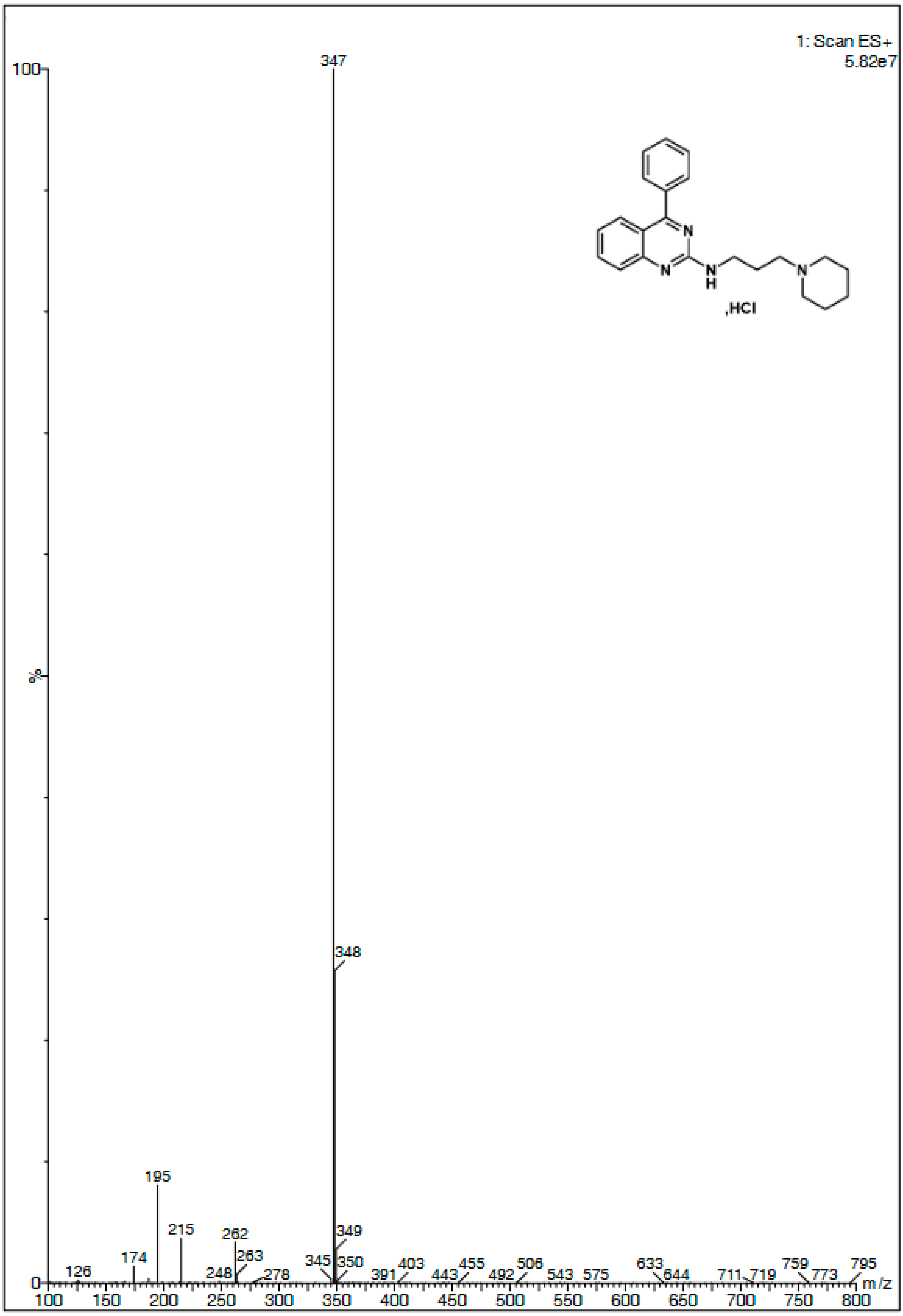

**1H- and 13C-NMR Spectra in $CDCl_3$ of compound 9i**

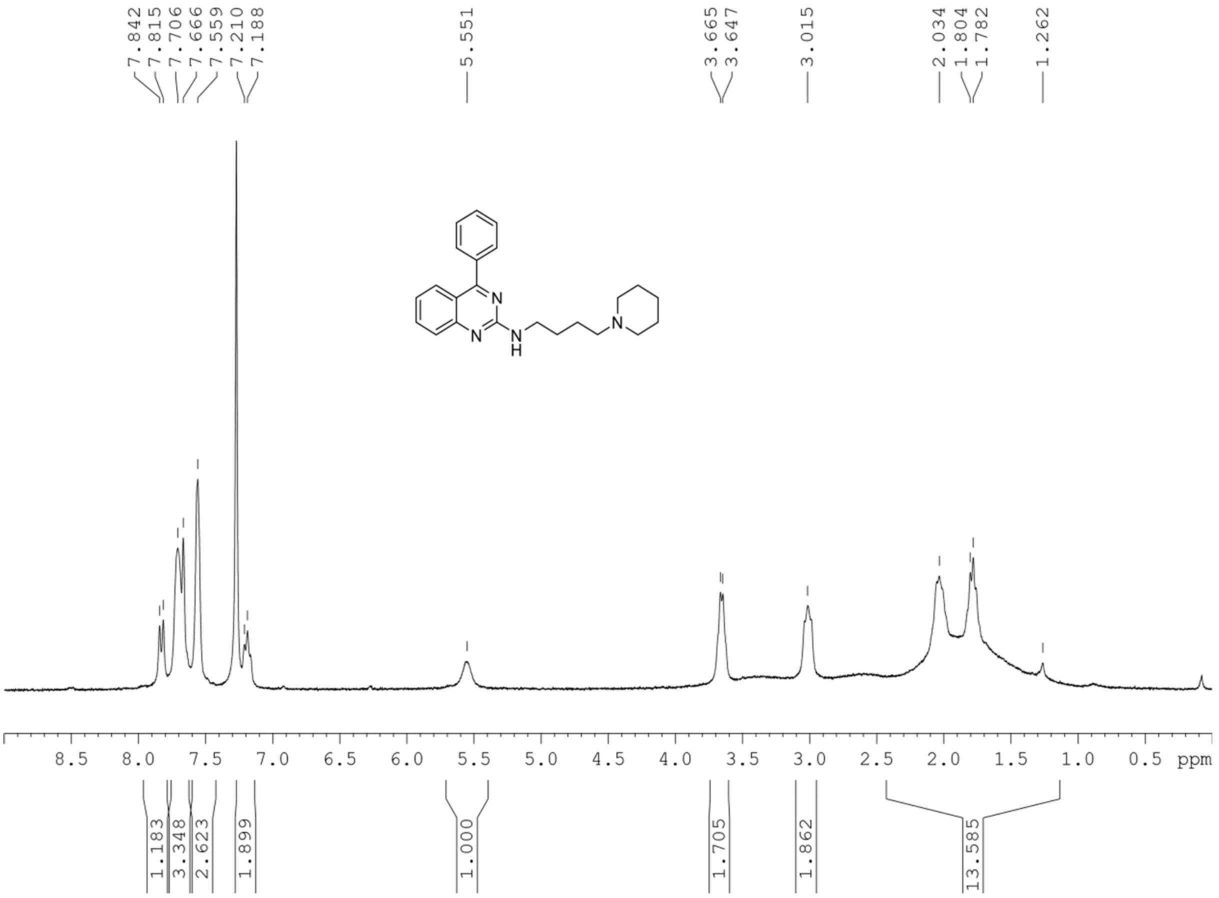


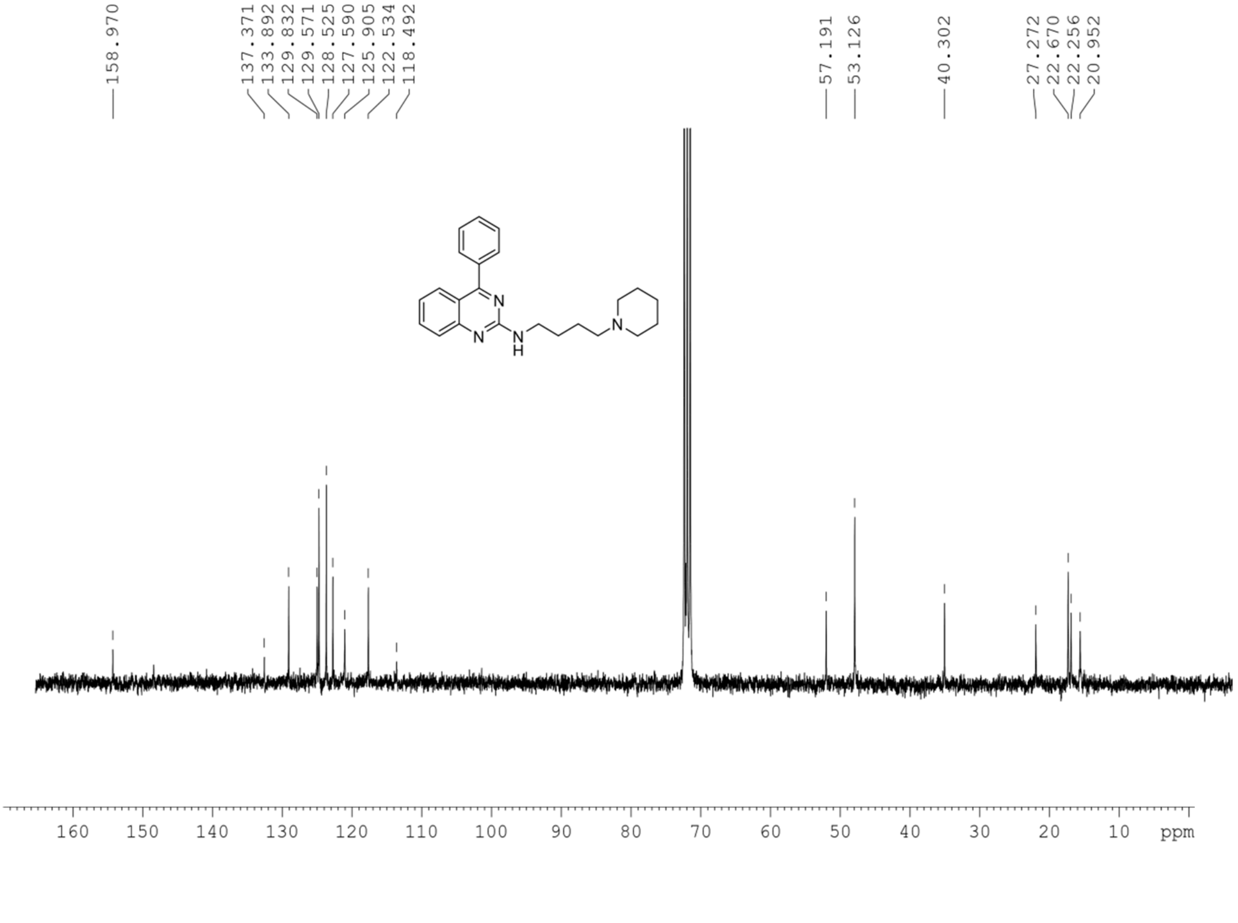

**LC-MS mass spectrum of compound 9i**

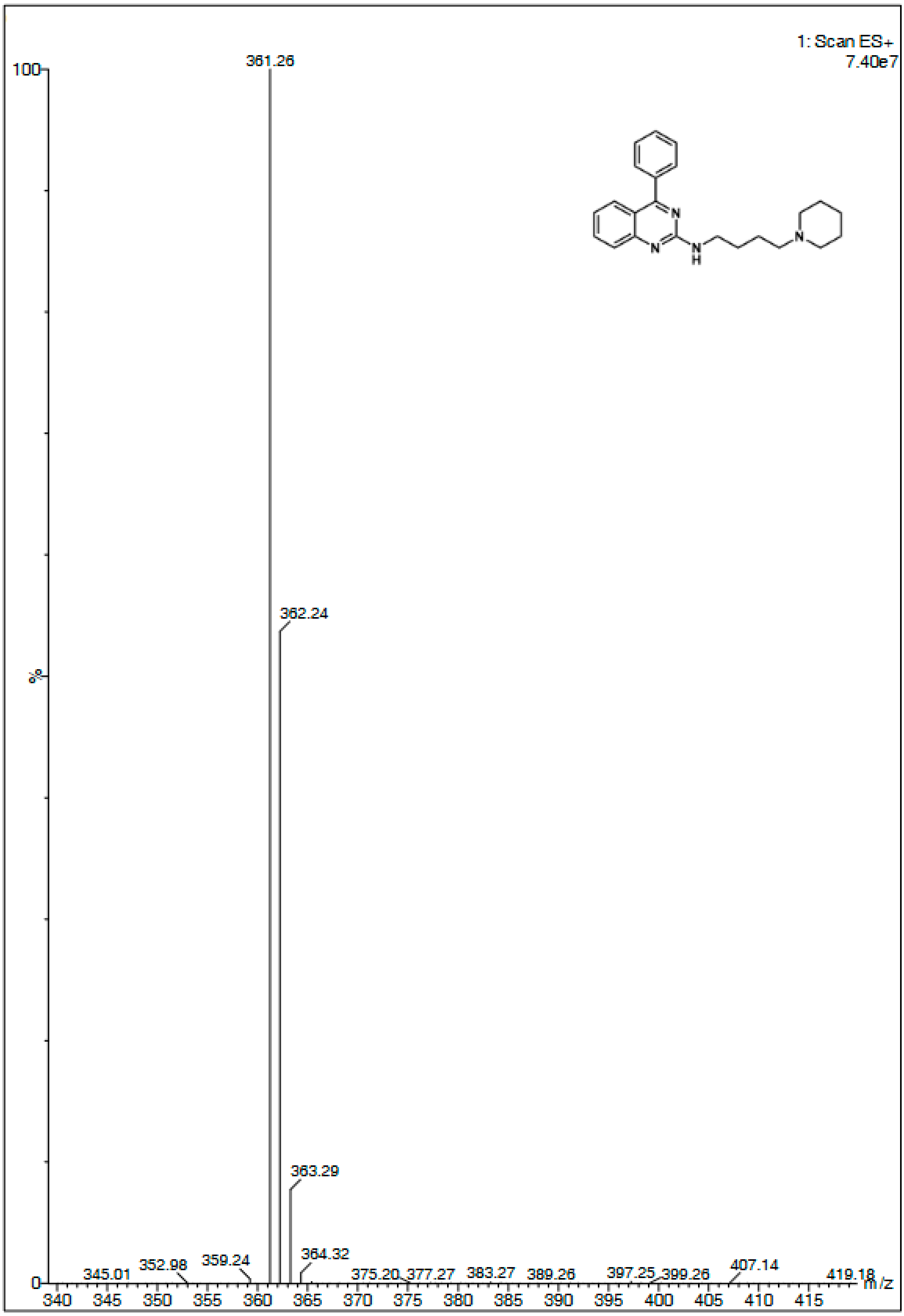

**1H- and 13C-NMR Spectra in $CDCl_3$ of compound 9j**

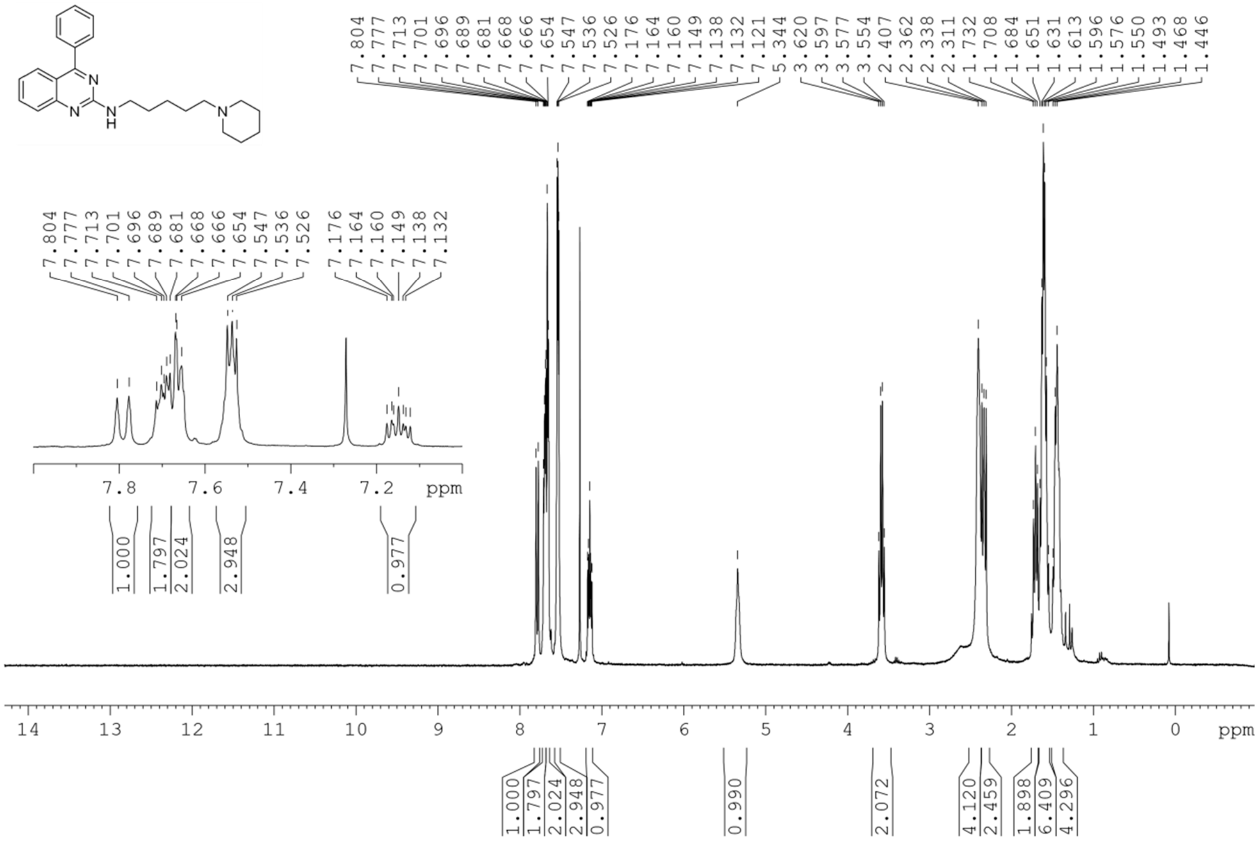


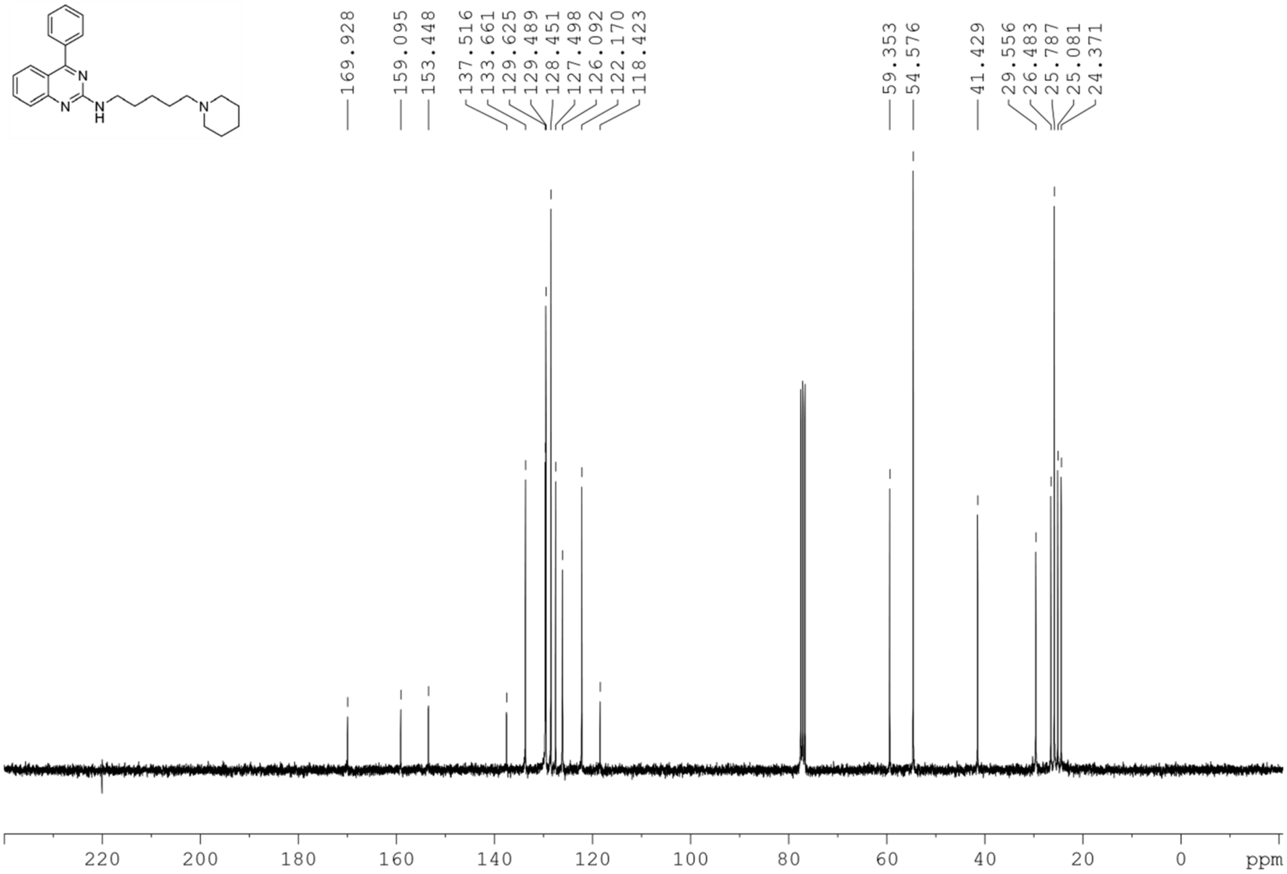

**LC-MS mass spectrum of compound 9j**

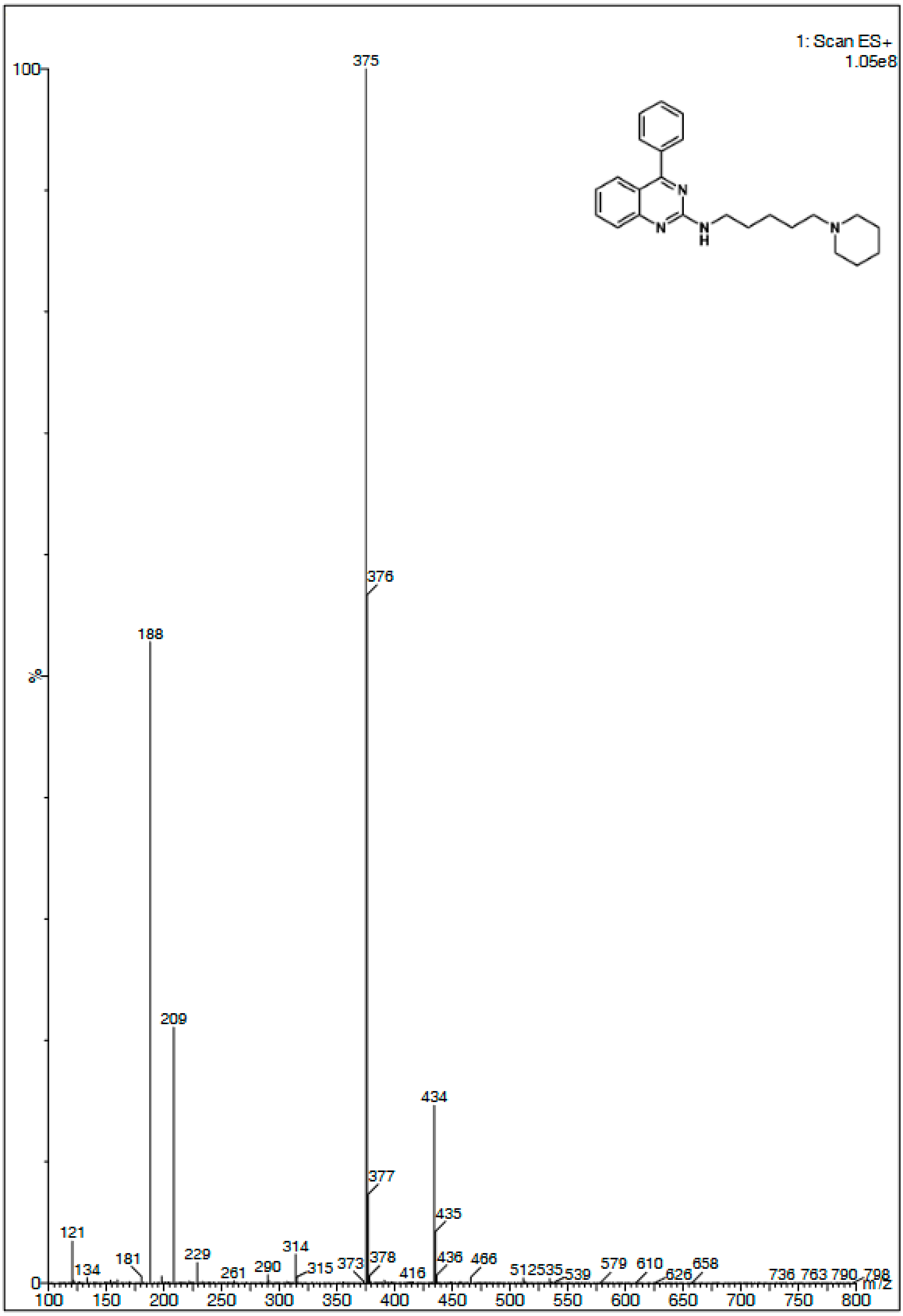

## 1H- and 13C-NMR Spectra in $CDCl_3$ of compound 9k

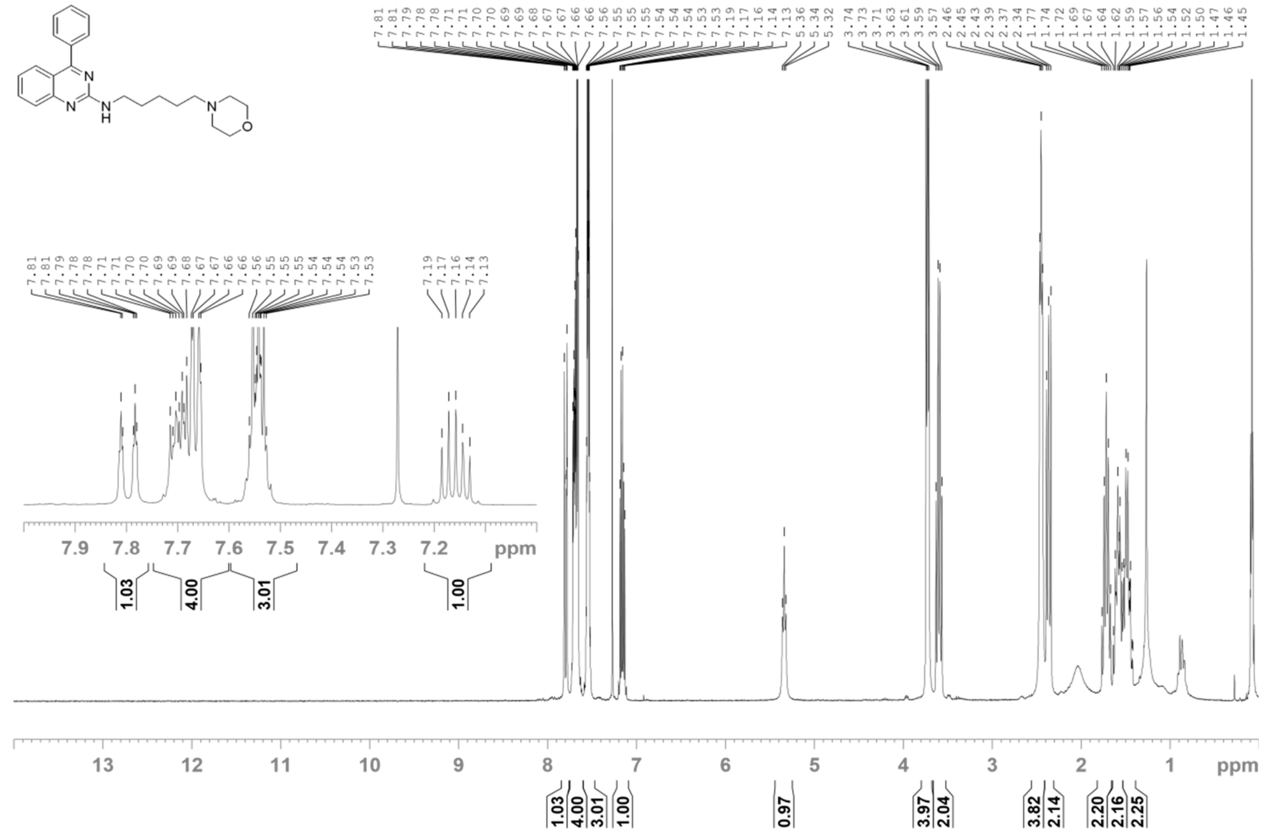


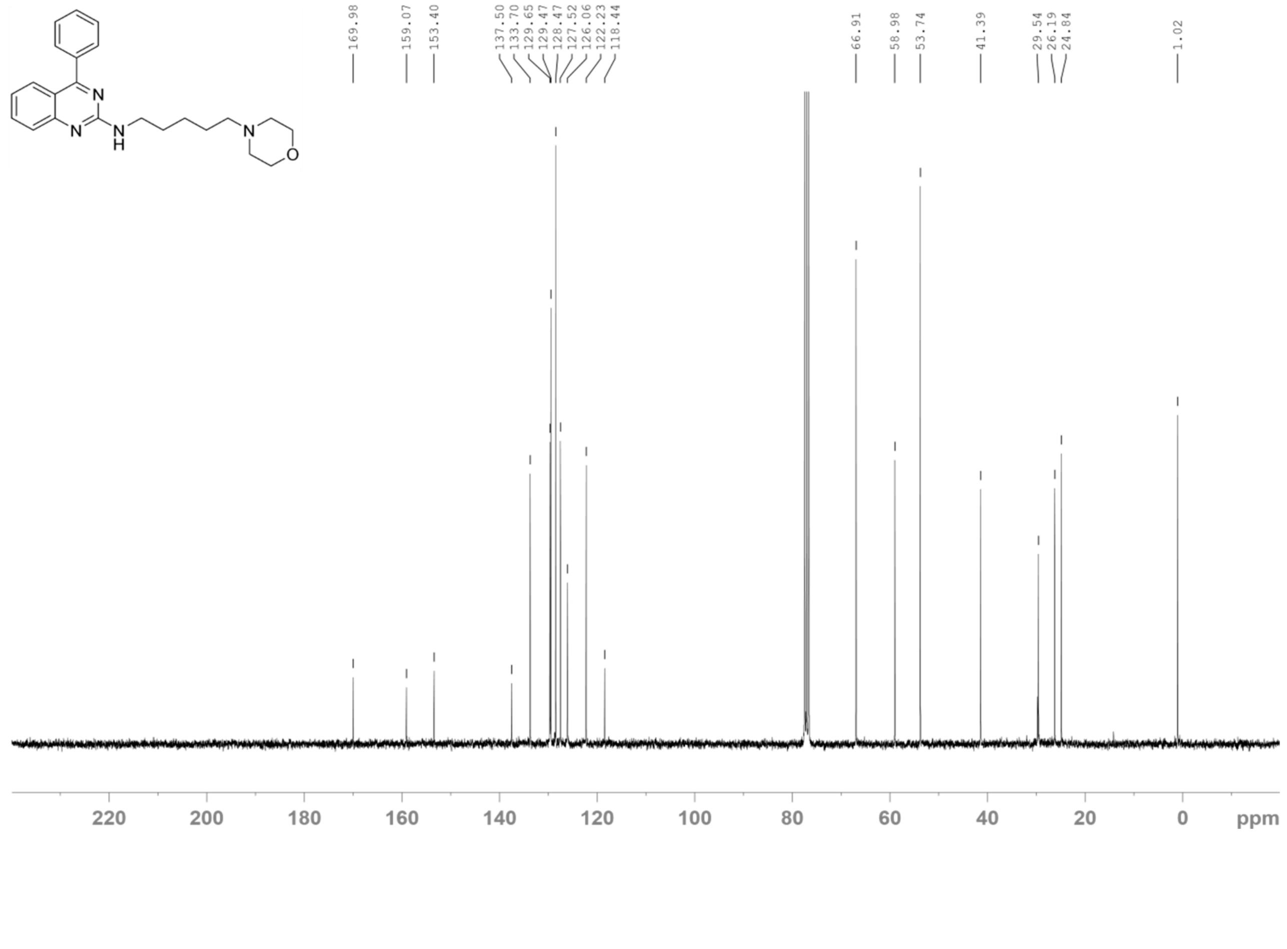

LC-MS mass spectrum of compound 9k

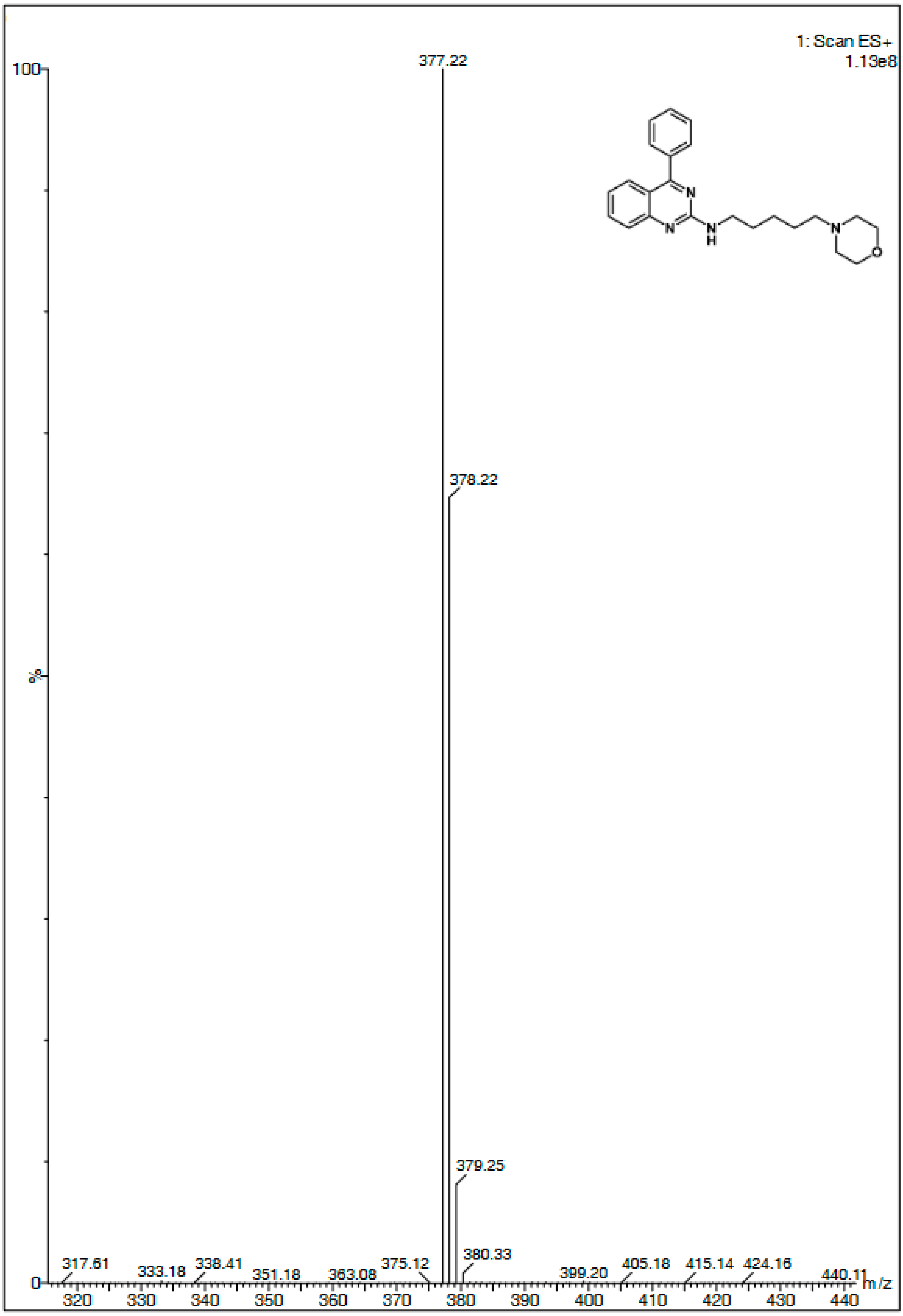

**1H- and 13C-NMR Spectra in $CDCl_3$ of compound 9l**

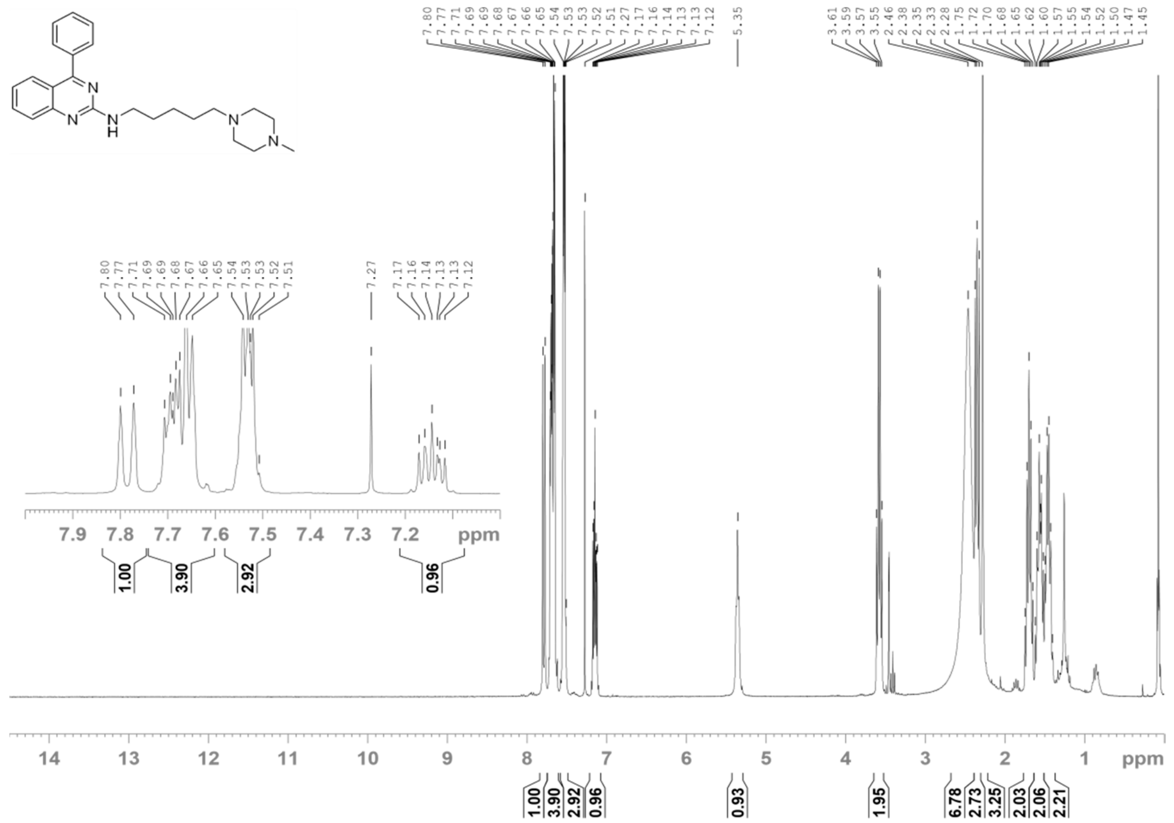


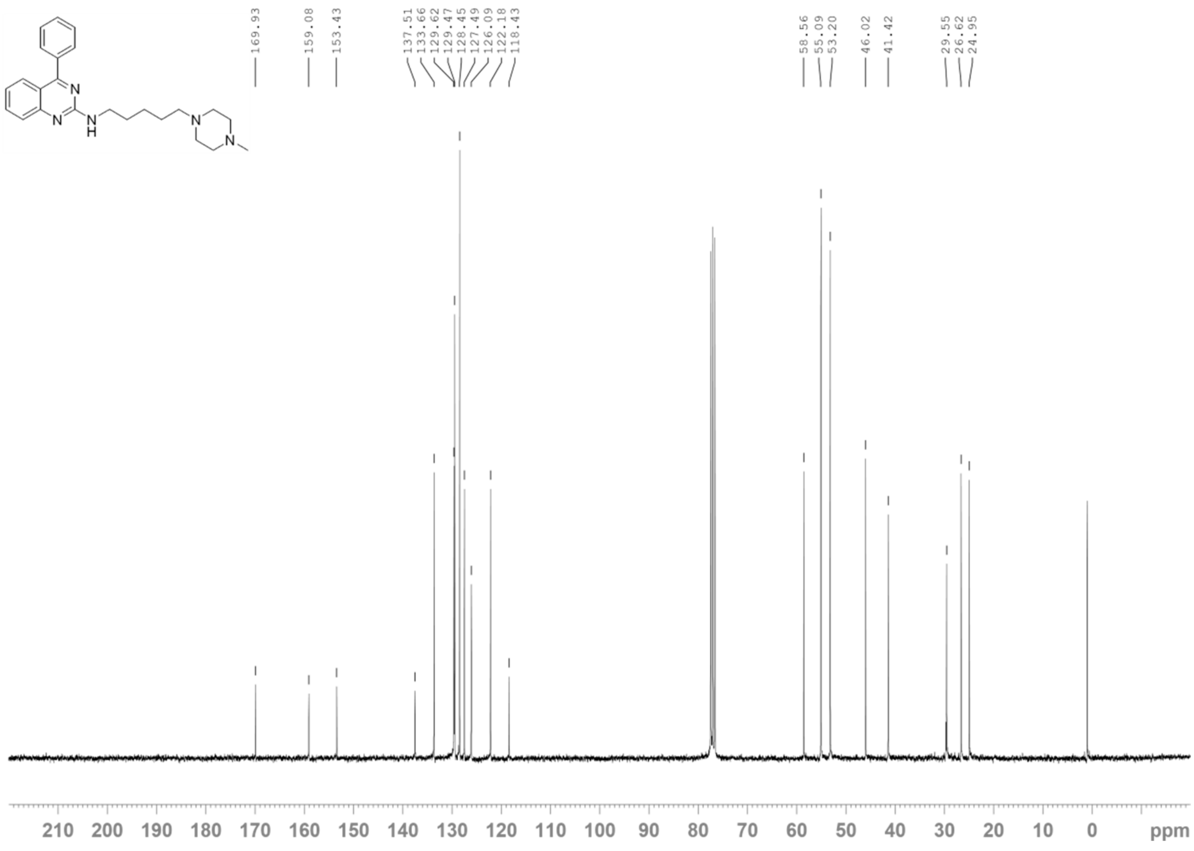

**LC-MS mass spectrum of compound 9l**

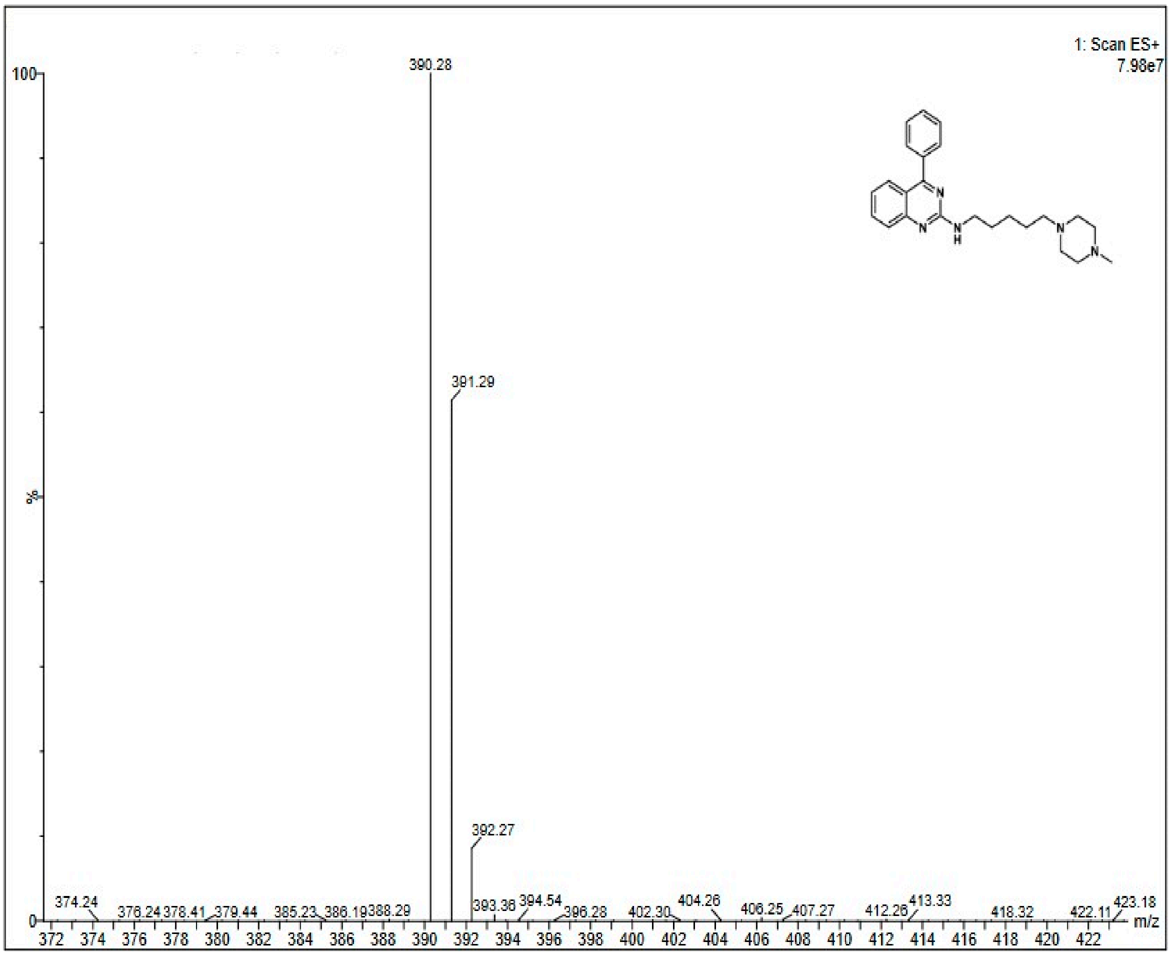

**1H- and 13C-NMR Spectra in $CDCl_3$ of compound 9m**

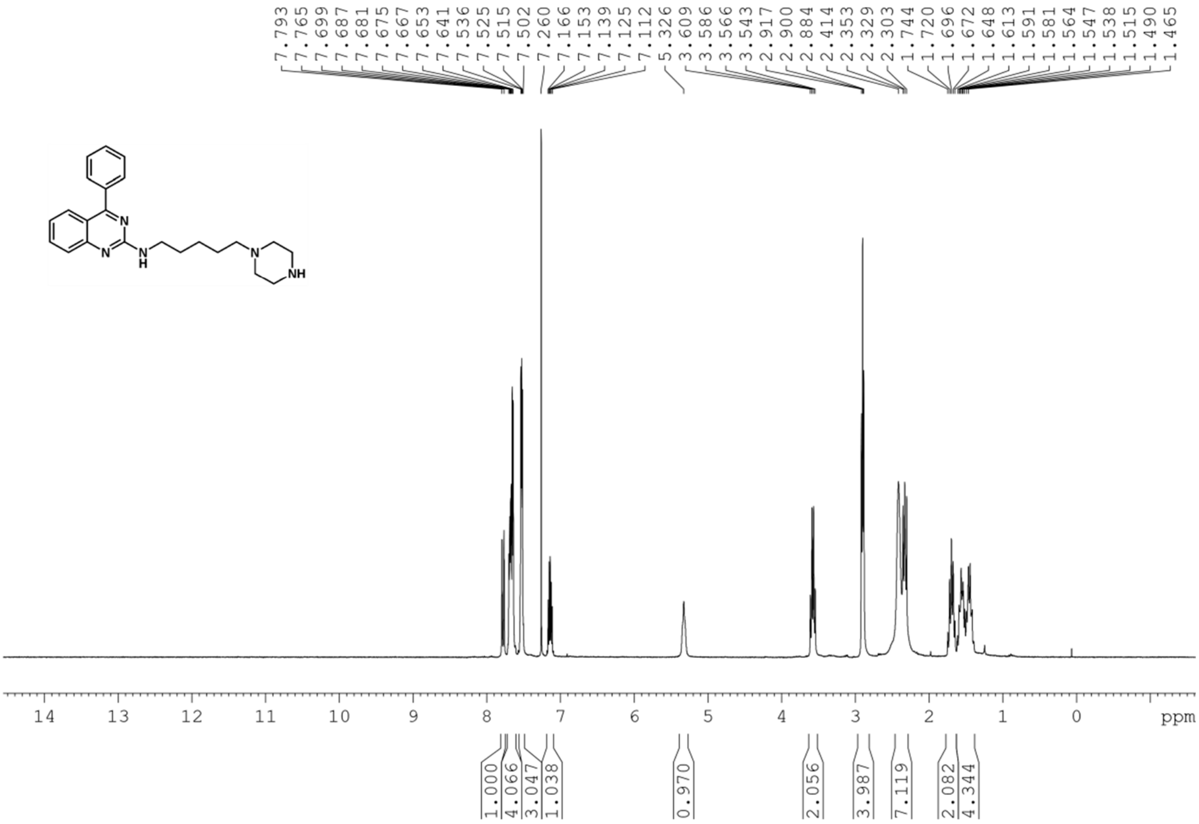


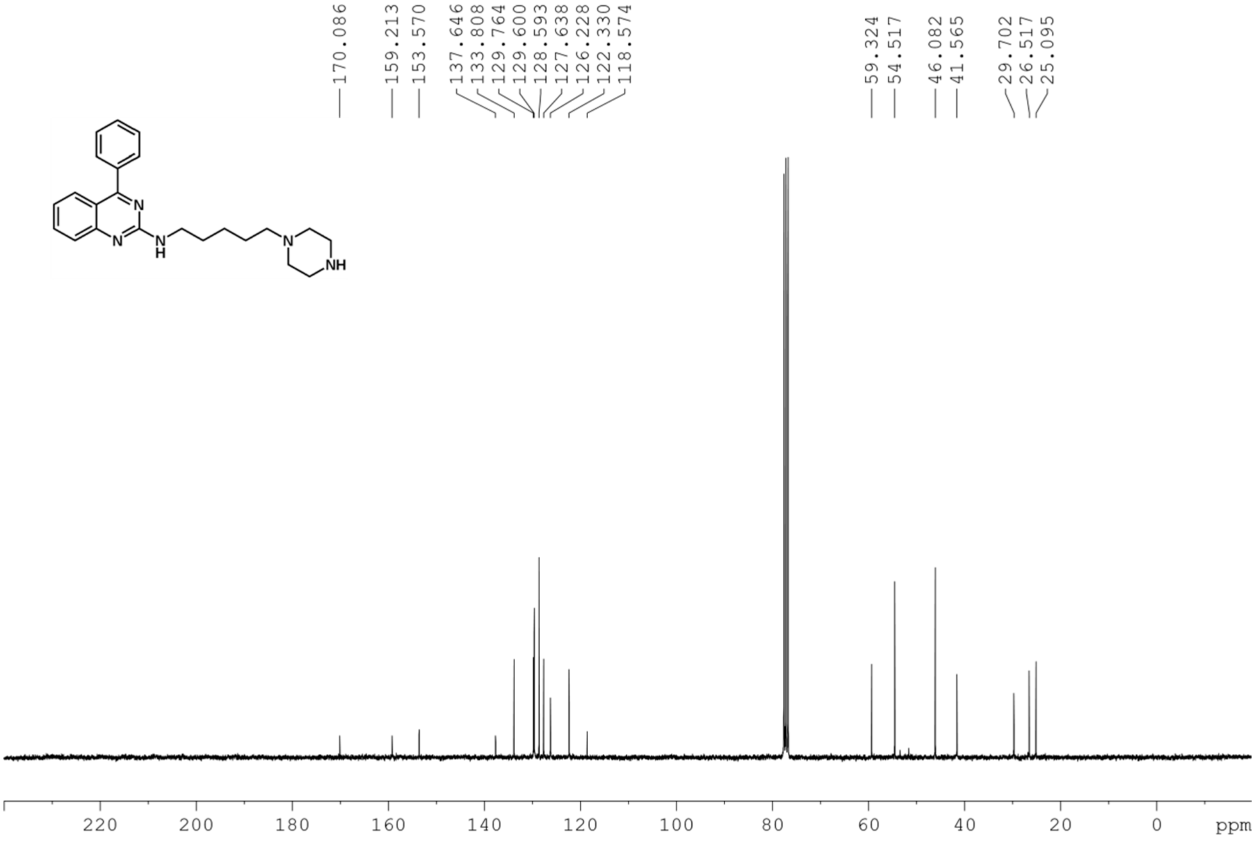

**LC-MS mass spectrum of compound 9m**

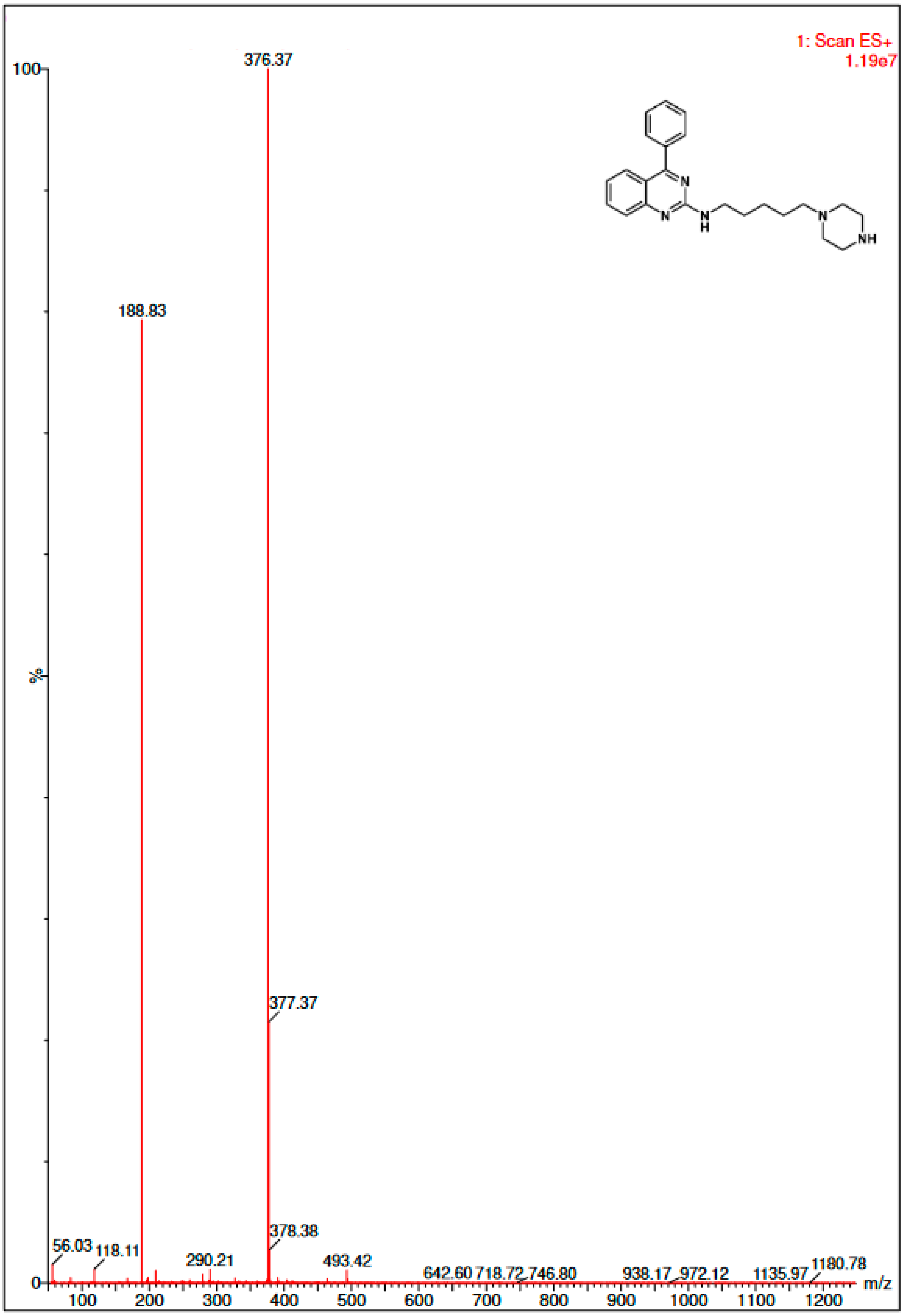

**1H- and 13C-NMR Spectra in $CDCl_3$ of compound 9n**

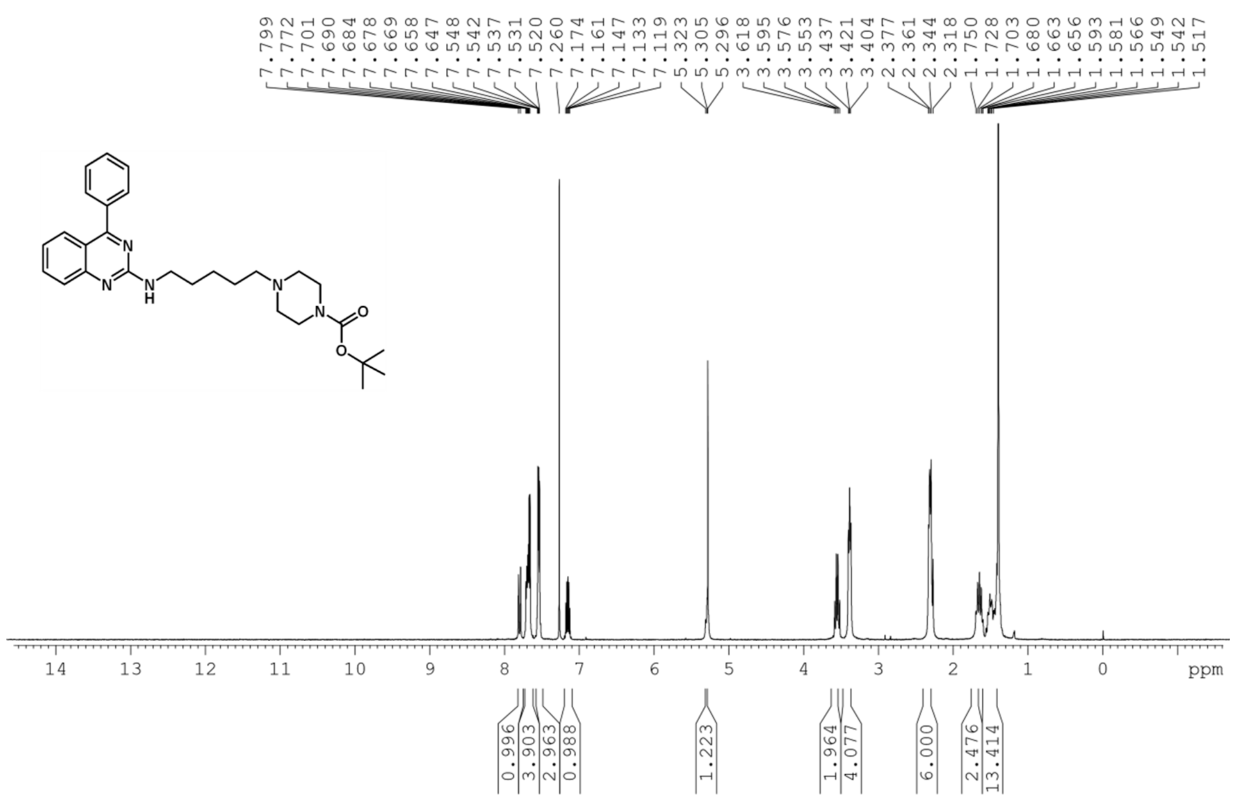

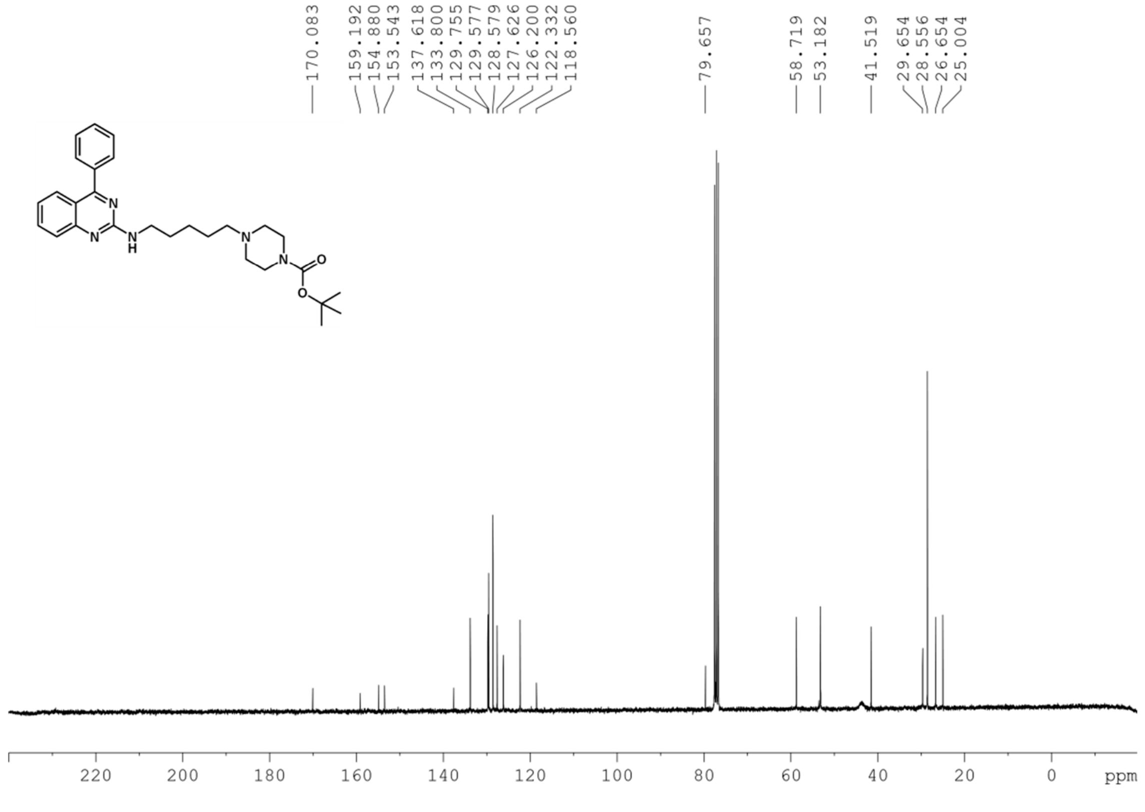

**LC-MS mass spectrum of compound 9n**

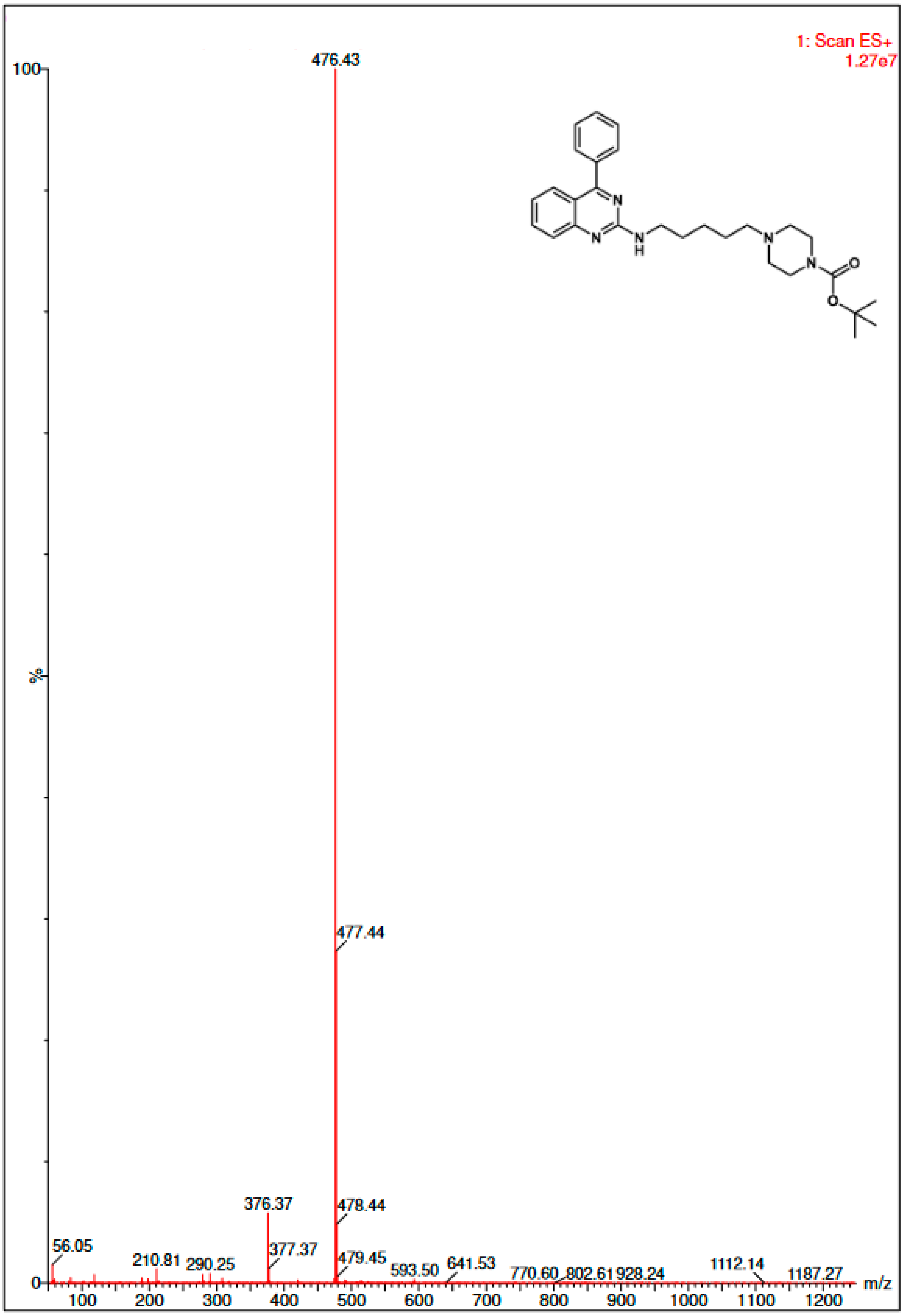

**1H- and 13C-NMR Spectra in $CDCl_3$ of compound 9o**

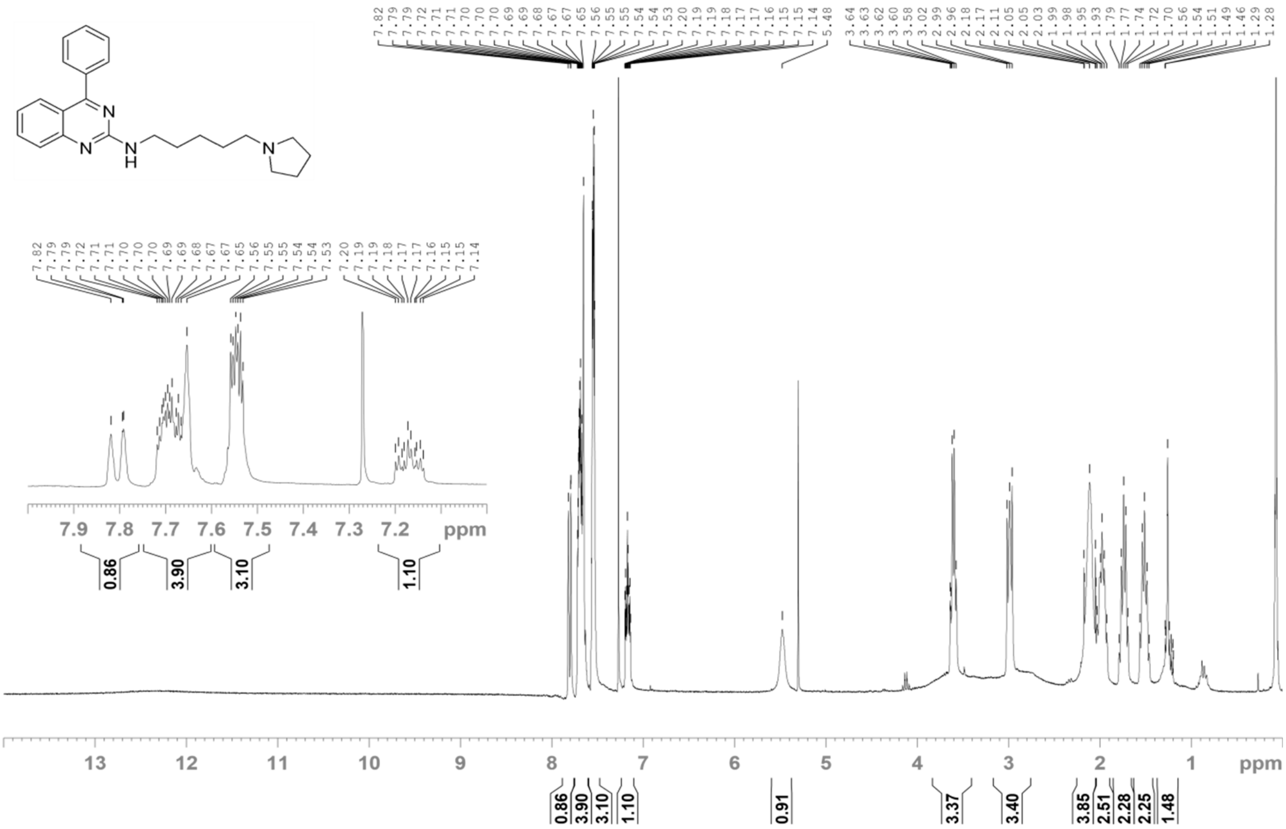

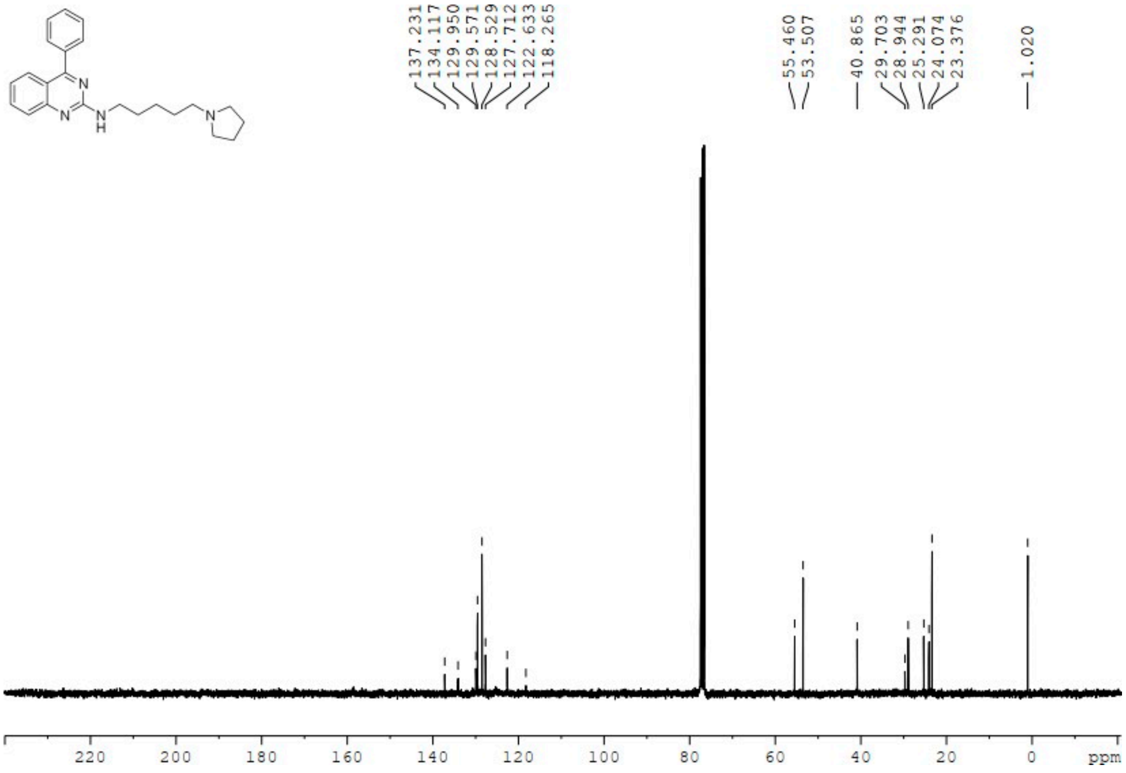

**LC-MS mass spectrum of compound 9o**

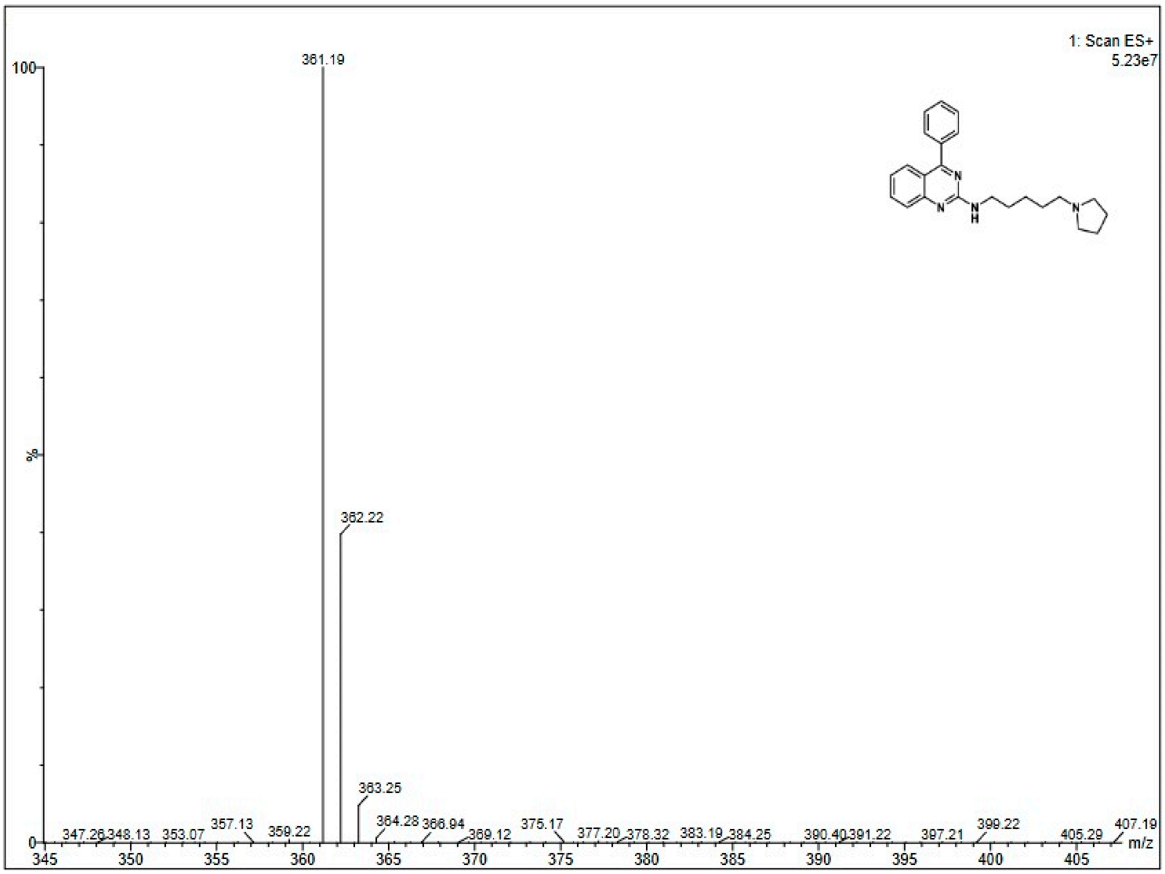

**1H- and 13C-NMR Spectra in $CDCl_3$ of compound 9p**

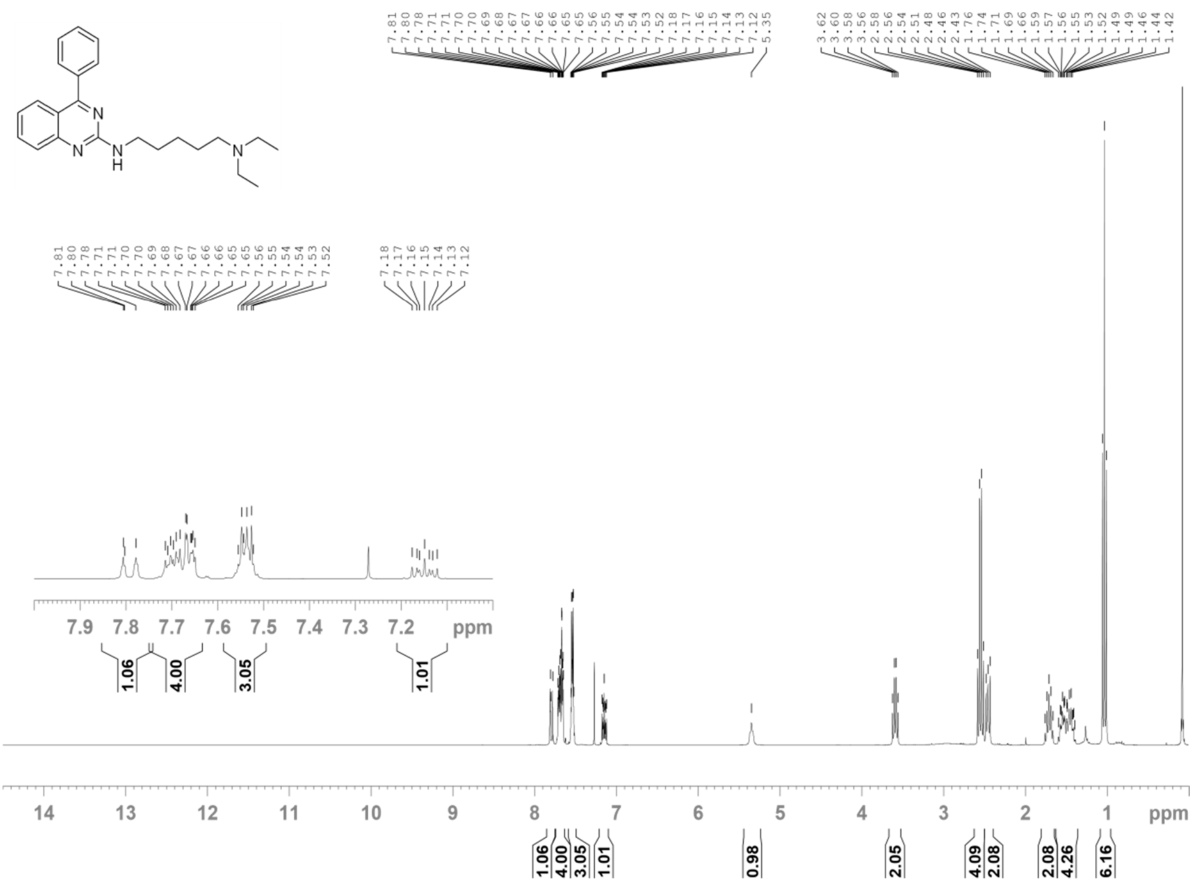

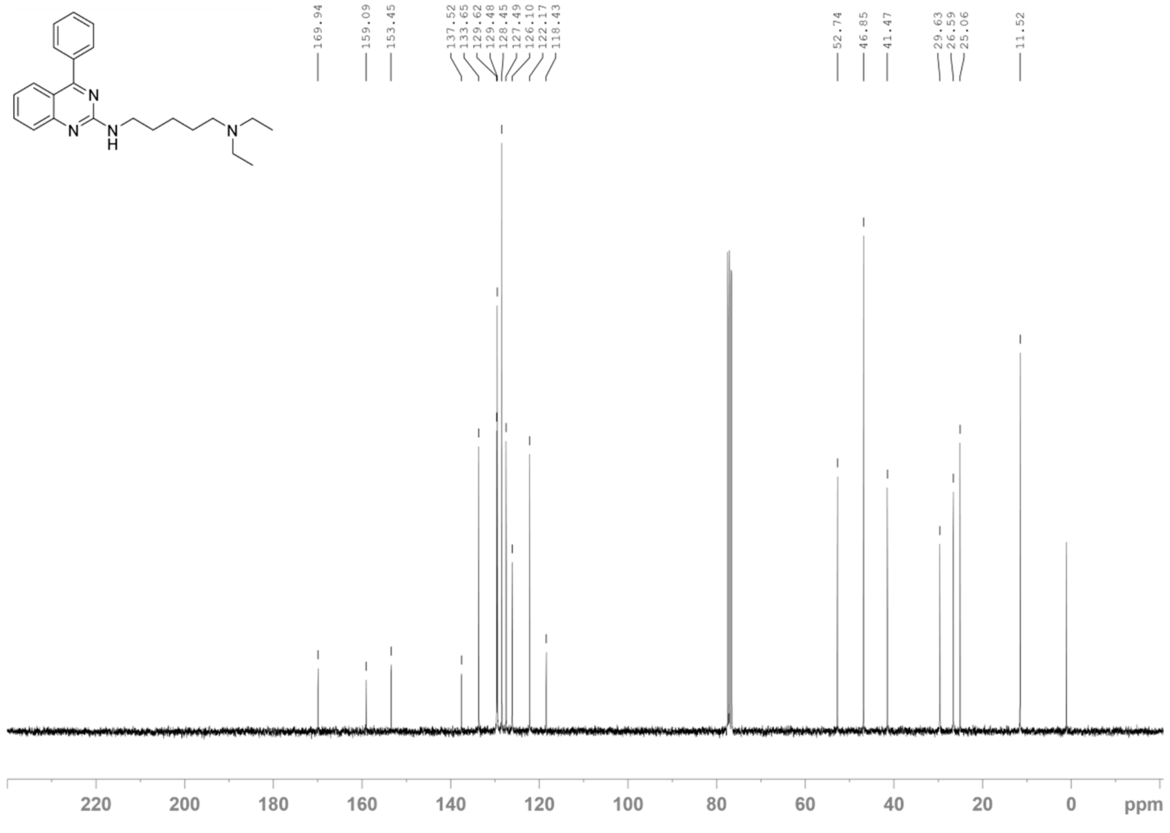

**LC-MS mass spectrum of compound 9p**

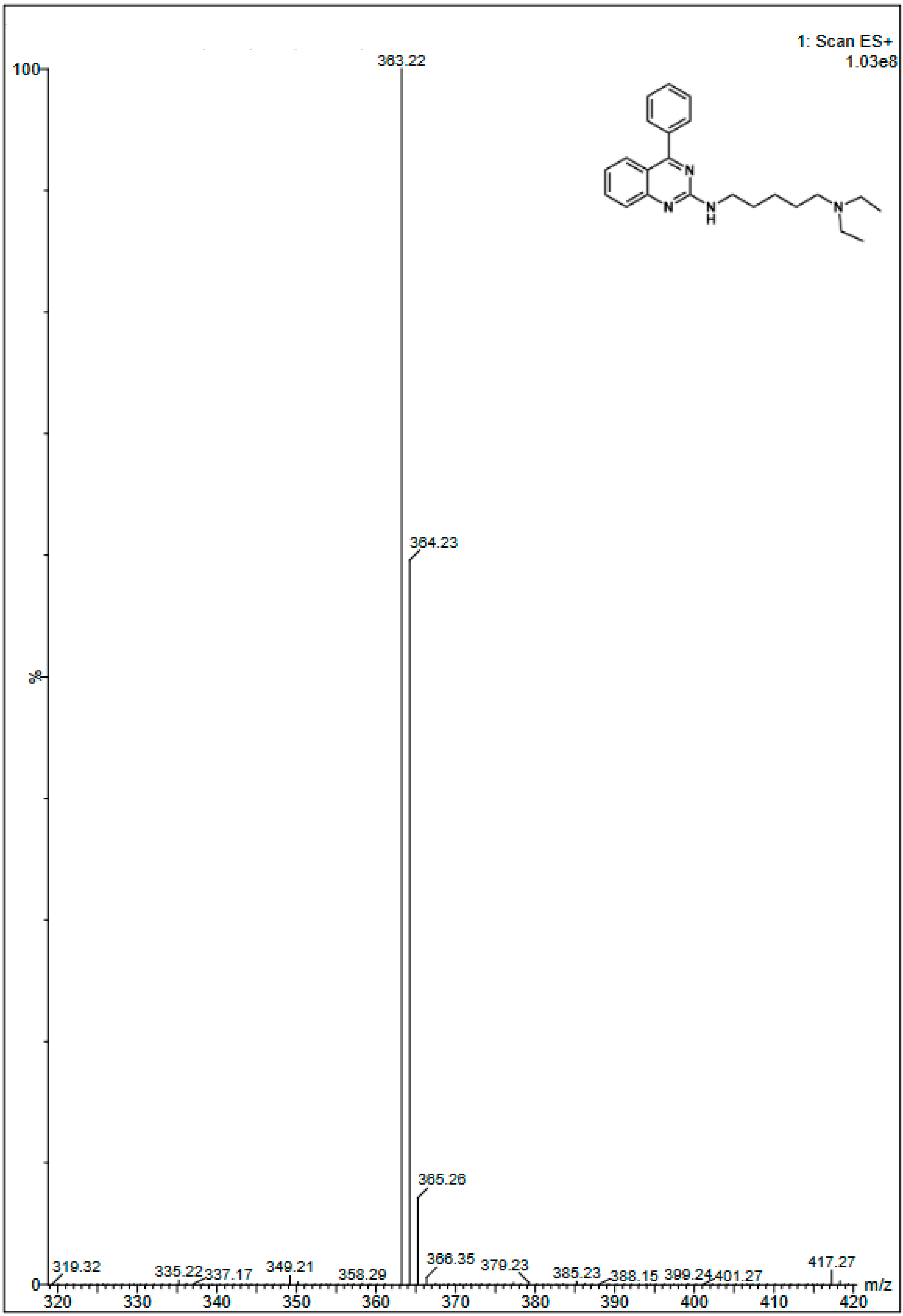

**1H- and 13C-NMR Spectra in $CDCl_3$ of compound 9q**

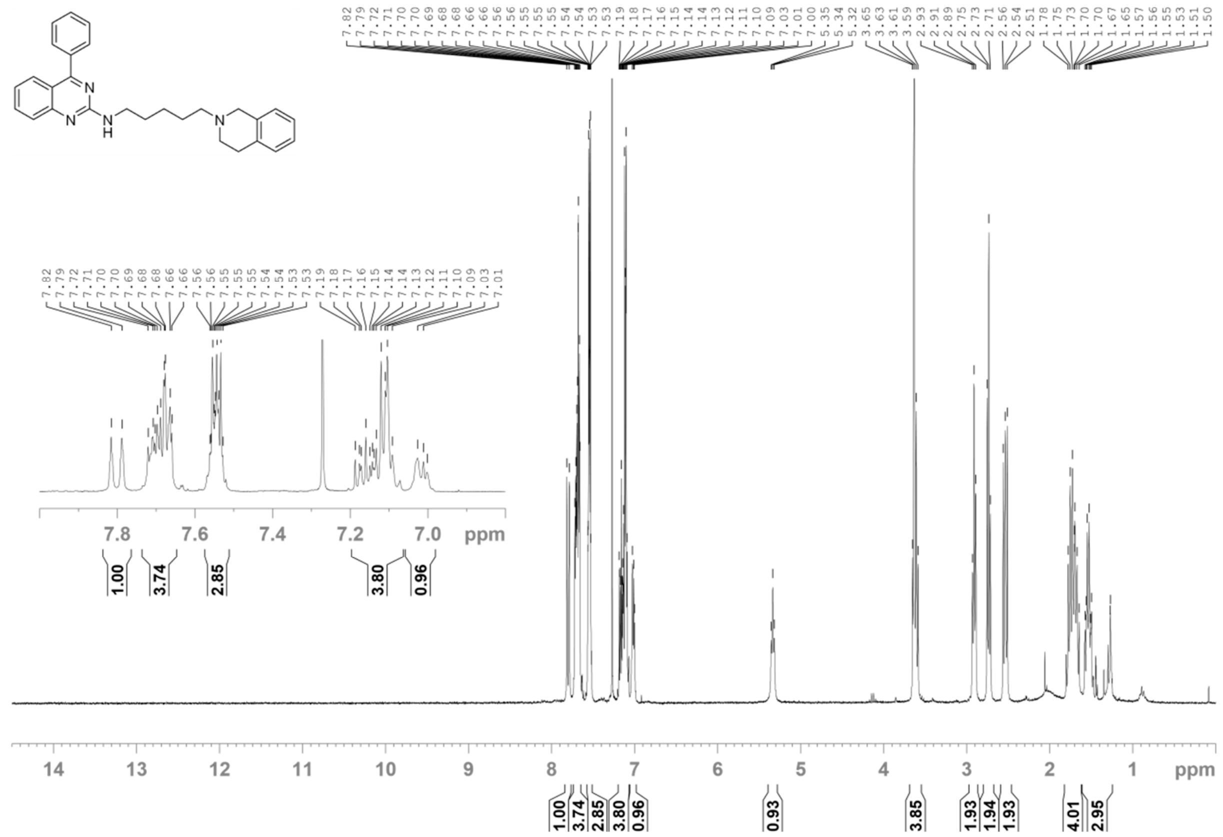

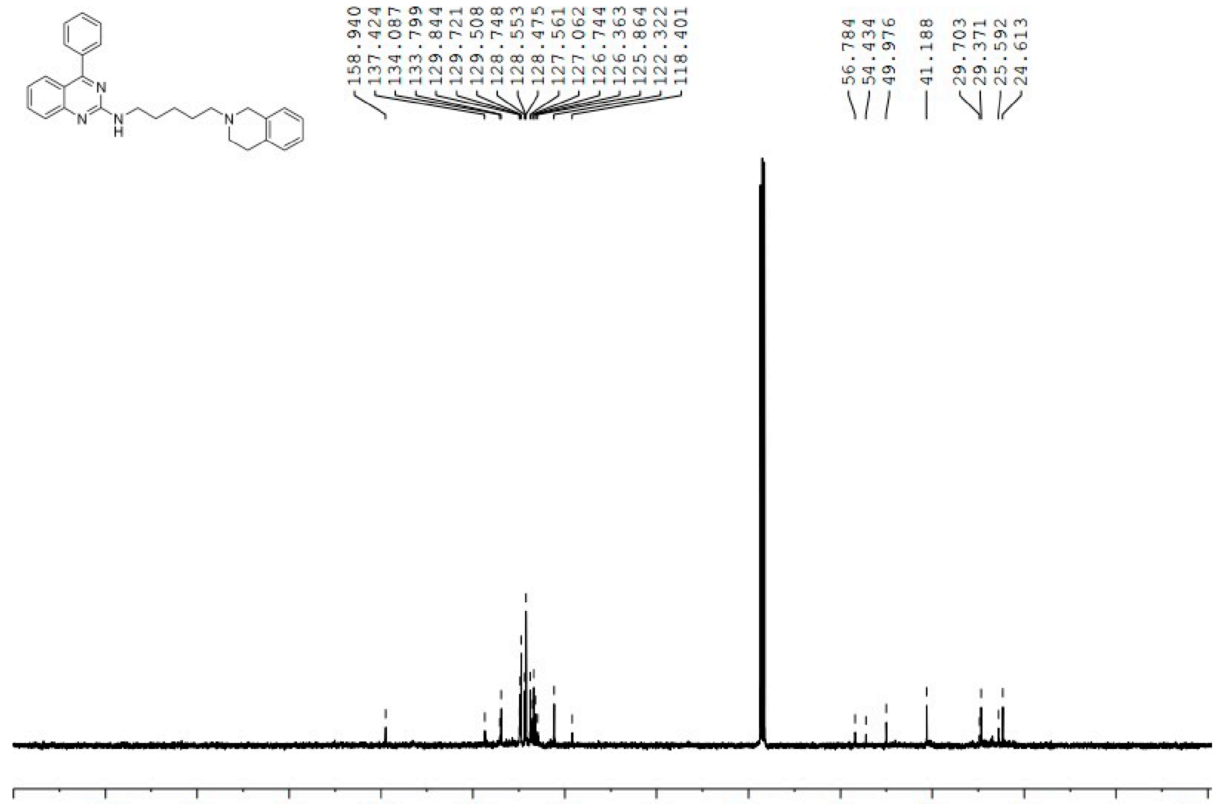

**LC-MS mass spectrum of compound 9q**

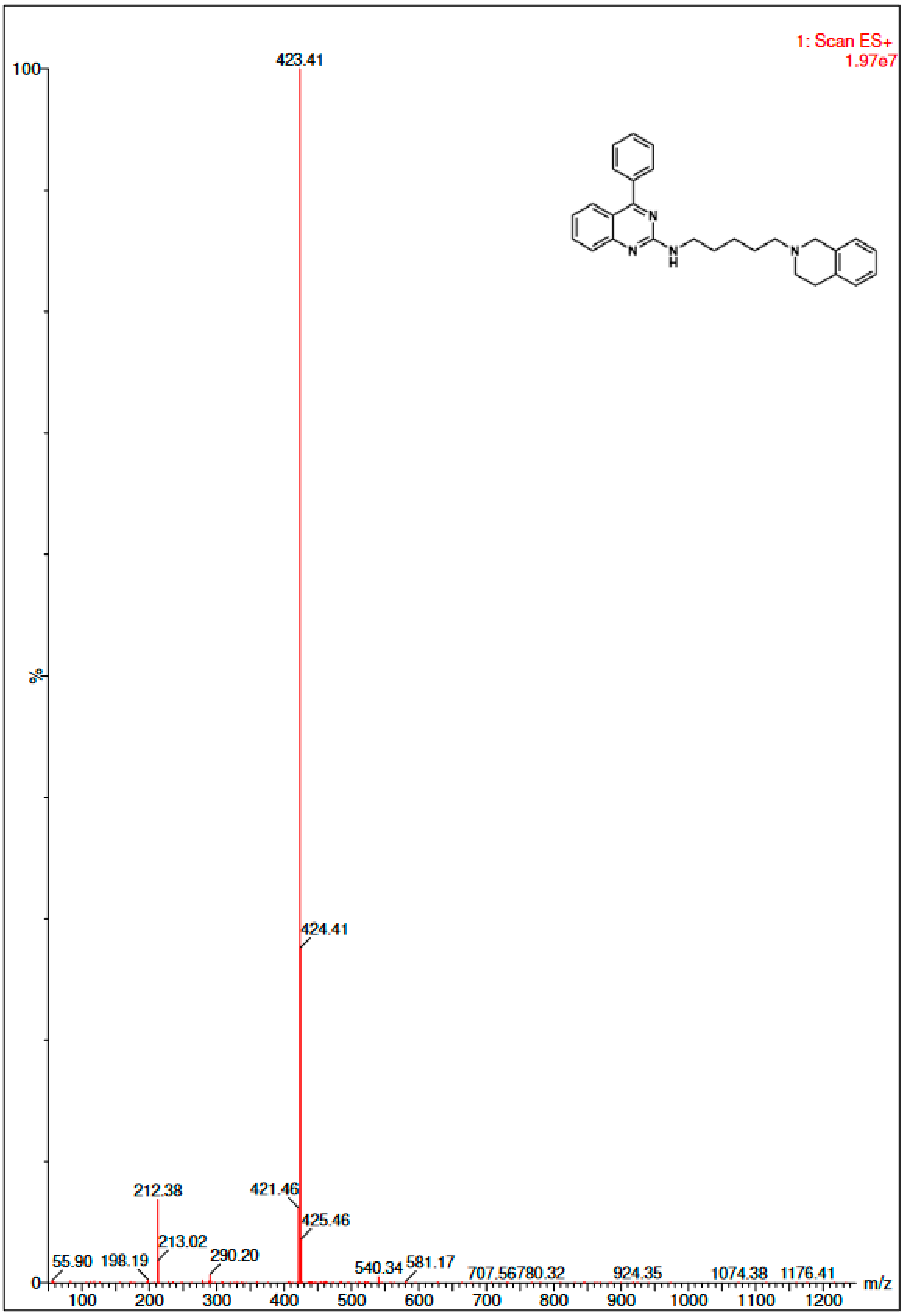

**1H- and 13C-NMR Spectra in $CDCl_3$ of compound 9r**

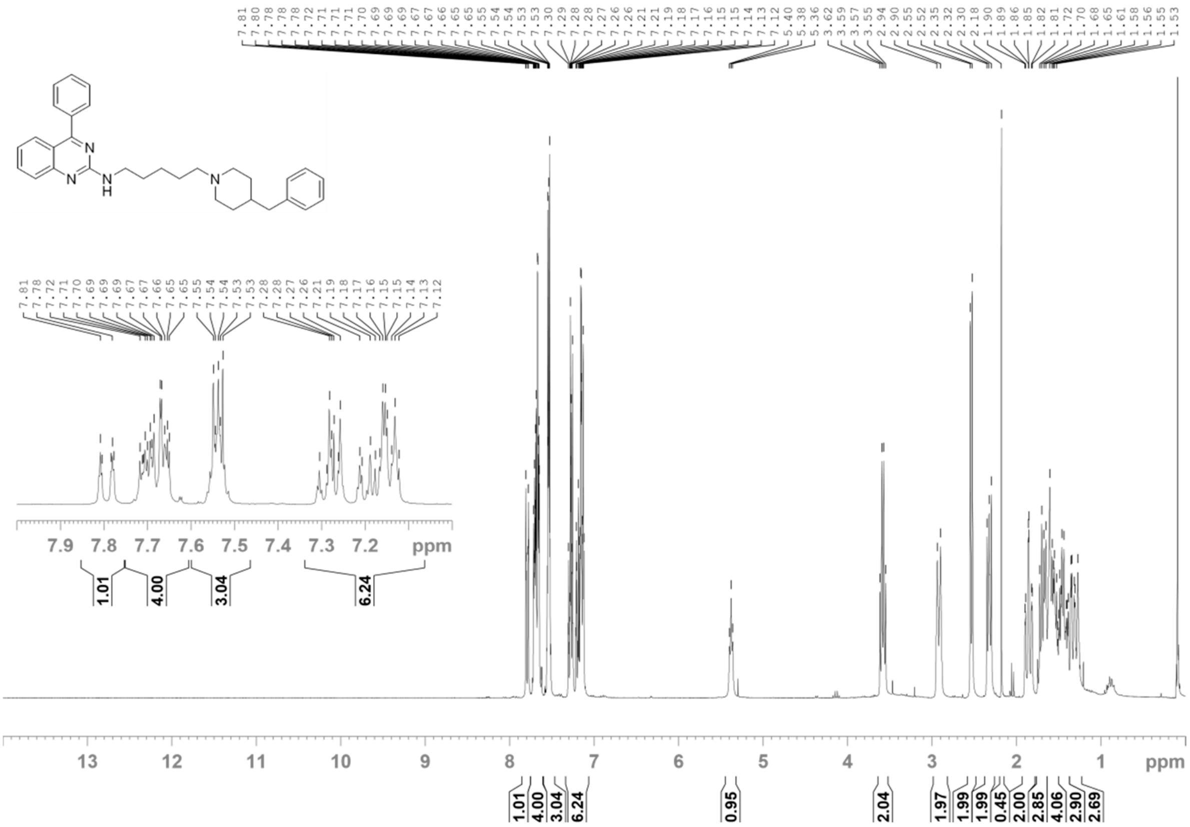


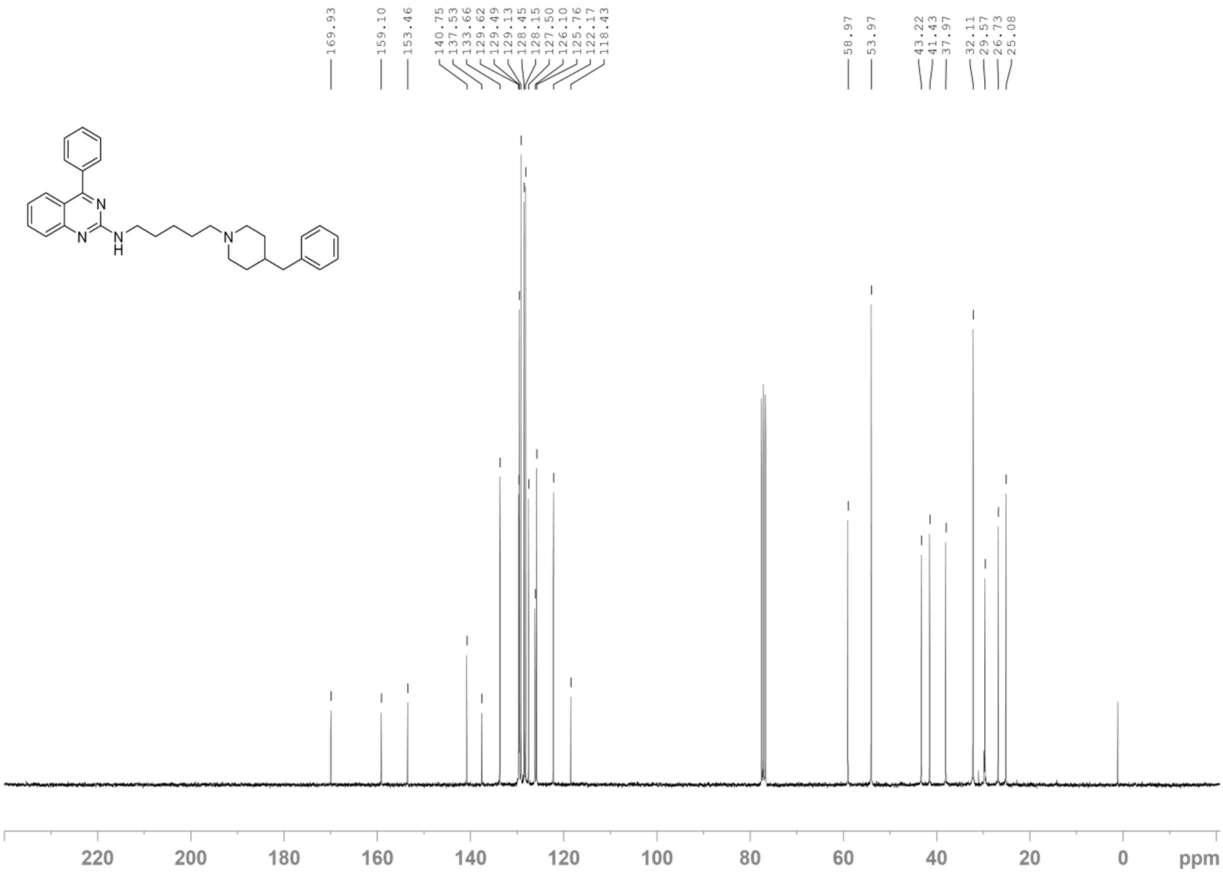

**LC-MS mass spectrum of compound 9r**

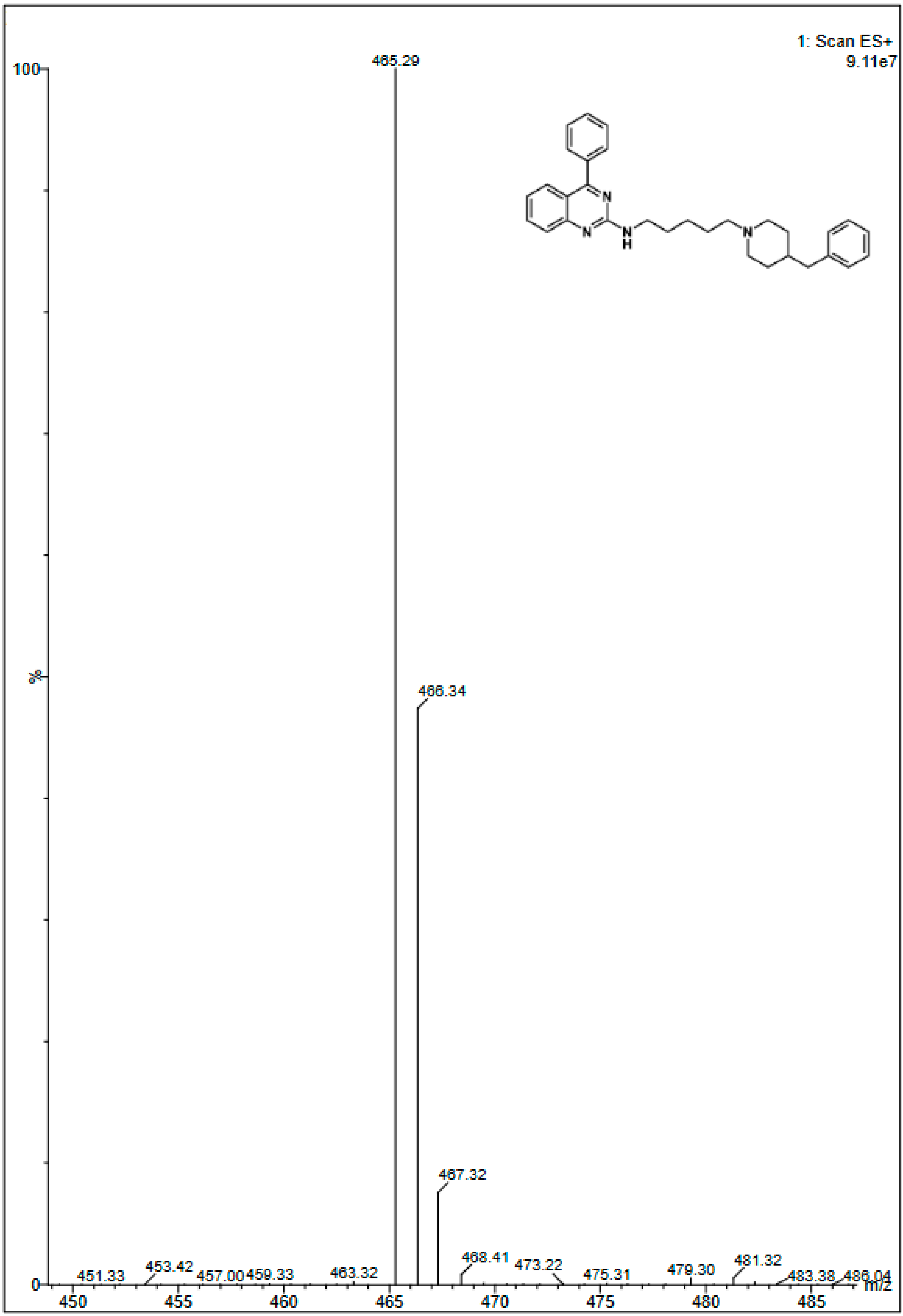

**1H- and 13C-NMR Spectra in $CDCl_3$ of compound 9s**

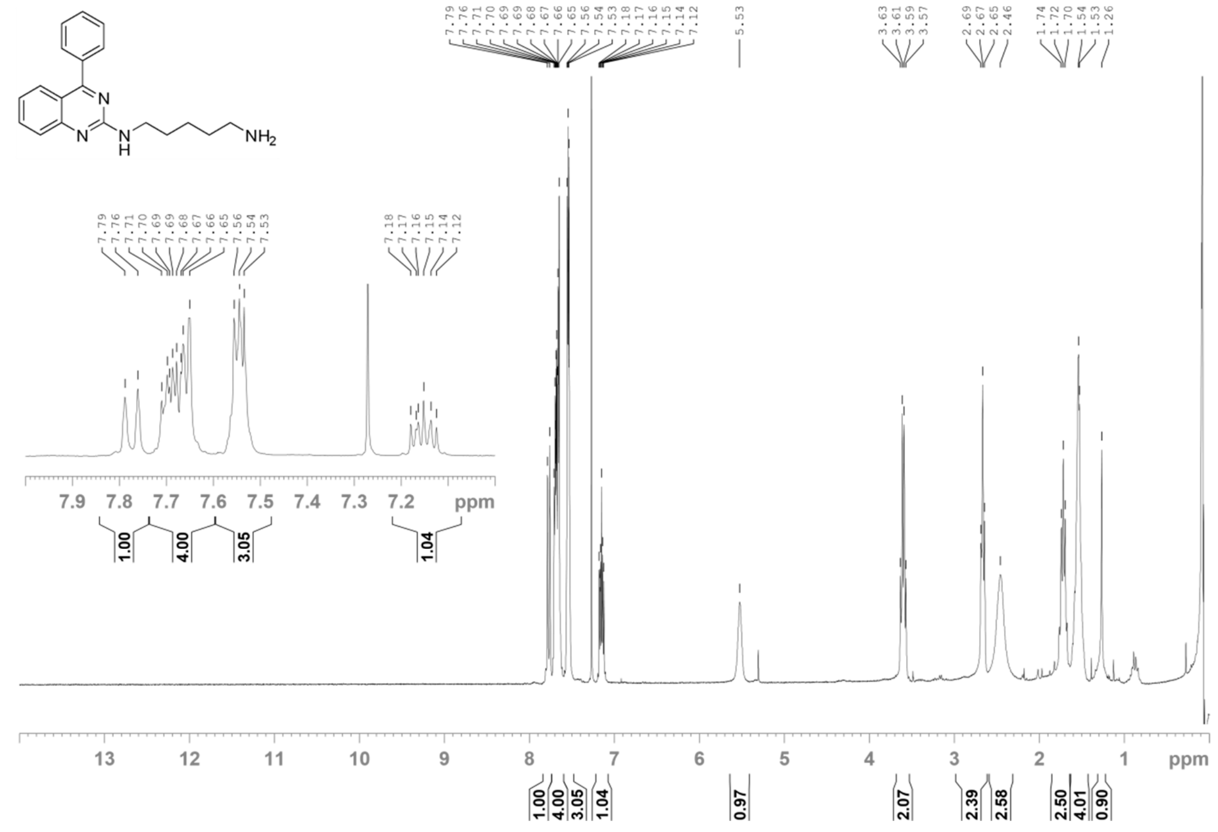

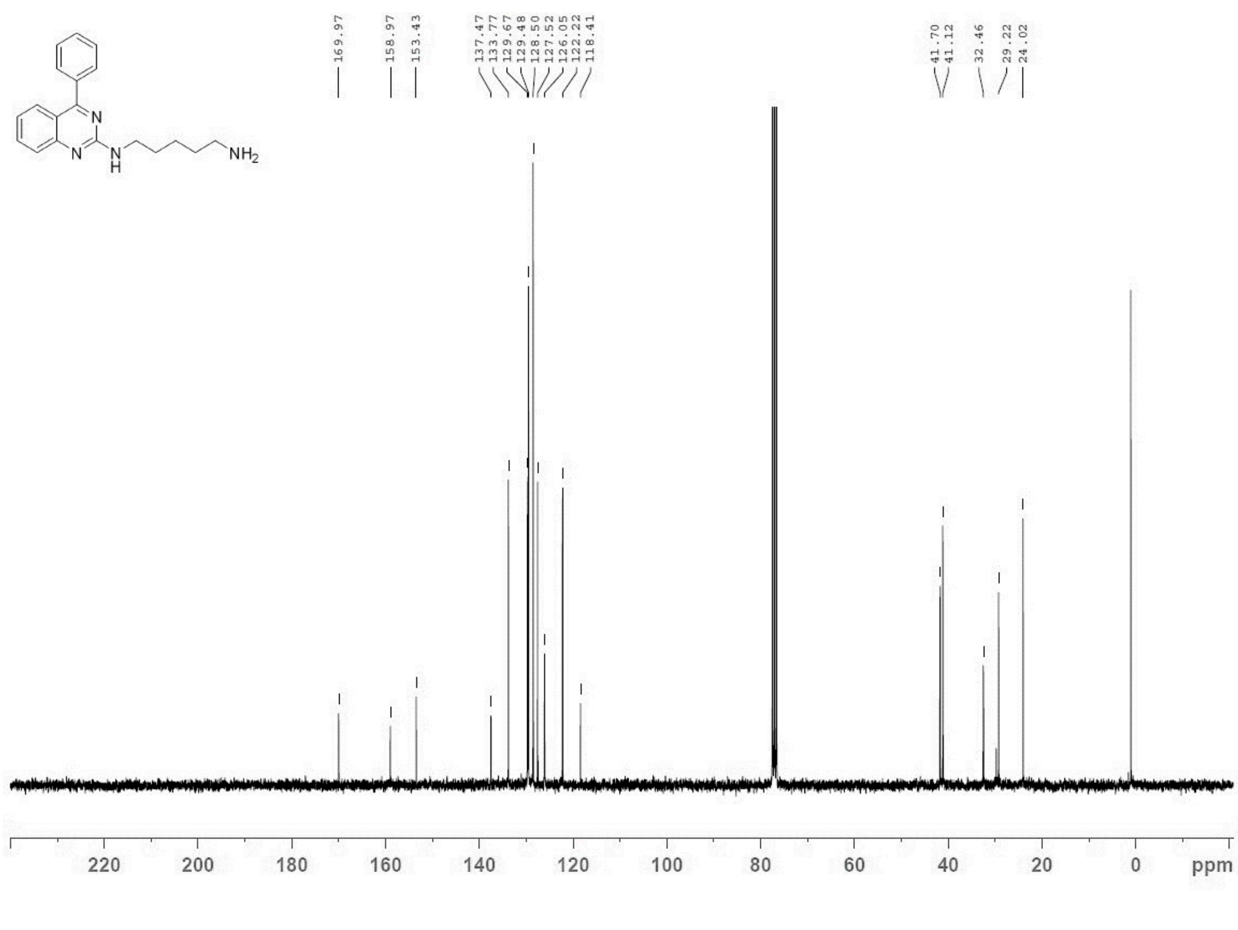

**LC-MS mass spectrum of compound 9s**

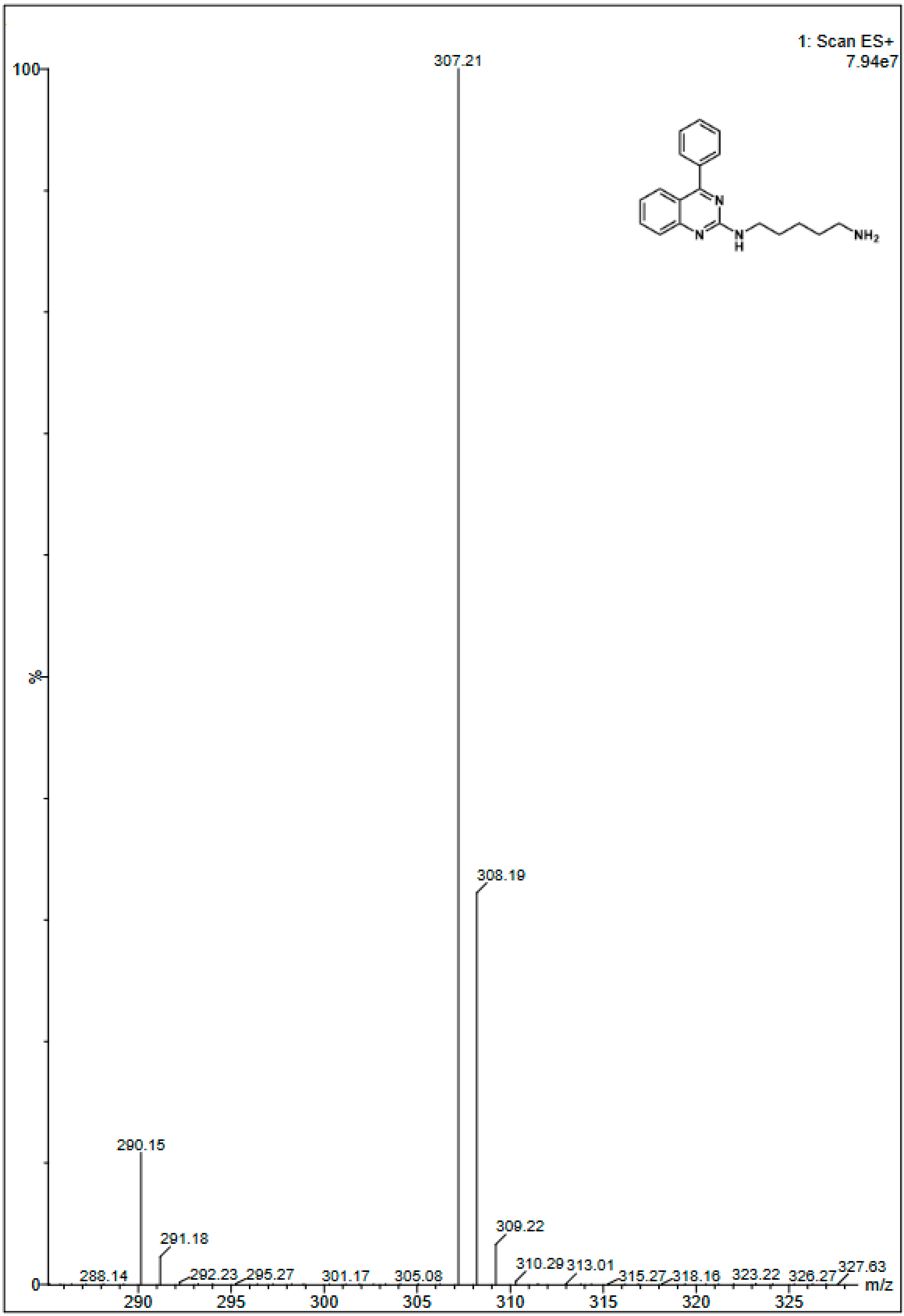

**1H- and 13C-NMR Spectra in $CDCl_3$ of compound 9t**

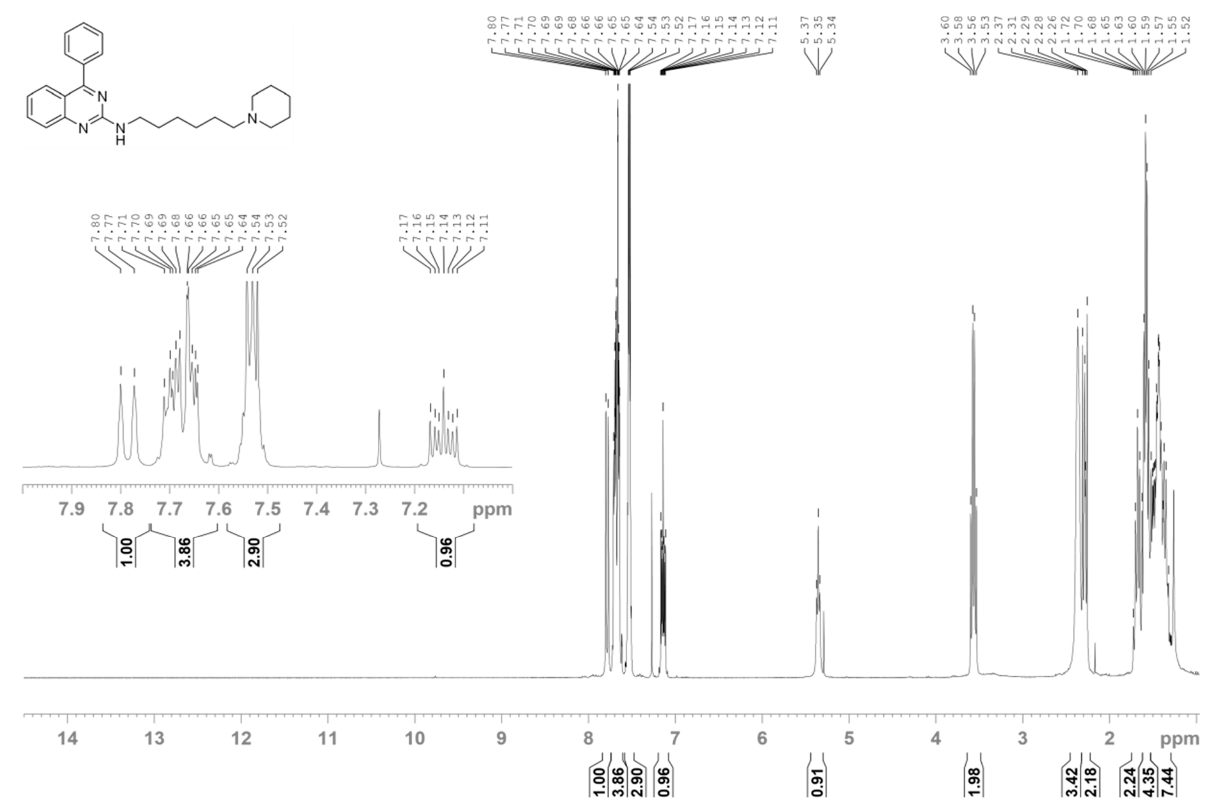


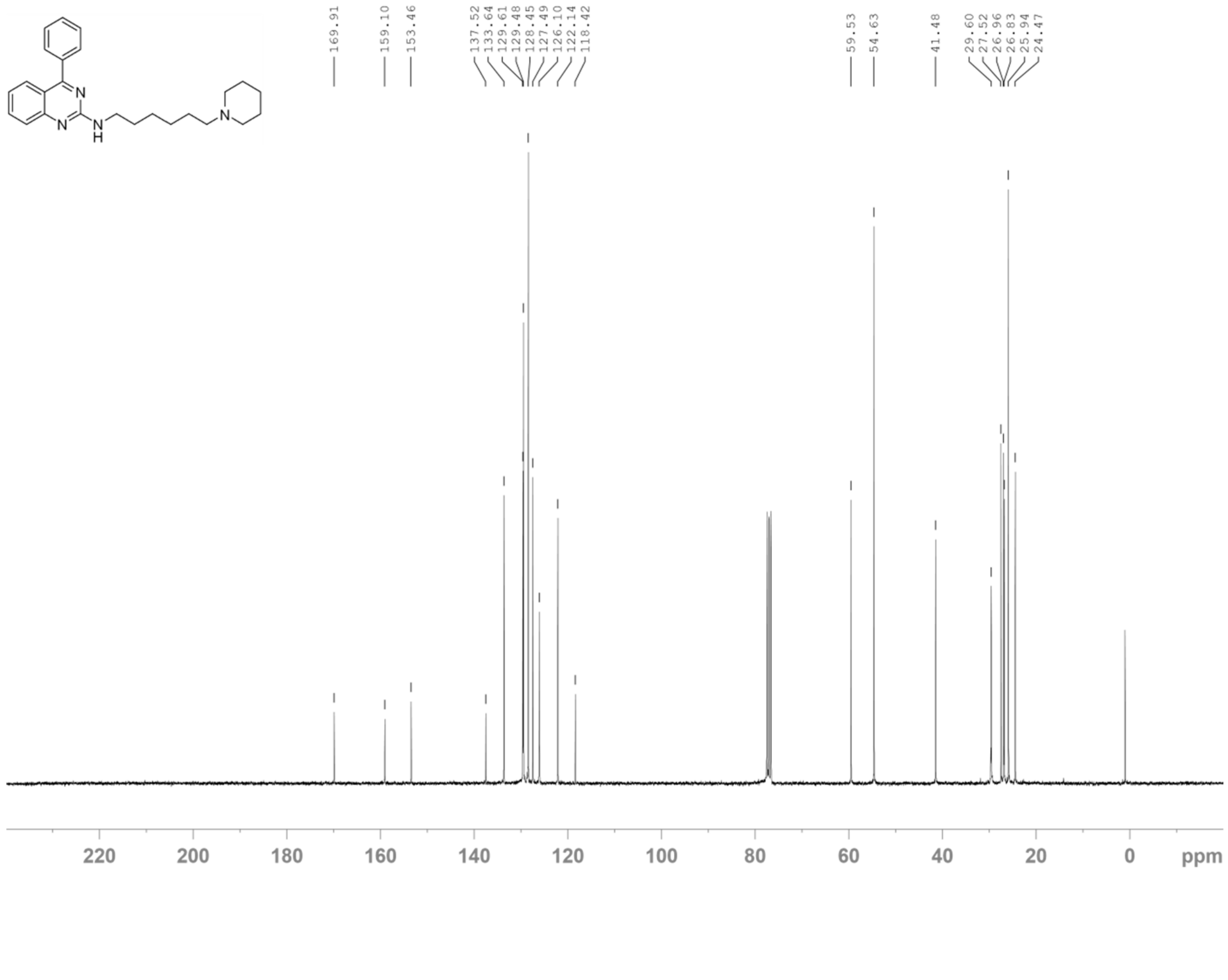

**LC-MS mass spectrum of compound 9t**

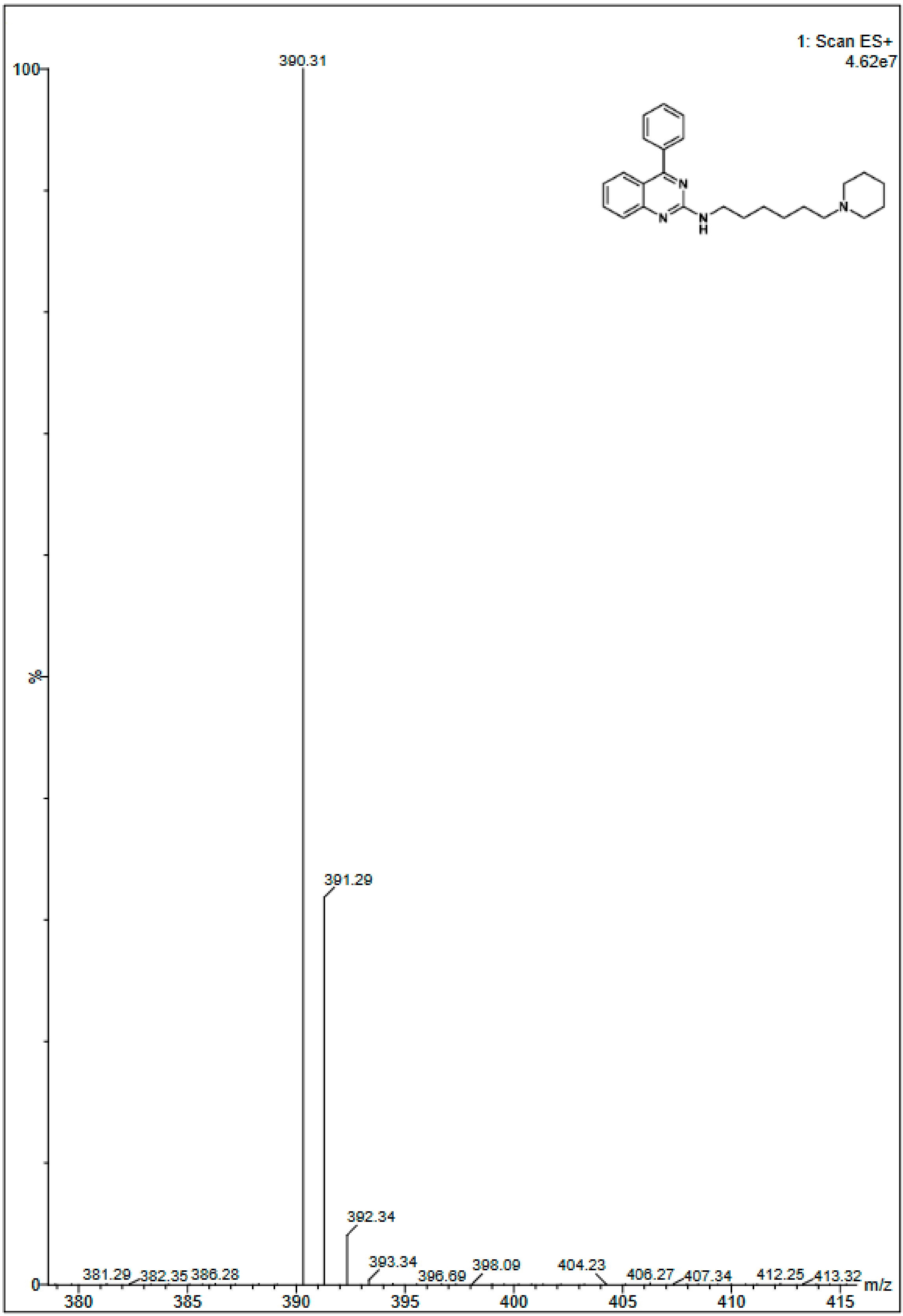

**1H- and 13C-NMR Spectra in CDCl$_3$ of compound 9u**

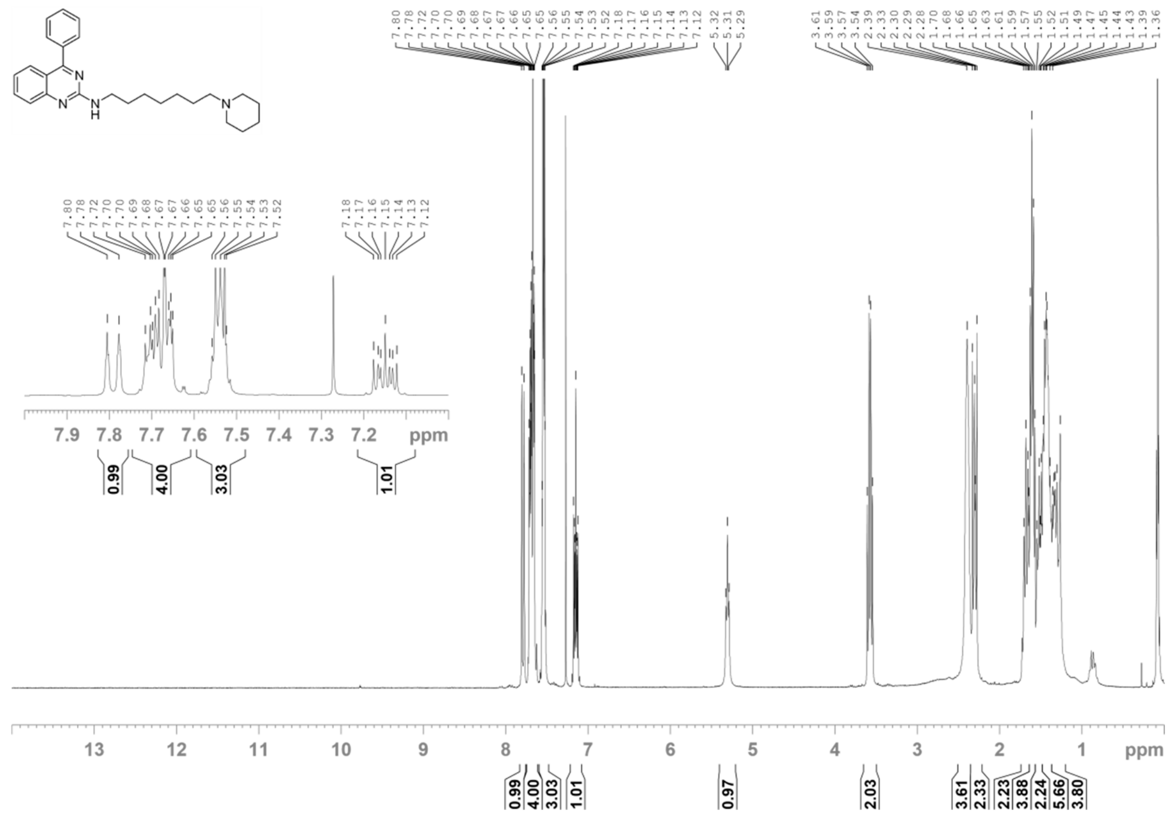

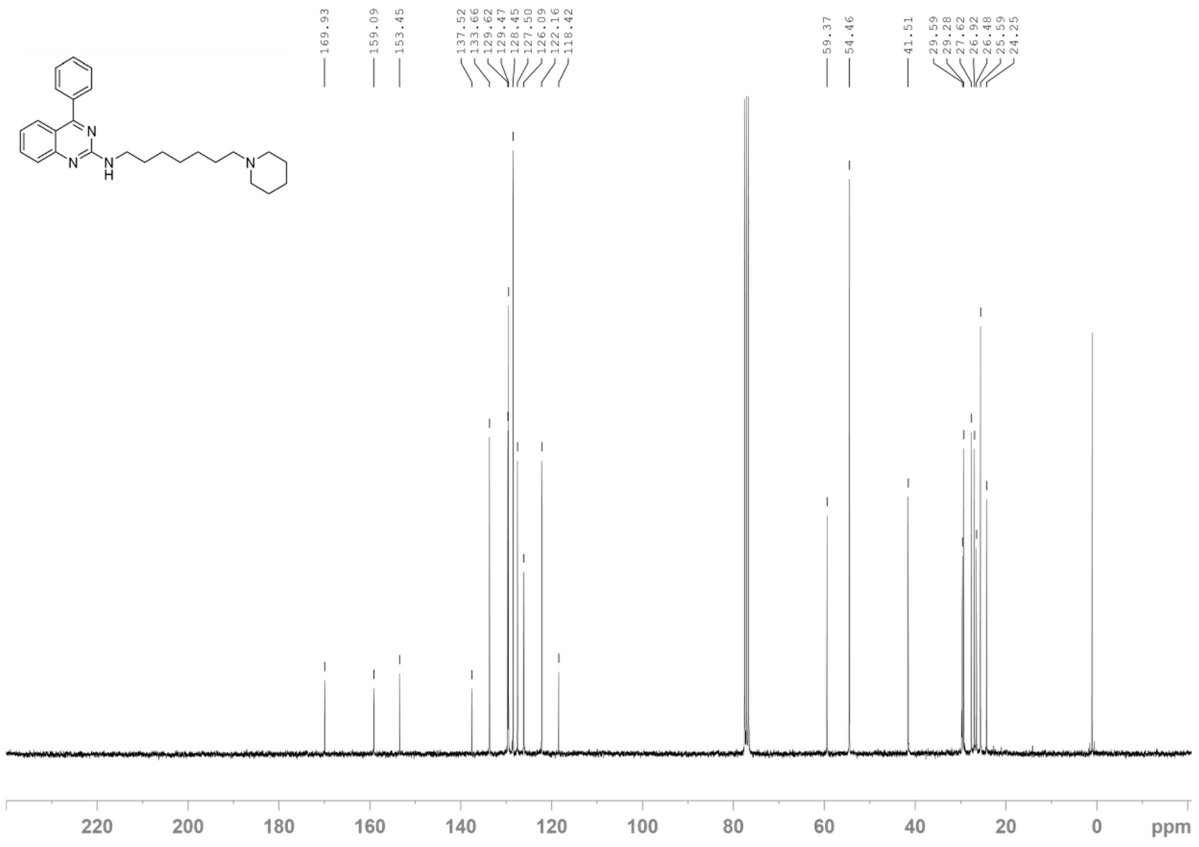

**LC-MS mass spectrum of compound 9u**

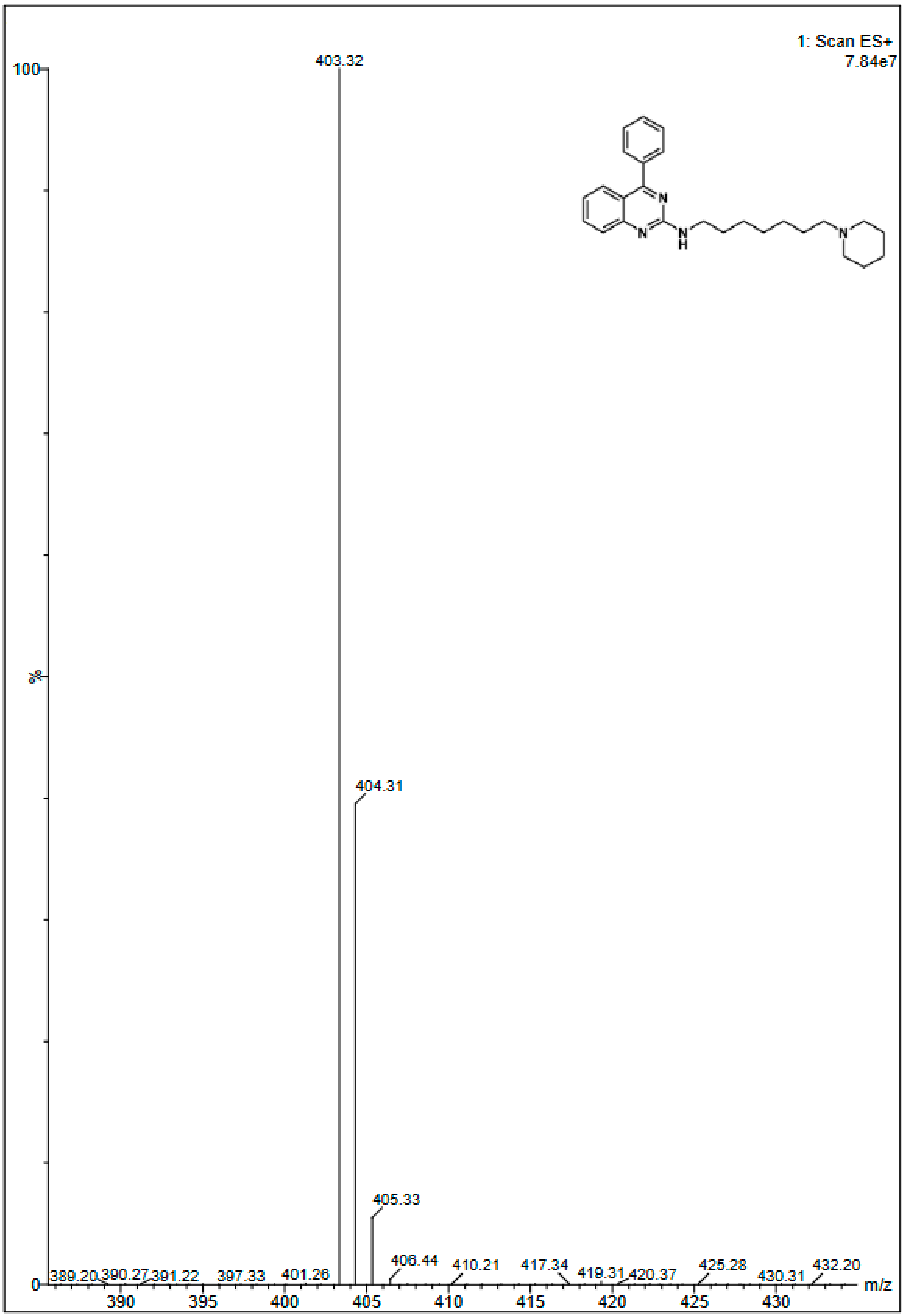

**1H- and 13C-NMR Spectra in $CDCl_3$ of compound 9v**

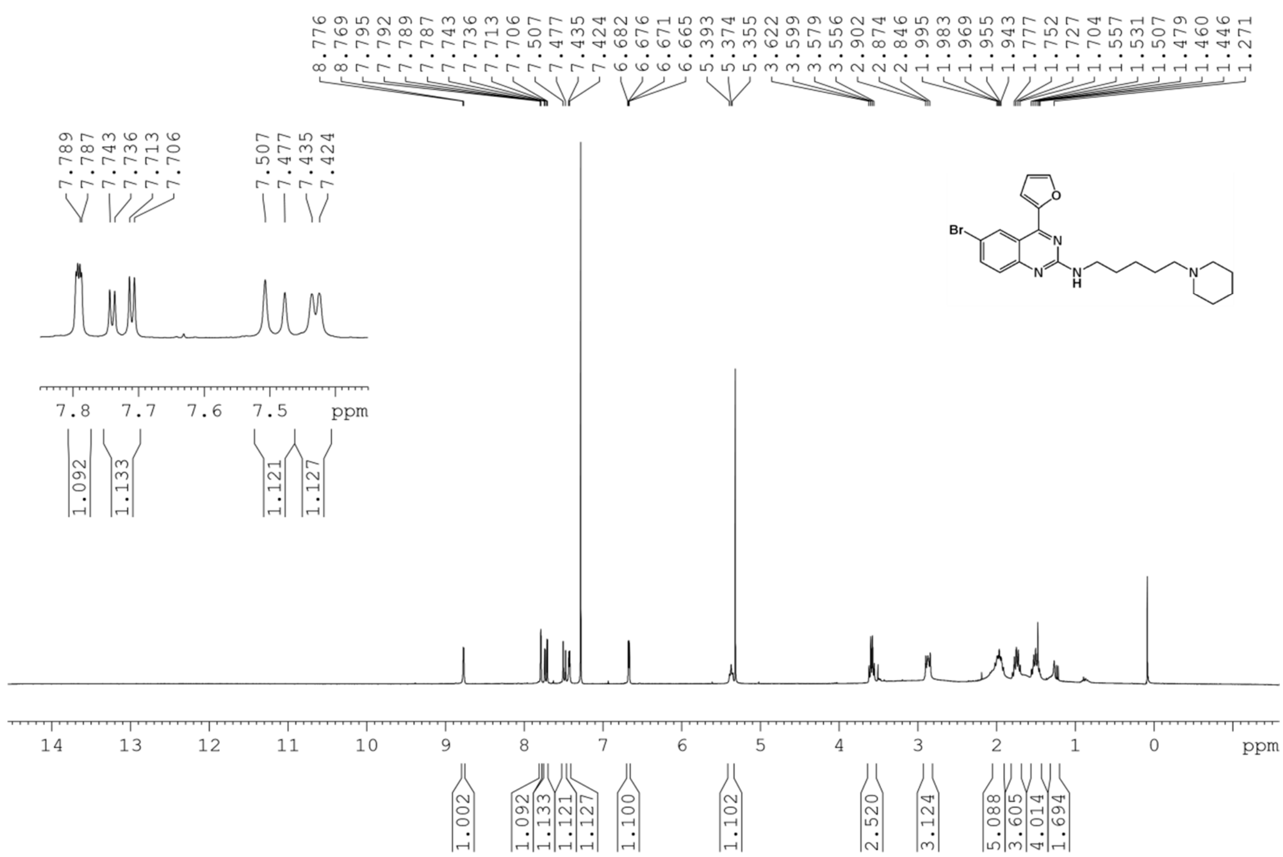


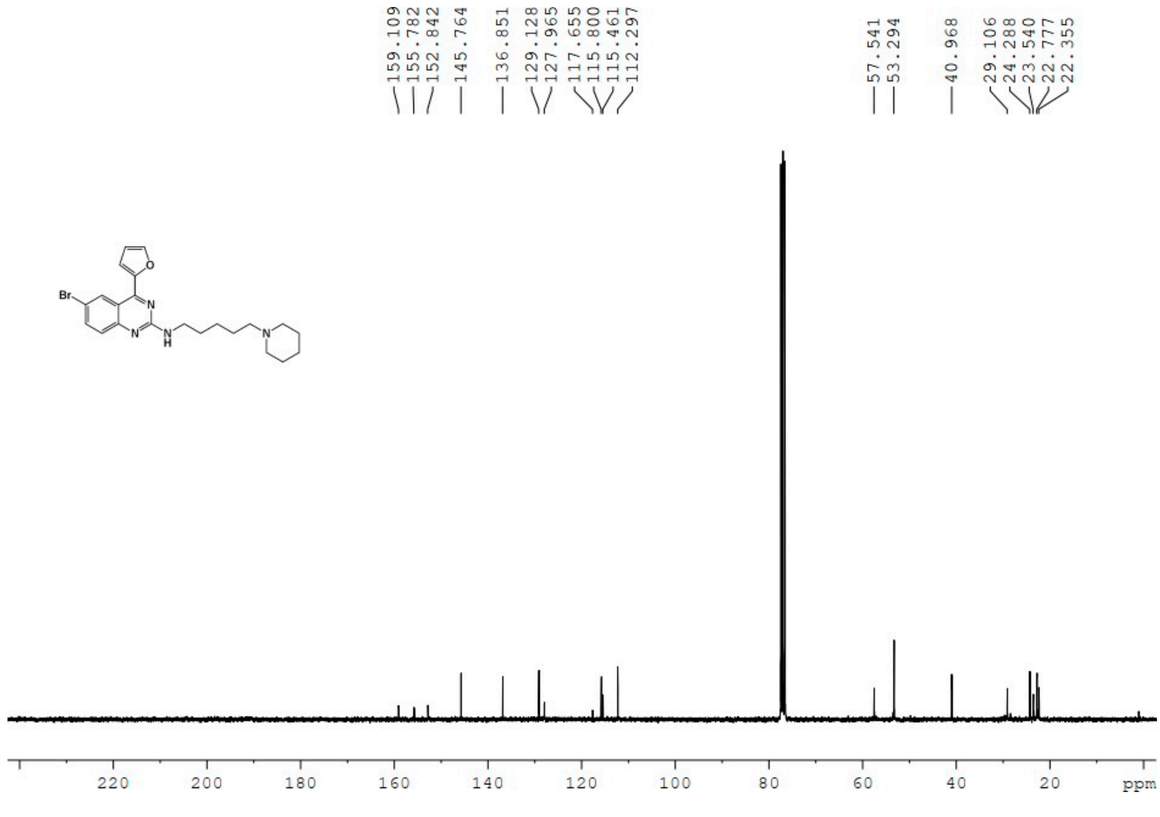

**LC-MS mass spectrum of compound 9v**

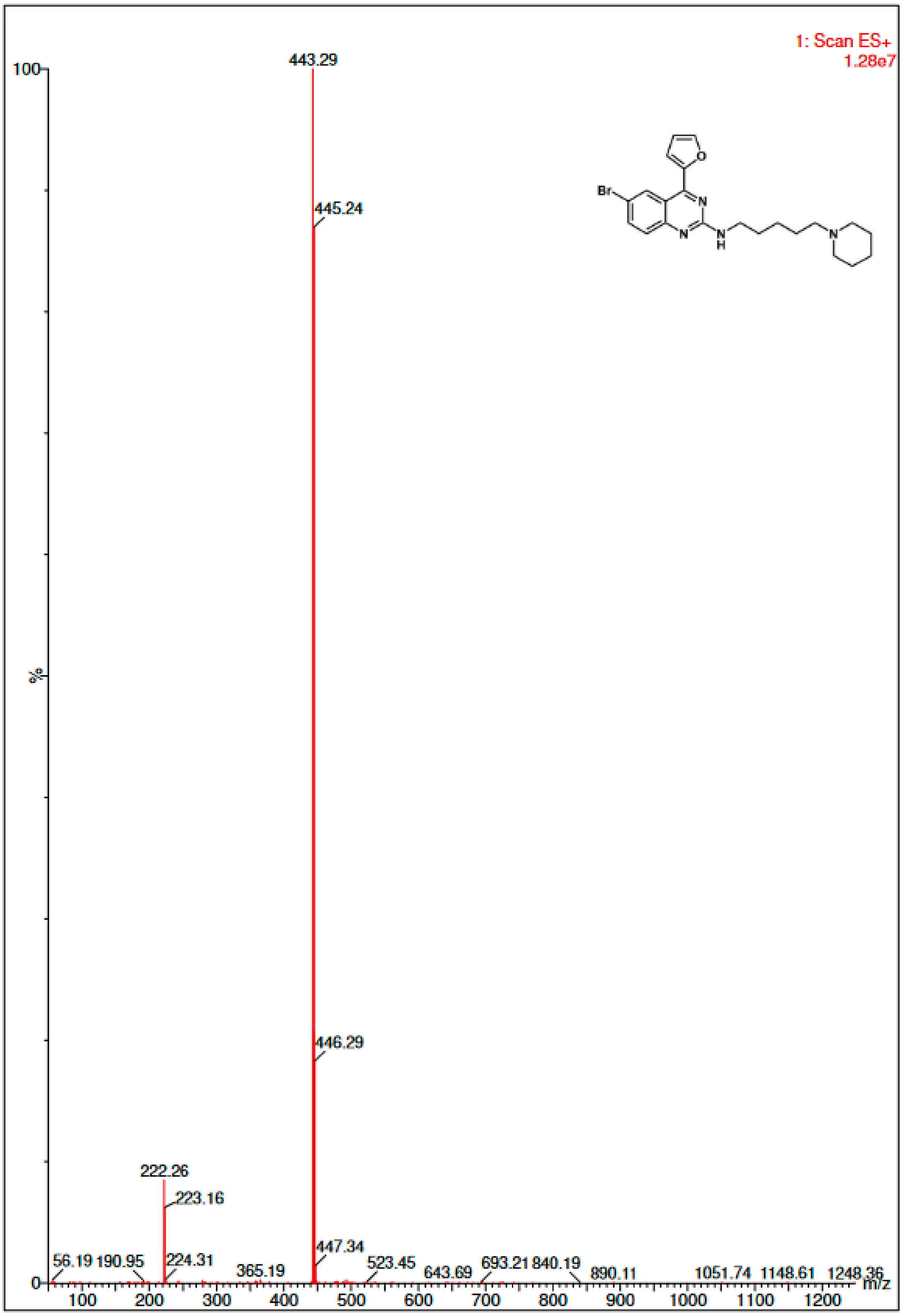

**1H- and 13C-NMR Spectra in $(CD_3)_2CO$ of compound 9w**

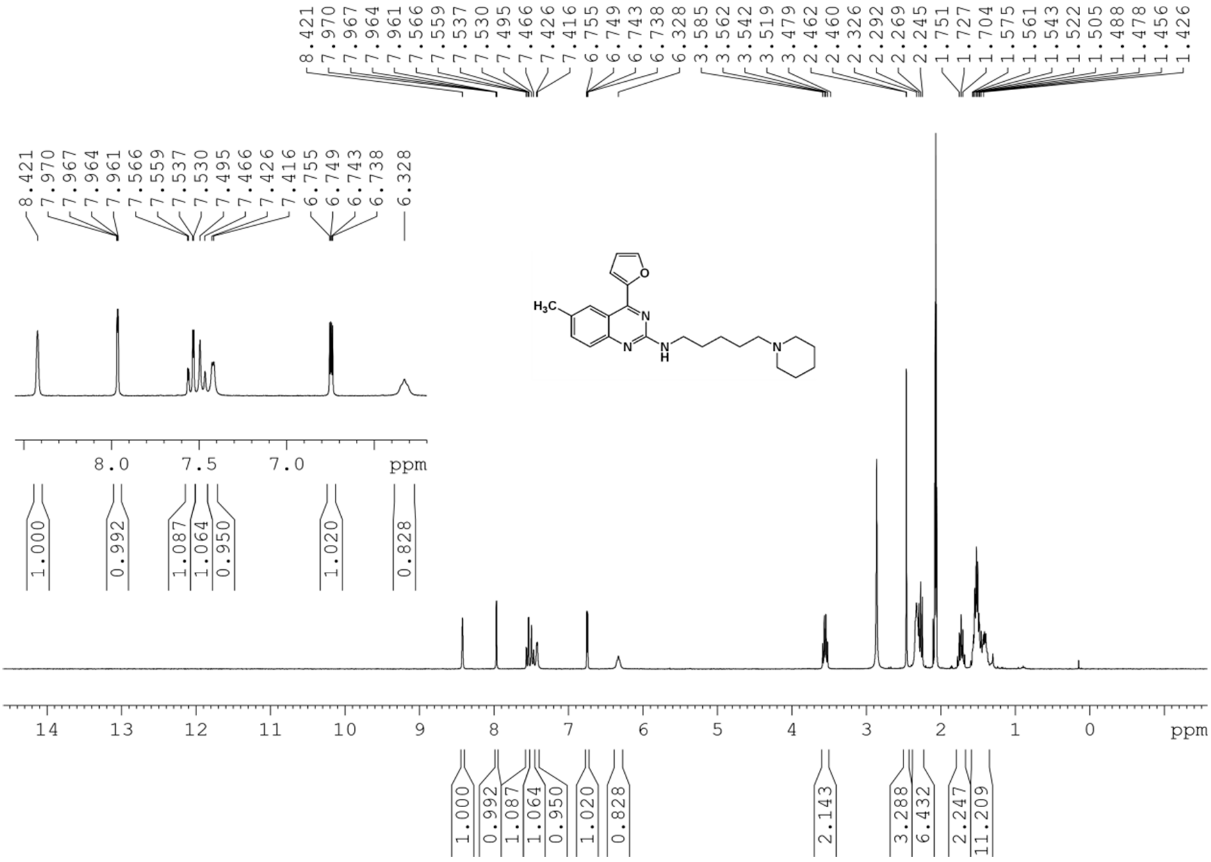

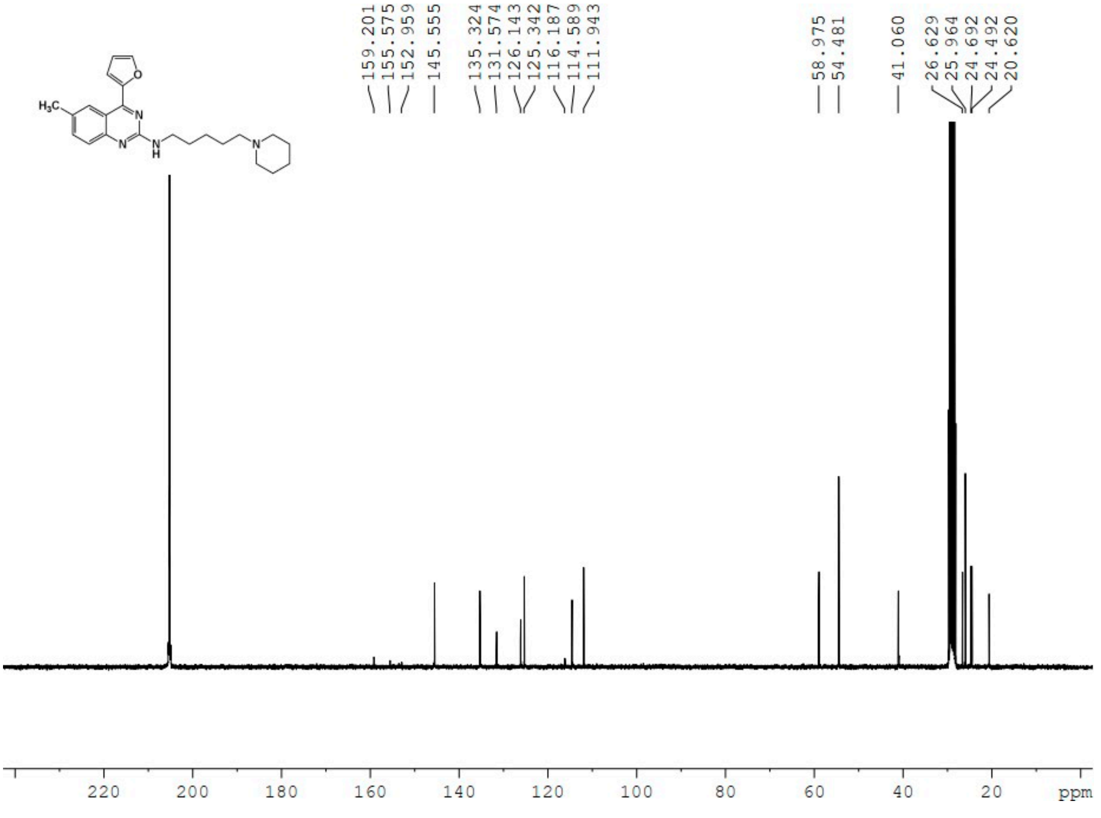

**LC-MS mass spectrum of compound 9w**

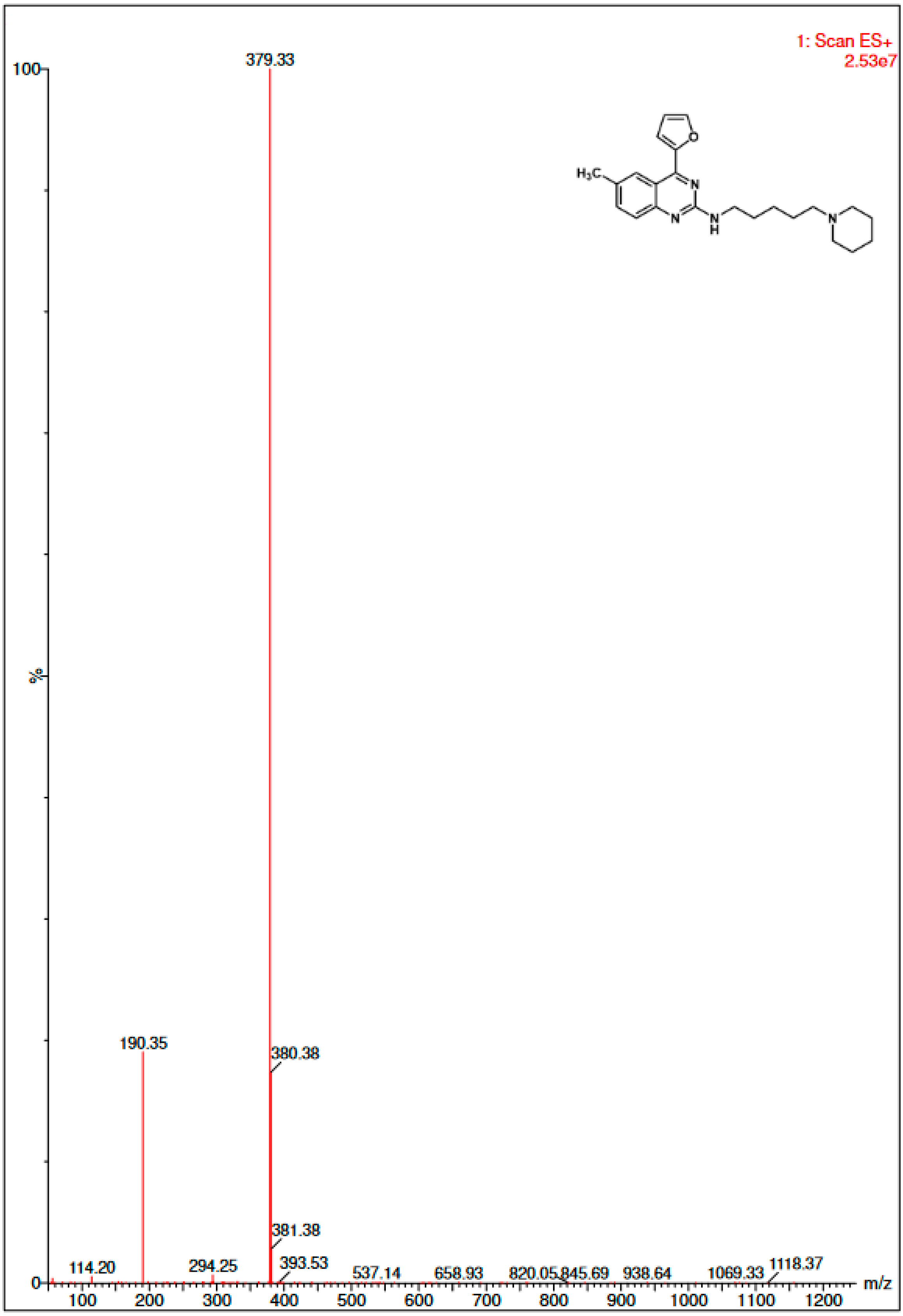

**1H- and 13C-NMR Spectra in CDCl$_3$ of compound 9x**

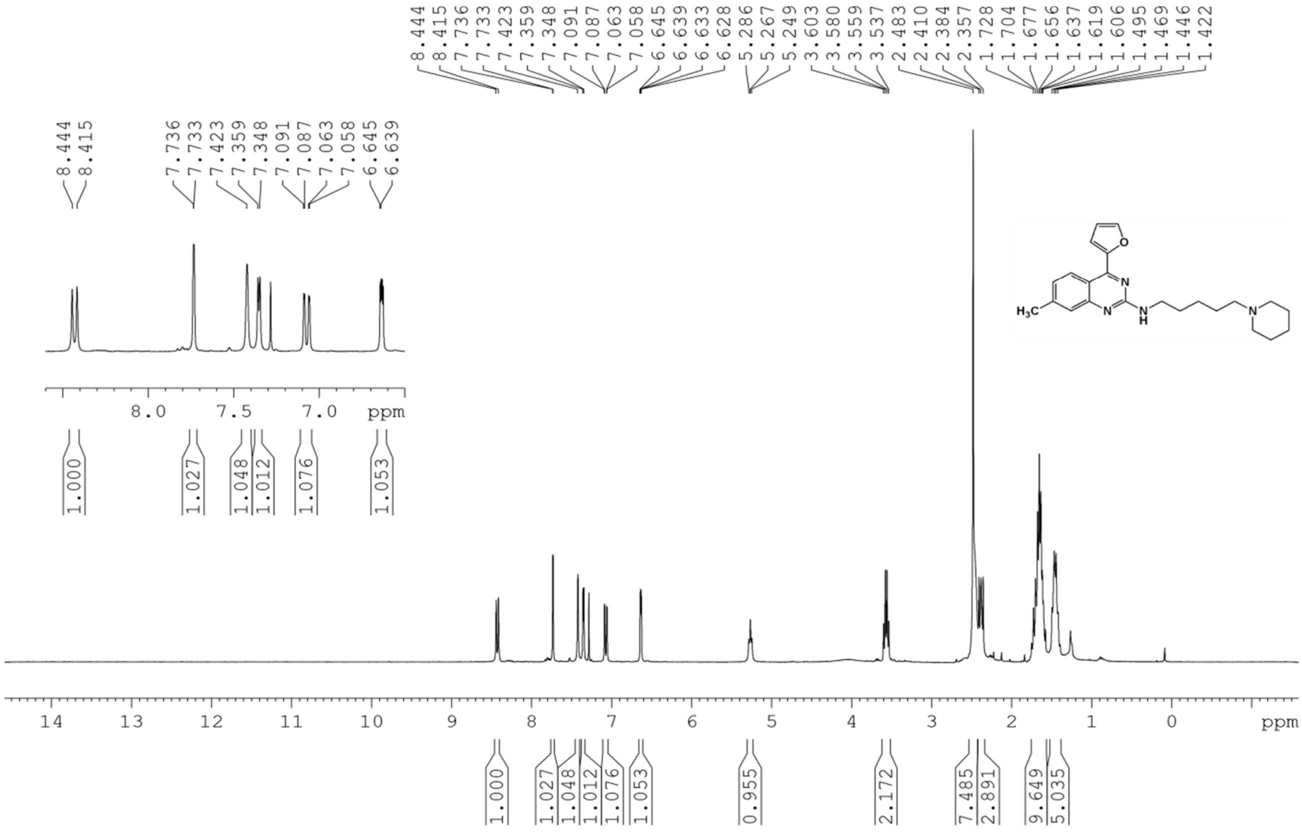


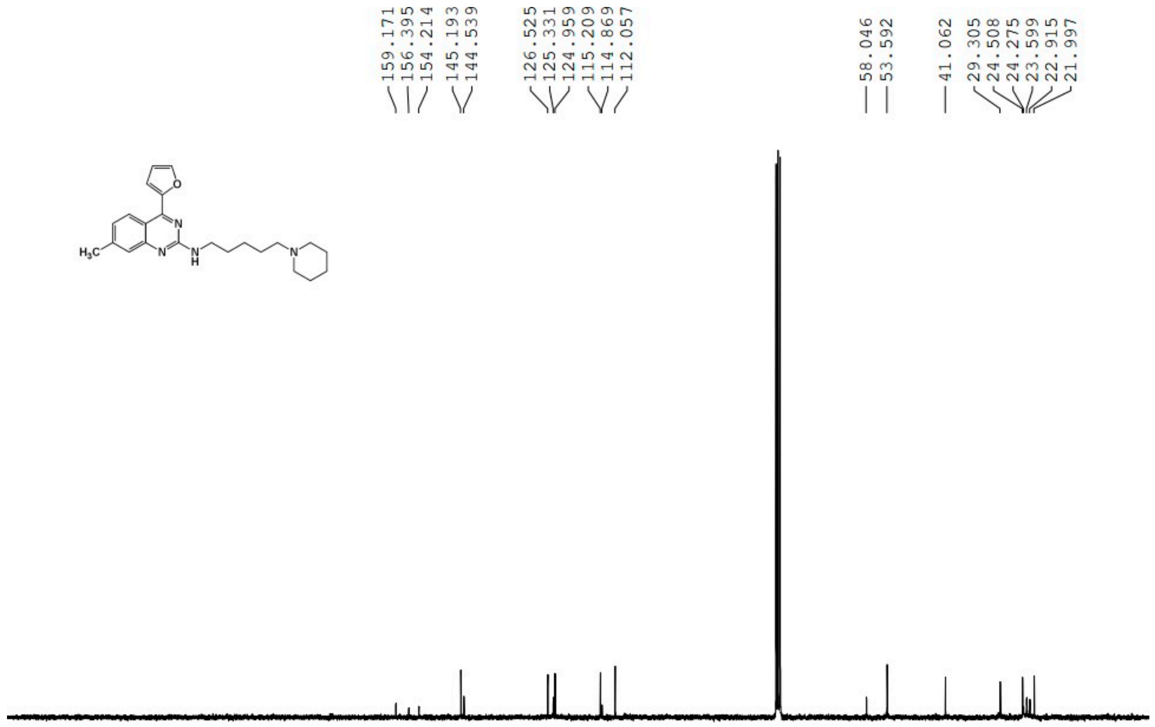

**LC-MS mass spectrum of compound 9x**

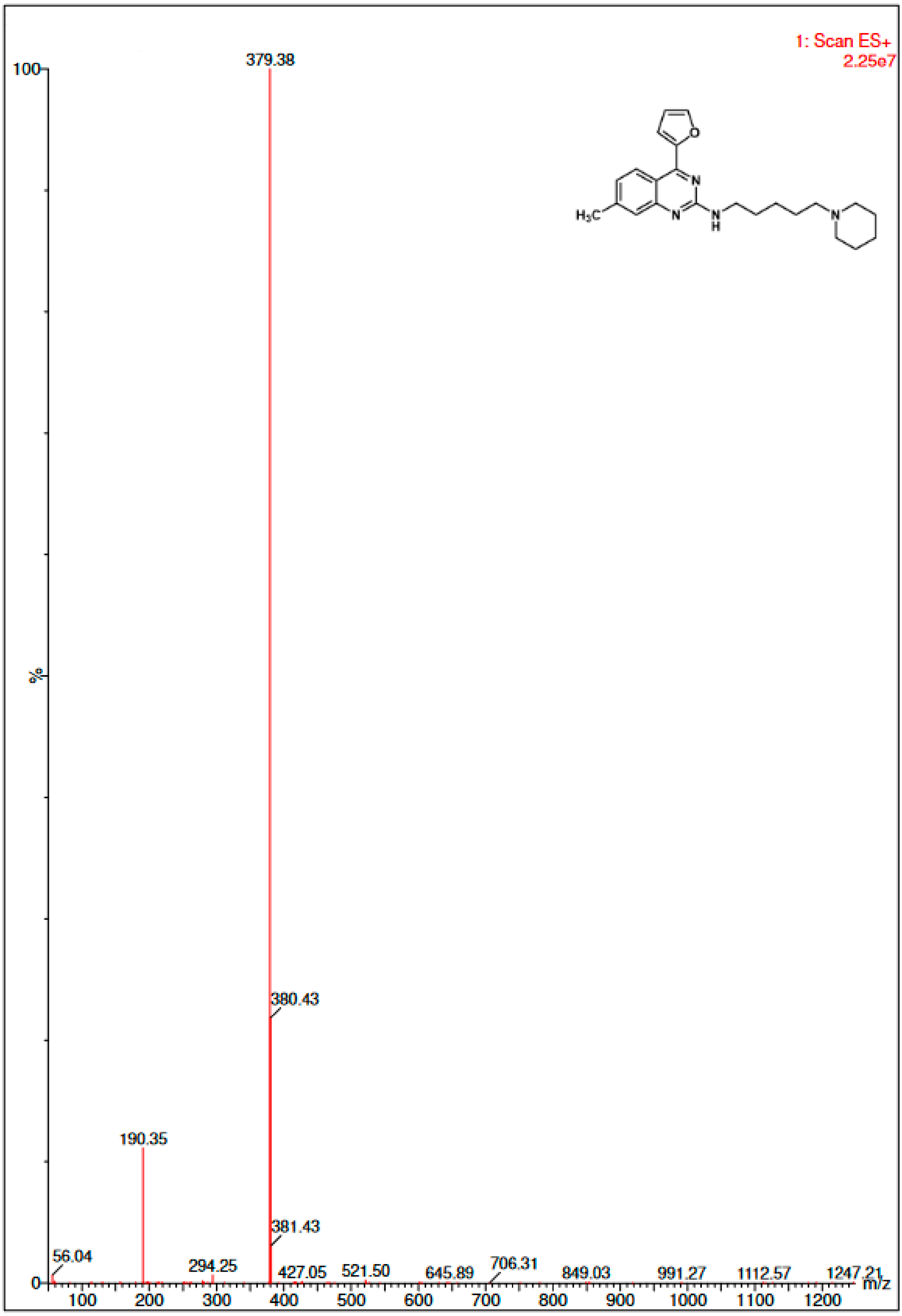

**1H-NMR Spectra in DMSO-d6 and 13C-NMR Spectra in $CDCl_3$ of compound 10a**

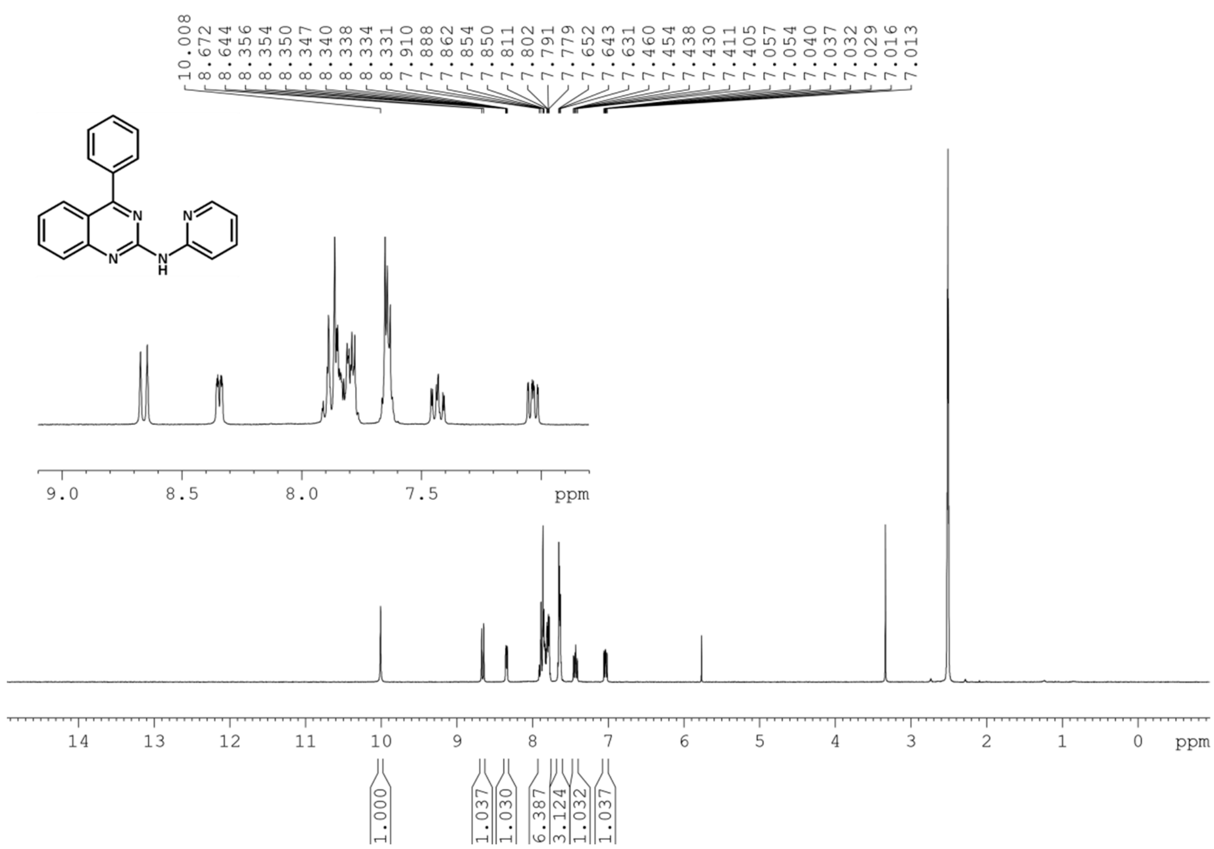


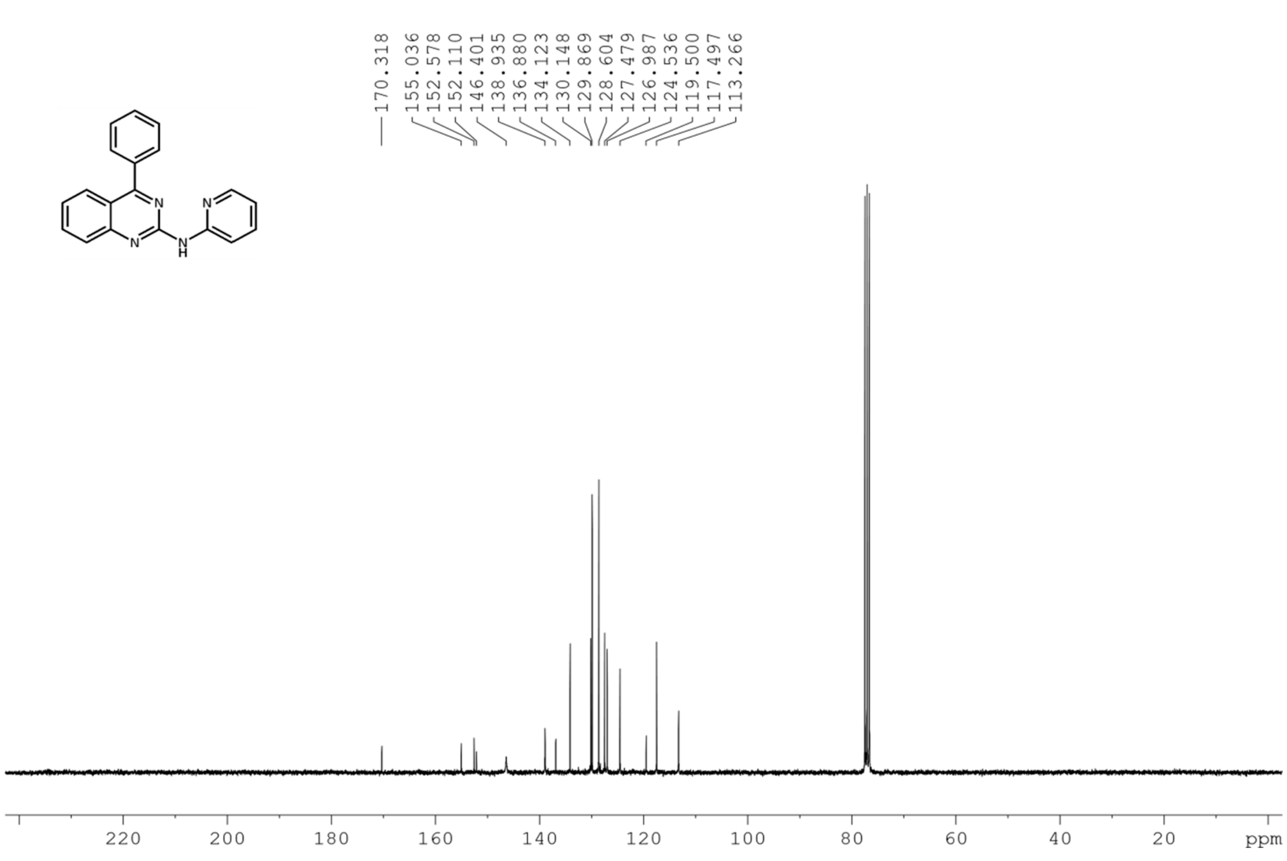

**LC-MS mass spectrum of compound 10a**

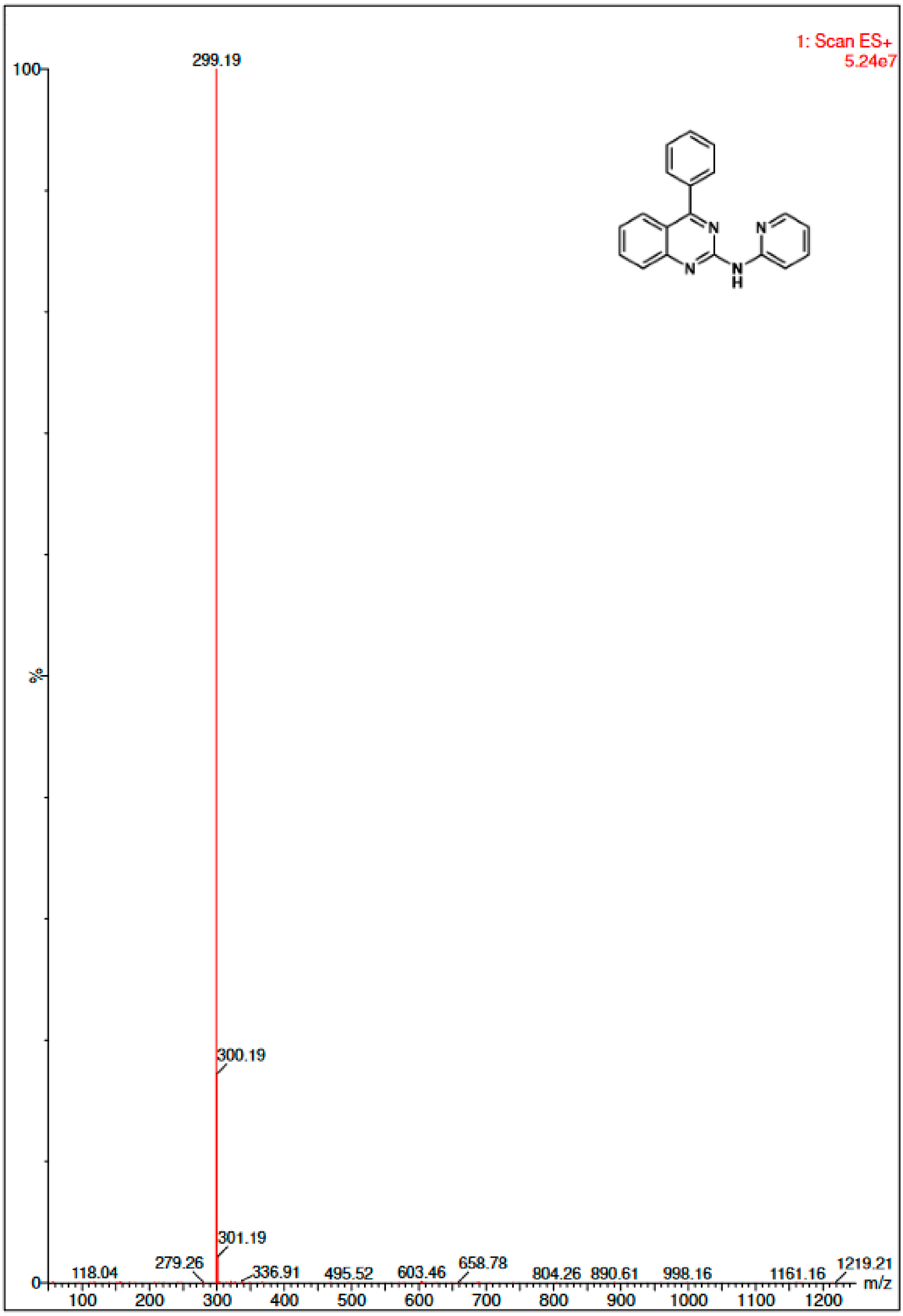

**1H-NMR Spectra in DMSO-d6 and 13C-NMR Spectra in $CDCl_3$ of compound 10b**

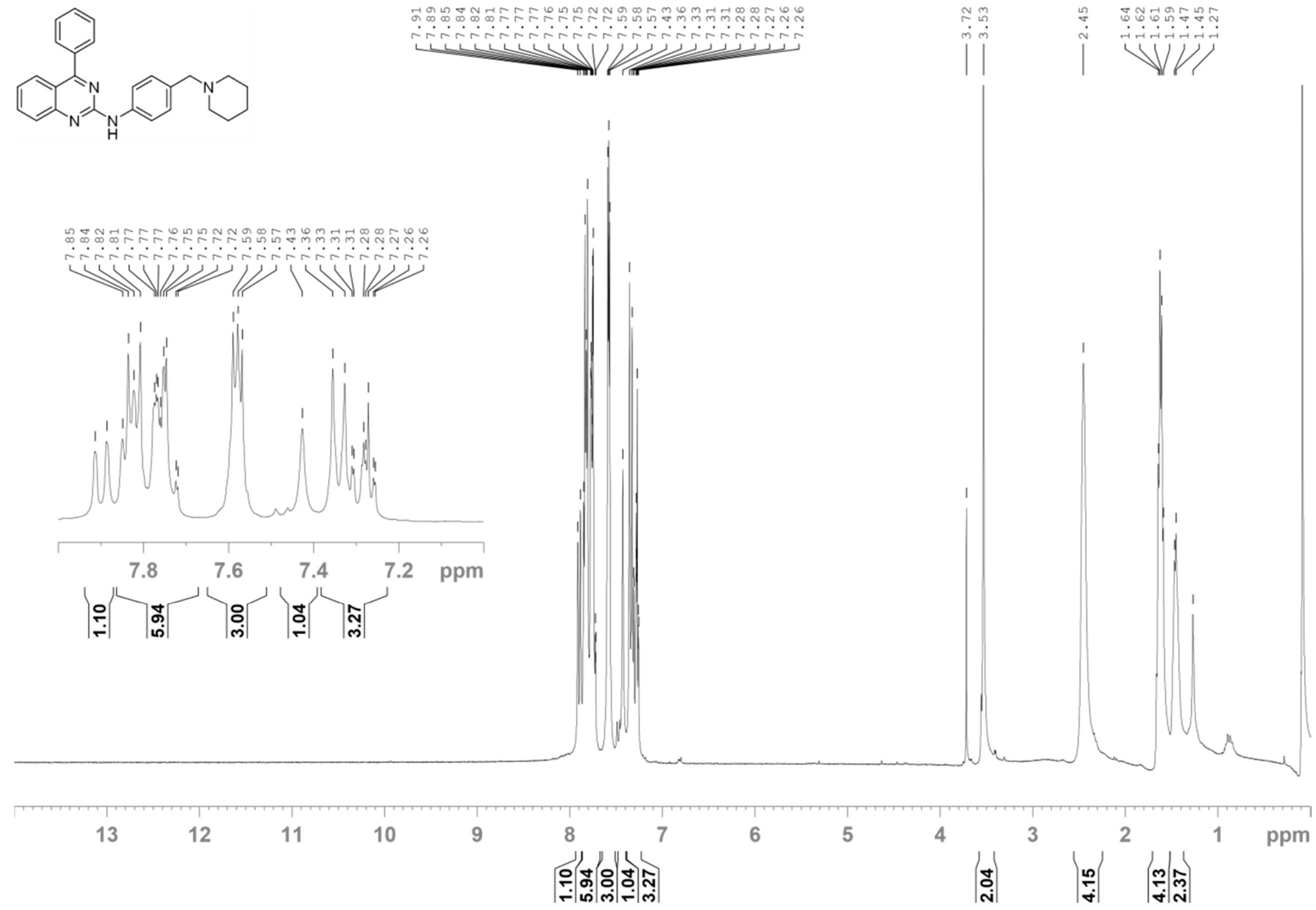

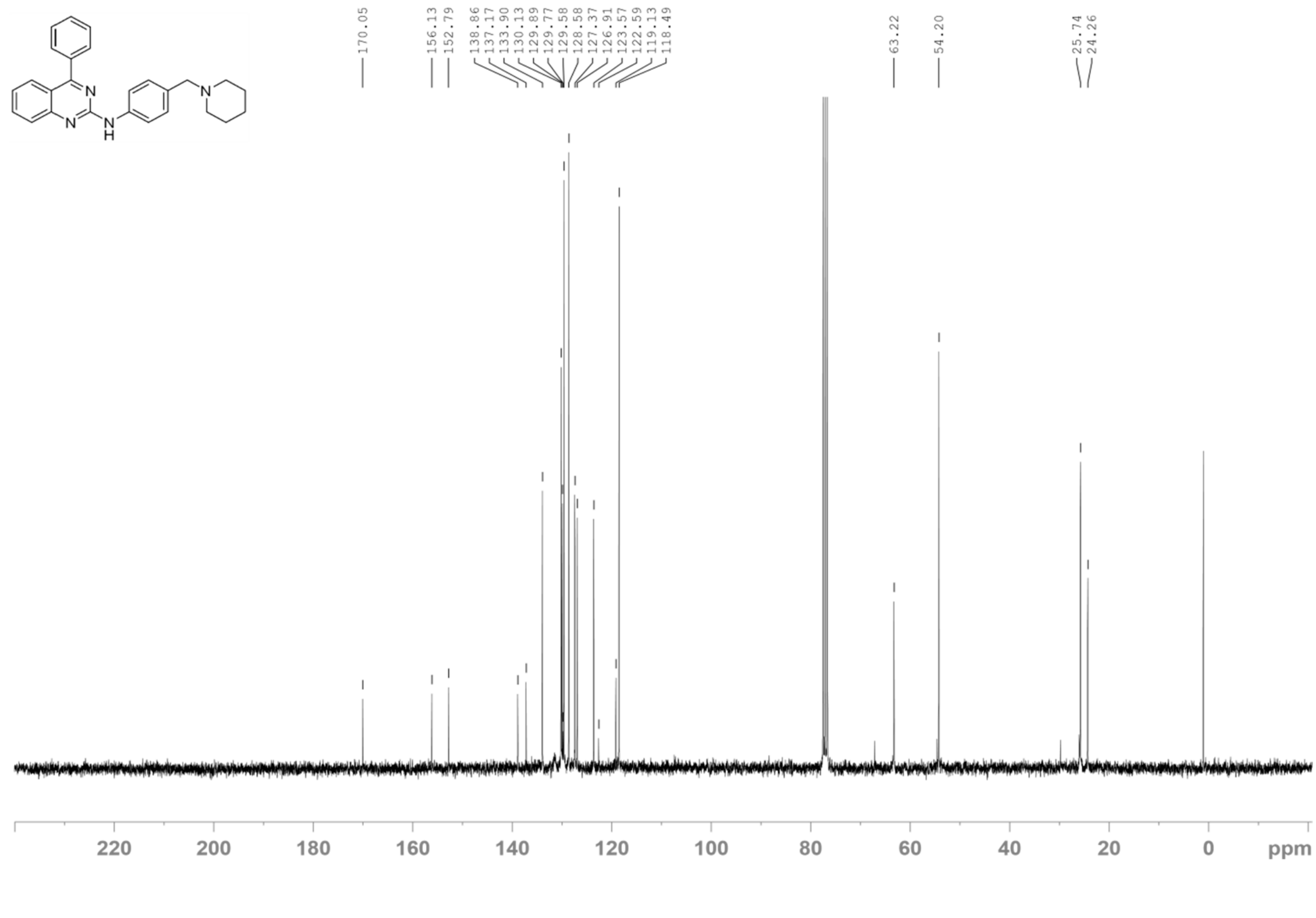

LC-MS mass spectrum of compound 10b

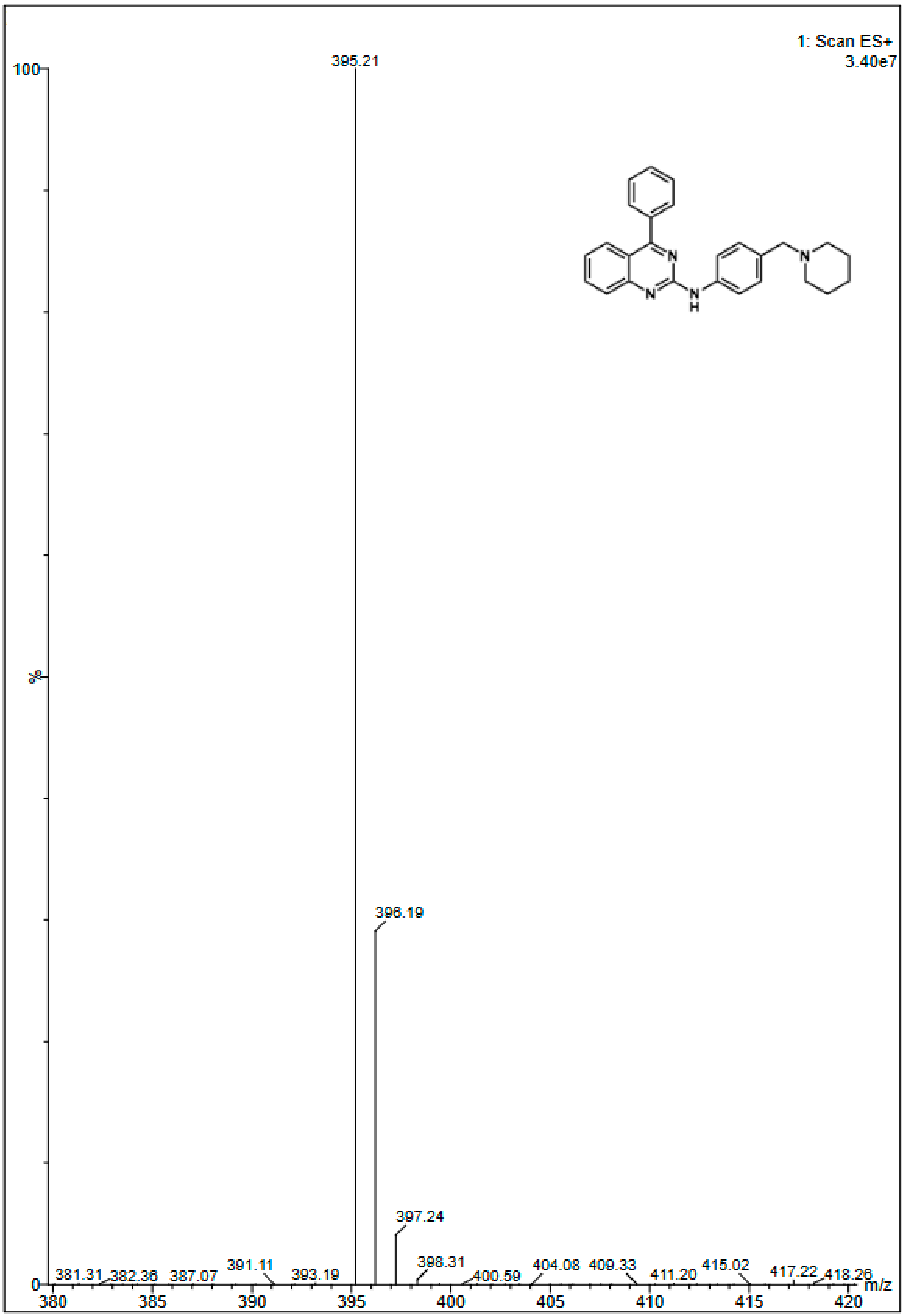

**1H- and 13C-NMR Spectra in $(CD_3)_2CO$ of compound 10c**

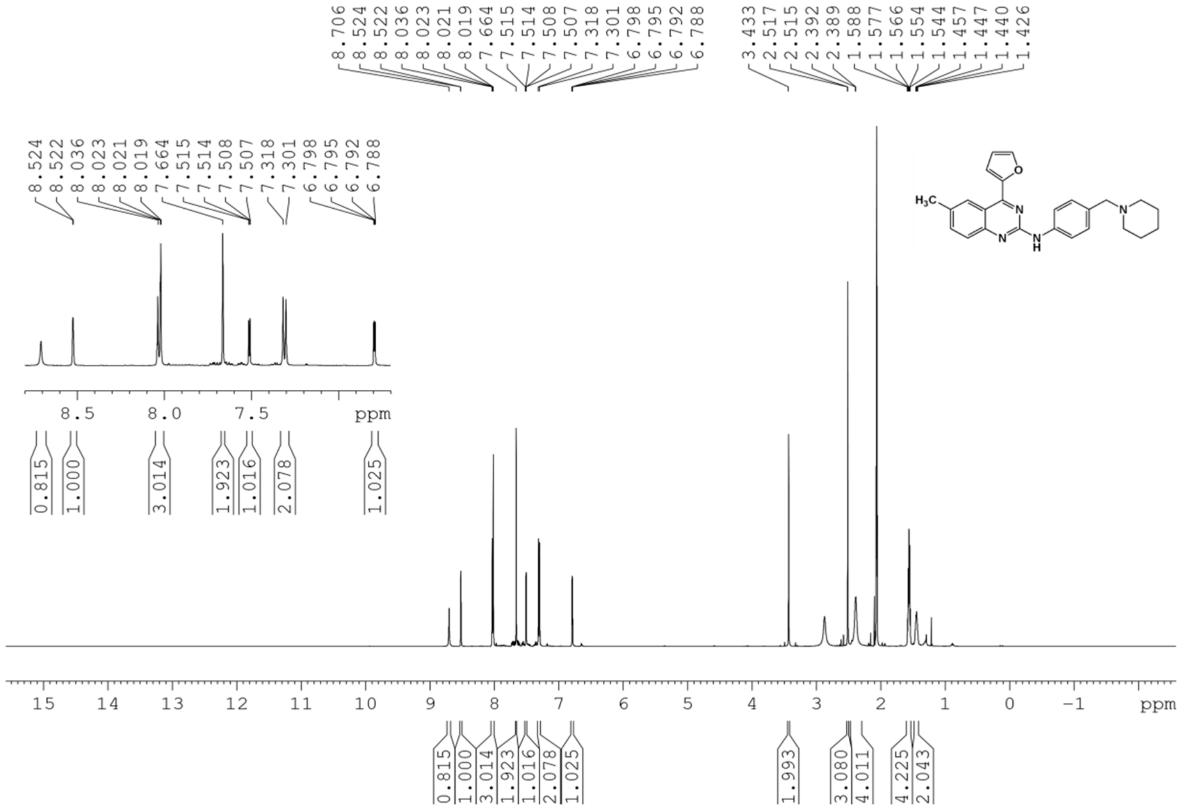

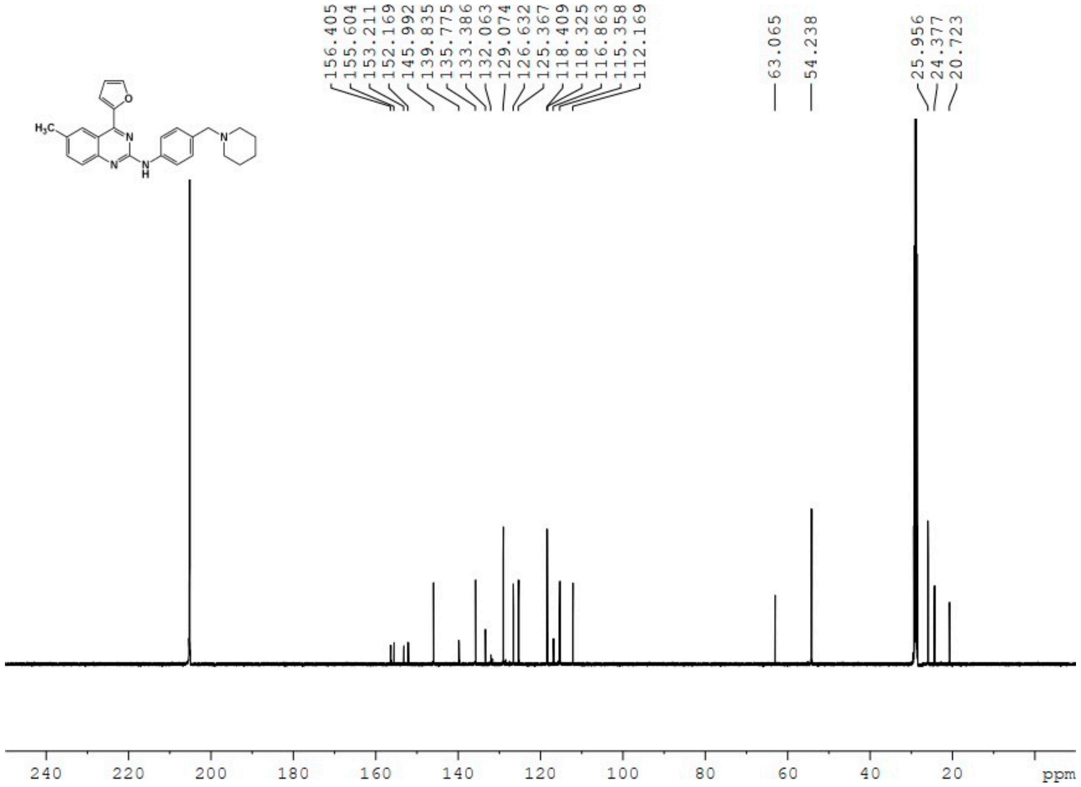

**LC-MS mass spectrum of compound 10c**

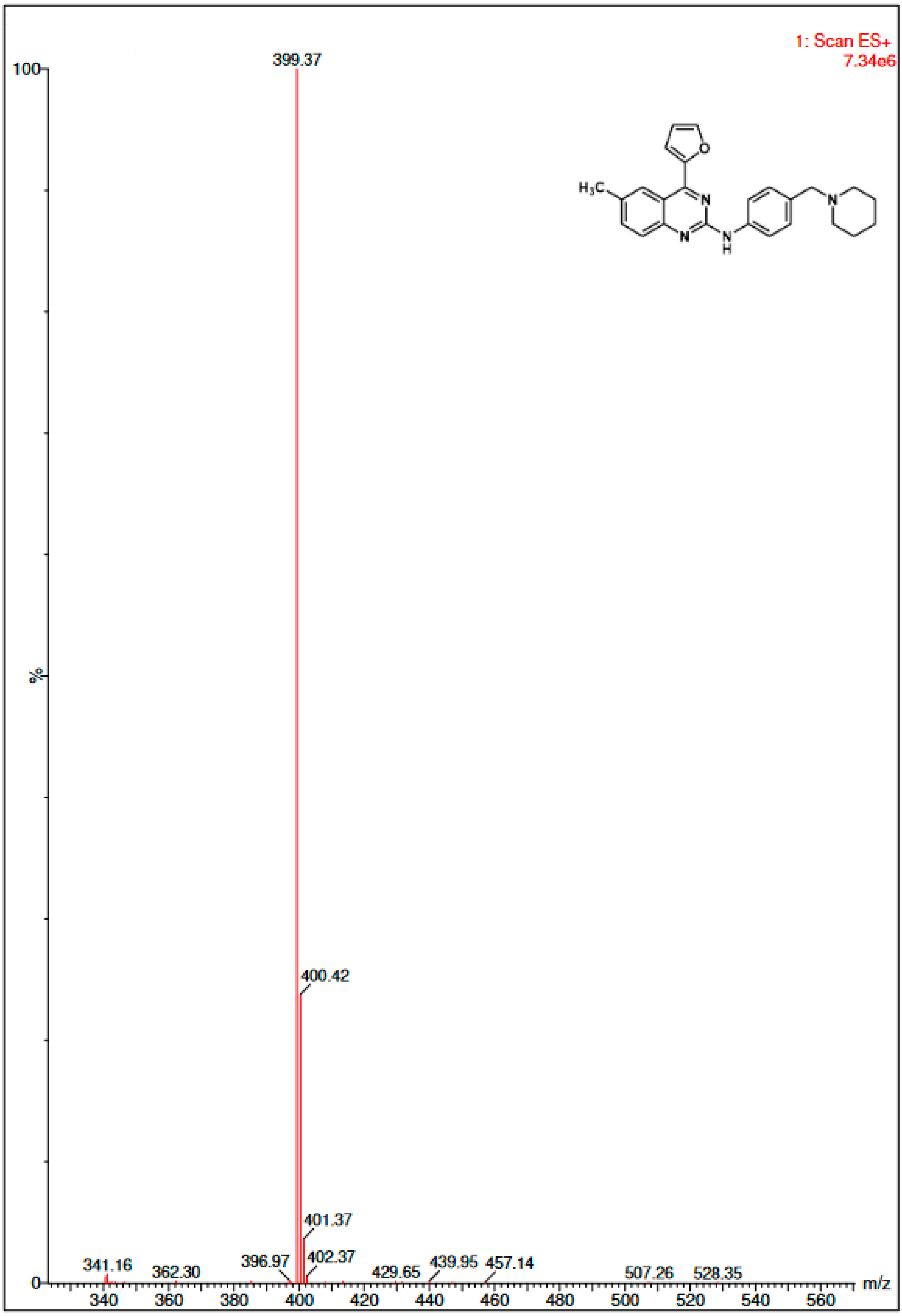

**1H- and 13C-NMR Spectra in $CD_3OD$ of compound 10d**

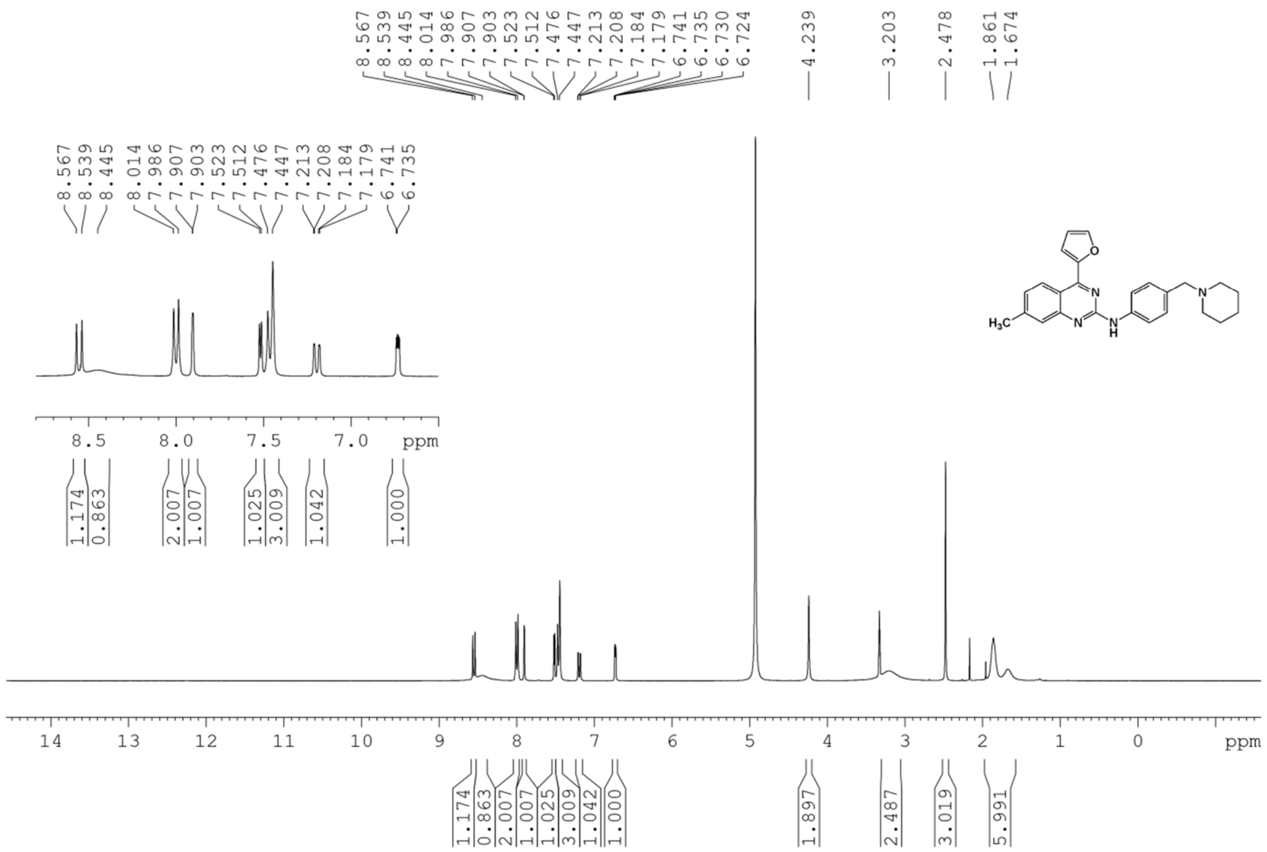


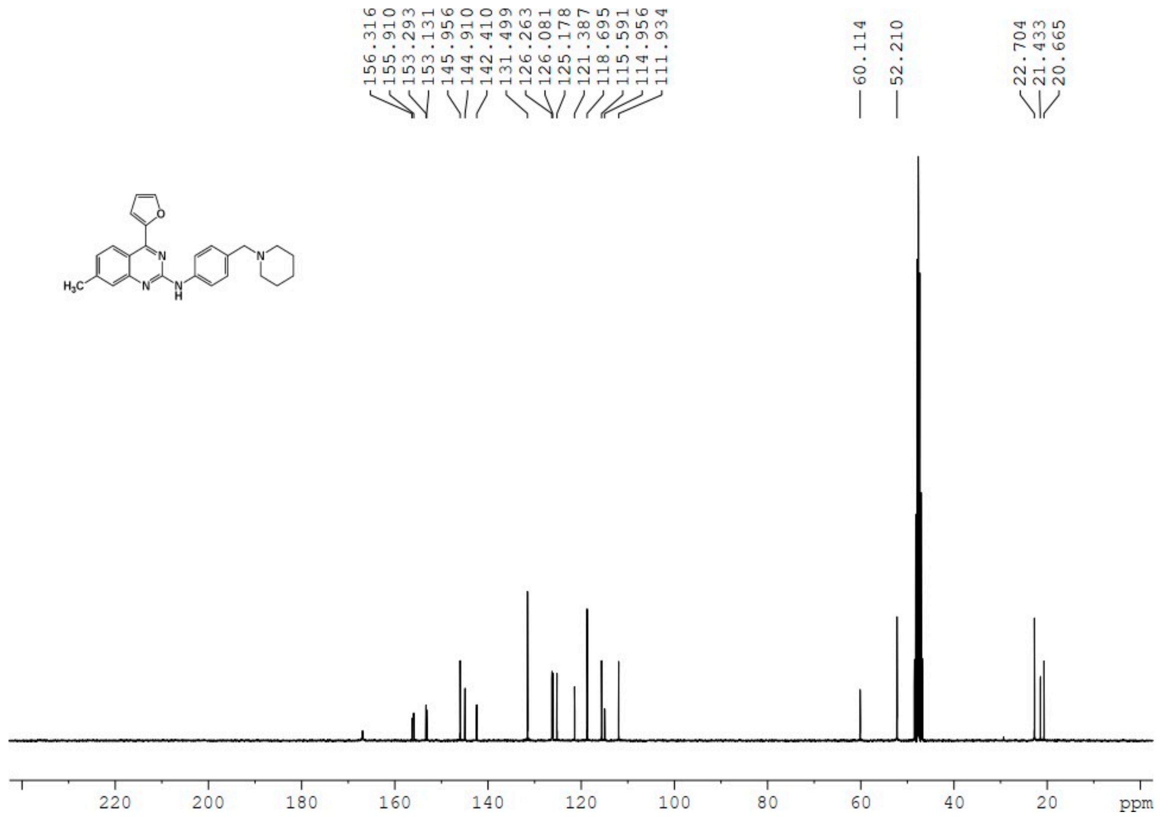

**LC-MS mass spectrum of compound 10d**

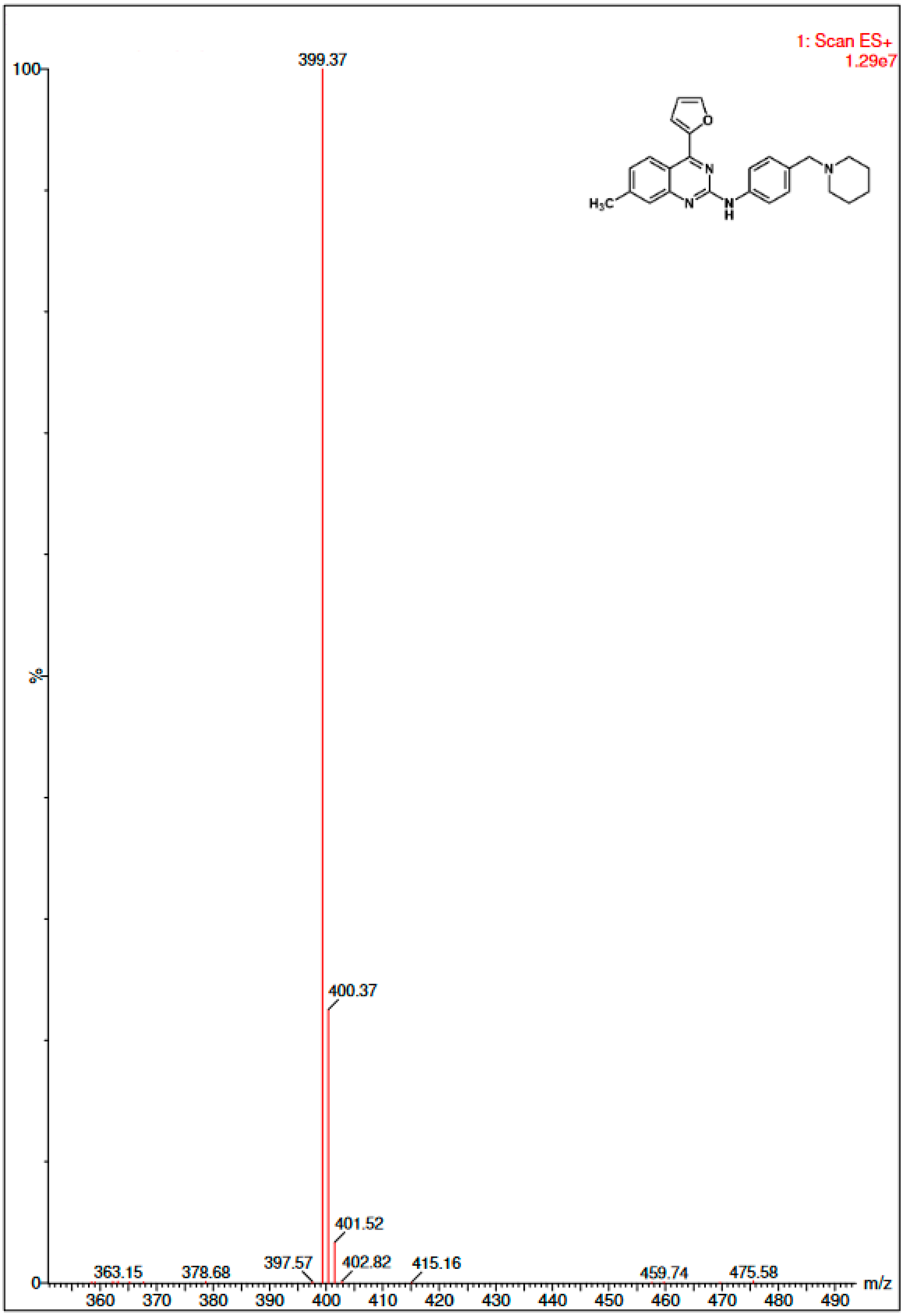

**Figure S2. Dose-response curve of ZM241385**

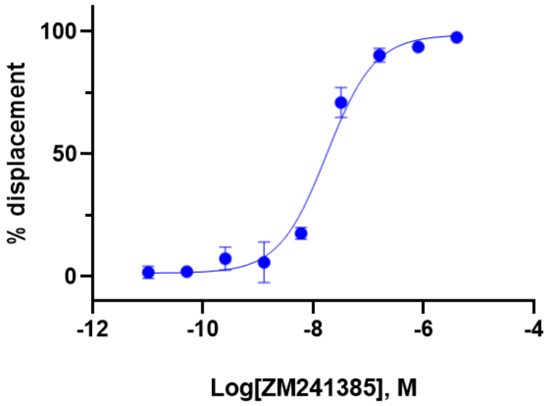

**Figure S3. FP competition binding experiments with compound 1 and some final compounds**

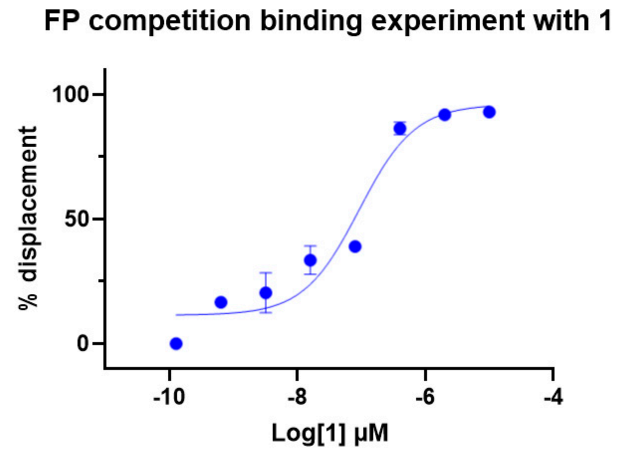


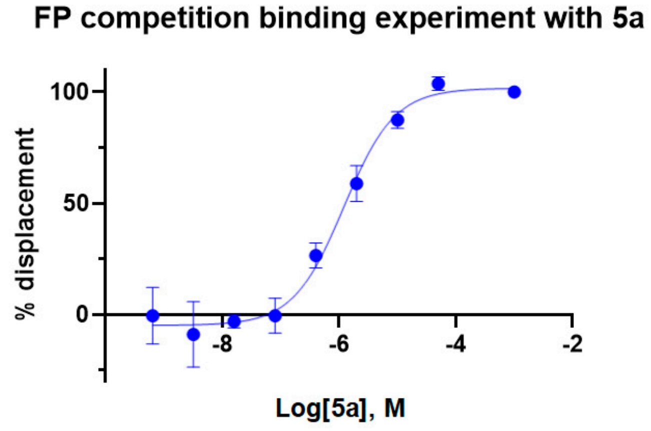


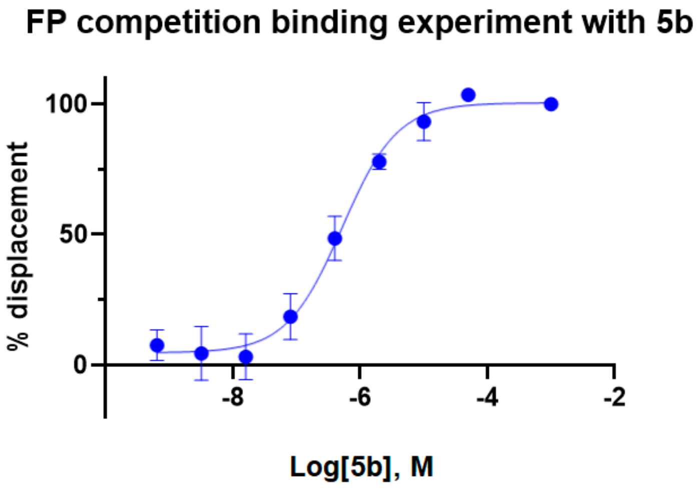

FP competition binding experiment with 5c

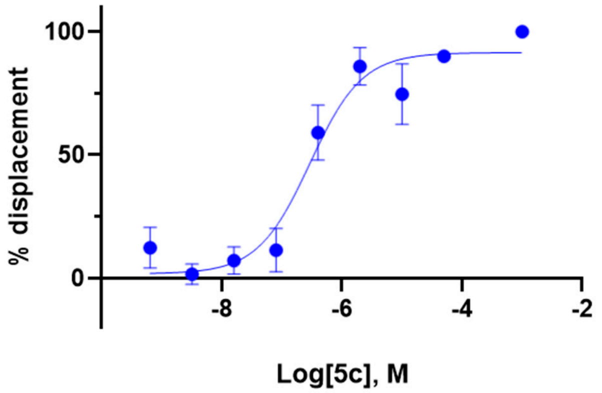


FP competition binding experiment with 5d

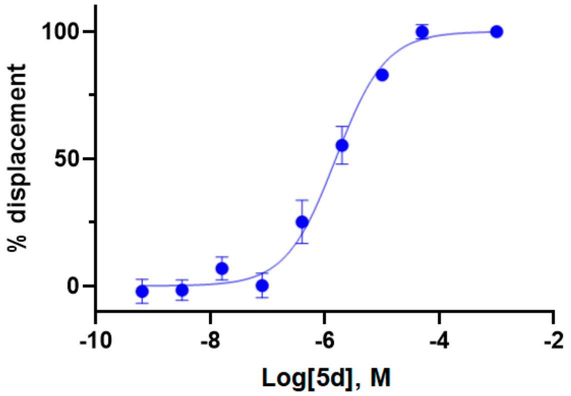


FP competition binding experiment with 5e

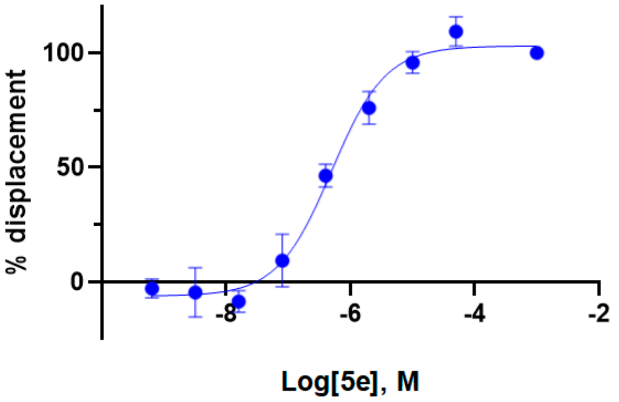

FP competition binding experiment with 5h

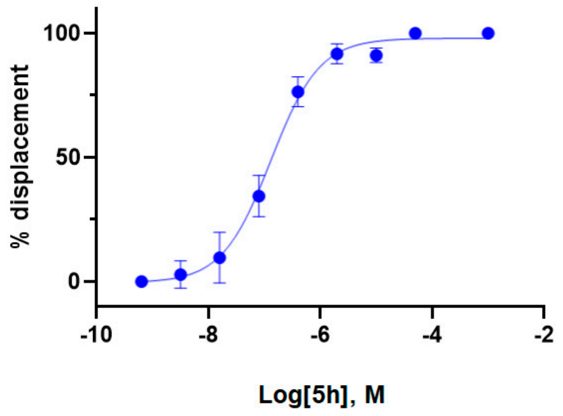


FP competition binding experiment with 5j

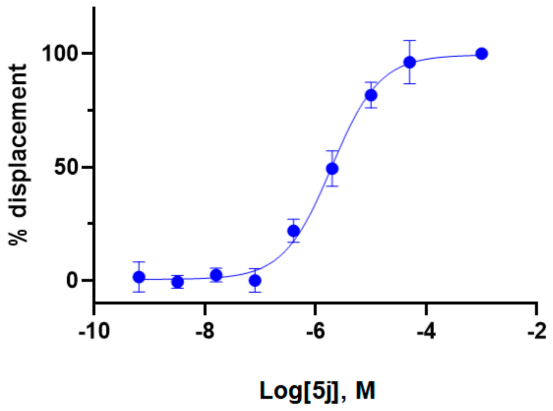


FP competition binding experiment with 5k

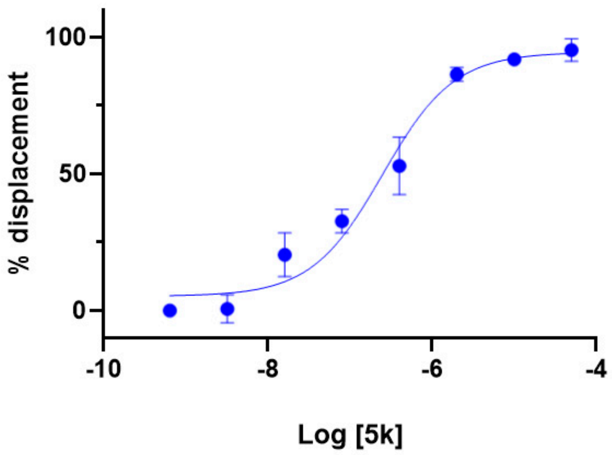

## FP competition binding experiment with 5l

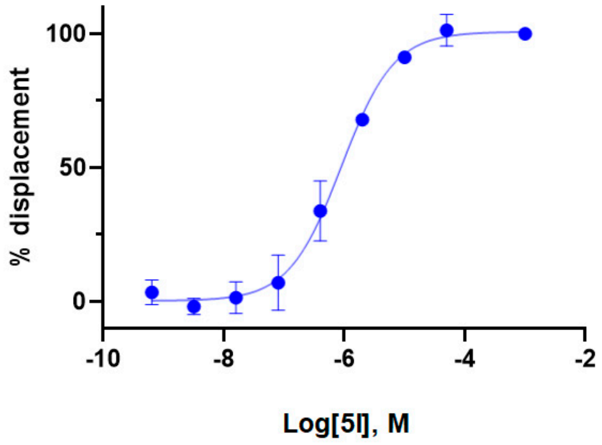


## FP competition binding experiment with 5m

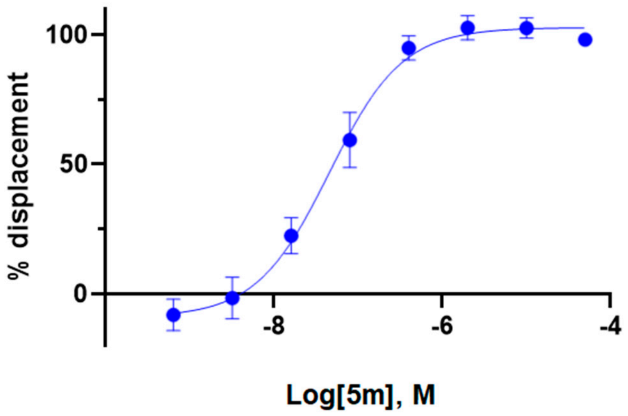


## FP competition binding experiment with 5n

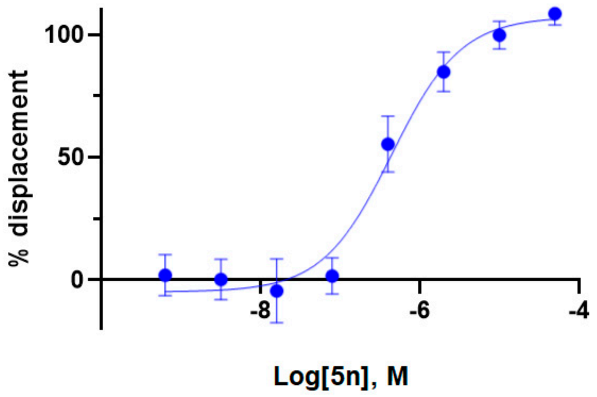

**FP competition binding experiment with 5o**

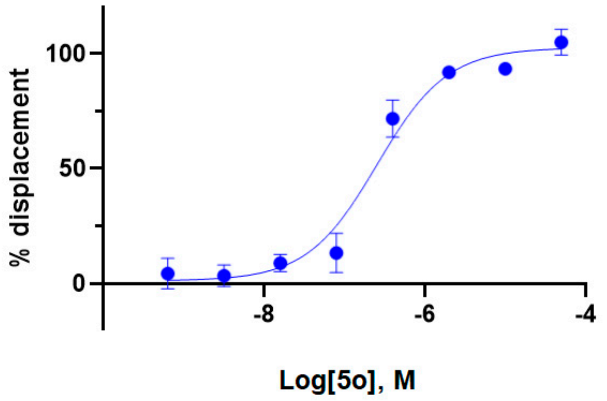


**FP competition binding experiment with 5p**

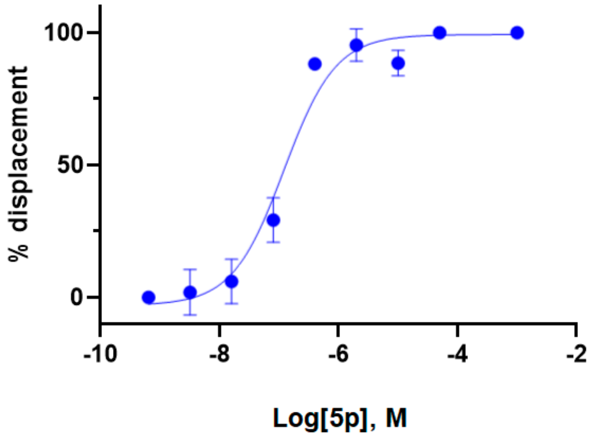


**FP competition binding experiment with 9a**

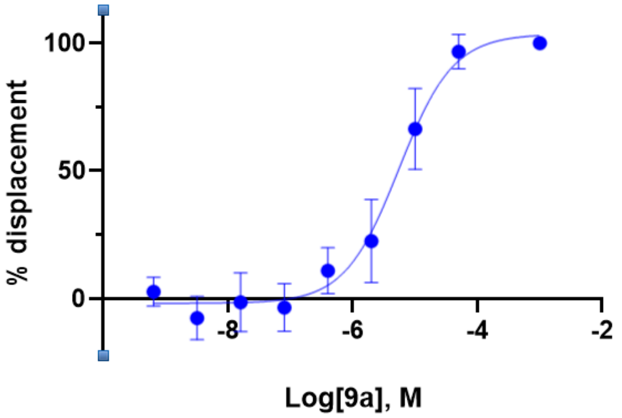

FP competition binding experiment with 9f

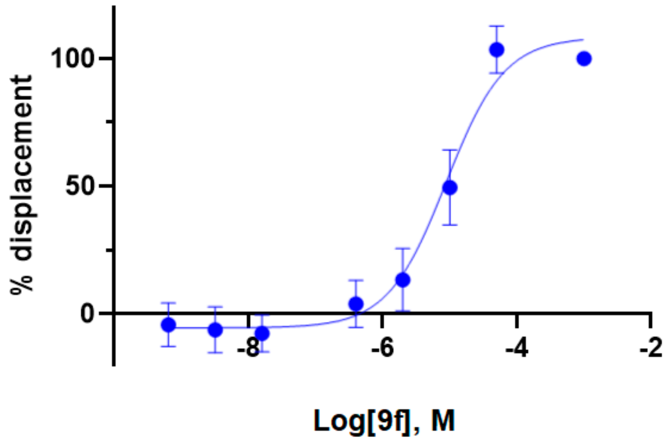


FP competition binding experiment with 9g

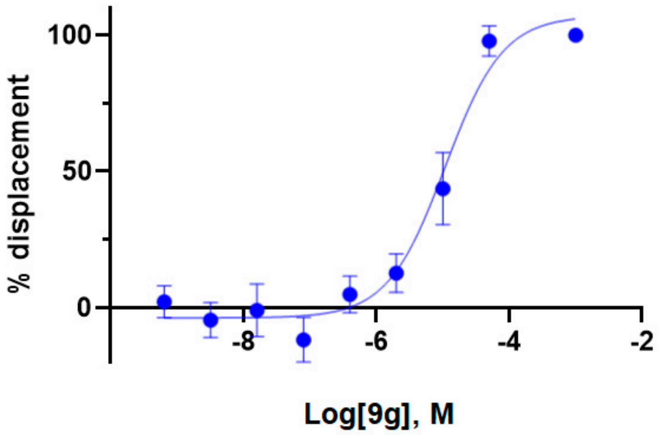


FP competition binding experiment with 9h

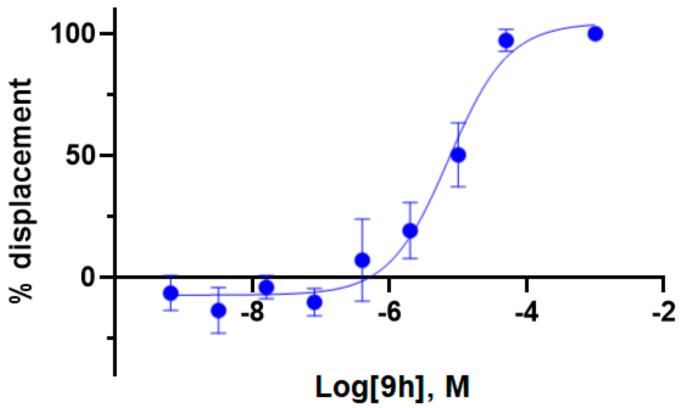

FP competition binding experiment with 9i

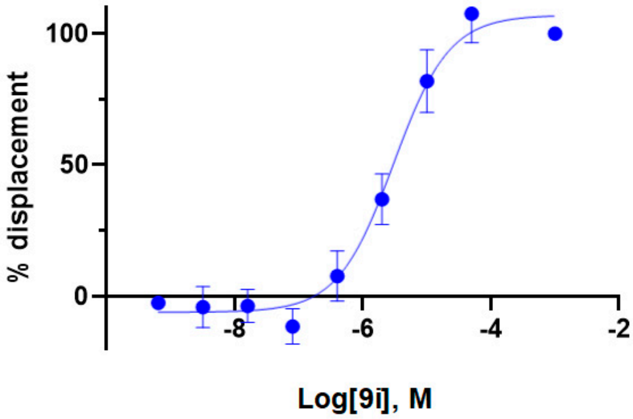


FP competition binding experiment with 9j

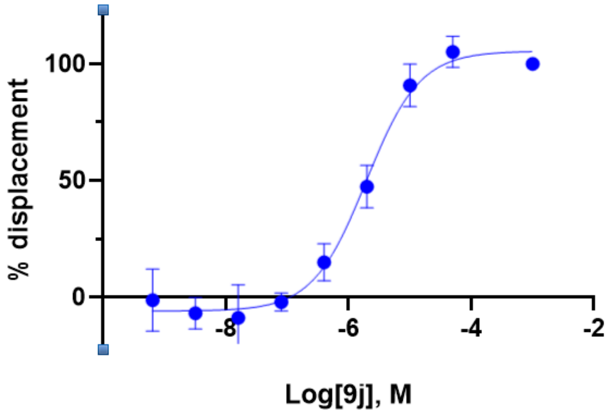


FP competition binding experiment with 9k

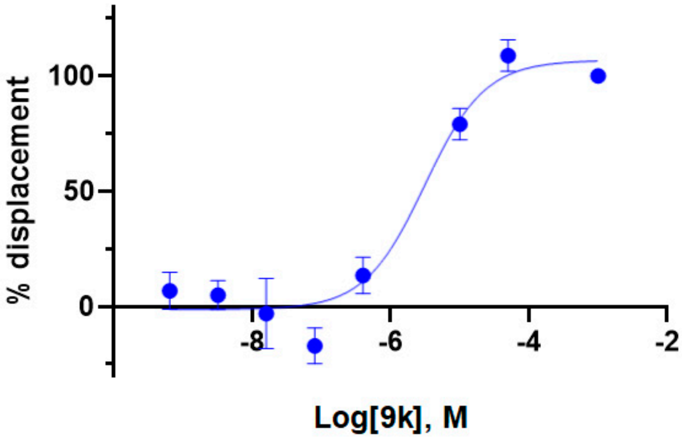

**FP competition binding experiment with 9l**

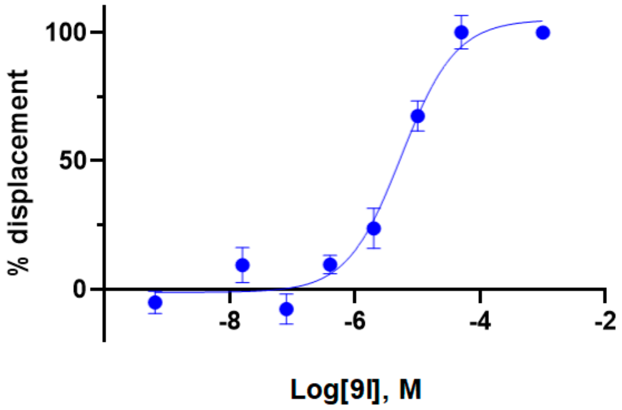


**FP competition binding experiment with 9m**

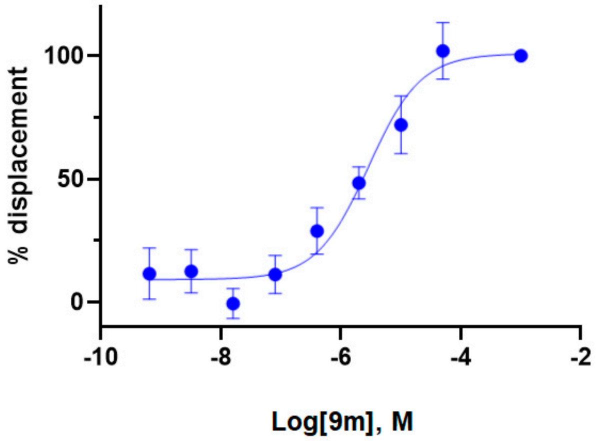


**FP competition binding experiment with 9n**

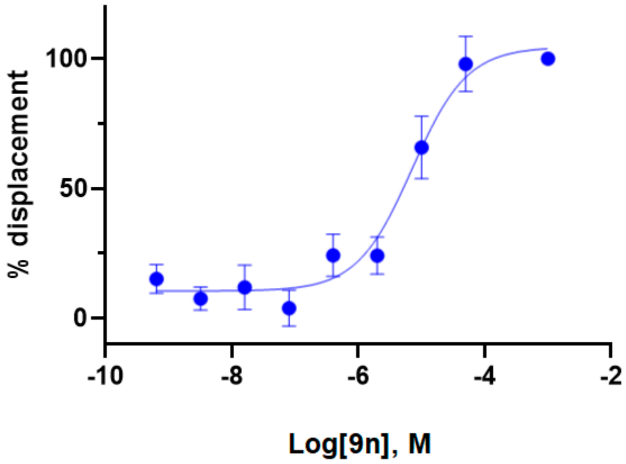

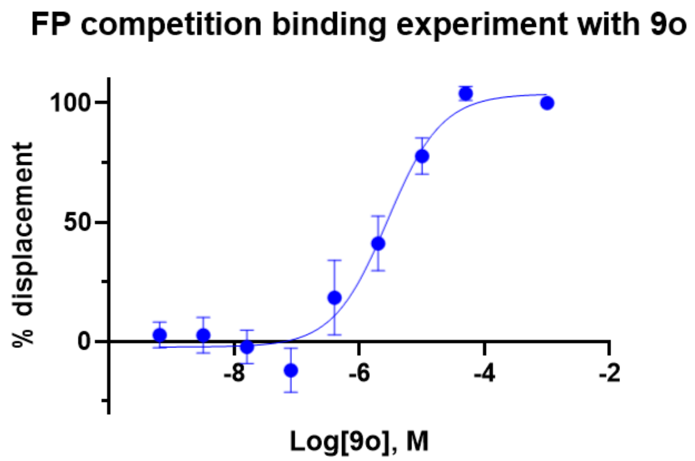
FP competition binding experiment with 9o
% displacement
100
50
0
-8
-6
-4
-2
Log[9o], M

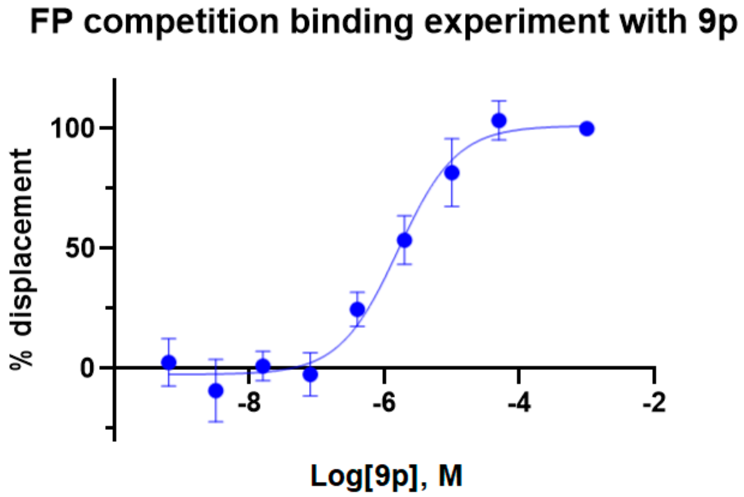
FP competition binding experiment with 9p
% displacement
100
50
0
-8
-6
-4
-2
Log[9p], M

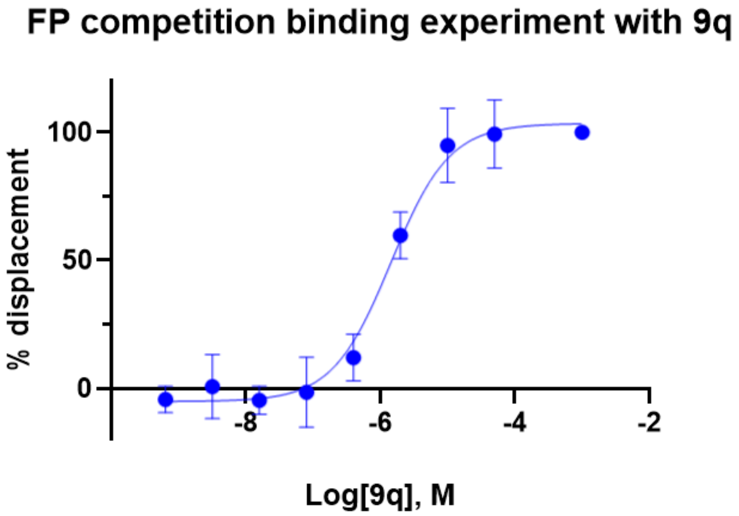
FP competition binding experiment with 9q
% displacement
100
50
0
-8
-6
-4
-2
Log[9q], M

**FP competition binding experiment with 9r**

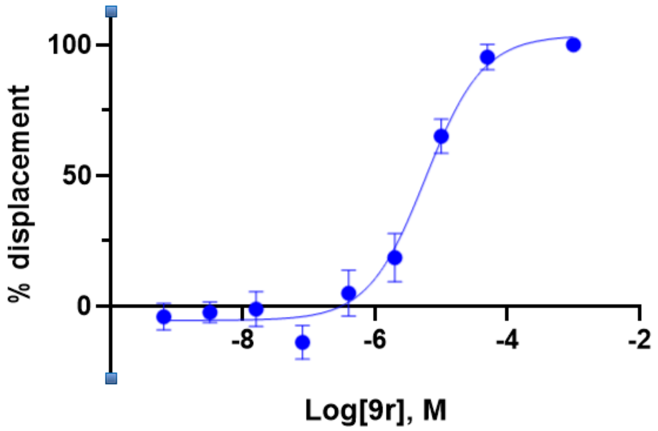


**FP competition binding experiment with 9t**

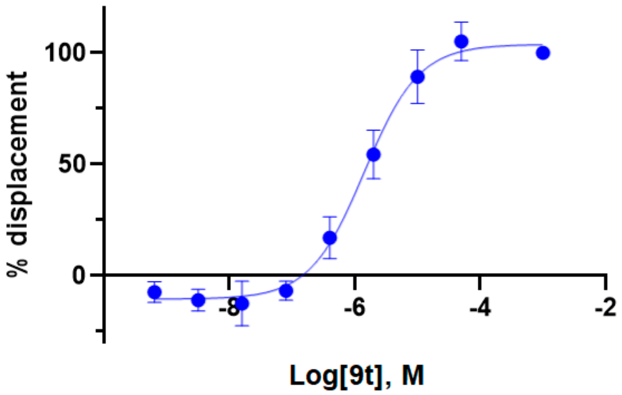


**FP competition binding experiment with 9u**

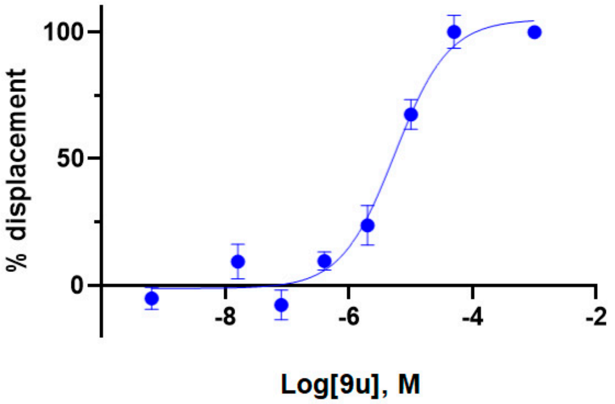

## FP competition binding experiment with 9w

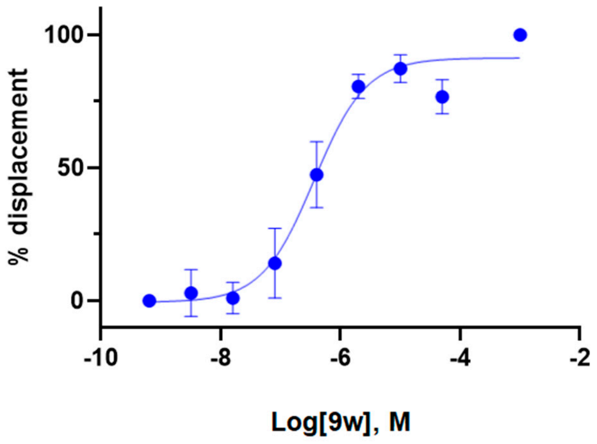


## FP competition binding experiment with 9x

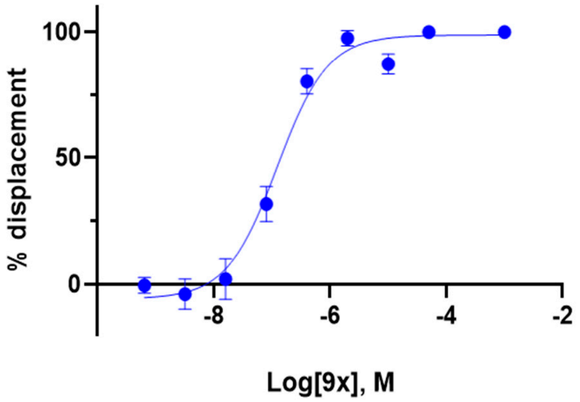


## FP competition binding experiment with 10b

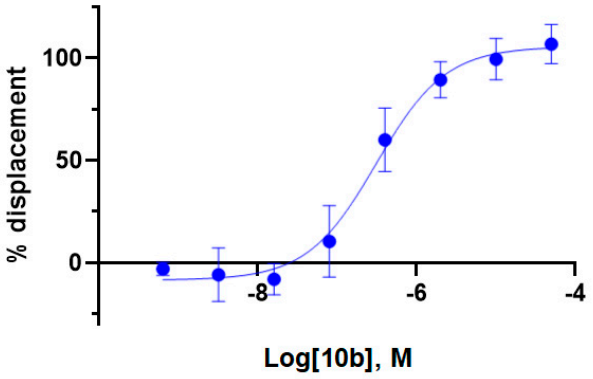

FP competition binding experiment with 10c

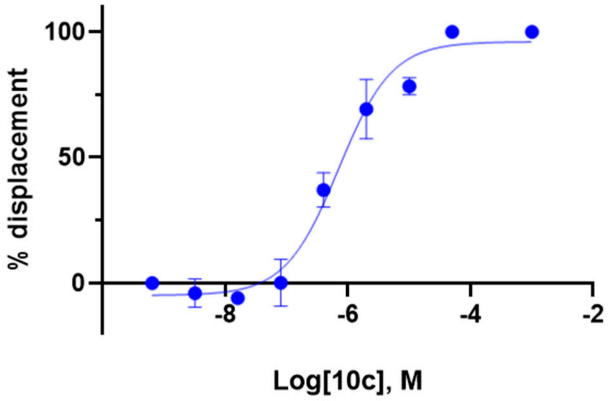


FP competition binding experiment with 10d

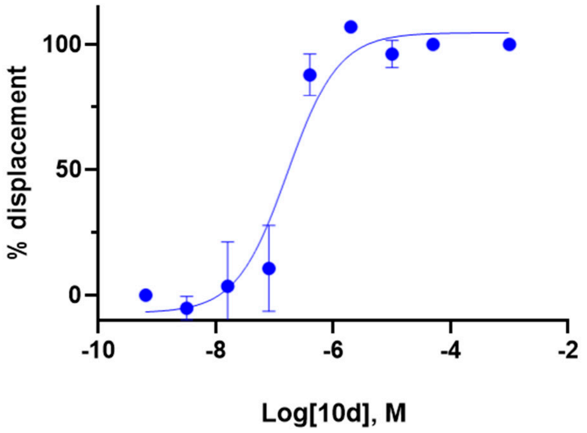


FP competition binding experiment with 11

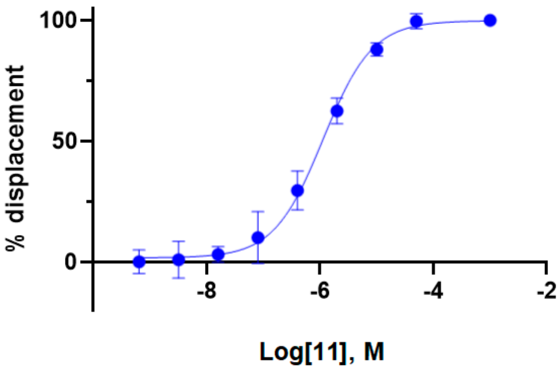

**Figure S4. cAMP assays of compounds 5m, 9w, 9x, 10c, 10d**

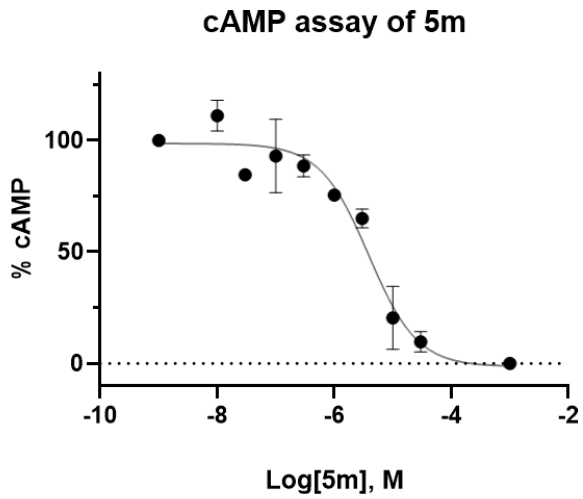


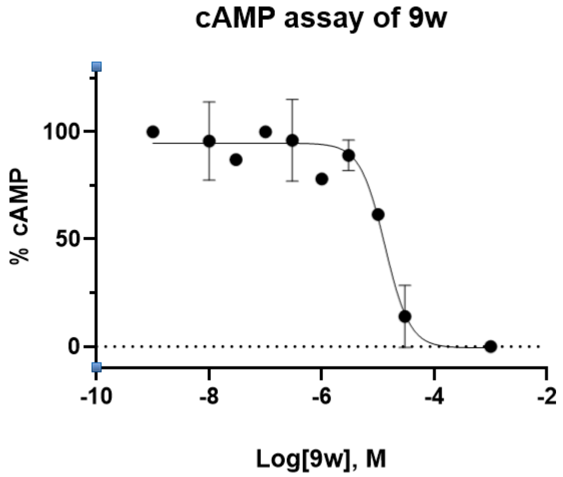


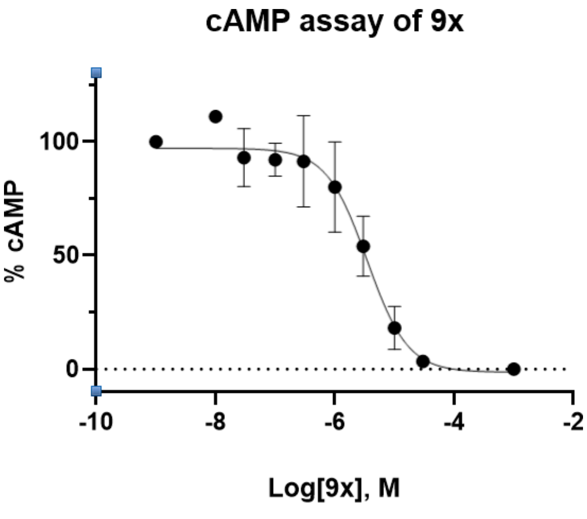

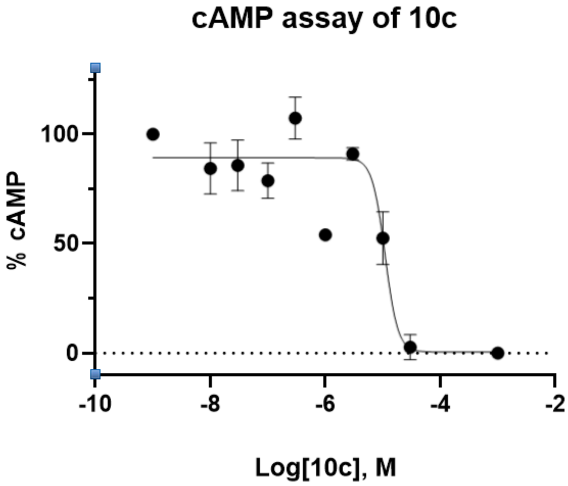
cAMP assay of 10c
% cAMP
100
50
0
-10
-8
-6
-4
-2
Log[10c], M

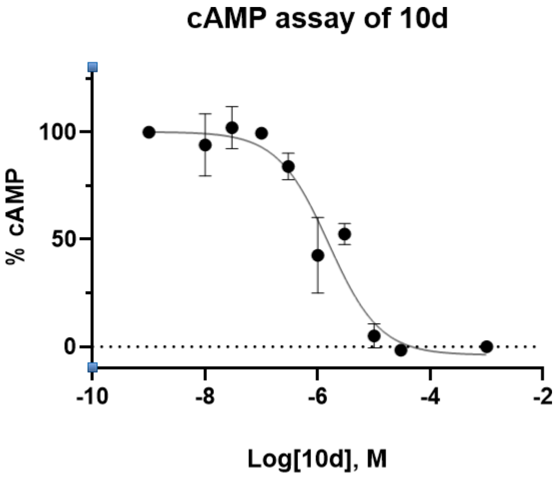
cAMP assay of 10d
% cAMP
100
50
0
-10
-8
-6
-4
-2
Log[10d], M
